# Phosphoryl Radicals from Trivalent Iminyl Phosphines: A Photocatalytic Approach to *N*‐Phosphoryl Azetidines

**DOI:** 10.1002/anie.9986925

**Published:** 2026-05-22

**Authors:** Chandu G. Krishnan, Gabriel Cormier, Stefano A. Serapian, Marco Bortolus, Luca Dell'Amico

**Affiliations:** ^1^ Department of Chemical Sciences University of Padova Padova Italy; ^2^ Department of Chemistry University of Pavia Pavia Italy

**Keywords:** azabicyclobutanes, iminyl phosphines, phosphorus radicals, photocatalysis, strain release

## Abstract

We report that iminyl phosphines, a class of bench‐stable and readily accessible trivalent P‐centered radical precursors, enable the synthesis of N‐phosphoryl azetidines via formation of a key phosphoryl radical under visible‐light single‐electron transfer (SET) catalysis. Mechanistic investigations reveal that SET oxidation of the iminyl phosphine engages a previously unexplored SET‐triggered polar pathway that channels the reaction away from classical phosphine oxidation toward the controlled generation of a P‐centered radical. A combination of experimental evidence, including electron paramagnetic resonance (EPR) spectroscopy and optical studies, supported by density functional theory (DFT) calculations, were used to shed light on this unconventional reaction manifold. This cooperative radical–polar mechanism facilitates the radical ring‐opening of azabicyclo[1.1.0]butanes (ABBs), granting access to previously inaccessible P–N bonds.

## Graphical abstract

Photochemically generated phosphoryl radicals from iminyl phosphines enable strain‐release addition to azabicyclobutanes, providing access to elusive N‐phosphoryl azetidines. Combined experimental, computational, and EPR studies reveal an unconventional water‐assisted photoredox pathway.

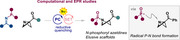

## 1 Introduction

**FIGURE 1:**
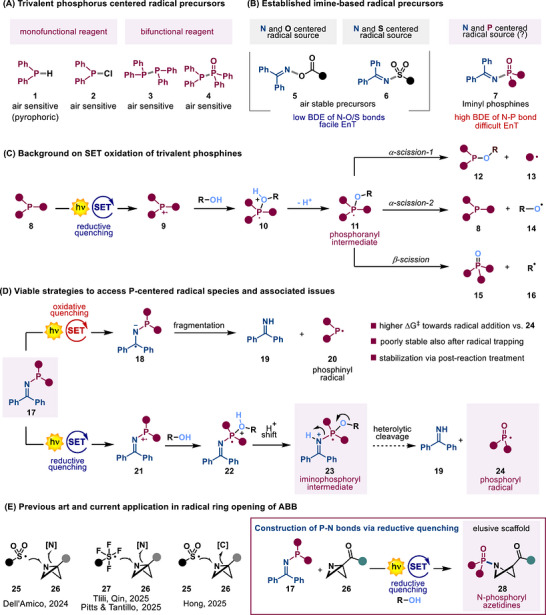
Development and application of stable trivalent phosphorus‐centered radical precursors. (A) Conventional limits of trivalent P‐radical precursors. (B) Imine based radical precursor and EnT limits for iminyl phosphines **7**. (C) Background on SET oxidation of P(III) compounds and possible reaction outcomes. (D) Challenge associated with oxidative quenching cycle for **7** (top). Our Strategy: Merged SET/polar process to access P‐radical **24** (bottom). (E) Current art for strain‐release of azabicyclo[1.1.0]butanes (left). This work: SET‐enabled N‐phosphoryl azetidines synthesis via P‐N bond formation with **17** (right).

Organophosphorus compounds are integral to a wide range of applications, from pharmaceuticals [1] and agrochemicals [2] to functional materials [3] as well as ligands in transition‐metal catalysis [4]. As such, the development of efficient and selective methods for P‐heteroatom bond formation, particularly under mild and functional‐group‐tolerant conditions, remains a longstanding objective in synthetic chemistry [5]. In recent years, photoredox catalysis has gained prominence as a powerful platform for generating phosphorus‐centered radicals, especially phosphoryl (R_2_P(═O)·) [6] and phosphinyl (R_2_P·) [7] species, useful in radical‐mediated phosphorylation reactions. However, despite substantial progress, these methods are still constrained by several limitations: (i) a narrow pool of structurally diverse P(III)‐radical precursors (**1**‐**4**) [6, 7], (ii) the dependence on air‐ and moisture‐sensitive (**1**‐ **4**), pyrophoric or toxic reagents (**1**), which often necessitate the use of a glove‐box (Figure 1A) [8, 9, 10, 11], and (iii) the need for post‐reaction treatment with crystalline sulfur [8, 9, 10, 11, 12, 13, 14, 15], or oxidizing reagents (*m*‐CPBA, H_2_O_2_) [9, 10, 11, 16, 17] when employing trivalent phosphorus precursors. Furthermore, these reagents are often used with transition‐metal salts and organometallic reagents, raising concerns about cost, and environmental sustainability of these processes [6, 7, 16, 17]. Recent advances in energy‐transfer (EnT) photochemistry have shown that oxime carbonates and esters **5** [18, 19], as well as sulfonamides **6** [20, 21, 22, 23], can be used to the generation of N, O, and S‐centered radicals (Figure 1B, left). The utility of these radical precursors arises from their relatively low bond dissociation energies (BDEs) and unique electronic properties, such as electron‐deficient nitrogen centers and the lone‐pair repulsion between nitrogen and adjacent heteroatoms (O, S) [24]. In stark contrast, the development of analogous bifunctional reagents **7** featuring nitrogen–phosphorus (N–P) bonds has proven considerably more challenging (Figure 1B, right) [25]. The primary obstacle lies in the remarkably high bond strength of the N–P linkage, which resists homolytic cleavage. Even when phosphorus‐ centered radicals are generated successfully through direct photoexcitation or EnT, their reactivity remains hindered by fast radical recombination [26]. For this reason, potential of N–P bond for radical transformation still remains as an underexplored area in photocatalysis unlike other N‐heteroatom bonds (O, S).

Literature evidence demonstrates that strategies based on SET oxidation of phosphines **8** are inherently challenging (Figure 1C), because the resulting phosphoranyl radical intermediates **11** preferentially undergo α‐scission‐1, α‐scission‐2, or more favorable β‐scission pathways, leading predominantly to neutral phosphine products **12**, **8**, and **15**, respectively [27, 28]. Taking this into consideration, we sought to develop an alternative strategy to activate P‐heteroatom bonds in iminyl phosphines **17** via single electron transfer (SET) approach (Figure 1D). We initially evaluated an oxidative quenching manifold. However, there are still quite a few roadblocks and challenges to successfully access P‐centered radicals from P(III) precursors. Prior studies suggest that the phosphinyl radical **20** has very low reactivity toward alkenes and strained cyclic compounds and is characterized by high activation energy barriers (ΔG^‡^) compared to the phosphoryl radical **24** [9, 10, 11]. This reduced reactivity may limit the effectiveness of these scaffolds when employed via an oxidative quenching (Figure 1D, top). Further, even if such a reaction would proceed, a subsequent oxidation step is needed to render the products stable and readily isolable [8, 9, 10, 11, 12, 13, 14, 15]. Building on recent studies that demonstrate the water activation of phosphine radical cations **9** and their utility as tunable intermediates for hydrogenation of alkenes [29] and to synthesize P‐chiral tertiary phosphine oxides (Figure 1C; R = H, β‐scission pathway) [30], we envisioned to develop an alternative activation strategy based on an opposite reductive quenching pathway. Here, iminyl phosphine **17** is oxidized via SET and followed by a polar activation mode, where the imine is acting as a leaving group thereby enabling a new route for the generation of reactive phosphoryl radical **24** (Figure 1D, bottom). However, several intrinsic constraints complicate the realization of this strategy. Foremost, the reduction potential of the iminyl phosphine **17** must fall within a regime that enables efficient photoinduced oxidation by the organophotocatalyst, while avoiding the oxidation of the substrate(s). In parallel, hydrolysis must proceed slower than the initial SET event to prevent premature degradation of the starting iminyl phosphine **17**. Finally, the reaction manifold must suppress the thermodynamically favorable phosphine oxide **7** formation, a dominant pathway that typically outcompetes productive radical generation [28, 29, 30].

To test this hypothesis, we selected azabicyclo[1.1.0]butanes (ABBs) **26** [31, 32] as model substrates. Although significant progress has been achieved in the radical ring‐opening of bicyclo[1.1.0]butanes and related [n.1.1]propellanes (*n* = 1, 3, 4) families [33], analogous transformations of ABBs have remained largely unexplored. Recently, our group reported the radical ring‐opening of ABBs via an energy transfer (EnT) strategy employing sulfonyl imines **6** as bifunctional radical precursors (Figure 1E, left) [23]. Subsequent studies by Tlili, Qin, Pitts, Tantillo as well as Hong and co‐workers have marked further advances in ABB activation, while demonstrating the high interest and potential of this chemistry [34, 35, 36, 37]. Despite these advances, existing ABB radical ring openings are only confined to S‐centered radicals (**25** and **27**) [23, 34, 35, 36, 37], whereas the addition of P‐centered radicals to ABBs via an unconventional P‐N bond forming radical process will grant access to *N*‐phosphoryl azetidines **28** (Figure 1E, right). Given the scarcity of these motifs and the lack of general synthetic methods for their preparation [38, 39], we envision that the iminyl phosphines **17** could serve as robust, bench‐stable, phosphorus‐centered radical precursors useful for a variety of chemical transformations.

## 2 Results and Discussion

### 2.1 Reaction Development

We commenced our investigation by synthesizing iminyl phosphines **29**, a bench stable and easily accessible source of P‐centered radicals [40]. Subsequent oxidation of **29** with elemental sulfur [8, 9, 10, 11, 12, 13, 14, 15] yielded the corresponding phosphine sulfide **30**. Further oxidation of **30** with *meta*‐chloroperbenzoic acid (*m‐*CPBA) [9, 10, 11] afforded the phosphine oxide **31** as a yellow solid. (See Supporting Information, section 2.0). Preliminary studies involved determining the triplet energy (T_1_) and spin distribution for compounds **29**, **30,** and **31** utilizing the freely accessible EnT decker machine learning‐based (ML) platform [41]. Bond dissociation energies (BDEs) were calculated through density functional theory (DFT) at the UωB97X‐D/Def2‐SVP level of theory in DCM, employing the SMD solvation model [10, 11].

**FIGURE 2:**
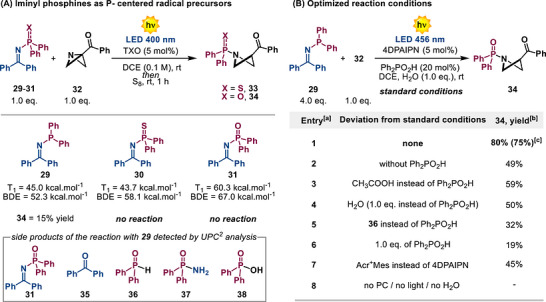
Iminyl phosphines as P‐ centered radical precursors. (A) Preliminary studies on possible P‐radical precursors. (B) Optimized reaction condition. ^[a]^Reaction performed at 0.1 mmol scale, **29** (4.0 eq.), **32** (1.0 eq.), under 456 nm LED irradiation, see Supporting Information, section 3.2. ^[b]1^H NMR yield using 1,3,5‐trimethoxybenzene as internal standard. ^[c]^Isolated yield.

This approach facilitates the assessment of dissociation probabilities under specific energy transfer conditions, particularly when the substrate's BDE is less than or equal to T_1_ values [42]. According to the model predictions, compounds **29**, **30**, and **31** possess triplet energies of 45.0, 43.7, and 60.3 kcal·mol^−1^, respectively (Figure 2A), which are within the accessible range of commonly used photosensitizers such as thioxanthone (TXO; *T*_1_ = 65.5 kcal·mol^−1^) [42]. However, the calculated BDEs for these compounds are 52.3, 58.1, and 67.0 kcal·mol^−1^, respectively, exceeding their predicted *T*_1_ values. Our experimental focus was directed towards validating these results for the radical ring opening of azabicyclo[1.1.0]butanes (ABBs). Specifically, iminyl phosphine **29** (1 eq.) was subjected to irradiation with a 400 nm LED light source in the presence of TXO (5 mol%) as photocatalyst, along with the model azabicyclobutane **32** (1.0 eq.). At the end of the reaction, the mixture was treated with 1.2 equivalents of sulfur under an argon atmosphere to oxidize the phosphine‐to‐phosphine sulfides, thereby facilitating the purification of products (Figure 2A). The formation of N‐thiophosphoryl azetidine **33** or the difunctionalized products were not observed. Instead, compound **34** was obtained in 15% yield, The presence of other side products such as the iminyl phosphine oxide **31** and hydrolysis products such as benzophenone **35**, diphenyl phosphine oxide **36**, diphenylphosphine amide **37** and diphenylphosphinic acid **38** [43] were observed by UPC^2^ analysis. No dimers of the corresponding iminyl radical (Ph_2_═N·) [23] or phosphinyl radical (Ph_2_P·) [10] were detected in the reaction media, suggesting that those species do not form during the reaction. We then carried out the reaction of **32** with **30** and **31** under similar reaction conditions. However, both **30**, having similar T_1_ and BDEs as **29**, and **31** remain unreacted. These observations along with the mismatch between the BDEs and T_1_ values suggest the operation of a SET manifold over a classical EnT catalysis. This prompted us to conduct further screening of a library of PCs, additives and optimization of other reaction parameters such as solvent, concentration while avoiding sulfur treatment at the end of the reaction (See Tables S3–S6). Pleasingly, using diphenylphosphinic acid **38** (20 mol%), with 4DPAIPN (5 mol%) in 1,2‐dichloroethane (DCE, 0.05 M) under blue LED irradiation (456 nm) afforded N‐phosphoryl azetidine **34** with 80% ^1^H NMR yield and a 75% isolated yield (Figure 2B, entry 1). Omitting additive **38** resulted in a reduced yield of 49% (entry 2). Substituting **38** with acetic acid (entry 3) or other acids did not improve the reaction outcome (See Table S4). Replacing **38** with addition of external water (H_2_O) at 1.0 equivalent maintained the yield at approximately 50% (entry 4). Furthermore, addition of 20 mol% diphenylphosphine oxide **36** instead of **38** led to a significant decrease in yield to 32% (entry 5), likely due to its known propensity for single‐electron transfer (SET) oxidation, which can compete with the desired photochemical pathway for product formation [6]. Increasing the amount of additive **38** from 20 mol% to 1.0 equivalent promoted excessive hydrolysis of the starting material **29** (entry 6). Reaction performed with highly oxidizing Acr^+^‐MesBF_4_^−^ instead of 4DPAIPN still provided the N‐phosphoryl azetidine **34** albeit with lower yield of 45% (entry 7), while supporting the activity of a reductive quenching process. Lastly, no reaction was observed in the absence of the PC, light or water (entry 8), indicating the key presence of all these three ingredients. It is worth noting that with this approach, it is possible to directly access N‐phosphoryl azetidine **34** avoiding two conventional approaches to synthesize phosphine oxide from trivalent phosphine reagent [8, 9, 10, 11, 16, 17] such as (i) initial oxidation of phosphine‐to‐phosphine sulfides (**33**) by treatment with sulfur (ii) and subsequent oxidative exchange of the S atom to O atom via treatment with oxidizing agents like *m‐CPBA* or H_2_O_2_.

### 2.2 Mechanistic Investigations

**FIGURE 3:**
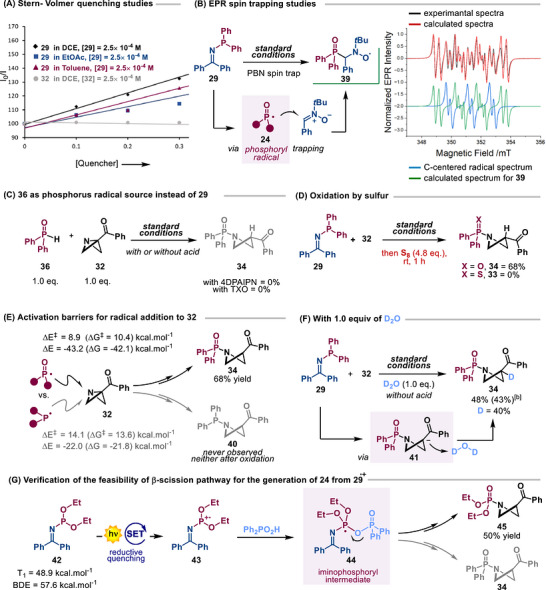
Mechanistic investigations.

**FIGURE 4:**
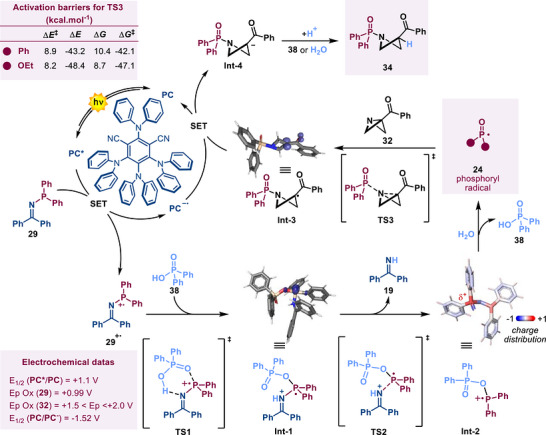
Proposed reaction mechanism. Density function theory (DFT) calculations performed at UωB97X‐D/Def2‐SVP level of theory including SMD solvation model. Distribution of the unpaired electron in **Int‐1** and **Int‐3** is shown in dark blue.

To elucidate the reaction mechanism and the roles of all the participating species, we conducted a series of mechanistic studies (Figure 3 and Supporting Information, section 4.0). We assessed the feasibility of the initial SET step between the excited 4DPAIPN* and the reaction partners such as iminyl phosphine **29** and azabicyclo[1.1.0]butane **32**, via Stern–Volmer quenching studies. Here, **29** is the sole quencher of the PC, as **32** showed no signs of quenching 4DPAIPN in DCE (Figure 3A). Consequently, changing the solvent to ethyl acetate and toluene resulted in different quenching constants and can therefore serve as evidence for an underlying SET mechanism over EnT process [42]. Although 4DPAIPN is known for its strong reducing capabilities, its exited state oxidation potential 4DPAIPN* (*E*_ox_ = +1.1 V, Figure 4, electrochemical data) [44] as well as the formation of N‐phosphoryl azetidine **34** using photocatalysts such as highly oxidizing Acr^+^‐MesBF_4_^−^ further support that the reaction proceeds through the pathway involving SET oxidation of **29**. Recorded UV/visible absorption spectra of **29**, **30,** and **31** and their mixtures indicates that the only absorbing species is **29** in the range above 427 nm (see Supporting Information, section 4.3). However, direct excitation studies without the PC showed no signs of product (see Supporting Information, section 4.4), in agreement with the activity of a SET manifold versus EnT. Proved the feasibility of the first oxidative step, we next investigated the presence of the key radical **24**. A trapping experiment with TEMPO was conducted which completely suppressed the formation of N‐phosphoryl azetidine **34** (see Supporting Information, section 4.5), although, no TEMPO‐trapping adduct were detected by UPC^2^ analysis. To gain further mechanistic insight, we conducted electron paramagnetic resonance (EPR) studies utilizing the spin‐trapping agent α‐phenyl‐tert‐butylnitrone (PBN). For this, a solution of spin trap (PBN, N‐tert‐Butyl‐α‐phenylnitrone) was incubated with the reaction mixture (the photocatalyst 4DPAIPN, **29**, **32**, and **38**). The solution was thoroughly degassed and analyzed before and after 20 h of irradiation using blue LED light (λ = 456 nm). The solution showed a modest presence of trapped radicals immediately after preparation, the amount of which increased more than ten times with irradiation. The EPR spectrum obtained after irradiation (Figure 3B—shown with the global simulation in red) shows the presence of two radicals. The dominant species (75%) can be attributed to the trapping of the phosphoryl radical **24** based on the simulation (Figure 3B– green simulation) performed with EasySpin: [45] the hyperfine couplings and *g*_iso_ (*a*_14N_ = 14.5 G; *a*_1H_ = 3.6 G; *a*_31P_ = 16.5 G; *g*_iso_ = 2.0059) are comparable to what is reported in the literature (*a*_14N_ = 14.0–14.5 G; *a*_1H_ = 2.9‐4.3 G; *a*_31P_ = 14.9‐19.0 G) [46, 47, 48, 49]. The phosphoryl radical **24** is trapped also when either **32** or **38** are not present in solution (see Supporting Information, section 4.6). However, in the second case it is much less abundant, suggesting a key kinetic role of **38** in favoring the generation of the phosphoryl radical **24**. Following confirmation of the formation of the key phosphoryl radical intermediate **24** in the reaction medium, we sought to establish that this species originates specifically from iminyl phosphine **29** upon SET oxidation to **29**˙^+^. To exclude alternative sources, a series of control experiments was performed in which iminyl phosphine **29** was replaced with phosphine‐derived side products isolated from the reaction media (**36**–**38**). Among these, **36** is known to undergo SET oxidation under photoredox conditions to generate the corresponding phosphoryl radical **24** [6]. Accordingly, we evaluated whether **36** could serve as a viable precursor of **24**˙ using 4DPAIPN or thioxanthone (TXO). However, no formation of N‐phosphoryl azetidine **34** was observed (Figure 3C). Similarly, replacement of iminyl phosphine **29** with either **37** or **38** failed to afford product **34** (see Supporting Information, section 4.7). To discern whether radical **20** or **24** is responsible for the strain release of compound **32**, sulfur was subsequently added to the reaction after irradiation under argon atmosphere, which was then stirred for 1 h at ambient temperature. If the phosphinyl radical **20** is responsible for the ABB opening, the formation of compound **33** via sulfur oxidation should be observed. The absence of **33** along with generation of 5% of **30** (formed from the oxidation of unreacted **29**) suggests that the phosphoryl radical **24** is the only species responsible for strain release of **32** (Figure 3D). This finding is further corroborated by density functional theory (DFT) calculations performed at the UωB97X‐D/Def2‐SVP level of theory in dichloromethane (SMD solvation model) [10, 11]. The addition of a phosphinyl radical (Ph_2_P·) **20** to azabicyclo[1.1.0]butane **32** exhibits a relatively high activation barrier (Δ*E* ^‡^ = 14.1 kcal·mol^−1^; Δ*G* ^‡^ = 13.6 kcal·mol^−1^). In contrast, the phosphoryl radical **24** displays markedly enhanced reactivity toward azabicyclobutane **32** (Figure 3E), with a substantially lower activation barrier (Δ*E* ^‡^ = 8.9 kcal·mol^−1^; Δ*G* ^‡^ = 10.4 kcal·mol^−1^) and a pronouncedly exergonic reaction profile (Δ*E* = –22.0 kcal·mol^−1^; Δ*G* = –21.8 kcal·mol^−1 ^vs. Δ*E* = –43.2 kcal·mol^−1^; Δ*G* = –42.1 kcal·mol^−1^). To shed light on the second part of the catalytic manifold, we performed a reaction using 1.0 eq. of D_2_O, that resulted in 40% deuterium incorporation at C3 of the azetidine core. This finding suggests that upon radical opening of the ABB the transiently generated tertiary radical is reduced into the corresponding carbanion that reacts with H_2_O, as a proton source (Figure 3F). No reaction was observed without water (see Figure 2b, Supporting Information section 4.7.5). With the key steps of the catalytic cycle established, we finally turned to the transformation of the phosphine radical cation **29˙^+^** into the phosphoryl radical **24**. The superior performance of diphenylphosphinic acid **38** in enhancing the reaction yield, relative to other acid additives, led us to consider its potential nucleophilic attack on **29˙^+^**, followed by a β‐scission pathway to generate the radical **24** (Figure 3G, also see Supporting Information section 4.11). This process would be analogous to acyl radical generation from carboxylate anions and phosphine radical cations [50]. To probe this possibility, the standard iminyl phosphine **29** was replaced with the phosphite derivative **42**. The machine learning model predicts a triplet energy of 48.9 kcal·mol^−1^ for **42** with a DFT‐calculated bond dissociation energy (BDE) of 57.6 kcal·mol^−1^, consistent with the trend observed for **29**. Under these conditions, the N‐phosphoryl azetidine **34** was not detected in the reaction mixture; instead, azetidine **45** was obtained in 50% ^1^H NMR yield.

### 2.3 Computational Studies

To support our experimental findings while shedding light on the mechanism and the nature of key catalytic intermediates, DFT calculations were performed at the UωB97X‐D/Def2‐SVP level of theory [51, 52] in implicit dichloromethane (SMD solvation model) [53] using *Gaussian16 Rev C.02* [54]. On occasion, we ran preliminary calculations using ORCA (v. 5.0.4) [55] (see Supporting Information section 5.0) at the UωB97X‐D3/Def2‐SVP level of theory, in the gas phase [52, 56]. Consistent with the experimental observations (Figure 3G), the pathway involving nucleophilic attack of **38** on **29˙^+^**, followed by direct β‐scission of the resulting iminophosphoranyl intermediate to generate radical **24**, is confirmed not to be accessible at room temperature, since the predicted free energy barrier (∆*G* ^‡^) is over 22.5 kcal·mol^−1^ (see Supporting Information section 5.0 and calculations online) [57, 58, 59]. In our search for alternative reliable transition states involving the key reactants H_2_O and/or **38**, we reasoned that a pre‐association complex between **29˙^+^** and **38** should still be the mechanistically relevant starting point for subsequent investigation (Figure 4). Formation of this complex (**29˙^+^–38**) is slightly exergonic, with ∆*G* estimated at –2.6 kcal·mol^−1^. This complexation leads to the formation of iminophosphoranyl intermediate **(Int‐1)** via proton transfer from **38** through **TS1**. This step proceeds with a calculated free‐energy barrier of Δ*G* ^‡^ = +8.8 kcal·mol^−^^1^ and overall endergonicity of Δ*G* = +8.7 kcal·mol^−^^1^. This alternative mechanistic route proceeding from **Int‐1** bears a lower overall energetic cost compared to the β‐scission pathway (∆∆*G* ^‡^ = at least –2.7 kcal·mol^−1^). Notably, **Int‐1** features the unpaired electron mainly confined to the P atom, pointing directly away from the P–N bond and with marginal delocalization to the adjacent O and C atoms. This electronic arrangement enables heterolytic cleavage of the P–N bond, resulting in the formation of **19** and the phosphoranyl radical cation (**Int‐2**) with a ∆G^‡^ of +11.3 kcal·mol^−1^ and again an overall ∆G of +5.5 kcal·mol^−1^ (+14.0 kcal·mol^−1^ with respect to the initial **29˙^+^–38** complex). Accessing the transition state **TS2** on the way from **Int‐1** to the **Int‐2**–**19** complex only requires a modest elongation of the P–N bond (from 1.78 to 1.96 Å). Although the detailed mechanism for nucleophilic attack on **Int‐2** was not fully explored, Merz–Kollman–Singh charge fitting [60] on the optimized uncomplexed **Int‐2** (Figure 4) indicates that the non‐radical phosphorus center carries a higher positive charge (+0.65*e*) than the radical phosphorus (+0.42*e*), identifying it as the more favorable site for nucleophilic attack by H_2_O. Consistently, free‐energy comparisons between the optimized **Int‐2–H_2_O** complex and the resulting **24–38** complex show that the process is strongly exergonic (Δ*G* = –16.1 kcal·mol^−^^1^). This charge distribution further supports the fact that phosphoryl radical **24** originates from the iminyl phosphine **29** frameworks rather than from **38** (Refer to Figure 3G). The resulting phosphoryl radical **24** then undergo radical strain release with azabicyclo[1.1.0]butane **32** via **TS3** to generate a tertiary‐alkyl radical intermediate **Int‐3**. This process is characterized by a ∆*G* ^‡^ of +10.4 kcal·mol^−1^ and a very high exergonic reaction profile (∆*G* = –42.1 kcal·mol^−1^). The resulting intermediate **Int‐3** then undergoes a facile single‐electron transfer (SET) reduction by the PC radical anion to generate the anionic intermediate **Int‐4**, which subsequently yield the final N‐phosphoryl azetidine **34**.

### 2.4 Generality of the Method

**FIGURE 5:**
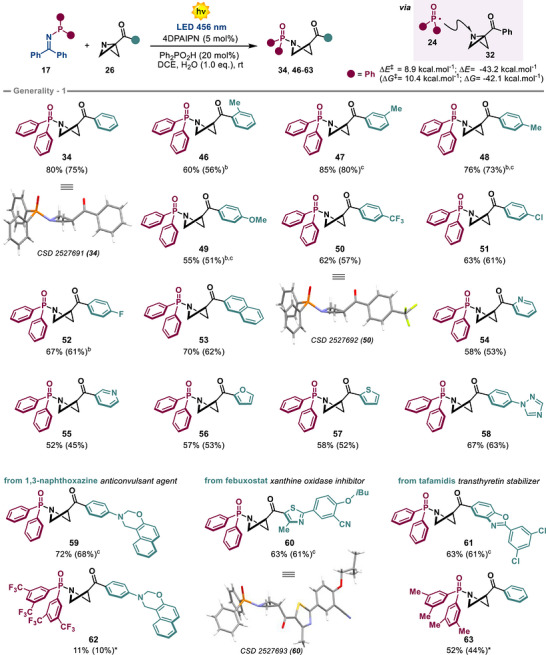
Generality of the developed method. Reactions were performed at 0.1 mmol scale. See Supporting Information, section 3.3. Isolated yields are given in brackets. ^[b]^Reaction time 48 h. ^[c]^Without acid additive. ^[^*^]^Iminyl phosphine generated in situ. CSD, Cambridge Structural Database (CSD 2527691, CSD 2527692, CSD 2527693).

We next assessed the generality of the investigated P–N bond forming reaction between **17** and ABBs **26** (Figure 5). ABBs featuring aryl substituents with EDGs were well tolerated, delivering azetidines **46**–**49** in yields of up to 85%. In contrast, *para*‐EWGs (CF_3_, Cl, and F) afforded the corresponding products **50**–**52** in slightly diminished yields, up to 67%. Replacement of the phenyl group in ABB **32** with a naphthyl moiety, as well as a range of other heteroaromatic substituents including pyridyl (*ortho* and *meta*), furan, thiophene, and triazole proceeded smoothly, furnishing N‐phosphoryl azetidines **53**–**58** in yields ranging from 52% to 70%. The method was next applied to more complex, biologically relevant scaffolds. Remarkably, azabicyclo[1.1.0]butanes derived from a 1,3‐naphthoxazine derivative, as well as from pharmaceutically relevant compounds such as febuxostat and tafamidis were efficiently converted into the respective azetidines **59**–**61** in yields of up to 72%. Introduction of trifluoromethyl groups at the 3,5‐positions of the phosphine aryl ring led to a drastic reduction in yield, affording azetidine **62** in only 11%. In contrast, (bis(3,5‐dimethyl) on the phosphine **63** are tolerated in the reaction, albeit with moderate efficiency (52% yield).

**FIGURE 6:**
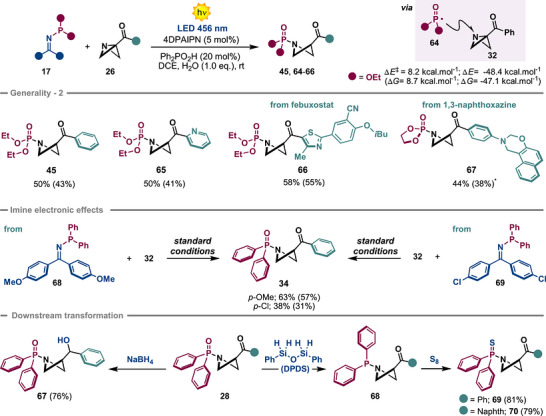
Utility of the developed method and product manipulation. Reactions were performed at 0.1 mmol scale (See Supporting Information, section 3.3 and 3.6). Isolated yields are given in brackets. ^[^*^]^Iminyl phosphine generated in situ.

We next explored the feasibility of replacing the diaryl phosphoryl radical with a dialkoxy phosphoryl radical (Figure 6). DFT calculations predicted a comparable activation barrier for radical addition to model ABB **32** towards **64** relative to **24** (See Figure 6 pink box, top right). Pleasingly, reaction of **42** with **32** and an *o‐*pyridyl‐substituted ABB afforded azetidines **45** and **65** in 50% yield. The versatility of the diethoxy phosphoryl radical **64** was further demonstrated through late‐stage functionalization of a febuxostat‐derived ABB, delivering azetidine **66** in 58% yield. Notably, we successfully introduced a cyclic phosphite moiety into the azetidine core, resulting in the naphthoxazine‐derived azetidine **67** with a yield of 44%. It is worth noting that, compounds **62, 63,** and **67** were synthesized through a three‐component reaction via in‐situ generation of the corresponding iminyl phosphines (See Supporting Information section 3.4). Next, we evaluated the electronic effect on the aromatic phenyl rings on imine for the photocatalytic process (Figure 6, imine electronic effects). The *p*‐OMe‐substituted iminyl phosphine **68** afforded the N‐phosphoryl azetidine **34** in 63% yield, whereas the *p‐*Cl‐substituted iminyl phosphine **69** provided a lower yield of 38%. We finally performed some synthetic manipulations that enabled further selective modification of the carbonyl and phosphine functionalities (Figure 6, downstream transformations). Azetidine **34** was reduced to the corresponding secondary alcohol **70** using NaBH_4_ in 76% yield. In addition, compounds **34** and **53** underwent selective reduction with air‐stable 1,3‐diphenyl‐disiloxane (DPDS) generating azetidine **71**. Subsequent oxidation of the P(III) azetidine afforded the N‐thiophosphoryl azetidines **72** and **73** in up to 81% yield.

## 3 Conclusion

In summary, we have shown that iminyl phosphines can be efficiently engaged under photocatalytic conditions to access rare P–N bonds within azetidine scaffolds. The reaction proceeds through an unconventional mechanistic pathway in which initial single‐electron transfer (SET) oxidation of the iminyl phosphine triggers a cascade of events, assisted by hydrogen‐bond donors, that culminates in the generation of a high‐energy phosphoryl radical. This key intermediate was directly detected by EPR spectroscopy and identified as the species responsible for azabicyclo[1.1.0]butane (ABB) ring opening. Subsequent P–N bond formation, reduction of the resulting tertiary radical by the PC radical anion, and protonation deliver the N‐phosphoryl azetidine products. A series of control experiments and DFT calculations supported the proposed mechanism. The potential and generality of the method was demonstrated across a broad range of structurally diverse and biologically relevant substrates, providing N‐phosphoryl azetidines in high yields (up to 85%). Moreover, these products can be readily converted into N‐thiophosphoryl azetidines, enabling downstream transformation. Overall, this work establishes iminyl phosphines as a new class of bench stable trivalent P‐centered radical precursors and offers a mechanistic platform for the photochemical installation of P‐atom into diverse molecular frameworks.

## Author Contributions

Chandu G. Krishnan conceived the project and devised the experiments with Luca Dell'Amico. Chandu G. Krishnan, and Gabriel Cormier carried out the reactions and isolated and characterized the products. Luca Dell'Amico, Chandu G. Krishnan, and Gabriel Cormier rationalized the experimental results. Marco Bortolus performed the spectroscopic investigations using EPR. Stefano A. Serapian performed the DFT studies. Luca Dell'Amico and Chandu G. Krishnan wrote the manuscript with contributions from all the authors. Luca Dell'Amico directed the work.

## Conflicts of Interest

The authors declare no conflicts of interest.

## Supporting information




**Supporting File 1**: anie72854‐sup‐0001‐SuppMat.pdf.

## Data Availability

The data that support the findings of this study are available on request from the corresponding author. The data are not publicly available due to privacy or ethical restrictions.
