# Supplementary material for: Phosphoryl Radicals from Trivalent Iminyl Phosphines: A Photocatalytic Approach to N‐Phosphoryl Azetidines
Source: Angew Chem Int Ed Engl. 2026 May 22;65(29):e9986925. doi: 10.1002/anie.9986925 (PMC13360835; doi:10.1002/anie.9986925)
Supplement: Supplementary file 1 — Supporting File 1: anie72854‐sup‐0001‐SuppMat.pdf. [file ANIE-65-e9986925-s001.pdf]

Supporting Information

Phosphoryl Radicals from Trivalent Iminyl Phosphines: A Photocatalytic Approach to *N*-Phosphoryl Azetidines

Chandu G. Krishnan,[1] Gabriel Cormier,[1] Stefano A. Serapian,[2] Marco Bortolus,[1] Luca Dell'Amico*[1]

[1]*Department of Chemical Sciences, University of Padova, via Marzolo 1, 35131, Padova, Italy*
[2]*Department of Chemistry, University of Pavia, via Taramelli 10, 27100, Pavia, Italy.*

luca.dellamico@unipd.it

Table of Contents

1. General information……………………………………………………………………………………3-4

1.1. Reagents, solvents and experimental conditions……………………………………………………3

1.2. Analytical techniques………………………………………………………………………………3

1.3. Computational methods……………………………………………………………………………3

1.4. Naming of compounds……………………………………………………………………………...4

1.5. Photochemical reaction setup and light source……………………………………………………4

2. Substrate synthesis……………………………………………………………………………………...5-12

2.1. Synthesis of novel class of iminyl phosphines……………………………………………………5-9

2.2. Synthesis of azabicyclo[1.1.0]butanes……………………………………………………………9-12

3. Photochemical reactions……………………………………………………………………………13-26

3.1. Preliminary trials……………………………………………………………………………..13-14

3.2. Optimization………………………………………………………………………………………14-17

3.3. General procedure for the photochemical reactions………………………………………………17-22

3.4. Substrate limitations………………………………………………………………………………..22

3.5. Three component reaction…………………………………………………………………………23-25

3.6. Large scale synthesis……………………………………………………………………………..25

3.7. Product Manipulation……………………………………………………………………………..25-26

4. Mechanistic investigations…………………………………………………………………………27-45

4.1. Electrochemical studies and evaluation of the SET process……………………………………..27

4.2. Stern- Volmer luminescence quenching studies…………………………………………....28-29

4.3. UV–visible absorption studies……………………………………………………………………...29

4.4. Direct excitation studies……………………………………………………………………………29-30

4.5. TEMPO trapping studies……………………………………………………………………………30

4.6. EPR spin trapping studies…………………………………………………………………………….31-32

4.7. Control experiments………………………………………………………………………………33-36

4.8. Oxidation by sulfur experiment……………………………………………………………………36

4.9. Activation energy barriers for radical addition……………………………………………………36-37

4.10. Deuterium incorporation studies…………………………………………………………………37

4.11. Experimental investigation on β-scission pathway…………………………………………...37-39

4.12. Determination of Molar extinction coefficient……………………………………………..39-41

4.13. Quantum yield measurement……………………………………………………………………42-44

4.14. Comparative study of reported phosphoryl radical precursor in the aza-BCB system……………..45

5. Computational studies………………………………………………………………………………………46

5.1. Triplet energy and Bond dissociation energies……………………………………………………….47

6. Crytal structures…………………………………………………………………………………………48-50

7. NMR data's………………………………………………………………………………………...51-109

8. References………………………………………………………………………………………...110-111

1. General information

1.1. Reagents, solvents and experimental conditions

All commercially available fine chemicals were obtained from Acros (Fisher), Aldrich (Merck), Alfa Aesar (Fisher), Fluorochem, TCI, or BLDpharm and were used without further purification unless otherwise noted. Anhydrous solvents were purchased from Acros in AcroSeal® bottles or from Aldrich (Merck) in Sure/Seal™ bottles and stored over 3 or 4 A^o molecular sieves prior to use. The anhydrous 1,2-dichloroethane (DCE, 99.8%) were commercially purchased from Sigma-Aldrich and is directly used under argon atmosphere unless specified. Photocatalytic reactions were conducted under an argon atmosphere in oven-dried Schlenk tubes. Reaction mixtures were degassed by three freeze pump thaw cycles (5 minutes each for 0.1 mmol scale, 20 minutes for 1.00 mmol scale) to remove dissolved oxygen before irradiation. Thioxanthone and $Ir[dF(CF_3)ppy]_2(dtbbpy)PF_6$ were purchased and used as received. Other organic photocatalysts were synthesized according to reported literature procedures.

1.2. Analytical techniques

NMR spectra were recorded on Bruker 400 Avance III HD equipped with a BBI-z grad probe head 5mm (^{1}H: 400 MHz, ^{13}C: 100.5 MHz, ^{19}F: 376 MHz, ^{31}P: 162 MHz), Bruker 500 Avance III equipped with a BBI-ATM-z grad probe head 5mm (^{1}H: 500 MHz, ^{13}C: 101 MHz, ^{19}F: 470 MHz, ^{31}P: 202 MHz) and Bruker 600 Avance III HD equipped with a BBI-z grad probe head 5mm (^{1}H: 600 MHz, ^{13}C: 150 MHz, ^{19}F: 565 MHz, ^{31}P: 243 MHz). The chemical shifts in $CDCl_3$ were reported in the scale relative to $CHCl_3$ (7.26 ppm) for ^{1}H NMR, to $CDCl_3$ (77.16 ppm) for ^{13}C NMR and CD_2Cl_2 in the scale relative to CH_2Cl_2 (5.32 ppm) for ^{1}H NMR, to CD_2Cl_2 (53.84 ppm) as internal references. Coupling constants are given in Hz. The following abbreviations are used to indicate the multiplicity: s, singlet; d, doublet; t, triplet; q, quartet; m, multiplet; br, broad signal; app, apparent. NMR yields were calculated by using 1,3,5-trimethoxybenzene as internal standard unless otherwise noted. In cases where the ^{1}H NMR yield was difficult to determine accurately, triphenyl phosphite was used as an internal standard, and the yield was calculated by ^{31}P NMR spectroscopy.

All the cyclic voltammograms were recorded with a scan rate of 0.1 V/s and were carried out in 1,2-dichloroethane (DCE, 0.1 M), tetrabutylammonium hexafluorophosphate ($TBAPF_6$) at room temperature (rt), on an BASi EC Epsilon potentiostat-galvanostat in a glass cell. A typical three-electrode cell was employed, which was composed of a glassy carbon (GC) working electrode (3.0 mm diameter), a platinum wire as counter electrode and a saturated aqueous calomel electrode (SCE) as reference electrode. Oxygen was removed by bubbling the solvent with high-purity nitrogen (N_2), introduced from a line into the cell by means of a glass pipe. The potential of ferrocenium/ferrocene (Fc+/Fc) couple was used as internal reference system to calibrate the potentiostat. All the results are subsequently converted in V vs SCE, in agreement with the value reported in literature [$E_{1/2}$(Fc+/Fc) = +0.38 V vs SCE]. Thin-layer chromatography (TLC) analysis was performed on pre-coated Merck TLC plates (silica gel 60 GF254, 0.25 mm). Organic solutions were concentrated under reduced pressure on a Heidolph rotary evaporator.

High-Resolution Mass Spectra (HRMS) were obtained using Waters GCT gas chromatograph coupled with a time-of-flight mass spectrometer (GC/MS-TOF) with electron ionization (EI). Routine MS for calculating the ratio of 3:4 was conducted in a Waters Acquity UPC^2 using CO_2/MeOH as mobile phase.

1.3. Computational methods

Input and output for the climbing-image nudged elastic band (CI-NEB) calculation[1] with ORCA (v. 5.0.4)[2] (details see section 5.0) are provided electronically in a zip folder. Full input and output of every other calculation mentioned in the present section are retrievable and downloadable from a dedicated (published) *ioChem-BD* repository.[4] Unless otherwise stated, all calculations were carried out using *Gaussian16 Rev C.02*[5] at the UωB97X-D/Def2-SVP level of theory,[6,7] using the SMD implicit solvation model[8] for dichloromethane. All energy values reported in the computational studies section are calculated at this level of theory. Input models for all structures were constructed with GaussView.[9]

1.4. Naming of compounds

Compound names are those generated by ChemDraw Professional 23.1.1 software (PerkinElmer), following the IUPAC nomenclature.

1.5. Photochemical reaction setup and light source

The Kessil lamp PR160L-456 (50W) was purchased from Kessil webpage: https://www.kessil.com/science/PR160L.php. The light emission spectra of Kessil lamps can be found on the same website.

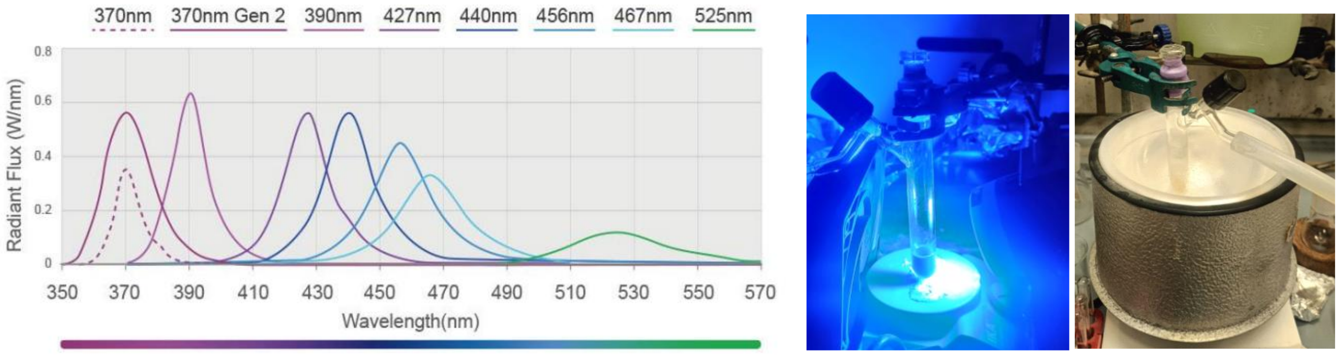

Figure S1. (Left) Emission spectra of Kessil PR160L lamps, (Middle) photoreactor setup, (Right) freeze pump thaw.

2. Substrate synthesis

2.1. Synthesis of novel class of iminyl phosphines

2.1.1. Synthesis of N-(diphenylphosphaneyl)-1,1-diphenylmethanimine (29)

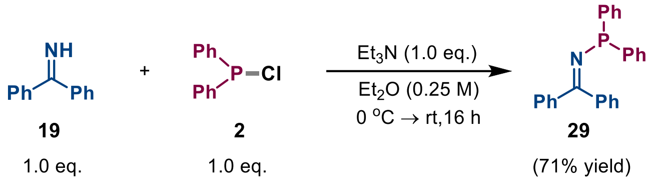

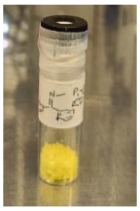

An oven dried round bottom flask equipped with a magnetic stir bar was charged with commercially available benzophenone imine **19** (1.4 mL, 10.0 mmol, 1.0 eq.) and triethylamine (1.4 mL, 10.0 mmol, 1.0 eq.) in anhydrous Et_2O (40.0 mL, 0.25 M). The solution was cooled to 0 °C using an ice-water bath, and chlorodiphenylphosphine **2** (1.8 mL, 10.0 mmol, 1.0 eq.) was added dropwise under an argon atmosphere. The reaction mixture turned into a yellow precipitate and was stirred at room temperature for 16 h. The resulting suspension was filtered by vacuum filtration using a Büchner funnel and washed with Et_2O (5.0 mL × 3 times), and the solvent was removed under reduced pressure. The residue was treated with small amounts of CH_3CN (4.0 mL) and sonicated to precipitate yellow solids, which were collected by vacuum filtration using a Büchner funnel. Then, the resulting yellow solid was further washed with CH_3CN (4.0 mL × 3 times), and dried to afford iminyl phosphine **29**.

Supplementary advice 1

It's recommended to use a bigger magnetic stirring bar as the formation of precipitate upon the addition of chlorodiphenylphosphine **2** creates difficulty with stirring.

Supplementary advice 2

Before attempting recrystallization using acetonitrile (CH_3CN), it is strongly recommended to thoroughly dry the crude material under high vacuum. Complete removal of residual solvents greatly facilitates successful recrystallization.

Supplementary advice 3

If crystallization does not occur upon sonication, the reaction mixture should be cooled using an ice-water bath for 2 h following the addition of acetonitrile (CH_3CN). Under these conditions, yellow crystalline iminyl phosphine **29** gradually precipitates and can be isolated by vacuum filtration using a Büchner funnel. However, employing this cooling-assisted crystallization method results in a decreased isolated yield (54%) compared to the standard procedure.

Stability and storage conditions

The compound is a bench-stable solid under ambient laboratory conditions and can be stored at room temperature for extended periods without detectable degradation.

Yellow solid; 2.6 g, 7.1 mmol, 71% yield; **^{1}H NMR (400 MHz, CD_2Cl_2) *δ*:** 7.51 – 7.44 (m, 4H), 7.43 – 7.35 (m, 6H), 7.34 – 7.29 (m, 4H), 7.27 – 7.22 (m, 6H) ppm; **^{13}C NMR (101 MHz, CD_2Cl_2) *δ*:** 175.54 (d, J = 10.9 Hz), 142.46 (d, J = 11.6 Hz), 140.38 (d, J = 7.3 Hz), 132.29, 132.08, 129.99, 128.87, 128.54 (d, J = 4.4 Hz), 128.47 (d, J = 4.7 Hz) ppm; **^{31}P NMR (162 MHz, CD_2Cl_2) *δ*:** 35.8 ppm. The obtained ^{1}H NMR data were consistent with those reported in the literature.[10]

2.1.2. Synthesis of N-(diphenylphosphaneyl)-1,1-bis(4-methoxyphenyl)methanimine (68)

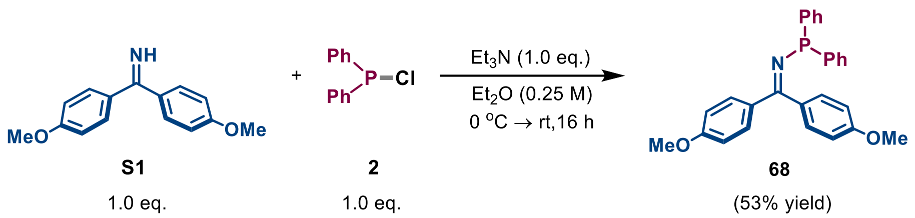

An oven dried round bottom flask equipped with a magnetic stir bar was charged with benzophenone imine **S1** (1.09 mg, 5.0 mmol, 1.0 eq.) and triethylamine (0.70 mL, 5.0 mmol, 1.0 eq.) in anhydrous Et_2O (20.0 mL, 0.25 M). The solution was cooled to 0 °C using an ice-water bath, and chlorodiphenylphosphine **2** (0.90 mL, 5.0 mmol, 1.0 eq.) was added dropwise under an argon atmosphere. The reaction mixture turned into a yellow precipitate and was stirred at room temperature for 16 h. The resulting suspension was filtered under vacuum through a Büchner funnel and washed with Et_2O (5.0 mL × 3). The solvent was removed under reduced pressure to afford a residual orange oil. This oil was dissolved in a minimal amount of Et_2O (2.0 mL with sonication), and impurities were precipitated by addition of hexane. The precipitate was removed by vacuum filtration, and the filtrate was concentrated under reduced pressure to afford iminyl phosphine **68**.

Supplementary advice 4

It's recommended to use a bigger magnetic stirring bar as the formation of precipitate upon the addition of chlorodiphenylphosphine **2** creates difficulty with stirring.

Stability and storage conditions

The compound is obtained as a stable orange oil, exhibiting no detectable degradation under ambient laboratory conditions and remained stable upon storage in a refrigerator for extended periods.

Orange oil; 1.13 g, 2.66 mmol, 53% yield; **^{1}H NMR (400 MHz, $CDCl_3$) *δ*:** δ 7.60 – 7.50 (m, 4H), 7.40 (d, J = 6.8 Hz, 4H), 7.31 – 7.23 (m, 6H), 6.84 (d, J = 8.5 Hz, 4H), 3.86 (s, 6H) ppm; **^{13}C NMR (101 MHz, $CDCl_3$) *δ*:** δ 174.9 (d, J = 11.6 Hz), 160.8, 142.6 (d, J = 11.6 Hz), 133.2 (d, J = 8.0 Hz), 132.3 (d, J = 23.3 Hz), 132.1, 131.8, 130.1 (d, J = 2.5 Hz), 128.5, 128.2 (d, J = 7.3 Hz), 55.5 ppm; **^{31}P NMR (162 MHz, $CDCl_3$) *δ*:** 35.8 ppm.

2.1.3. Synthesis of 1,1-bis(4-chlorophenyl)-N-(diphenylphosphaneyl)methanimine (69)

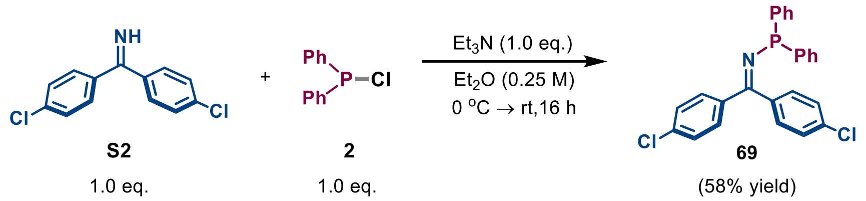

An oven dried round bottom flask equipped with a magnetic stir bar was charged with benzophenone imine **S2** (1.25 mg, 5.0 mmol, 1.0 eq.) and triethylamine (0.70 mL, 5.0 mmol, 1.0 eq.) in anhydrous Et_2O (20.0 mL, 0.25 M). The solution was cooled to 0 °C using an ice-water bath, and chlorodiphenylphosphine **2** (0.90 mL, 5.0 mmol, 1.0 eq.) was added dropwise under an argon atmosphere. The reaction mixture turned into a yellow precipitate and was stirred at room temperature for 16 h. The resulting suspension was filtered under vacuum through a Büchner funnel and washed with Et_2O (5.0 mL × 3). The solvent was removed under reduced pressure to afford a residual orange oil. This oil was dissolved in a minimal amount of Et_2O (2.0 mL with sonication), and impurities were precipitated by addition of hexane. The precipitate was removed by vacuum filtration, and the filtrate was concentrated under reduced pressure to afford iminyl phosphine **69**.

Supplementary advice 5

It’s recommended to use a bigger magnetic stirring bar as the formation of precipitate upon the addition of chlorodiphenylphosphine **2** creates difficulty with stirring.

Stability and storage conditions

The compound is obtained as a stable orange oil, exhibiting no detectable degradation under ambient laboratory conditions and remained stable upon storage in a refrigerator for extended periods.

Orange oil; 1.26 g, 2.90 mmol, 58% yield; **^{1}H NMR (400 MHz, $CDCl_3$) *δ*:** 7.52 – 7.33 (m, 6H), 7.27 (q, *J* = 8.0 Hz, 12H) ppm; **^{13}C NMR (101 MHz, $CDCl_3$) *δ*:** 171.8 (d, *J* = 9.8 Hz), 141.3 (d, *J* = 10.9 Hz), 138.1 (d, *J* = 6.9 Hz), 136.0, 132.2 (d, *J* = 21.1 Hz), 131.6 (d, *J* = 21.4 Hz), 129.4 (d, *J* = 2.5 Hz), 128.8, 128.6, 128.3 (d, *J* = 7.3 Hz) ppm; **^{31}P NMR (162 MHz, $CDCl_3$) *δ*:** 37.7 ppm.

2.1.4. Synthesis of N-(diphenylmethylene)-P,P-diphenylphosphinothioic amide (30)

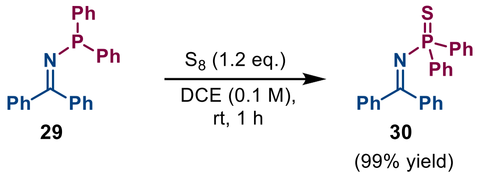

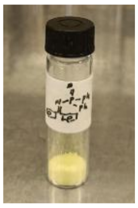

A flame-dried round bottom flask (50.0 mL) was placed under an argon atmosphere and charged with iminyl phosphine **29** (365.0 mg, 1.0 mmol, 1.0 eq.) in anhydrous DCE (10.0 mL, 0.1 M). Elemental sulfur (38.4 mg, 1.2 mmol, 1.2 eq.) was added, and the reaction mixture was stirred at room temperature for 1 hour. The solvent was then removed under reduced pressure, and the residue was purified by silica gel column chromatography to afford the corresponding product **30**.

Stability and storage conditions

The compound is a bench-stable solid under ambient laboratory conditions and can be stored at room temperature for extended periods without detectable degradation.

Pale yellow amorphous solid; 392.0 mg, 0.99 mmol, 99% yield; Eluent: Hexane/EtOAc, 95/5 to 80/20;
^{1}H NMR (400 MHz, $CDCl_3$) *δ*: 7.92 (dd, *J* = 13.3, 8.0 Hz, 4H), 7.51 – 7.27 (m, 16H) ppm; **^{13}C NMR (101 MHz, $CDCl_3$) *δ*:** 181.93 (d, *J* = 9.1 Hz), 138.41 (d, *J* = 17.8 Hz), 136.52 (d, *J* = 103.9 Hz), 131.45 (d, *J* = 10.5 Hz), 131.16, 130.95 (d, *J* = 2.9 Hz), 129.49, 128.22 (d, *J* = 13.1 Hz), 127.92 ppm; **^{31}P NMR (162 MHz, $CDCl_3$) *δ*:** 48.0 ppm.

2.1.5. Synthesis of N-(diphenylmethylene)-P,P-diphenylphosphinic amide (31).

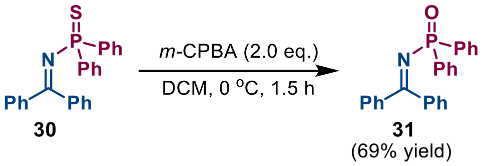

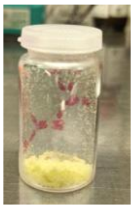

Iminyl phosphine **30** (200.0 mg, 0.5 mmol, 1.0 eq.) was placed in a round-bottom flask (50.0 mL) and dissolved with DCM (10.2 mL, 0.05M). After the solution was cooled to 0 ºC using an ice-water bath, *m*-CPBA (contains ca. 30% H_2O, 123.3 mg, 0.5 mmol, 1.0 eq.) was slowly added and the mixture was stirred for 1 hour at 0 ºC. Additional *m*-CPBA (contains ca. 30% H_2O, 123.3 mg, 0.5 mmol, 1.0 eq.) was slowly added, and then, the mixture was stirred for 30 minutes at 0 ºC. The reaction mixture was then quenched with sat. aq. $Na_2S_2O_3$ (4.0 mL), sat. aq. $NaHCO_3$ (4.0 mL) and the mixture was further stirred for 5 minutes and extracted with DCM (3.0 mL × 3 times). The organic layer

was dried with Na_2SO_4, filtered, and concentrated under reduced pressure. The product was purified by silica-gel column chromatography to afford the product **31**.

Stability and storage conditions

The compound is a bench-stable solid under ambient laboratory conditions and can be stored at room temperature for extended periods without detectable degradation.

Light yellow solid; (132.0 mg, 0.035 mmol, 69% yield); Eluent: Hexane/EtOAc = 80/20 to 50/50; **^{1}H NMR (400 MHz, CDCl$_3$) δ:** 7.93 (dd, J = 11.9, 6.5 Hz, 4H), 7.60 – 7.35 (m, 16H) ppm; **^{13}C NMR (101 MHz, CDCl$_3$) δ:** 182.08 (d, J = 8.0 Hz), 138.87 (d, J = 16.7 Hz), 135.06 (d, J = 130.8 Hz), 131.80 (d, J = 9.1 Hz), 131.46, 131.38 (d, J = 2.9 Hz), 129.72, 128.45 (d, J = 12.4 Hz), 128.05 ppm; **^{31}P NMR (162 MHz, CDCl$_3$) δ:** 16.5 ppm.

2.1.6. Synthesis of diethyl (diphenylmethylene)phosphoramidite (42).

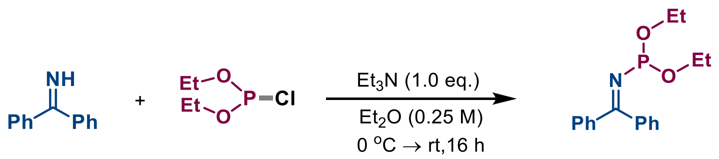

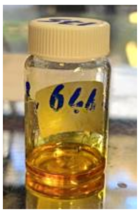

19 1.0 eq. — **S3** 1.0 eq. — **42** (49% yield)

An oven dried round bottom flask equipped with a magnetic stir bar was charged with benzophenone imine **19** (1.7 mL, 10.0 mmol, 1.0 eq.) and triethylamine (1.4 mL, 10.0 mmol, 1.0 eq.) in anhydrous Et_2O (40.0 mL, 0.25 M). The solution was cooled to 0 °C using an ice-water bath, and diethyl chlorophosphate **S3** (1.5 mL, 10.0 mmol, 1.0 eq.) was added dropwise under an argon atmosphere. The reaction mixture turned yellow and was stirred at room temperature for 16 h. The resulting suspension was filtered, and the solvent was removed under reduced pressure. The crude residue was purified by Kugelrohr vacuum distillation. The temperature gradually increased by 5 ºC from 160 °C to 200 °C under reduced pressure to remove low-boiling impurities and residual byproducts. The pure iminyl phosphine **42** was subsequently collected as an orange fraction distilling between 200 °C and 220 °C (under vacuum). The distilled product was immediately sealed and stored under an inert atmosphere to prevent oxidation or hydrolysis (1.48 g, 4.93 mmol, 49% yield).

Supplementary advice 6

It's recommended to use a bigger magnetic stirring bar as the formation of precipitate upon the addition of diethyl chlorophosphite **S3** creates difficulty with stirring.

Stability and storage conditions

The compound should be stored in a refrigerator, as minor decomposition was observed in NMR samples after 48 hours at room temperature.

Orange viscous oil; **^{1}H NMR (400 MHz, CDCl$_3$) δ:** 7.58 – 7.50 (m, 4H), 7.48 – 7.35 (m, 6H), 4.11 – 3.89 (m, 4H), 1.26 (t, J = 7.1 Hz, 6H) ppm; **^{13}C NMR (101 MHz, CDCl$_3$) δ**: 177.26 (d, J = 20.8 Hz), 139.49 (d, J = 6.9 Hz), 130.4, 129.1 (d, J = 2.9 Hz), 128.1, 59.3 (d, J = 9.4 Hz), 17.2 (d, J = 4.7 Hz) ppm; **^{31}P NMR (162 MHz, CDCl$_3$) δ**: 135.2 ppm. **HRMS (ESI):** m/z calcd. for $C_{17}H_{21}NO_2P$ $[M+H]^+$: 302.1310, found: 302.1298.

2.1.7. Synthesis of N-(diphenylphosphino)benzalimine (S7).

Step-1: Synthesis of *(E)*-1-phenyl-N-(trimethylsilyl)methanimine (S6).

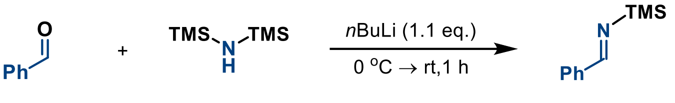

S4 1.0 eq. S5 1.1 eq. S6 (29% yield)

A flame-dried single-necked round-bottom flask (50.0 mL) equipped with a magnetic stir bar was charged with bis(trimethylsilyl)amine **S5** (6.9 mL, 33.0 mmol, 1.1 eq.) under an argon atmosphere. *n*-butyllithium (10.6 mL, 1.6 M in hexane, 33.0 mmol, 1.1 eq.) was added dropwise at 0 °C, and the mixture was allowed to warm to room temperature and stirred for 15 minutes. The reaction mixture was then cooled to 0 °C, followed by the addition of benzaldehyde **S4** (3.0 mL, 30.0 mmol, 1.0 eq). After stirring for an additional 45 minutes at room temperature, the solvent was removed under reduced pressure. The crude residue was purified by Kugelrohr vacuum distillation. Low-boiling impurities and residual byproducts were removed by distilling at temperatures ranging from 80 °C to 100 °C under reduced pressure. The pure N-(trimethylsilyl)methanimine **S6** was subsequently collected as a pale-yellow liquid fraction (29%, 1.5 g, 8.7 mmol) distilling between 110 °C and 120 °C (under vacuum). The distilled product was immediately used for the next step. The obtained ^{1}H NMR data were consistent with those reported in the literature.[11]

Step-2: Synthesis of N-(diphenylphosphino)benzalimine (S7).

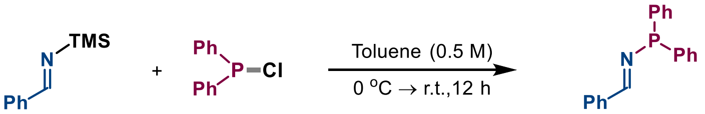

S6 1.0 eq. 2 1.0 eq. S7 (31% yield)

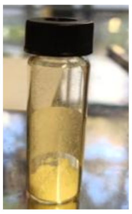

In a 100.0 ml round bottom flask was placed N-trimethylsilylbenzalimine **S6** (1.5 g, 8.5 mmol, 1.0 eq.) under argon atmosphere and added toluene (18.0 mL, 0.5 M). The reaction flask was cooled to 0° C and chlorodiphenylphosphine **2** (1.5 mL, 8.5 mmol, 1.0 eq.) was added dropwise. The reaction mixture was allowed to stir overnight. Removal of the solvent in vacuo resulted in a yellow sticky solid. Careful crystallization from acetonitrile will afford the desired N-(diphenylphosphino)benzalimine **S7** (31%, 766.0 mg, 2.7 mmol). ^{1}H NMR data is in accordance with the literature.[11]

Stability and storage conditions

The compound is a bench-stable solid under ambient laboratory conditions and can be stored at room temperature for extended periods without detectable degradation.

Pale-yellow solid; **^{1}H NMR (400 MHz, $CDCl_3$) *δ*:** 8.23 (d, *J* = 21.3 Hz, 1H), 7.78 (dd, *J* = 7.3, 2.8 Hz, 2H), 7.56 – 7.50 (m, 4H), 7.45 – 7.36 (m, 9H) ppm; **^{13}C NMR (101 MHz, $CDCl_3$) *δ*:** 165.0 (d, *J* = 3.7 Hz), 139.1 (d, *J* = 14.6 Hz), 137.6 (d, *J* = 12.5 Hz), 132.9 (d, *J* = 20.4 Hz), 131.4, 129.7 (d, *J* = 128.2 Hz), 129.5, 128.7 (d, *J* = 3.6 Hz), 128.6 (d, *J* = 8.5 Hz) ppm; **^{31}P NMR (162 MHz, $CDCl_3$) *δ*:** 49.2 ppm. **HRMS (ESI):** m/z calcd. for $C_{19}H_{17}NP$ $[M+H]^+$: 290.1099, found: 290.1078.

2.2. Synthesis of azabicyclo[1.1.0]butanes

2.2.1. Synthesis of 2,3-dibromopropan-1-amine hydrobromide

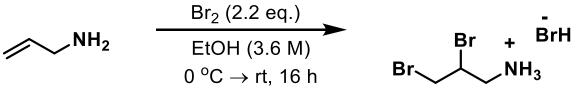

S8 1.0 eq. S9 (70% yield)

The following general procedure has been adapted from the literature.[12] A solution of Br_2 (5.0 mL, 99.0 mmol, 2.2 eq.) was very slowly added dropwise under vigorous stirring to ethanol (15.0 mL) in a 100.0 mL round bottom flask at 0 °C (Caution: exothermic, fuming). After the addition was complete, allylamine **S8** (3.4 mL, 45.0 mmol, 1.0 eq.) was added very slowly dropwise under vigorous stirring at 0 °C. The mixture was allowed to warm to room temperature and stirred for 16 hours. The precipitate was collected by vacuum filtration using a Büchner funnel filtration and washed with small portions of ice-cold Et_2O. The crude material was recrystallized from MeOH to give the hydrobromide salt **S9** as colorless prisms (70% yield, 9.4 g after two crop collections). The spectroscopic data was identical to that reported in the literature.[12]

Supplementary advice 7

It's recommended to use a bigger magnetic stirring bar as the formation of thick precipitate creates difficulty with stirring.

Supplementary advice 8

The addition of bromine to ethanol is strongly exothermic and fuming; adequate ventilation and an efficient ice bath are essential. The addition rate should be carefully controlled to maintain the internal temperature below 5 °C.

Stability and storage conditions

The product is bench-stable solid and can be stored under ambient conditions.

2.2.2. Preparation of Weinreb amides (S10–S13).

The following procedure has been adapted from the literature.[13] An oven-dried flask was charged with the corresponding carboxylic acid (10.0 mmol, 1.0 eq.), DMAP (5.0 mol%), EDC.HCl (15.0 mmol, 1.5 eq.), and N,O-dimethylhydroxylamine hydrochloride (12.0 mmol, 1.2 eq.). Under argon atmosphere, dry DCM (0.3 M) was added into the flask. The reaction mixture was cooled to 0 °C and stirred for 10 minutes. Triethylamine (20.0 mmol, 2.0 eq.) was then added dropwise into the reaction vessel. Once the addition finished, the ice bath was removed, and the mixture was stirred for 16 hours at room temperature. The reaction was quenched with 10% solution of citric acid. After separation of the organic layer, the aqueous phase was neutralized with saturated aqueous solution of $NaHCO_3$ and extracted with DCM. The organic layers were combined and washed with brine. The organic phase was then dried over anhydrous sodium sulfate, filtered, and concentrated under reduced pressure. The crude residue was pure enough to go to the next step without further purification. NMR data of the newly synthesized Weinreb amides **S10–S13** are reported below.

N-methoxy-N-methyl-4-(1H-1,2,4-triazol-1-yl)benzamide (S10)

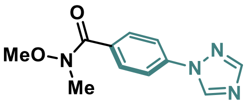

S10

White solid; 2.26 g, 9.72 mmol, 97% yield; **^{1}H NMR (400 MHz, $CDCl_3$) *δ*:** 8.62 (s, 1H), 8.12 (s, 1H), 7.88 (d, *J* = 8.7 Hz, 2H), 7.74 (d, *J* = 8.7 Hz, 2H), 3.55 (s, 3H), 3.39 (s, 3H) ppm; **^{13}C NMR (101 MHz, $CDCl_3$) *δ*:** 168.4, 153.0, 141.1, 138.5, 133.7, 130.3, 119.3, 61.3, 33.6 ppm; **HRMS (ESI):** m/z calcd. for $C_{11}H_{13}N_4O$ $[M+H]^+$: 233.1039, found: 233.1119.

N-methoxy-N-methyl-4-(1H-naphtho[1,2-e][1,3]oxazin-2(3H)-yl)benzamide (S11)

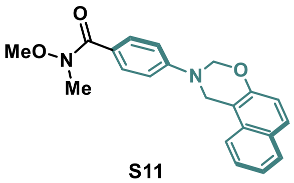

S11

Brown oil; 3.204 g, 9.20 mmol, 92% yield; **^{1}H NMR (400 MHz, $CDCl_3$) *δ*:** 7.81 – 7.63 (m, 5H), 7.53 (t, *J* = 7.4 Hz, 1H), 7.39 (t, *J* = 7.3 Hz, 1H), 7.13 (d, *J* = 8.3 Hz, 2H), 7.06 (d, *J* = 8.9 Hz, 1H), 5.46 (s, 2H), 5.00 (s, 2H), 3.54 (s, 3H), 3.33 (s, 3H) ppm; **^{13}C NMR (101 MHz, $CDCl_3$) *δ*:** 169.4, 152.3, 150.5, 131.2, 130.4, 129.1, 128.8, 128.6, 127.0, 125.9, 123.9, 120.9, 118.8, 116.4, 112.5, 78.3, 61.0, 47.8, 34.0 ppm; **HRMS (ESI):** m/z calcd. for $C_{21}H_{21}N_2O_3$ $[M+H]^+$: 349.1552, found: 349.1547.

2-(3-cyano-4-isobutoxyphenyl)-N-methoxy-N,4-dimethylthiazole-5-carboxamide (S12)

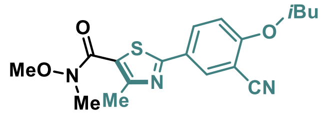

S12

White solid; 2.55 g, 7.10 mmol, 71% yield; **^{1}H NMR (400 MHz, CDCl$_3$) δ:** 8.19 – 8.10 (m, 3H), 7.01 (d, J = 8.9 Hz, 2H), 3.90 (d, J = 6.5 Hz, 2H), 3.74 (s, 3H), 3.37 (s, 3H), 2.79 (s, 3H), 2.21 (app. spt, J = 6.7 Hz, 1H), 1.09 (d, J = 6.6 Hz, 3H) ppm; **^{13}C NMR (101 MHz, CDCl$_3$) δ:** 167.1, 162.9, 162.4, 132.6, 132.2, 126.3, 118.7, 115.7, 112.7, 103.0, 75.8, 61.9, 33.0, 28.3, 19.2, 18.7 ppm; **HRMS (ESI):** m/z calcd. for $C_{18}H_{22}N_3O_3S$ [M+H]$^+$: 360.1382, found: 360.1527.

2-(3,5-dichlorophenyl)-N-methoxy-N-methylbenzo[d]oxazole-6-carboxamide (S13)

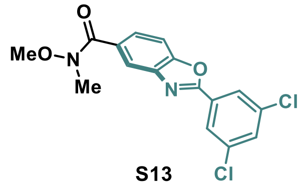

S13

Pink solid; 3.27 g, 9.29 mmol, 92% yield; **^{1}H NMR (400 MHz, CDCl$_3$) δ:** 8.18 – 8.14 (m, 2H), 8.00 (s, 1H), 7.79 (s, 2H), 7.54 (s, 1H), 3.57 (s, 3H), 3.42 (s, 3H) ppm; **^{13}C NMR (101 MHz, CDCl$_3$) δ:** 168.9, 162.3, 150.3, 143.7, 136.1, 131.8, 131.8, 129.7, 126.2, 125.9, 120.0, 111.6, 61.3, 33.9 ppm; **HRMS (ESI):** m/z calcd. for $C_{16}H_{13}Cl_2N_2O_3$ [M+H]$^+$: 351.0303, found: 351.0446.

2.2.3. Preparation of azabicyclo[1.1.0]butanes (S14–S17).

The following procedure has been adapted from the literature.[14] Phenyl lithium (in Bu_2O, 16.8 mmol, 3.6 eq.) was added dropwise (at a rate of 0.5 mL/min, syringe pump) to a suspension of salt **S9** (1.67 g, 5.61 mmol, 1.2 eq.) in anhydrous tetrahydrofuran (20.0 mL) at −78 °C (dry ice/acetone). After complete addition, the resulting grey to black solution was stirred for further 2 hours at −78 °C. TMEDA (freshly distilled from CaH_2, 0.9 mL, 5.9 mmol, 1.2 eq.), followed by *sec*-butyl lithium (in hexane, 5.9 mmol, 1.2 eq.) were added dropwise (at a rate of 0.5 mL/min, syringe pump), and the resulting solution was stirred for another 1 hour at −78 °C. Then, the corresponding Weinreb amide (4.68 mmol, 1.0 eq.) was added dropwise (at a rate of 0.5 mL/min, syringe pump) and the solution was stirred for 5 minutes before removing the cooling bath. Afterwards, the mixture was allowed to reach room temperature and stirred for 1 hour. Water (30.0 mL) was then added, phases were separated, and the aqueous one was extracted with ethyl acetate. The combined organic phases were dried over anhydrous sodium sulfate, filtered, and concentrated under reduced pressure. The crude residue was purified by silica gel column chromatography. NMR data of the newly synthesized azabicyclo[1.1.0]butanes **S14–S17** are reported below.

Supplementary advice 9

Do not remove Bu_2O for purification, liquid loading works perfectly

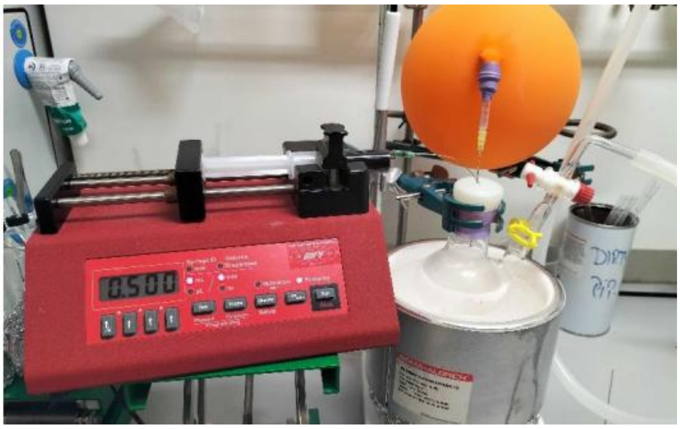

Figure S2. Reaction set-up for the synthesis of azabicyclo[1.1.0]butanes. Phenyl lithium addition is shown.

(4-(1H-1,2,4-triazol-1-yl)phenyl)(1-azabicyclo[1.1.0]butan-3-yl)methanoneone (S14)

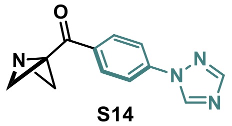

S14

White solid; 58 mg, 0.26 mmol, 4% yield; Eluent: Hexane/EtOAc = 80/20 to 60/40 **^{1}H NMR (400 MHz, CDCl$_3$) *δ*:** 8.67 (s, 1H), 8.13 – 8.03 (m, 3H), 7.84 – 7.76 (m, 2H), 3.04 (s, 2H), 1.76 (s, 2H) ppm; **^{13}C NMR (101 MHz, CDCl$_3$) *δ*:** 195.8, 153.1, 141.2, 140.5, 135.8, 130.7, 119.5, 56.8, 28.7 ppm; **HRMS (ESI):** m/z calcd. for $C_{12}H_{11}N_4O$ [M+H]$^+$: 227.0933, found: 227.1029.

(4-(1H-naphtho[1,2-e][1,3]oxazin-2(3H)-yl)phenyl)(1-azabicyclo[1.1.0]butan-3-yl)methanone (S15)

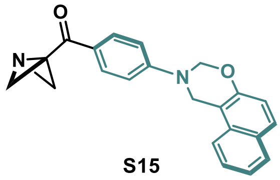

S15

Brown solid; 1.43 g, 4.18 mmol, 70% yield; Eluent: Hexane/EtOAc = 85/15 to 70/30 **^{1}H NMR (400 MHz, CDCl$_3$) *δ*:** 7.98 (d, *J* = 9.0 Hz, 2H), 7.80 (d, *J* = 8.1 Hz, 1H), 7.74 – 7.66 (m, 2H), 7.55 (t, *J* = 7.6 Hz, 1H), 7.41 (t, *J* = 7.5 Hz, 1H), 7.16 (d, *J* = 8.8 Hz, 2H), 7.07 (d, *J* = 8.9 Hz, 1H), 5.50 (s, 2H), 5.05 (s, 2H), 3.02 (s, 2H), 1.69 (s, 2H) ppm; **^{13}C NMR (101 MHz, CDCl$_3$) *δ*:** 194.5, 152.7, 152.3, 131.3, 131.2, 129.3, 129.0, 129.0, 128.9, 127.2, 124.2, 120.9, 118.8, 115.8, 112.3, 56.7, 47.6, 28.6 ppm; **HRMS (ESI)**: m/z calcd. for $C_{22}H_{19}N_2O_2$ [M+H]$^+$: 343.1447, found: 343.1744.

5-(5-(1-azabicyclo[1.1.0]butane-3-carbonyl)-4-methylthiazol-2-yl)-2-isobutoxybenzonitrile (S16)

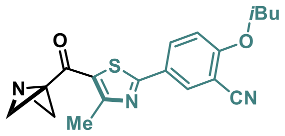

S16

White solid; 342 mg, 0.70 mmol, 12% yield; Eluent: Hexane/EtOAc = 85/15 to 70/30 **^{1}H NMR (400 MHz, CDCl$_3$) *δ*:** 8.23 – 8.20 (m, 1H), 8.14 (dd, *J* = 8.8, 1.9 Hz, 1H), 7.03 (d, *J* = 8.9 Hz, 1H), 3.91 (d, *J* = 6.5 Hz, 2H), 3.12 (s, 2H), 2.80 (s, 3H), 2.21 (app. spt, *J* = 6.8 Hz, 1H), 1.74 (s, 2H), 1.10 (d, *J* = 6.7 Hz, 3H) ppm; **^{13}C NMR (101 MHz, CDCl$_3$) *δ*:** 188.5, 168.1, 162.9, 162.1, 132.9, 132.5, 128.2, 125.8, 115.4, 112.8, 103.3, 75.9, 57.0, 31.1, 28.3, 19.2, 18.8 ppm; **HRMS (ESI)**: m/z calcd. for $C_{19}H_{20}N_3O_2S$ [M+H]$^+$: 354.1276, found: 354.1420.

(1-azabicyclo[1.1.0]butan-3-yl)(2-(3,5-dichlorophenyl)benzo[d]oxazol-5-yl)methanone (S17)

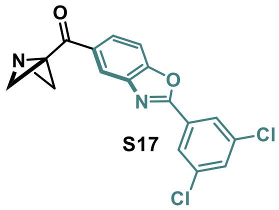

S17

White solid; 240 mg, 0.70 mmol, 12% yield; Eluent: Hexane/EtOAc = 85/15 to 70/30 **^{1}H NMR (400 MHz, CDCl$_3$) *δ*:** 8.26 – 8.24 (m, 1H), 8.18 (d, *J* = 1.9 Hz, 2H), 8.09 (dd, *J* = 8.3, 1.4 Hz, 1H), 7.86 (d, *J* = 8.3 Hz, 1H), 7.57 (t, *J* = 1.9 Hz, 1H), 3.12 (s, 2H), 1.82 (s, 2H) ppm; **^{13}C NMR (101 MHz, CDCl$_3$) *δ*:** 195.9, 163.5, 150.8, 146.2, 136.2, 134.5, 132.3, 129.3, 126.5, 126.4, 120.5, 111.8, 57.1, 28.9 ppm; **HRMS (ESI)**: m/z calcd. for $C_{17}H_{11}Cl_2N_2O_2$ [M+H]$^+$: 345.0198, found: 345.0174.

Stability and storage conditions

We did not observe any decomposition of azabicyclo[1.1.0]butanes **S14–S17** at ambient temperature. However, after use, the samples were stored in a refrigerator.

3. Photochemical reactions

3.1. Preliminary trials

3.1.1. Trials employing iminyl phosphine 29 as substrate.

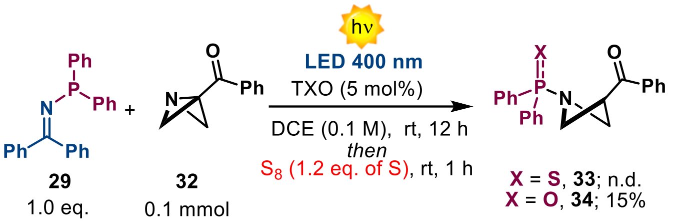

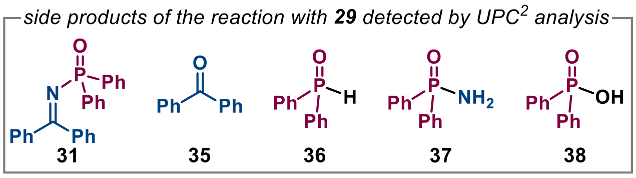

Procedure: In an oven-dried 10.0 mL Schlenk tube containing an oven dried Teflon coated stirring bar was added thioxanthone (1.1 mg, 5.0 mol%), iminyl phosphine **29** (36.5 mg, 0.1 mmol, 1.0 eq.), azabicyclo[1.1.0]butane **32** (15.9 mg, 0.1 mmol, 1.0 eq.) and were dissolved using the anhydrous DCE (0.1 M, 1.0 mL, commercially available, 99.8% from Sigma-Aldrich). The Schlenk tubes were then closed with the proper greased glass stoppers. The resulting solution was subjected to three cycles of free-pump-thaw to remove the oxygen and in the last cycle, the vessel was filled with argon. The reaction was irradiated under 400 nm blue LEDs for 12 hours with fan cooling. Afterwards, LED was removed and sulfur (3.8 mg, 0.12 mmol, 1.2 eq.) was added into the reaction mixture under argon atmosphere and stirred for another 1 hour at room temperature. Then, the solvent was evaporated under reduced pressure to afford the crude mixture. The crude mixture was further purified by silica-gel column chromatography (eluent: Hexane/Ethyl acetate, 90/10 to 0/100) to afford the corresponding N-phosphoryl azetidine **34** in 15% yield. ^{1}H NMR yield of the product was determined using 1,3,5-trimethoxybenzene as internal standard.

The same reactions were repeated by replacing **29** with **30** and **31** without sulfur treatment. However, both remain unreacted. We also performed the reaction with aldimine derived iminyl phosphine **S6**, unfortunately only traces of N-phosphoryl azetidine **34** was detected (< 5% based on ^{1}H NMR yield using 1,3,5-trimethoxybenzene as internal standard).

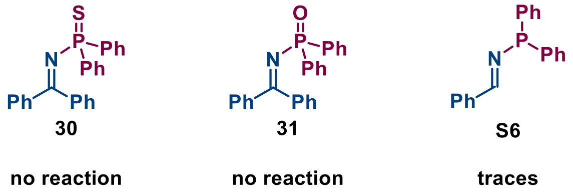

3.1.2. Trials employing iminyl phosphine 30 as substrate.

Procedure: In an oven-dried 10.0 mL Schlenk tube containing an oven dried Teflon coated stirring bar was added photocatalyst (5.0 mol%), iminyl phosphine **30** (39.7 mg, 0.1 mmol, 1.0 eq.), azabicyclo[1.1.0]butane **32** (15.9, 0.1 mmol, 1.0 eq.) and were dissolved using the anhydrous DCE (0.1 M, 1.0 mL, commercially available, 99.8% from Sigma-Aldrich).The Schlenk tubes were then closed with the proper greased glass stoppers. The resulting solution was subjected to three cycles of free-pump-thaw to remove the oxygen and in the last cycle, the vessel was filled with argon. The reaction was irradiated under blue LEDs for 20 hours with fan cooling. Afterwards, LED was removed and the solvent was evaporated under reduced pressure to afford the crude mixture and was analyzed by ^{1}H and ^{31}P NMR spectroscopy using 1,3,5-trimethoxybenzene and triphenyl phosphite as an internal standard. No formation of the desired N-thiophosphoryl azetidine **33** or difunctionalized product was detected under these conditions.

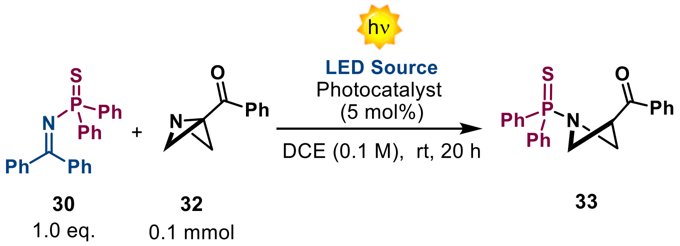

Entry[a]	Photocatalyst (5.0 mol%)	Λ (nm)	Recov. 32 (%)[b]	Yield 33(%)[b]
1	-	400	100%	n.d.
2	-	427	100%	n.d.
3	-	440	100%	n.d.
4	-	456	100%	n.d.
5	4CzIPN	456	0%	n.d.
6	Thioxanthone (TXO)	400	100%	n.d.
7	4DPAIPN	456	0%	n.d.

Table S1: [a]Reaction performed at 0.1 mmol scale, **30** (1.0 eq.), **32** (1.0 eq.), DCE (0.1 M), rt, 20 h. [b]^{1}H NMR yield using 1,3,5-trimethoxybenzene as internal standard. ^{31}P NMR yield using triphenyl phosphite as an internal standard.

3.1.3. Trials employing iminyl phosphine 31 as substrate.

Procedure: In an oven-dried 10.0 mL Schlenk tube containing an oven dried Teflon coated stirring bar was added photocatalyst (5.0 mol%), iminyl phosphine **31** (38.1 mg, 0.1 mmol, 1.0 eq.), azabicyclo[1.1.0]butane **32** (15.9 mg, 0.1 mmol, 1.0 eq.) and were dissolved using the anhydrous DCE (0.1 M, 1.0 mL, commercially available, 99.8% from Sigma-Aldrich).The Schlenk tubes were then closed with the proper greased glass stoppers. The resulting solution was subjected to three cycles of free-pump-thaw to remove the oxygen and in the last cycle, the vessel was filled with argon. The reaction was irradiated under blue LEDs for 20 hours with fan cooling. Afterwards, LED was removed and the solvent was evaporated under reduced pressure to afford the crude mixture and was analyzed by ^{1}H and ^{31}P NMR spectroscopy using 1,3,5-trimethoxybenzene and triphenyl phosphite as an internal standard. No formation of the desired N-phosphoryl azetidine **34** or difunctionalized product was detected under these conditions.

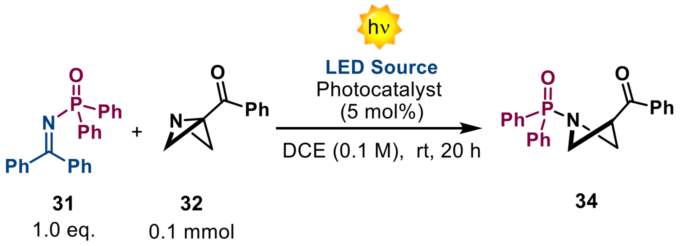

Entry[a]	Photocatalyst (5.0 mol%)	Λ (nm)	Recov. 32 (%)[b]	Yield 34(%)[b]
1	-	400	100%	n.d.
2	-	427	100%	n.d.
3	-	440	100%	n.d.
4	-	456	100%	n.d.
5	4CzIPN	456	0%	n.d.
6	Thioxanthone (TXO)	400	100%	n.d.
7	4DPAIPN	456	0%	n.d.

Table S2: [a]Reaction performed at 0.1 mmol scale, **31** (1.0 eq.), **32** (1.0 eq.), DCE (0.1 M), rt, 20 h. [b]^{1}H NMR yield using 1,3,5-trimethoxybenzene as internal standard. ^{31}P NMR yield using triphenyl phosphite as an internal standard.

3.2. Optimization

3.2.1. Optimization of the photocatalysts and other reaction parameters.

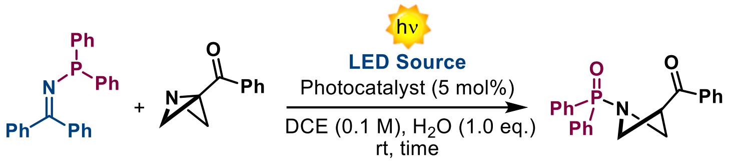

29 x.0 eq. **32** 0.1 mmol **34**

General procedure: In an oven-dried 10.0 mL Schlenk tube containing an oven dried Teflon coated stirring bar was added the corresponding photocatalyst (5.0 mol%), iminyl phosphine **29** (x.0 eq.) azabicyclo[1.1.0]butane **32** (15.9 mg, 0.1 mmol, 1.0 eq.) and were added and dissolved using the anhydrous DCE (1.0 mL, 0.1 M, commercially available, 99.8% from Sigma-Aldrich). The Schlenk tubes were then closed with the proper greased glass stoppers. The resulting solution was subjected to three cycles of free-pump-thaw to remove the oxygen and in the last cycle, the vessel was filled with argon. The reaction was irradiated under blue LED irradiation for desired hours with fan cooling. After completion, the resultant mixture was evaporated under reduced pressure to obtain the crude. ^{1}H NMR yield of the product was determined using 1,3,5-trimethoxybenzene as internal standard.

Employed photocatalysts (PCs)

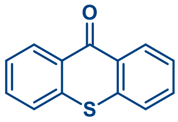

TXO

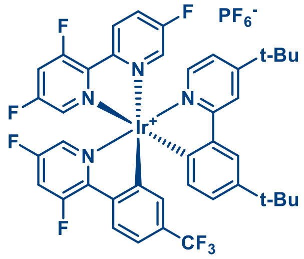

Ir[dF(CF$_3$)ppy]$_3$(dtbbpy)PF$_6$

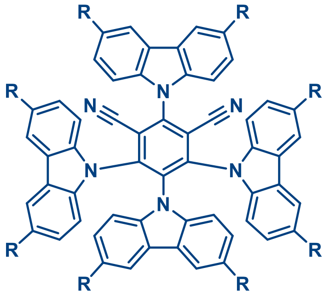

R= H; 4CzIPN
R= OMe; BisOMeCzIPN

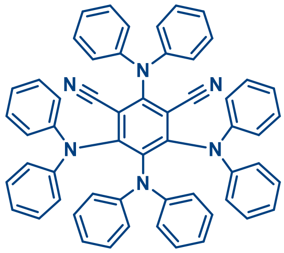

4DPAIPN

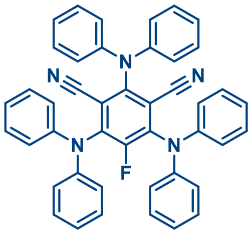

3DPAFIPN

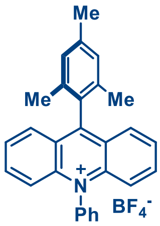

TAcr$^+$-MesBF$_4^-$

Entry[a]	Photocatalyst (5.0 mol%)	29 (eq.)	Λ (nm)	Time (h)	Yield 34 (%)[b]
1	Thioxanthone (TXO)	1.0	400	12	15
2	4CzIPN	1.0	456	12	18
3	4CzIPN	1.0	427	12	9
4	4CzIPN	3.0	456	12	28
5	Ir[dF(CF$_3$)ppy]$_3$(dtbbpy)PF$_6$	3.0	456	12	25
6	4CzIPN	4.0	456	20	33
7	4CzIPN	5.0	456	20	31
**8**	**4DPAIPN**	**4.0**	**456**	**20**	**49 (44)[c]**

9	3DPAFIPN	4.0	456	20	33
10	BisOMeCzIPN	4.0	456	20	36
11	$TAcr^+$-$MesBF_4^-$	4.0	456	20	25
12	4DPAIPN	4.0	456	16	44
13	4DPAIPN	5.0	456	20	48
14	-	4.0	456	20	n.d.
15	-	4.0	-	20	n.d.

Table S3: [a]Reaction performed at 0.1 mmol scale, **29** (x.0 eq), **32** (1.0 eq), photocatalyst (5.0 mol%). [b]^{1}H NMR yield using 1,3,5-trimethoxybenzene as internal standard. [c]Isolated yield. n.d. = not determined.

3.2.2. Screening of additives

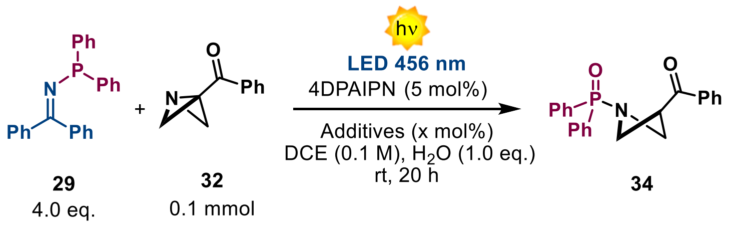

Entry[a]	Additives (mol%)	Yield 34 (%)[b]
1	Benzophenone, **35** (20.0)	44
2	$Ph_2P(O)H$, **36** (20.0)	32
3	$Ph_2P(O)NH_2$, **37** (20.0)	37
**4**	**$Ph_2P(O)OH$, 38 (20.0)**	**70 (66)**[c]
5	CH_3COOH (20.0)	59
6	C_6H_5COOH (20.0)	48
7	$Ph_2P(O)OH$, **38** (10.0)	45
8	$Ph_2P(O)OH$, **38** (100.0)	19
9	$Ph_2P(O)OH$, **38** (50.0)	55
10	$(Me_3Si)_3SiH$ (100.0 mol%)	49
11	γ-terpinene (100.0 mol%)	40
12	Hantzsch ester (100.0 mol%)	39
13	H_2O (1.0 eq.)	50
14	H_2O (4.0 eq.)	36
15	H_2O (10.0 eq.)	n.d.
16	Under air	n.d.

Table S4: [a]Reaction performed at 0.1 mmol scale, **29** (4.0 eq.), **32** (1.0 eq.), 4DPAIPN (5.0 mol%). [b]^{1}H NMR yield using 1,3,5-trimethoxybenzene as internal standard. [c]Isolated yield.

3.2.3. Screening of solvent concentration

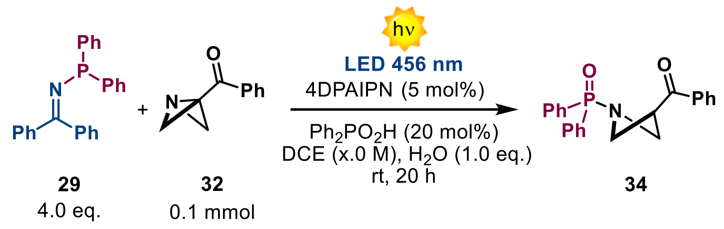

Entry[a]	Concentration (M)	Yield 34 (%)[b]
1	0.1	70
2	0.2	41
**3**	**0.05**	**80 (75)[c]**
4	0.025	38

Table S5: [a]Reaction performed at 0.1 mmol scale, **29** (4.0 eq.), **32** (1.0 eq.), 4DPAIPN (5.0 mol%). [b]^{1}H NMR yield using 1,3,5-trimethoxybenzene as internal standard. [c]Isolated yield.

3.2.4. Screening of solvents

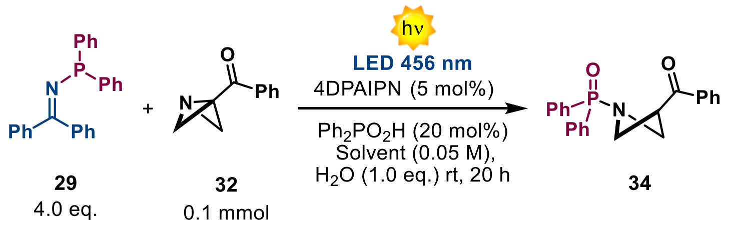

Entry[a]	Solvent (0.05 M)	Yield (%)[b]	Comments
**1**	**DCE**	**80 (75)[c]**	**-**
2	DCM	26	-
3	EtOAc	13	-
4	MeCN	16	Poor solubility
5	Toluene	21	Suspension
6	THF	n.d.	Not soluble
7	$PhCF_3$	11	-

Table S6: [a]Reaction performed at 0.1 mmol scale, **29** (4.0 eq.), **32** (1.0 eq.), 4DPAIPN (5.0 mol%). [b]1H NMR yield using 1,3,5-trimethoxybenzene as internal standard. [c]Isolated yield.

3.3. General procedure for the photochemical reactions of iminyl phosphines with azabicyclo[1.1.0]butanes.

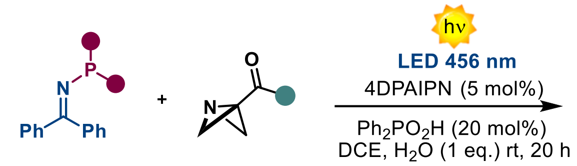

29 4.0 eq. | 32 1.0 eq. | 34

Procedure: In an oven-dried 10.0 mL Schlenk tube containing an oven dried Teflon coated stirring bar was added 4DPAIPN (4.0 mg, 5.0 mol%), diphenylphosphinic acid **38** (4.4 mg, 20.0 mol%), iminyl phosphine **29** (0.4 mmol, 4.0 eq.), the respective azabicyclo[1.1.0]butane (0.1 mmol, 1.0 eq.) and were dissolved using the anhydrous DCE (0.05 M, 2.0 mL, commercially available, 99.8% from Sigma-Aldrich). The Schlenk tubes were then closed with the proper greased glass stoppers. The resulting solution was subjected to three cycles of freeze-pump-thaw to remove the oxygen and in the last cycle, the vessel was filled with argon. The reaction was irradiated under 456 nm blue LEDs for 20 hours with fan cooling. Afterwards, LED was removed and the solvent was evaporated under reduced pressure to afford the crude mixture. The following was further purified by silica-gel column chromatography to afford the corresponding *N*-phosphoryl azetidines **(34, 45–61, 64, 65)**. ^{1}H NMR yield of the product was determined using 1,3,5-trimethoxybenzene as internal standard.

In cases where the ^{1}H NMR yield was difficult to determine accurately, triphenyl phosphite was used as an internal standard, and the yield was calculated by ^{31}P NMR spectroscopy.

Note: For compounds **34**, **46**, **47**, **53**, **54**, **55**, **58**, **62**, **63**, **66,** we attempted multiple purification using column chromatography and preparative TLC; however, multiple attempts for purifying these compounds led to minor decomposition of the products, resulting in co-elution with decomposition byproducts. Consequently, isolated yields are based on the product obtained after two purification attempts.

(1-(diphenylphosphoryl)azetidin-3-yl)(phenyl)methanone (34)

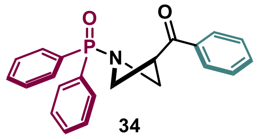

34

White solid; 27.2 mg, 0.075 mmol, 75% yield; Eluent: Hexane/EtOAc, 50/50 to 0/100, then Hexane/acetone 0/100; **^{1}H NMR (400 MHz, CDCl$_3$) *δ*:** 7.89 (dd, *J* = 11.0, 7.6 Hz, 4H), 7.79 (d, *J* = 7.4 Hz, 2H), 7.58 – 7.52 (m, 1H), 7.51 – 7.39 (m, 8H), 4.35 – 4.25 (m, 1H), 4.19 (app. q, *J* = 9.1 Hz, 2H), 4.02 (app. q, *J* = 7.2 Hz, 2H) ppm; **^{13}C NMR (101 MHz, CDCl$_3$) *δ*:** 197.5, 134.7, 133.8, 132.4 (d, *J* = 9.1 Hz), 132.1 (d, *J* = 2.9 Hz), 130.8 (d, *J* = 129.7 Hz), 129.0, 128.8 (d, *J* = 12.4 Hz), 128.4, 48.4 (d, *J* = 3.6 Hz), 38.0 (d, *J* = 14.5 Hz) ppm; **^{31}P NMR (162 MHz, CDCl$_3$) *δ*:** 25.8 ppm; **HRMS (ESI)**: m/z calcd. for $C_{22}H_{21}NO_2P$ [M+H]$^+$: 362.1310, found: 362.1299.

(1-(diphenylphosphoryl)azetidin-3-yl)(o-tolyl)methanone (46)

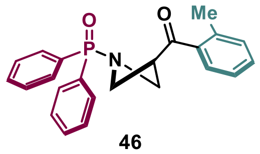

46

Pale-yellow solid; 21.1 mg, 0.056 mmol, 56% yield; Eluent: Hexane/EtOAc, 50/50 to 0/100, then Hexane/acetone 0/100; **^{1}H NMR (400 MHz, CDCl$_3$) *δ*:** 7.90 (dd, *J* = 11.0, 7.6 Hz, 4H), 7.53 – 7.33 (m, 8H), 7.28 (s, 1H), 7.24 – 7.16 (m, 1H), 4.32 – 4.20 (m, 1H), 4.12 (app. q, *J* = 9.2 Hz, 2H), 3.99 (app. q, *J* = 7.5 Hz, 2H), 2.59 (s, 3H) ppm; **^{13}C NMR (101 MHz, CDCl$_3$) *δ*:** 200.7, 140.0, 134.8, 132.7, 132.4 (d, *J* = 9.1 Hz), 132.3, 132.1 (d, *J* = 2.9 Hz), 130.9 (d, *J* = 129.0 Hz), 129.1, 128.8 (d, *J* = 12.4 Hz), 126.0, 48.7 (d, *J* = 3.6 Hz), 39.7 (d, *J* = 14.5 Hz), 22.0 ppm; **^{31}P NMR (162 MHz, CDCl$_3$) *δ*:** 25.8 ppm; **HRMS (ESI)**: m/z calcd. for $C_{23}H_{23}NO_2P$ [M+H]$^+$: 376.1466, found: 376.1457.

(1-(diphenylphosphoryl)azetidin-3-yl)(m-tolyl)methanone (47)

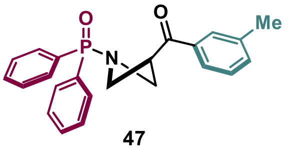

47

Pale-yellow solid; 30.1 mg, 0.080 mmol, 80% yield; Eluent: Hexane/EtOAc, 50/50 to 0/100, then Hexane/acetone 0/100; **^{1}H NMR (400 MHz, $CDCl_3$) *δ*:** 7.89 (dd, *J* = 11.8, 7.3 Hz, 4H), 7.54 – 7.40 (m, 6H), 7.40 – 7.29 (m, 2H), 7.20 – 7.09 (m, 2H), 4.34 – 4.25 (m, 1H), 4.19 (app. q, *J* = 8.7 Hz, 2H), 4.02 (app. q, *J* = 7.4 Hz, 2H), 2.38 (s, 3H) ppm; **^{13}C NMR (101 MHz, $CDCl_3$) *δ*:** 197.8, 138.9, 134.8, 134.6, 132.5 (d, *J* = 9.4 Hz), 132.1 (d, *J* = 2.9 Hz), 130.8 (d, *J* = 129.0 Hz), 128.9, 128.8 (d, *J* = 12.7 Hz), 128.5, 125.6, 48.5 (d, *J* = 3.6 Hz), 38.1 (d, *J* = 14.5 Hz), 21.5 ppm; **^{31}P NMR (162 MHz, $CDCl_3$) *δ*:** 25.8 ppm; **HRMS (ESI)**: m/z calcd. for $C_{23}H_{23}NO_2P$ $[M+H]^+$: 376.1466, found: 376.1457.

(1-(diphenylphosphoryl)azetidin-3-yl)(p-tolyl)methanone (48)

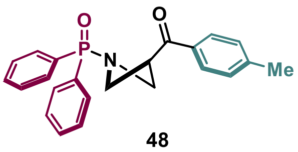

48

Pale-yellow solid; 27.5 mg, 0.073 mmol, 73% yield; Eluent: Hexane/EtOAc, 50/50 to 0/100, then Hexane/acetone 0/100; **^{1}H NMR (400 MHz, $CDCl_3$) *δ*:** 7.92 – 7.85 (m, 4H), 7.69 (d, *J* = 8.3 Hz, 2H), 7.53 – 7.39 (m, 6H), 7.24 (d, *J* = 8.2 Hz, 2H), 4.33 – 4.23 (m, 1H), 4.22 – 4.14 (m, 2H), 4.02 (app. q, *J* = 7.3 Hz, 1H), 2.39 (s, 3H) ppm; **^{13}C NMR (101 MHz, $CDCl_3$) *δ*:** 197.3, 144.7, 132.4 (d, *J* = 9.4 Hz), 132.3, 132.1 (d, *J* = 2.9 Hz), 130.7 (d, *J* = 129.3 Hz), 129.7, 128.8 (d, *J* = 12.4 Hz), 128.5, 48.5 (d, *J* = 3.6 Hz), 37.9 (d, *J* = 14.9 Hz), 21.8 ppm; **^{31}P NMR (162 MHz, $CDCl_3$) *δ*:** 25.9 ppm; **HRMS (ESI)**: m/z calcd. for $C_{23}H_{23}NO_2P$ $[M+H]^+$: 376.1466, found: 376.1457.

(1-(diphenylphosphoryl)azetidin-3-yl)(4-methoxyphenyl)methanone (49)

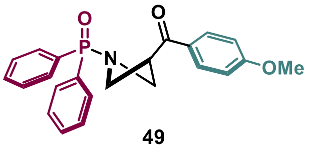

49

Pale-yellow solid; 20.0 mg, 0.051 mmol, 51% yield; Eluent: Hexane/EtOAc, 50/50 to 0/100, then Hexane/acetone 0/100; **^{1}H NMR (400 MHz, $CDCl_3$) *δ*:** 7.94 – 7.85 (m, 4H), 7.77 (d, *J* = 8.9 Hz, 2H), 7.52 – 7.37 (m, 6H), 6.91 (d, *J* = 8.9 Hz, 2H), 4.31 – 4.12 (m, 3H), 4.02 (app. q, *J* = 7.2 Hz, 2H), 3.84 (s, 3H) ppm; **^{13}C NMR (101 MHz, $CDCl_3$) *δ*:** 196.2, 164.0, 132.4 (d, *J* = 9.3 Hz), 132.1 (d, *J* = 2.9 Hz), 130.8 (d, *J* = 129.3 Hz), 130.7, 128.8 (d, *J* = 12.4 Hz), 127.9, 114.2, 55.7, 48.5 (d, *J* = 3.5 Hz), 37.7 (d, *J* = 14.5 Hz) ppm; **^{31}P NMR (162 MHz, $CDCl_3$) *δ*:** 25.8 ppm; **HRMS (ESI)**: m/z calcd. for $C_{23}H_{23}NO_3P$ $[M+H]^+$: 392.1416, found: 392.1401.

(1-(diphenylphosphoryl)azetidin-3-yl)(4-(trifluoromethyl)phenyl)methanone (50)

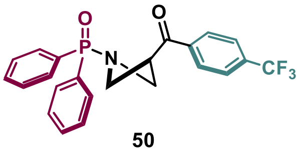

50

Pale-yellow solid; 24.4 mg, 0.057 mmol, 57% yield; Eluent: Hexane/EtOAc, 50/50 to 0/100, then Hexane/acetone 0/100; **^{1}H NMR (400 MHz, $CDCl_3$) *δ*:** 7.94 – 7.85 (m, 6H), 7.72 (d, *J* = 8.1 Hz, 2H), 7.53 – 7.40 (m, 6H), 4.35 – 4.27 (m, 1H), 4.21 (app. q, *J* = 8.6 Hz, 2H), 4.03 (app. q, *J* = 7.5 Hz, 2H) ppm; **^{13}C NMR (101 MHz, $CDCl_3$) *δ*:** 196.5, 137.3, 135.0 (d, *J* = 32.7 Hz), 132.3 (d, *J* = 9.1 Hz), 132.1 (d, *J* = 2.9 Hz), 130.4 (d, *J* = 129.3 Hz), 128.8, 128.7 (d, *J* = 3.6 Hz), 126.0 (q, *J* = 3.6 Hz), 123.4 (q, *J* = 272.1 Hz), 48.1 (d, *J* = 3.6 Hz), 38.1 (d, *J* = 14.5 Hz) ppm; **^{31}P NMR (162 MHz, $CDCl_3$) *δ*:** 26.2 ppm; **^{19}F NMR (377 MHz, $CDCl_3$) *δ*:** 63.8 ppm; **HRMS (ESI)**: m/z calcd. for $C_{23}H_{19}F_3NO_2P$ $[M+H]^+$: 430.1184, found: 430.1344.

(4-chlorophenyl)(1-(diphenylphosphoryl)azetidin-3-yl)methanone (51)

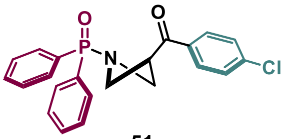

51

Pale-yellow solid; 24.1 mg, 0.061 mmol, 61% yield; Eluent: Hexane/EtOAc, 50/50 to 0/100, then Hexane/acetone 0/100; **^{1}H NMR (400 MHz, $CDCl_3$) *δ*:** 7.88 (dd, *J* = 11.5, 7.4 Hz, 4H), 7.73 (d, *J* = 8.5 Hz, 2H), 7.53 – 7.38 (m, 8H), 4.31 – 4.22 (m, 1H), 4.17 (app. q, *J* = 8.5 Hz, 2H), 4.01 (app. q, *J* = 7.4 Hz, 2H) ppm; **^{13}C NMR (101 MHz, $CDCl_3$) *δ*:** 196.4, 140.3, 133.1, 132.4 (d, *J* = 9.1 Hz), 132.2 (d, *J* = 2.9 Hz), 130.6 (d, *J* = 129.3 Hz), 129.8, 129.4, 128.8 (d, *J* = 12.4 Hz), 48.3 (d, *J* = 3.6 Hz), 38.0 (d, *J* = 14.5 Hz) ppm; **^{31}P NMR (162 MHz, $CDCl_3$) *δ*:** 26.0 ppm; **HRMS (ESI)**: m/z calcd. for $C_{22}H_{20}ClNO_2P$ $[M+H]^+$: 396.0920, found: 396.1107.

(1-(diphenylphosphoryl)azetidin-3-yl)(4-fluorophenyl)methanone (52)

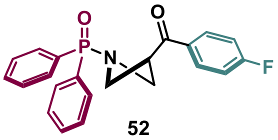

52

Pale-yellow solid; 23.3 mg, 0.061 mmol, 61% yield; Eluent: Hexane/EtOAc, 50/50 to 0/100, then Hexane/acetone 0/100; **^{1}H NMR (400 MHz, $CDCl_3$) *δ*:** 7.94 – 7.78 (m, 6H), 7.53 – 7.38 (m, 6H), 7.16 – 7.07 (m, 2H), 4.32 – 4.13 (m, 3H), 4.02 (app. q, *J* = 7.3 Hz, 2H) ppm; **^{13}C NMR (101 MHz, $CDCl_3$) *δ*:** 196.0, 166.1 (d, *J* = 255.8 Hz), 132.4 (d, *J* = 9.1 Hz), 132.2 (d, *J* = 2.9 Hz), 131.2 (d, *J* = 3.3 Hz), 131.1 (d, *J* = 9.4 Hz), 130.7 (d, *J* = 129.3 Hz), 128.8 (d, *J* = 12.4 Hz), 116.2 (d, *J* = 21.8 Hz), 48.3 (d, *J* = 3.6 Hz), 37.92 (d, *J* = 14.5 Hz) ppm; **^{31}P NMR (162 MHz, $CDCl_3$) *δ*:** 25.9 ppm; **^{19}F NMR (377 MHz, $CDCl_3$) *δ*:** -104.4 ppm; **HRMS (ESI)**: m/z calcd. for $C_{22}H_{20}FNO_2P$ $[M+H]^+$: 380.1216, found: 380.1205.

(1-(diphenylphosphoryl)azetidin-3-yl)(naphthalen-2-yl)methanone (53)

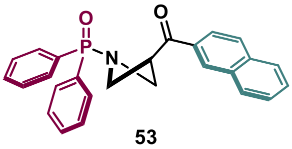

53

Pale-yellow solid; 25.5 mg, 0.062 mmol, 62% yield; Eluent: Hexane/EtOAc, 50/50 to 0/100, then Hexane/acetone 0/100; **^{1}H NMR (400 MHz, $CDCl_3$) *δ*:** 8.25 (s, 1H), 7.99 – 7.82 (m, 8H), 7.64 – 7.52 (m, 2H), 7.51 – 7.39 (m, 6H), 4.54 – 4.43 (m, 1H), 4.28 (app. q, *J* = 9.2 Hz, 2H), 4.09 (app. q, *J* = 7.4 Hz, 2H) ppm; **^{13}C NMR (101 MHz, $CDCl_3$) *δ*:** 197.6, 135.9, 132.6, 132.4 (d, *J* = 9.3 Hz), 132.2 (d, *J* = 2.5 Hz), 132.1, 130.7 (d, *J* = 130.3 Hz), 130.3, 129.7, 128.8 (d, *J* = 12.4 Hz), 128.0, 127.1, 123.9, 48.6 (d, *J* = 3.5 Hz), 38.1 (d, *J* = 14.4 Hz) ppm; **^{31}P NMR (162 MHz, $CDCl_3$) *δ*:** 26.1 ppm; **HRMS (ESI)**: m/z calcd. for $C_{26}H_{23}NO_2P$ $[M+H]^+$: 412.1466, found: 412.1453.

(1-(diphenylphosphoryl)azetidin-3-yl)(pyridin-2-yl)methanone (54)

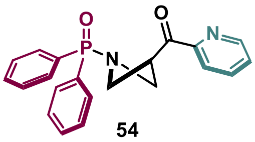

54

Black solid; 19.3 mg, 0.053 mmol, 53% yield; Eluent: Hexane/EtOAc, 50/50 to 0/100, then Hexane/acetone 0/100; **^{1}H NMR (400 MHz, $CDCl_3$) *δ*:** 8.56 (d, *J* = 5.3 Hz, 1H), 8.08 (d, *J* = 7.8 Hz, 1H), 7.95 – 7.78 (m, 5H), 7.52 – 7.38 (m, 7H), 4.70 – 4.56 (m, 1H), 4.18 (app. q, *J* = 9.3 Hz, 2H), 4.03 (app. q, *J* = 8.0 Hz, 2H) ppm; **^{13}C NMR (101 MHz, $CDCl_3$) *δ*:** 199.1, 152.0, 149.2, 137.1, 132.5 (d, *J* = 9.1 Hz), 132.0 (d, *J* = 2.9 Hz), 131.1 (d, *J* = 129.0 Hz), 128.7 (d, *J* = 12.4 Hz), 127.5, 122.5, 48.7 (d, *J* = 3.6 Hz), 38.3 (d, *J* = 15.3 Hz) ppm; **^{31}P NMR (162 MHz, $CDCl_3$) *δ*:** 25.3 ppm; **HRMS (ESI)**: m/z calcd. for $C_{21}H_{20}N_2O_2P$ $[M+H]^+$: 363.1262, found: 363.1264.

(1-(diphenylphosphoryl)azetidin-3-yl)(pyridin-3-yl)methanone (55)

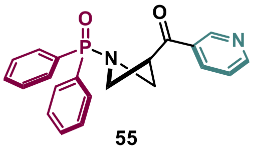

55

Black solid; 16.3 mg, 0.045 mmol, 45% yield; Eluent: Hexane/EtOAc, 50/50 to 0/100, then Hexane/acetone 0/100; **^{1}H NMR (400 MHz, $CDCl_3$) *δ*:** 8.97 (s, 1H), 8.77 (d, *J* = 4.7 Hz, 1H), 8.13 (d, *J* = 7.9 Hz, 1H), 7.89 (dd, *J* = 11.7, 8.0 Hz, 4H), 7.54 – 7.37 (m, 7H), 4.29 (d, *J* = 7.2 Hz, 1H), 4.20 (app. q, *J* = 9.4 Hz, 2H), 4.05 (app. q, *J* = 7.6 Hz, 2H) ppm; **^{13}C NMR (101 MHz, $CDCl_3$) *δ*:** 196.5, 154.2, 149.9, 135.8, 132.4 (d, *J* = 9.4 Hz), 132.3 (d, *J* = 2.9 Hz), 130.5 (d, *J* = 129.3 Hz), 130.2, 128.9 (d, *J* = 12.4 Hz), 124.1, 48.1 (d, *J* = 3.6 Hz), 38.1 (d, *J* = 14.5 Hz) ppm; **^{31}P NMR (162 MHz, $CDCl_3$) *δ*:** 26.1 ppm; **HRMS (ESI)**: m/z calcd. for $C_{21}H_{20}N_2O_2P$ $[M+H]^+$: 363.1262, found: 363.1264.

(1-(diphenylphosphoryl)azetidin-3-yl)(furan-2-yl)methanone (56)

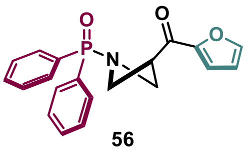

56

Pale-yellow solid; 18.5 mg, 0.053 mmol, 53% yield; Eluent: Hexane/EtOAc, 50/50 to 0/100, then Hexane/acetone 0/100; **^{1}H NMR (400 MHz, $CDCl_3$) *δ*:** 7.90 (dd, *J* = 11.7, 8.0 Hz, 4H), 7.55 (s, 1H), 7.52 – 7.41 (m, 6H), 7.17 (d, *J* = 3.6 Hz, 1H), 6.55 – 6.51 (m, 1H), 4.19 – 4.10 (m, 3H), 4.06 – 3.98 (m, 2H) ppm; **^{13}C NMR (101 MHz, $CDCl_3$) *δ*:** 187.0, 151.6, 147.0, 132.5 (d, *J* = 9.3 Hz), 132.2 (d, *J* = 2.4 Hz), 130.7 (d, *J* = 129.1 Hz), 128.8 (d, *J* = 12.4 Hz), 118.0, 112.6, 48.2 (d, *J* = 3.6 Hz), 37.9 (d, *J* = 14.9 Hz) ppm; **^{31}P NMR (162 MHz, $CDCl_3$) *δ*:** 25.7 ppm; **HRMS (ESI)**: m/z calcd. for $C_{20}H_{19}NO_3P$ $[M+H]^+$: 352.1103, found: 352.1100.

(1-(diphenylphosphoryl)azetidin-3-yl)(thiophen-2-yl)methanone (57)

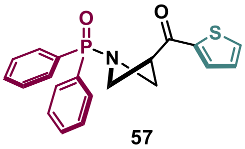

57

Pale-yellow solid; 19.2 mg, 0.052 mmol, 52% yield; Eluent: Hexane/EtOAc, 50/50 to 0/100, then Hexane/acetone 0/100; **^{1}H NMR (400 MHz, $CDCl_3$) δ:** 7.90 (dd, J = 11.8, 8.0 Hz, 4H), 7.66 (d, J = 4.9 Hz, 1H), 7.54 – 7.36 (m, 7H), 7.12 – 7.06 (m, 1H), 4.27 – 4.12 (m, 3H), 4.04 (app. q, J = 5.8 Hz, 2H) ppm; **^{13}C NMR (101 MHz, $CDCl_3$) δ:** 190.8, 142.2, 134.5, 132.4 (d, J = 9.4 Hz), 132.3, 132.2 (d, J = 2.9 Hz), 130.7 (d, J = 129.3 Hz), 128.8 (d, J = 12.4 Hz), 128.5, 48.6 (d, J = 3.6 Hz), 38.5 (d, J = 14.5 Hz) ppm; **^{31}P NMR (162 MHz, $CDCl_3$) δ:** 25.9 ppm; **HRMS (ESI)**: m/z calcd. for $C_{20}H_{19}NO_2PS$ $[M+H]^+$: 368.0874, found: 368.0862.

(4-(1H-1,2,4-triazol-1-yl)phenyl)(1-(diphenylphosphoryl)azetidin-3-yl)methanone (58)

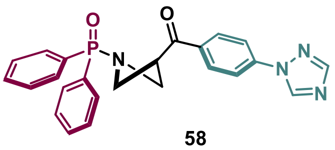

58

Pale-yellow solid; 27.0 mg, 0.063 mmol, 63% yield; Eluent: Hexane/EtOAc, 50/50 to 0/100, then Hexane/acetone 0/100; **^{1}H NMR (400 MHz, $CDCl_3$) δ:** 8.65 (s, 1H), 8.12 (s, 1H), 7.98 – 7.85 (m, 6H), 7.81 (d, J = 8.5 Hz, 2H), 7.52 – 7.37 (m, 6H), 4.37 – 4.26 (m, 1H), 4.20 (app. q, J = 9.3 Hz, 2H), 4.05 (app. q, J = 7.5 Hz, 2H) ppm; **^{13}C NMR (101 MHz, $CDCl_3$) δ:** 196.1, 153.3, 140.6, 134.0, 132.4 (d, J = 9.4 Hz), 132.2 (d, J = 2.9 Hz), 130.6 (d, J = 129.3 Hz), 130.4, 130.3, 128.8 (d, J = 12.4 Hz), 119.8, 48.3 (d, J = 4.0 Hz), 38.1 (d, J = 14.5 Hz) ppm; **^{31}P NMR (162 MHz, $CDCl_3$) δ:** 26.0 ppm; **HRMS (ESI)**: m/z calcd. for $C_{24}H_{22}N_4O_2P$ $[M+H]^+$: 429.1480, found: 429.1472.

(4-(1H-naphtho[1,2-e][1,3]oxazin-2(3H)-yl)phenyl)(1-(diphenylphosphoryl)azetidin-3-yl)methanone (59)

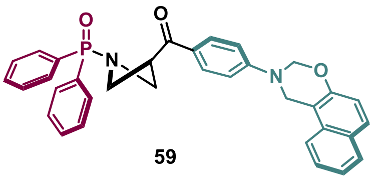

59

Yellow solid; 37.3 mg, 0.068 mmol, 68% yield; Eluent: Hexane/EtOAc, 50/50 to 0/100, then Hexane/acetone 0/100; **^{1}H NMR (400 MHz, $CDCl_3$) δ:** 7.88 (dd, J = 11.1, 7.5 Hz, 4H), 7.81 – 7.62 (m, 6H), 7.56 – 7.50 (m, 1H), 7.49 – 7.36 (m, 6H), 7.12 (d, J = 8.8 Hz, 2H), 7.04 (d, J = 8.9 Hz, 1H), 5.46 (s, 2H), 5.00 (s, 2H), 4.25 – 4.11 (m, 3H), 3.98 (app. q, J = 6.3 Hz, 2H) ppm; **^{13}C NMR (101 MHz, $CDCl_3$) δ:** 195.9, 152.5, 152.2, 132.4 (d, J = 9.1 Hz), 132.1 (d, J = 2.9 Hz), 131.1, 130.8 (d, J = 129.3 Hz), 130.5, 129.2, 128.9, 128.8, 128.8 (d, J = 12.4 Hz), 128.5, 127.1, 127.0, 124.1, 120.8, 118.7, 116.1, 112.2, 48.5 (d, J = 3.6 Hz), 47.6, 37.6 (d, J = 14.5 Hz) ppm; **^{31}P NMR (162 MHz, $CDCl_3$) δ:** 25.6 ppm; **HRMS (ESI)**: m/z calcd. for $C_{34}H_{30}N_2O_3P$ $[M+H]^+$: 545.1994, found: 545.1986.

5-(5-(1-(diphenylphosphoryl)azetidine-3-carbonyl)-4-methylthiazol-2-yl)-2-isobutoxybenzonitrile (60)

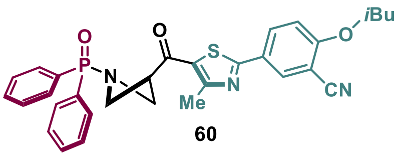

Pale-yellow solid; 33.9 mg, 0.061 mmol, 61% yield; Eluent: Hexane/EtOAc, 50/50 to 0/100, then Hexane/acetone 0/100; **^{1}H NMR (400 MHz, $CDCl_3$) δ:** 8.17 (s, 1H), 8.06 (d, J = 8.9 Hz, 1H), 7.90 (dd, J = 12.0, 7.3 Hz, 4H), 7.55 – 7.34 (m, 6H), 7.00 (d, J = 8.7 Hz, 1H), 4.23 – 4.10 (m, 2H), 4.09 – 3.97 (m, 3H), 3.89 (d, J = 6.5 Hz, 2H), 2.77 (s, 3H), 2.20 (app. spt, J = 6.7 Hz, 1H), 1.08 (d, J = 6.7 Hz, 6H) ppm; **^{13}C NMR (101 MHz, $CDCl_3$) δ:** 190.3, 167.4, 162.9, 161.3, 132.8, 132.4 (d, J = 9.1 Hz), 132.4, 132.2 (d, J = 2.9 Hz), 130.6 (d, J = 129.3 Hz), 128.9 (d, J = 12.4 Hz), 128.4, 125.7, 115.4, 112.8, 103.3, 75.9, 48.1 (d, J = 3.6 Hz), 41.2 (d, J = 14.9 Hz), 28.3, 19.2, 18.5 ppm; **^{31}P NMR (162 MHz, $CDCl_3$) δ:** 26.0 ppm; **HRMS (ESI)**: m/z calcd. for $C_{31}H_{31}N_3O_3PS$ $[M+H]^+$: 556.1824, found: 556.1814.

(2-(3,5-dichlorophenyl)benzo[d]oxazol-6-yl)(1-(diphenylphosphoryl)azetidin-3-yl)methanone (61)

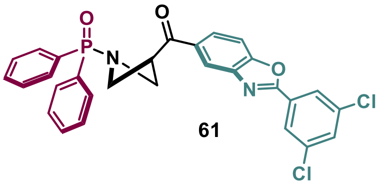

61

White solid; 33.5 mg, 0.061 mmol, 61% yield; Eluent: Hexane/EtOAc, 50/50 to 0/100, then Hexane/acetone 0/100; **^{1}H NMR (400 MHz, CDCl$_3$) *δ*:** 8.14 (s, 2H), 8.08 (s, 1H), 7.91 (dd, *J* = 11.4, 7.4 Hz, 4H), 7.81 (s, 2H), 7.55 (s, 1H), 7.52 – 7.40 (m, 6H), 4.43 – 4.32 (m, 1H), 4.24 (app. q, *J* = 9.0 Hz, 1H), 4.08 (app. q, *J* = 7.8 Hz, 2H) ppm; **^{13}C NMR (101 MHz, CDCl$_3$) *δ*:** 196.3, 163.5, 151.0, 146.1, 136.2, 132.7, 132.4 (d, *J* = 9.4 Hz), 132.2, 132.2 (d, *J* = 2.5 Hz), 130.7 (d, *J* = 129.0 Hz), 129.2, 128.8 (d, *J* = 12.4 Hz), 126.4, 125.7, 120.7, 111.2, 48.5 (d, *J* = 4.0 Hz), 38.3 (d, *J* = 14.5 Hz) ppm; **^{31}P NMR (162 MHz, CDCl$_3$) *δ*:** 25.9 ppm; **HRMS (ESI)**: m/z calcd. for $C_{29}H_{22}Cl_2N_2O_3P$ [M+H]$^+$: 547.0745, found: 547.0743.

diethyl (3-benzoylazetidin-1-yl)phosphonate (45)

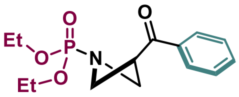

45

Yellow solid; 12.4 mg, 0.049 mmol, 49% yield; Eluent: Hexane/EtOAc, 50/50 to 0/100, then Hexane/acetone 0/100; **^{1}H NMR (400 MHz, CDCl$_3$) *δ*:** 1H NMR (400 MHz, CDCl3) δ 7.82 (d, *J* = 7.4 Hz, 2H), 7.59 (t, *J* = 7.4 Hz, 1H), 7.48 (t, *J* = 7.7 Hz, 2H), 4.34 – 4.23 (m, 1H), 4.22 – 4.03 (m, 8H), 1.33 (t, *J* = 7.1 Hz, 6H) ppm; **^{13}C NMR (101 MHz, CDCl$_3$) *δ*:** 197.38, 134.88, 133.83, 129.07, 128.37, 62.85 (d, *J* = 5.9 Hz), 50.68 (d, *J* = 1.5 Hz), 38.15 (d, *J* = 17.7 Hz), 16.44 (d, *J* = 6.5 Hz); **^{31}P NMR (162 MHz, CDCl$_3$) *δ*:** 7.0 ppm; **HRMS (ESI)**: m/z calcd. for $C_{14}H_{21}NO_4P$ [M+H]$^+$: 298.1208, found: 298.1212.

diethyl (3-picolinoylazetidin-1-yl)phosphonate (65)

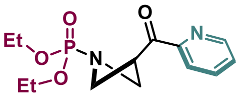

65

Black solid; 12.3 mg, 0.041 mmol, 41% yield; Eluent: Hexane/EtOAc, 50/50 to 0/100, then Hexane/acetone 0/100; **^{1}H NMR (400 MHz, CDCl$_3$) *δ*:** 8.63 (d, *J* = 4.4 Hz, 1H), 8.09 (d, *J* = 8.0 Hz, 1H), 7.90 – 7.82 (m, 1H), 7.51 – 7.45 (m, 1H), 4.68 – 4.57 (m, 1H), 4.23 – 4.03 (m, 8H), 1.33 (t, *J* = 7.1 Hz, 6H) ppm; **^{13}C NMR (101 MHz, CDCl$_3$) *δ*:** 199.1, 152.0, 149.3, 137.2, 127.6, 122.6, 62.7 (d, *J* = 5.8 Hz), 50.8 (d, *J* = 2.2 Hz), 38.2 (d, *J* = 17.8 Hz), 16.4 (d, *J* = 6.5 Hz) ppm; **^{31}P NMR (162 MHz, CDCl$_3$) *δ*:** 7.3 ppm; **HRMS (ESI)**: m/z calcd. for $C_{13}H_{20}N_2O_4P$ [M+H]$^+$: 299.1161, found: 299.1279.

diethyl (3-(2-(3-cyano-4-isobutoxyphenyl)-4-methylthiazole-5-carbonyl)azetidin-1-yl)phosphonate (66)

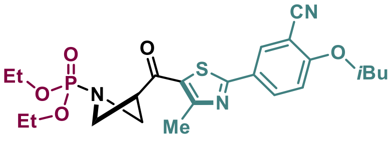

66

Yellow solid; 27.1 mg, 0.055 mmol, 55% yield; Eluent: Hexane/EtOAc, 50/50 to 0/100, then Hexane/acetone 0/100; **^{1}H NMR (400 MHz, CDCl$_3$) *δ*:** 8.19 (d, *J* = 2.0 Hz, 1H), 8.10 (dd, *J* = 8.8, 2.0 Hz, 1H), 7.02 (d, *J* = 8.9 Hz, 1H), 4.19 (app. q, *J* = 6.6 Hz, 2H), 4.14 – 4.07 (m, 6H), 4.04 – 3.96 (m, 1H), 3.91 (d, *J* = 6.5 Hz, 2H), 2.79 (s, 3H), 2.21 (app. spt, *J* = 6.7 Hz, 1H), 1.34 (t, *J* = 7.0 Hz, 6H), 1.10 (d, *J* = 6.7 Hz, 6H) ppm; **^{13}C NMR (150 MHz, CDCl$_3$) *δ*:** 190.0, 167.4, 163.0, 161.3, 132.9, 132.5, 130.2, 128.4, 125.7, 112.8, 103.3, 75.9, 62.9 (d, *J* = 5.9 Hz), 50.4, 41.4 (d, *J* = 17.9 Hz), 28.3, 19.2, 18.5, 16.5 (d, *J* = 6.5 Hz) ppm; **^{31}P NMR (162 MHz, CDCl$_3$) *δ*:** 6.9 ppm; **HRMS (ESI)**: m/z calcd. for $C_{23}H_{31}N_3O_5PS$ [M+H]$^+$: 492.1722, found: 492.1703.

3.4. Unsuccessful azabicyclo[1.1.0]butanes for the photochemical reaction.

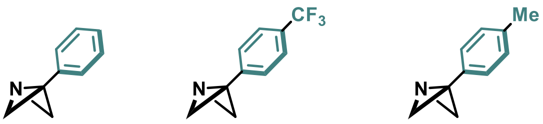

S18 S19 S20

3.5. Three component reaction (In-situ generation of iminyl phosphines).

After establishing the reaction condition for two-component photoredox process for the synthesis of N-phosphoryl azetidines and practical challenges associated with isolating some of the iminyl phosphines by column chromatography, we sought to develop a three-component reaction manifold in which the iminyl phosphine **29** is generated in-situ from commercially available benzophenone imine **19** and chloro-diaryl or -dialkyl phosphines. Notably, this multicomponent protocol proceeded smoothly to afford the model azetidine **34** in comparable yields to that obtained under the two-component conditions in the absence of additive **38**. We have performed a small optimization of the reaction parameters (see below).

3.5.1. Optimization of base and other parameters.

Procedure: In an oven-dried 10.0 mL Schlenk tube containing an oven dried Teflon coated stirring bar was added benzophenone imine **19** (x.0 eq.), the corresponding base (y.0 eq.) and chlorodiphenylphosphine **2** (z.0 eq.) and were dissolved using anhydrous DCE (1.0 mL, 0.1 M, commercially available, 99.8% from Sigma-Aldrich). After 30 minutes of stirring under argon, azabicyclo[1.1.0]butane **32** (0.1 mmol, 1.0 eq.) and 4DPAIPN (4.0 mg, 5.0 mol%) were added and dissolved using anhydrous DCE (0.05 M, 1.0 mL, commercially available, 99.8% from Sigma-Aldrich). The Schlenk tubes were then closed with the proper greased glass stoppers. The resulting solution was subjected to three cycles of freeze-pump-thaw to remove the oxygen and in the last cycle, the vessel was filled with argon. The reaction was irradiated under 456 nm blue LEDs for 20 hours with fan cooling. Afterwards, LED was removed and the solvent was evaporated under reduced pressure to afford the crude mixture. The following was further purified by silica-gel column chromatography to afford the corresponding *N*-phosphoryl azetidines. ^{1}H NMR yield of the product was determined using 1,3,5-trimethoxybenzene as internal standard. In cases where the ^{1}H NMR yield was difficult to determine accurately, triphenyl phosphite was used as an internal standard, and the yield was calculated by ^{31}P NMR spectroscopy.

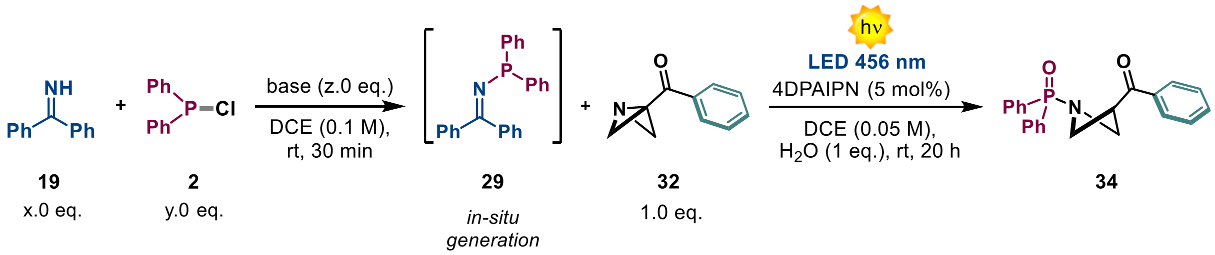

Entry[a]	19 (eq.)	2 (eq.)	Base	Yield 34 (%)[b]
1	4.0	4.0	Et_3N (4.0 eq.)	14
2	4.0	4.0	DBU (4.0 eq.)	39
3	4.0	4.0	DIPEA (4.0 eq.)	49 (43)[c]
**4**	**2.0**	**2.0**	**DIPEA (2.0 eq.)**	**51 (50)[c]**
5	0.5	2.0	DIPEA (4.0 eq.)	38
6	1.0	2.0	DIPEA (2.0 eq.)	35
7[d]	2.0	2.0	DIPEA (2.0 eq)	trace

Table S7: [a]Reaction performed at 0.1 mmol scale, **32** (1.0 eq.), 4DPAIPN (5.0 mol%). [b]^{1}H NMR yield using 1,3,5-trimethoxybenzene as internal standard. [c]Isolated yield. [d]Reaction performed with 20.0 mol% of Ph_2PO_2H.

3.5.2. General procedure for the three component photochemical reactions with azabicyclo[1.1.0]butanes.

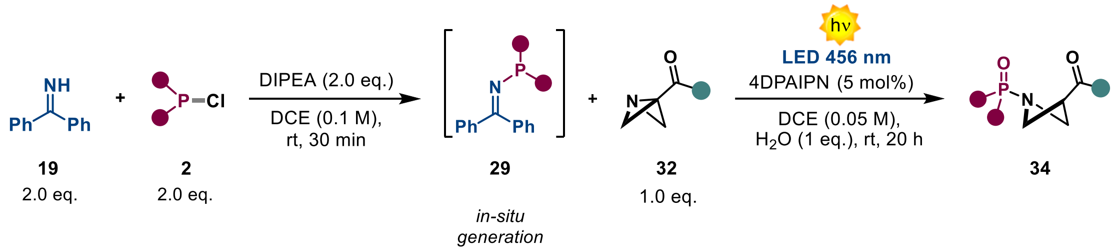

Procedure: In an oven-dried 10.0 mL Schlenk tube containing an oven dried Teflon coated stirring bar was added benzophenone imine **19** (0.2 mmol, 2.0 eq.), N,N-diisopropyléthylamine (0.2 mmol, 2.0 eq.) and chlorophosphine (0.2 mmol, 2 eq.) and were dissolved in anhydrous DCE (0.1 M, 1.0 mL, commercially available, 99.8% from Sigma-Aldrich). After 30 minutes of stirring under argon, azabicyclo[1.1.0]butane (0.1 mmol, 1.0 eq.) and 4DPAIPN (4.0 mg, 5.0 mol%) were added and dissolved using anhydrous DCE (0.05 M, 1.0 mL, commercially available, 99.8% from Sigma-Aldrich). The Schlenk tubes were then closed with the proper greased glass stoppers. The resulting solution was subjected to three cycles of freeze-pump-thaw to remove the oxygen and in the last cycle, the vessel was filled with argon. The reaction was irradiated under 456 nm blue LEDs for 20 hours with fan cooling. Afterwards, LED was removed and the solvent was evaporated under reduced pressure to afford the crude mixture. The following was further purified by silica-gel column chromatography to afford the corresponding *N*-phosphoryl azetidines. ^{1}H NMR yield of the product was determined using 1,3,5-trimethoxybenzene as internal standard. In cases where the ^{1}H NMR yield was difficult to determine accurately, triphenyl phosphite was used as an internal standard, and the yield was calculated by ^{31}P NMR spectroscopy.

(1-(diphenylphosphoryl)azetidin-3-yl)(phenyl)methanone (34)

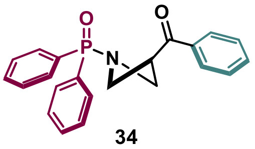

34

White solid; 15.5 mg, 0.050 mmol, 50% yield; Eluent: Hexane/EtOAc, 50/50 to 0/100, then Hexane/acetone 0/100; **^{1}H NMR (400 MHz, CDCl₃) *δ*:** 7.89 (dd, *J* = 11.0, 7.6 Hz, 4H), 7.79 (d, *J* = 7.4 Hz, 2H), 7.58 – 7.52 (m, 1H), 7.51 – 7.39 (m, 8H), 4.35 – 4.25 (m, 1H), 4.19 (app. q, *J* = 9.1 Hz, 2H), 4.02 (app. q, *J* = 7.2 Hz, 2H) ppm; **^{13}C NMR (101 MHz, $CDCl_3$) *δ*:** 197.5, 134.7, 133.8, 132.4 (d, *J* = 9.1 Hz), 132.1 (d, *J* = 2.9 Hz), 130.8 (d, *J* = 129.7 Hz), 129.0, 128.8 (d, *J* = 12.4 Hz), 128.4, 48.4 (d, *J* = 3.6 Hz), 38.0 (d, *J* = 14.5 Hz) ppm; **^{31}P NMR (162 MHz, $CDCl_3$) *δ*:** 25.8 ppm; **HRMS (ESI)**: m/z calcd. for $C_{22}H_{21}NO_2P$ $[M+H]^+$: 362.1310, found: 362.1299.

(4-(1H-naphtho[1,2-e][1,3]oxazin-2(3H)-yl)phenyl)(1-(bis(3,5 bis(trifluoromethyl)phenyl)phosphoryl)azetidin-3-yl)methanone (62)

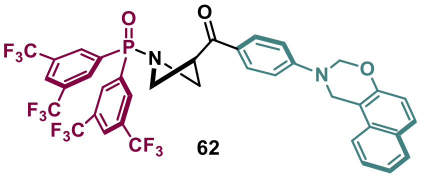

62

Yellow solid; 8.2 mg, 0.010 mmol, 10% yield; Eluent: Hexane/EtOAc, 50/50 to 0/100, then Hexane/acetone 0/100; **^{1}H NMR (600 MHz, $CDCl_3$) *δ*:** 8.47 (d, *J* = 10.2 Hz, 2H), 8.37 (d, *J* = 10.4 Hz, 2H), 8.08 (d, *J* = 14.0 Hz, 2H), 7.82 – 7.65 (m, 5H), 7.55 (t, *J* = 7.6 Hz, 1H), 7.41 (t, *J* = 7.5 Hz, 1H), 7.13 (d, *J* = 8.7 Hz, 2H), 7.06 (d, *J* = 8.9 Hz, 1H), 5.49 (s, 2H), 5.03 (s, 2H), 3.57 (t, *J* = 7.1 Hz, 1H), 3.53 – 3.40 (m, 4H) ppm; **^{31}P NMR (162 MHz, CDCl3) *δ*:** 25.7 ppm; **^{19}F NMR (377 MHz, $CDCl_3$) *δ*:** 53.4 ppm; **HRMS (ESI)**: m/z calcd. for $C_{38}H_{25}F_{12}N_2O_3P$ $[M+H]^+$: 817.1489, found: 817.1470.

(1-(bis(3,5-dimethylphenyl)phosphoryl)azetidin-3-yl)(phenyl)methanone (63)

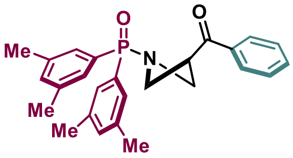

63

White solid; 17.0 mg, 0.041 mmol, 41% yield; Eluent: Hexane/EtOAc, 50/50 to 0/100, then Hexane/acetone 0/100; **^{1}H NMR (400 MHz, $CDCl_3$) *δ*:** 7.80 (d, *J* = 7.8 Hz, 2H), 7.56 (t, *J* = 7.3 Hz, 1H), 7.53 – 7.37 (m, 6H), 7.09 (s, 2H), 4.37 – 4.27 (m, 1H), 4.17 (q, *J* = 8.9 Hz, 2H), 4.04 (q, *J* = 7.8 Hz, 2H), 2.32 (s, 12H) ppm; **^{13}C NMR (101 MHz, $CDCl_3$) *δ*:** 197.8, 138.5 (d, *J* = 8.1 Hz), 133.8 (d, *J* = 15.2 Hz),133.7, 130.2, 129.9 (d,

J = 6.0 Hz), 129.0, 128.4, 53.6, 48.3, 38.1 (d, J = 9.1 Hz), 21.4 ppm; **^{31}P NMR (162 MHz, CDCl3) δ:** 27.3 ppm; **HRMS (ESI)**: m/z calcd. for $C_{26}H_{29}N_2O_2P$ $[M+H]^+$: 418.1930, found: 418.1921.

(4-(1H-naphtho[1,2-e][1,3]oxazin-2(3H)-yl)phenyl)(1-(2-oxido-1,3,2-dioxaphospholan-2-yl)azetidin-3-yl)methanone (67)

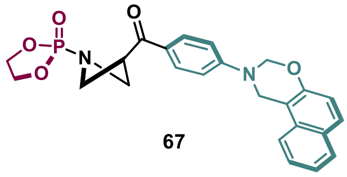

67

Yellow solid; 17.3 mg, 0.038 mmol, 38% yield; Eluent: Hexane/EtOAc, 50/50 to 0/100, then Hexane/acetone 0/100; **^{1}H NMR (400 MHz, $CDCl_3$) δ:** 7.78 (d, J = 8.3 Hz, 1H), 7.73 – 7.64 (m, 2H), 7.59 – 7.51 (m, 2H), 7.43 – 7.29 (m, 3H), 7.13 – 7.03 (m, 2H), 5.46 (s, 2H), 5.00 (s, 2H), 4.18 (app. q, J = 8.1 Hz, 2H), 3.77 – 3.68 (m, 3H), 3.66 – 3.58 (m, 2H), 3.31 (app. q, J = 8.5 Hz, 2H) ppm; **^{13}C NMR (150 MHz, $CDCl_3$) δ:** 196.6, 152.2, 135.9, 131.1, 131.0, 130.5, 128.9, 128.8, 128.2, 128.0, 127.1, 124.1, 120.9, 118.8, 116.1, 112.3, 66.6 (d, J = 2.1 Hz), 51.7 (d, J = 8.5 Hz), 47.7, 37.5 ppm; **^{31}P NMR (162 MHz, $CDCl_3$) δ:** 40.4 ppm; **HRMS (ESI)**: m/z calcd. for $C_{24}H_{24}N_2O_5P$ $[M+H]^+$: 451.1423, found: 451.1399.

3.6. Reaction performed at 1.0 mmol scale

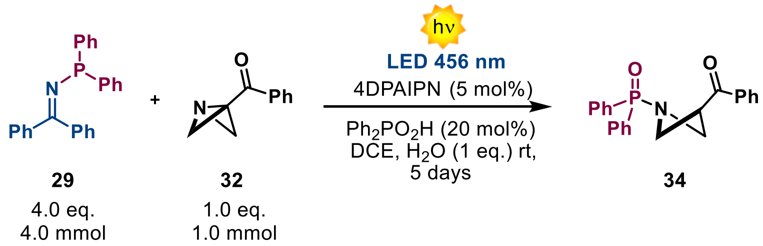

Procedure: In an oven-dried 50.0 mL Schlenk tube containing an oven dried Teflon coated stirring bar was added 4DPAIPN (40.0 mg, 5.0 mol%), diphenylphosphinic acid **38** (44.0 mg, 20.0 mol%), iminyl phosphine **29** (1.46 g, 4.0 mmol, 4.0 eq.), azabicyclo[1.1.0]butane **32** (159.2 mg, 1.0 mmol, 1.0 eq.) and were dissolved using the anhydrous DCE (0.05 M, 20.0 mL, commercially available, 99.8% from Sigma-Aldrich). The Schlenk was then closed with the proper greased glass stoppers. The resulting solution was subjected to three cycles of freeze-pump-thaw to remove the oxygen and in the last cycle, the vessel was filled with argon. The reaction was irradiated under 456 nm blue LEDs (only one LED light source) for 5 days with fan cooling. Afterwards, LED was removed and the solvent was evaporated under reduced pressure to afford the crude mixture. The following was further purified by silica-gel column chromatography (Hexane/Ethyl acetate, 50/50 to 0/100, then Hexane/acetone 0/100) to afford the *N*-phosphoryl azetidines **34** in 50% (180.7 mg, 0.5 mmol) isolated yield.

3.6. Product Manipulation

3.6.1. Synthesis of (3-(hydroxy(phenyl)methyl)azetidin-1-yl)diphenylphosphine oxide (70)

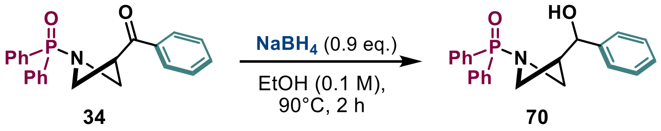

In a round bottom flask were placed the N-phosphoryl azetidine **34** (18.0 mg, 0.05 mmol, 1 eq.). The flask was evacuated and backfilled with argon (3 times) followed by the addition of absolute ethanol (0.5 mL, 0.1 M) and the sodium borohydride (1.7 mg, 0.045 mmol, 0.9 eq.). The mixture was stirred at 90 °C for 2 hours, after cooling to room temperature, the reaction was quenched with water, the volatiles were removed under reduced pressure, and the aqueous phase was extracted with DCM (3 times). The organic phase was dried with Na_2SO_4, filtrated, and evaporated under reduced pressure. The crude mixture was further purified by silica-gel column chromatography to afford the corresponding compound **70**. ^{1}H NMR yield of the product was determined using 1,3,5-trimethoxybenzene

as internal standard. We faced difficulty in purifying product **70** and the isolated product still contains some impurity, so we only considered the ^{1}H NMR yield in the manuscript.

White powder; 14.6 mg, 0.041 mmol, 82% yield; Eluent: Hexane/acetone, 90/10 to 50/50; **^{1}H NMR (600 MHz, $CDCl_3$) *δ*:** 7.84 (dd, *J* = 11.9, 7.3 Hz, 4H), 7.50 – 7.38 (m, 6H), 7.36 – 7.28 (m, 4H), 7.16 – 7.10 (m, 1H), 4.94 (d, *J* = 7.8 Hz, 1H), 3.95 – 3.87 (m, 1H), 3.84 (app. q, *J* = 8.3 Hz, 1H), 3.70 – 3.59 (m, 2H), 3.06 (h, *J* = 7.1 Hz, 1H), 2.91 (br. s, 1H) ppm; **^{13}C NMR (150 MHz, $CDCl_3$) *δ*:** 142.2, 132.6 – 132.2 (m), 132.0, 128.8 – 128.6 (m), 128.5, 127.8 (d, *J* = 90.3 Hz), 127.2, 126.4, 75.8, 49.3 (d, *J* = 4.5 Hz), 48.5 (d, *J* = 4.5 Hz), 37.9 (d, *J* = 14.0 Hz) ppm; **^{31}P NMR (162 MHz, $CDCl_3$) *δ*:** 26.5 ppm; **HRMS (ESI)**: m/z calcd. for $C_{22}H_{23}NO_2P$ $[M+H]^+$: 364.1466, found: 364.1463.

3.6.2. Synthesis of N-thiophosphoryl azetidines

Synthesis of $PhSiH_2$-O-SiH_2Ph (DPDS)

The compound $PhSiH_2$-O-SiH_2Ph (DPDS) was synthesized following a reported procedure.[15] A 200.0 mL round-bottom flask equipped with a magnetic stir bar was charged with $CuCl_2$ (5.4 g, 40.0 mmol) and CuI (381.0 mg, 2.0 mmol) in Et_2O (27.0 mL, 1.7 M) under an argon atmosphere. The resulting suspension was stirred at room temperature (750 rpm) for 30 minutes, after which phenylsilane (5.54 mL, 45 mmol, 1.0 eq.) was added dropwise. The reaction mixture was stirred for an additional 20 hours at room temperature. Upon completion, the mixture was filtered directly onto crushed ice and washed with Et_2O. The combined ether and melted aqueous layer was gently swirled every 5 min for 15 min until the ice fully melted. The organic phase was separated, washed with cold deionized water, dried over anhydrous Na_2SO_4, and concentrated under reduced pressure. The desired product, $PhSiH_2$-O-SiH_2Ph, was obtained as a pale-yellow oil (76%, 1.76 g, 7.62 mmol).

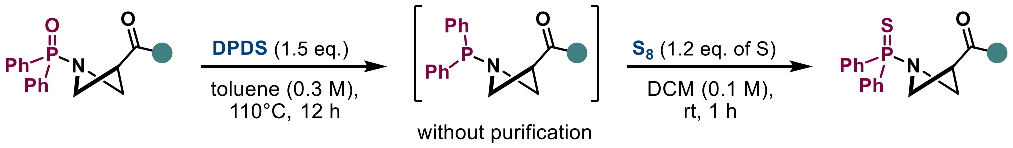

General procedure. In a Teflon-cock sealed tube were placed 1,3-diphenyl-disiloxane (DPDS, 1.5 eq.) and N-phosphoryl azetidine (0.1 mmol, 1.0 eq.). The sealed tube was evacuated and backfilled with N_2 (3 times) followed by the addition of toluene (0.33 mL, 0.3 M). The mixture was stirred at 110 °C for 12 hours, and then, after cooling to room temperature, the volatiles were removed under reduced pressure. Afterwards, sulfur (3.8 mg, 1.2 mmol, 1.2 eq.) was added and dissolved using DCM (1.0 mL, 0.1 M) under N_2 atmosphere and stirred for another 1 hour at room temperature. Then, the solvent was evaporated under reduced pressure to afford the crude mixture. The crude mixture was further purified by silica-gel column chromatography (eluent: Hexane/Ethyl acetate, 100/0 to 80/20) to afford the corresponding N-thiophosphoryl azetidine. ^{1}H NMR yield of the product was determined using 1,3,5-trimethoxybenzene as internal standard.

(1-(diphenylphosphorothioyl)azetidin-3-yl)(phenyl)methanone (72)

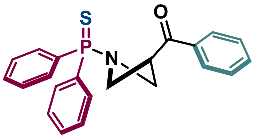

72

White solid; 30.5 mg, 0.081 mmol, 81% yield; Eluent: Hexane/EtOAc, 100/0 to 80/20; **^{1}H NMR (400 MHz, $CDCl_3$) *δ*:** 8.02 (dd, *J* = 13.1, 8.0 Hz, 4H), 7.81 (d, *J* = 8.3 Hz, 2H), 7.56 (t, *J* = 7.4 Hz, 1H), 7.50 – 7.38 (m, 8H), 4.26 – 4.17 (m, 1H), 4.12 – 4.00 (m, 2H), 3.94 – 3.83 (m, 2H) ppm; **^{13}C NMR (101 MHz, $CDCl_3$) *δ*:** 197.6, 134.8, 133.8, 132.1, 132.0 (d, *J* = 103.5 Hz), 132.0 (d, *J* = 3.3 Hz), 132.0, 128.9 (d, *J* = 27.2 Hz), 128.6 (d, *J* = 14.2 Hz), 48.3 (d, *J* = 4.0 Hz), 37.9 (d, *J* = 16.0 Hz) ppm; **^{31}P NMR (162 MHz, $CDCl_3$) *δ*:** 67.7 ppm; **HRMS (ESI)**: m/z calcd. for $C_{22}H_{21}NOPS$ $[M+H]^+$: 378.1081, found: 378.1063.

(1-(diphenylphosphorothioyl)azetidin-3-yl)(naphthalen-2-yl)methanone (73)

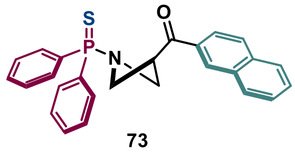

73

White solid; 33.9 mg, 0.079 mmol, 79% yield; Eluent: Hexane/EtOAc, 100/0 to 80/20; **^{1}H NMR (400 MHz, $CDCl_3$) *δ*:** 8.24 (s, 1H), 8.03 (dd, *J* = 13.1, 7.8 Hz, 4H), 7.97 – 7.84 (m, 4H), 7.63 – 7.52 (m, 2H), 7.49 – 7.39 (m, 6H), 4.42 – 4.32 (m, 1H), 4.20 – 4.09 (m, 2H), 4.00 – 3.91 (m, 2H) ppm; **^{13}C NMR (101 MHz, $CDCl_3$) *δ*:** ^{13}C NMR (101 MHz, $CDCl_3$) δ 197.6, 134.8, 133.8, 132.0 (d, *J* = 103.5 Hz), 132.0 (d, *J* = 10.9 Hz), 132.0 (d, *J* = 3.3 Hz), 129.0, 128.7, 128.6 (d, *J* = 14.2 Hz), 48.3 (d, *J* = 4.0 Hz), 37.9 (d, *J* = 16.0 Hz) ppm; **^{31}P NMR (162 MHz, $CDCl_3$) *δ*:** 67.8 ppm; **HRMS (ESI)**: m/z calcd. for $C_{26}H_{23}NOPS$ $[M+H]^+$: 428.1238, found: 428.1220.

4. Mechanistic investigations

4.1. Electrochemical studies and evaluation of the SET process

To evaluate the feasibility of a single-electron transfer (SET) event between the excited photocatalyst (4DPAIPN*) and the reaction partners, we first examined the redox properties of iminyl phosphine **29** and azabicyclo[1.1.0]butane **32** by cyclic voltammetry (CV) studies. Measurements were carried out in dry 1,2-dichloroethane (DCE) containing 0.1 M $TBAPF_6$ as the supporting electrolyte, using a glassy carbon working electrode, platinum counter electrode, and a saturated calomel electrode (SCE) as the reference. The potentials were internally calibrated against the ferrocene/ferrocenium (Fc/Fc^+) couple.

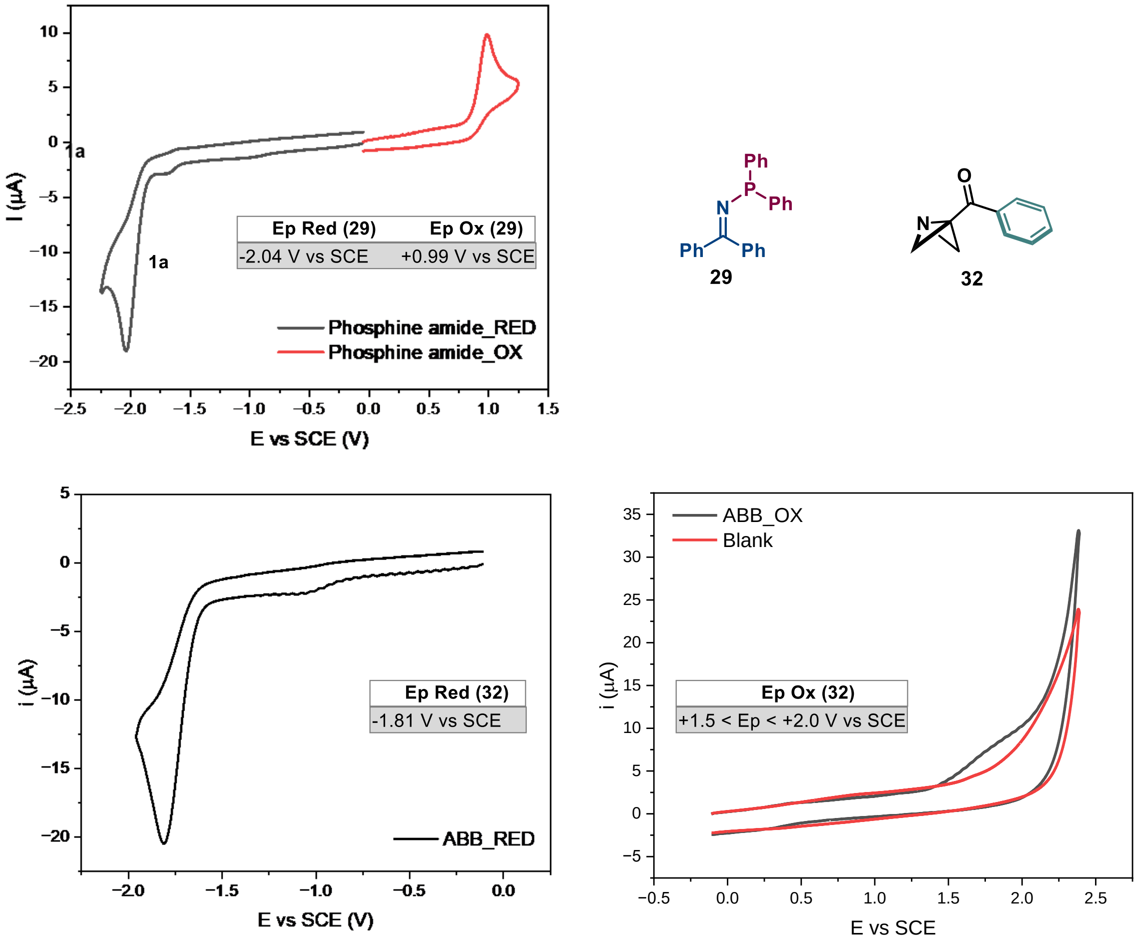

Figure S3: Cyclic voltammetry (CV) of **29** and **32** in 0.1 M $TBAPF_6$, using a glassy carbon disk working electrode, platinum counter electrode and SCE reference electrode at 0.1 V/s scan rate.

Cyclic voltammetry measurements revealed that iminyl phosphine **29** exhibits an irreversible reduction peak at −2.04 V vs SCE and an oxidation peak at +0.99 V vs SCE in DCE. Similarly, azabicyclo[1.1.0]butane **32** displays an irreversible reduction at −1.81 V vs SCE and an oxidation potential in the range of +1.5 to +2.0 V vs SCE. These electrochemical data are consistent with the hypothesis that the excited photocatalyst 4DPAIPN* ($E_{1/2}$ (PC^*/PC^-) = +1.1 V vs SCE) can engage in single-electron transfer (SET) with iminyl phosphine **29** via a reductive quenching cycle. The redox potential of **32** indicates that it cannot participate in SET with the excited-state photocatalyst, thereby ruling out initiation via SET from **32**. Furthermore, the observed reactivity with the formation of N-phosphoryl azetidine **34** when employing photocatalysts of varying redox potentials such as 4CzIPN (Table S3, entry 6), TXO (Table S3, entry 1), and the highly oxidizing Mes-$Acr^+BF_4^-$ (Table S3, entry 11) supports a mechanistic scenario in which the reaction proceeds predominantly via SET oxidation of **29**.

4.2. Stern- Volmer luminescence quenching studies

The interaction between the excited photocatalyst (4DPAIPN*) and the reaction partners (**29** and **32**) was further investigated by steady-state Stern-Volmer fluorescence quenching experiments. For this purpose, the fluorescence of the excited photocatalyst (4DPAIPN*) was measured in the presence of each quencher at varying concentrations. Emission spectra of 4DPAIPN (2.5×10^{-4} M) in 1,2-dichloroethane (DCE) were recorded upon incremental addition of either iminyl phosphine **29** (2.5×10^{-3} M) or azabicyclo[1.1.0]butane **32** (2.5×10^{-3} M). A pronounced decrease in fluorescence intensity was observed upon addition of **29** (Figure S4), whereas no measurable quenching occurred in the presence of **32**. These results clearly indicate that **29** act as the sole quencher of the excited photocatalyst, consistent with its ability to undergo single-electron oxidation by 4DPAIPN*.

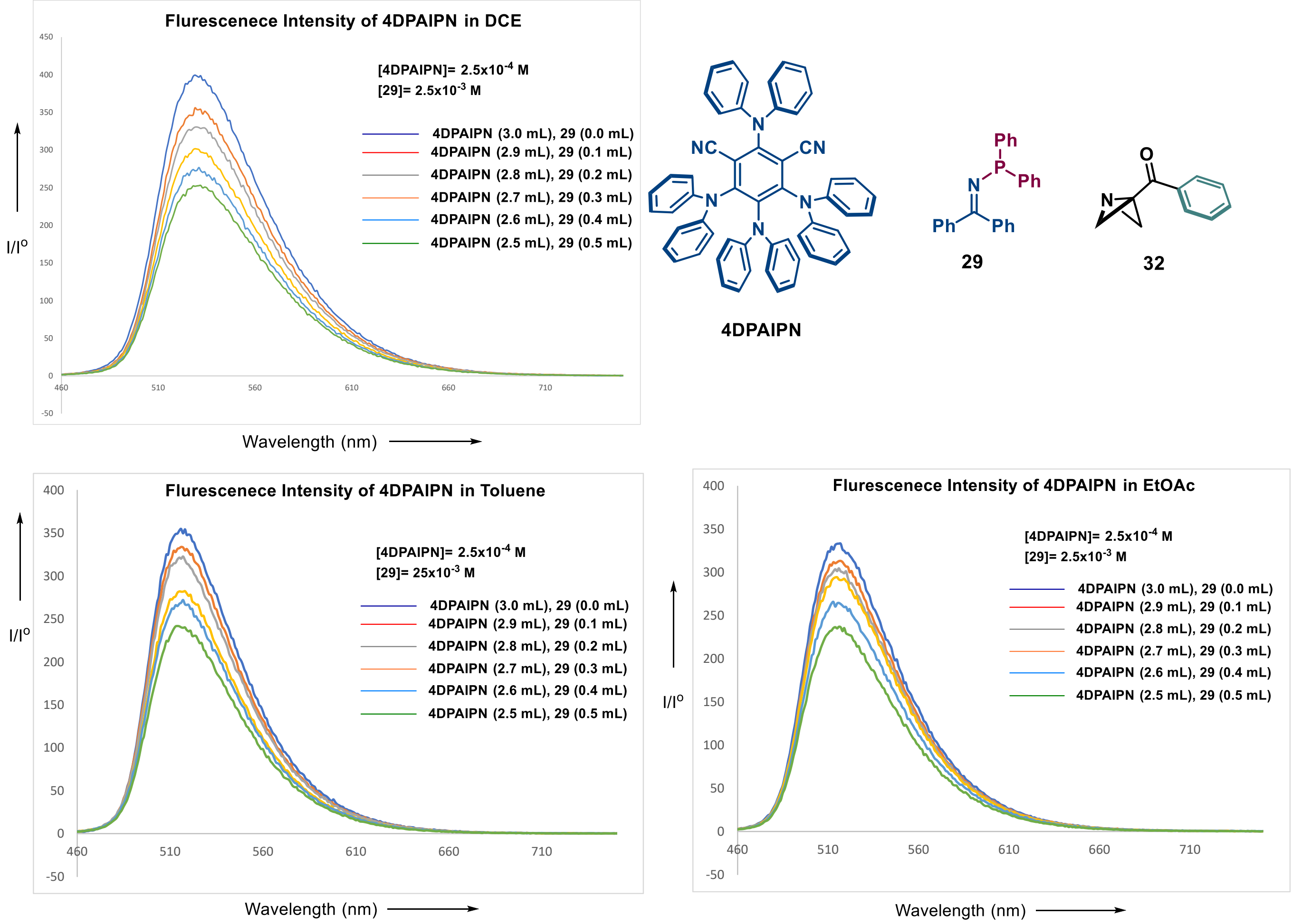

Figure S4: Stern-Volmer fluorescence quenching experiments.

To further probe the quenching pathway, similar experiments were performed in solvents of varying polarity, including Ethyl acetate and toluene (Figure S4 and S5). The observed variation in Stern-Volmer quenching constants (K_{sv}) across solvents supports an electron-transfer-driven mechanism rather than energy transfer (EnT), as the efficiency of SET processes is highly dependent on solvent polarity and dielectric constant.[16]

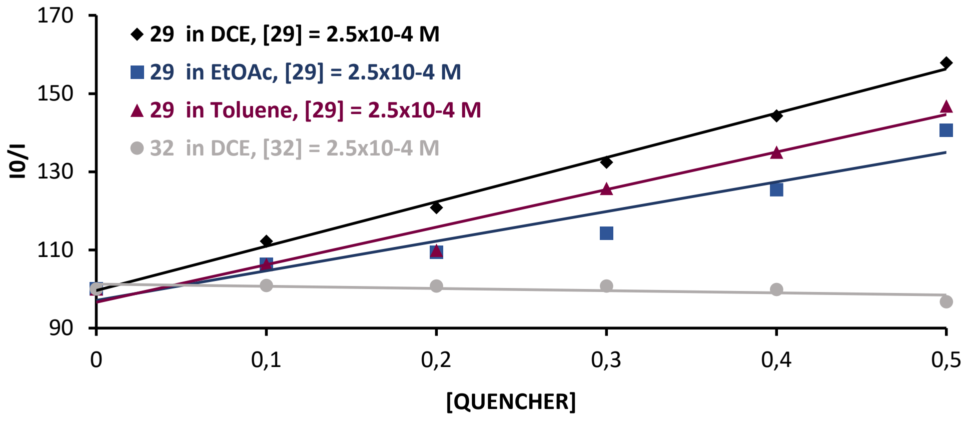

Figure S5: Variation in Stern-Volmer quenching constants (K_{sv}) across different solvents.

4.3. UV–visible absorption studies

UV–visible absorption spectra of iminyl phosphines **29**, **30**, and **31** were recorded in DCE to evaluate their light absorption characteristics relevant to the photocatalytic process. As shown in Figure S6, recorded UV–visible absorption spectra of **29**, **30** and **31** indicates that the only absorbing species is **29** in the range above 427 nm as no absorption of **30** and **31** was observed in these range.

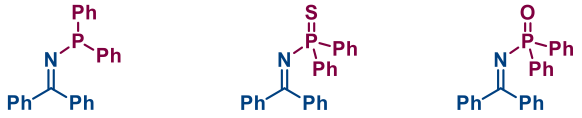

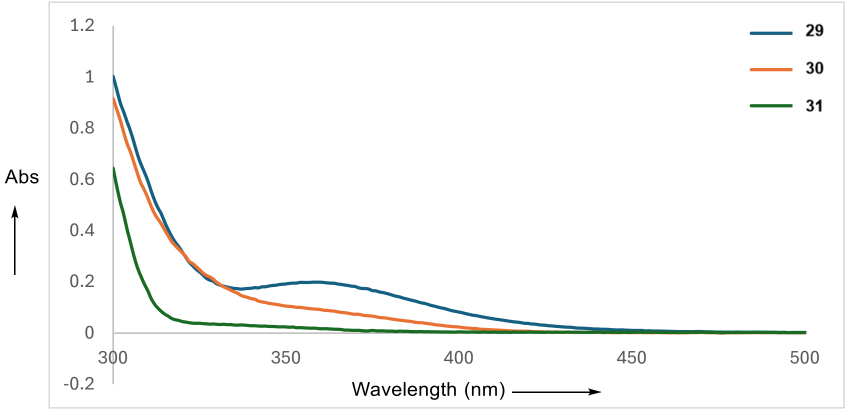

Figure S6: Optical absorption spectra, recorded in DCE in 1 cm path quartz cuvettes using Varian Cary 50 UV-vis spectrophotometer, [**29**] = 10^{-4} M, [**30**] = 10^{-4} M, [**31**] = 10^{-4} M.

4.4. Direct excitation studies

To evaluate the possibility of direct photolysis, control experiments were conducted in which **29** was irradiated under different wavelength of light with standard reaction conditions in the absence of 4DPAIPN (Table S8). No formation of N-phosphoryl azetidine **34** was observed. Furthermore, the absence of productive reactivity is supporting the fact that the reaction proceeds via single-electron transfer (SET) pathway rather than an energy transfer (EnT) mechanism.

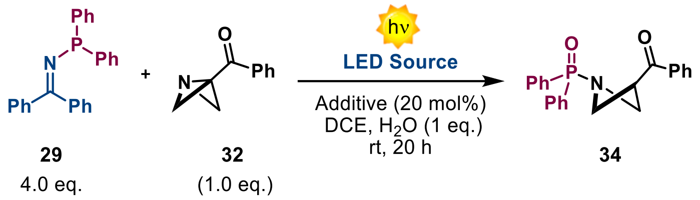

Entry[a]	Light source	Additive (20.0 mol%)	Yield 34 (%)[b]
1	400 nm	-	n.d.
2	427 nm	-	n.d.
3	456 nm	-	n.d.
4	456 nm	Ph_2PO_2H (**38**)	n.d.
5	370 nm	-	n.d.

Table S8: [a]Reaction performed at 0.1 mmol scale, **29** (4.0 eq.), **32** (1.0 eq.). [b]^{1}H NMR yield using 1,3,5-trimethoxybenzene as internal standard. [c]Isolated yield.

Procedure: In an oven-dried 10 mL Schlenk tube containing an oven dried Teflon coated stirring bar was added iminyl phosphine **29** (146.2 mg, 0.4 mmol, 4.0 eq.), azabicyclo[1.1.0]butane **32** (15.9 mg, 0.1 mmol, 1.0 eq.) and were dissolved using anhydrous DCE (0.05 M, 2.0 mL, commercially available, 99.8% from Sigma-Aldrich).The Schlenk tubes were then closed with the proper greased glass stoppers. The resulting solution was subjected to three cycles of free-pump-thaw to remove the oxygen and in the last cycle, the vessel was filled with argon. The reaction was irradiated with LEDs for 20 hours with fan cooling. Afterwards, LED was removed and the solvent was evaporated under reduced pressure to afford the crude mixture. The crude mixture was then analyzed using UPC^2 (ultraperformance convergence chromatography) and NMR spectroscopy.

4.5. TEMPO trapping studies

To validate the involvement of radical intermediates in the photocatalytic transformation, a trapping experiment was performed using 2,2,6,6-tetramethylpiperidine-1-oxyl (TEMPO, 3.0 eq.).

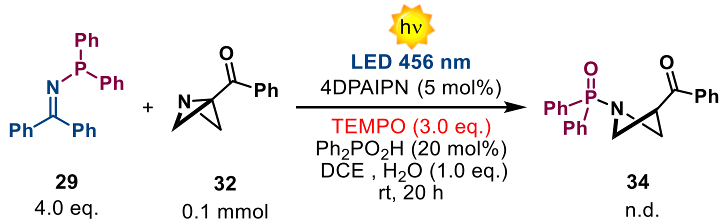

Procedure: In an oven-dried 10 mL Schlenk tube containing an oven dried Teflon coated stirring bar was added 4DPAIPN (4.0 mg, 5.0 mol%), diphenylphosphinic acid (4.4 mg, 20.0 mol%), iminyl phosphine **29** (146.2 mg, 0.4 mmol, 4.0 eq.), TEMPO (46.9 mg, 0.3 mmol, 3.0 eq.), azabicyclo[1.1.0]butane **32** (15.9 mg, 0.1 mmol, 1.0 eq.) and were dissolved using the anhydrous DCE (0.05 M, 2.0 mL, commercially available, 99.8% from Sigma-Aldrich).The Schlenk tubes were then closed with the proper greased glass stoppers. The resulting solution was subjected to three cycles of free-pump-thaw to remove the oxygen and in the last cycle, the vessel was filled with argon. The reaction was irradiated under 456 nm blue LEDs for 20 hours with fan cooling. Afterwards, LED was removed and the solvent was evaporated under reduced pressure to afford the crude mixture. The crude mixture was analyzed using UPC^2 (ultraperformance convergence chromatography) and NMR spectroscopy.

The addition of TEMPO completely inhibited the formation of the N-phosphoryl azetidine **34**, confirming that the reaction proceeds through a radical-mediated pathway. Despite complete suppression of product formation, no TEMPO-adducts derived from the putative radical intermediates could be detected by UPC^2 (ultraperformance convergence chromatography) analysis.

4.6. EPR Spin trapping studies

To gain further mechanistic insight, we conducted electron paramagnetic resonance (EPR) studies utilizing the spin-trapping agent α-phenyl-tert-butylnitrone (PBN).

A solution of a spin trap (PBN, N-tert-Butyl-α-phenylnitrone) was incubated with the reaction mixture (the photocatalyst **4DPAIPN**, **29**, **32**, and **38**). The solution was thoroughly degassed and analysed before and after 20 hours of irradiation using blue LED light (456 nm). The solution showed a modest presence of trapped radicals immediately after preparation, the amount of which increased more than ten times with irradiation. The EPR spectrum obtained after irradiation (Figure S7 – shown with the global simulation in red) shows the presence of two radicals. The dominant species (75%) can be attributed to the trapping of the phosphoryl radical **24** based on the simulation (Figure S7– green simulation), performed with EasySpin[17]. The hyperfine couplings and g_{iso} (a_{14N} = 14.5 G; a_{1H} = 3.6 G; a_{31P} = 16.5 G; g_{iso} = 2.0059) are comparable to what is reported in the literature (a_{14N} = 14.0-14.5 G; a_{1H} = 2.9-4.3 G; a_{31P} = 14.9-19.0 G)[18-21]. The phosphoryl radical **24** is trapped also when either azabicyclo[1.1.0]butanes **32** or **38** are not present in solution (Figure S8). However, in the second case it is much less abundant, suggesting a key kinetic role of **38** in favouring the generation of the phosphoryl radical **24**. When **38** is not present, the dominant species is the second trapped radical, its line shape being a triplet of doublets with some unresolved hyperfine splitting (evidenced by the blue simulation). The hyperfine couplings and slightly different g-factor (a_{14N} = 14.7 G; a_{1H} = 2.6 G; g_{iso} = 2.0060) do not support it being a P-centred radical. Since no radicals are trapped by PBN in the presence of **4DPAIPN** only, it is not a radical coming from the solvent. Based on the magnetic parameters, we can only suggest that this radical possibly comes from a rearrangement of one of the different components of the solution.

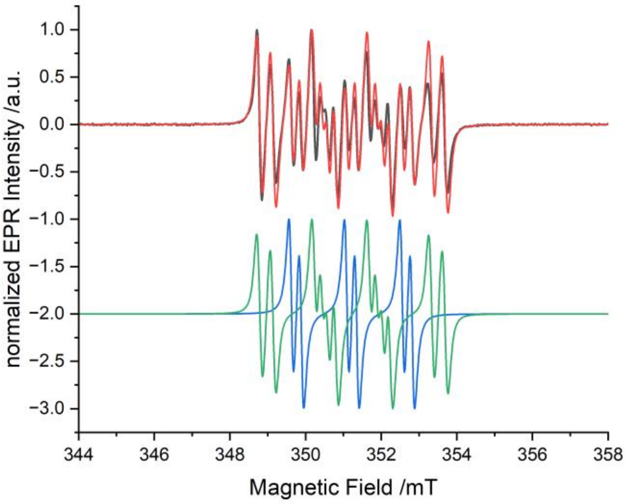

Figure S7: EPR spectrum (black) of the radicals trapped by PBN in the full reaction mixture after 20 h LED irradiation at 456 nm. The global simulation (red) was a weighted sum of the two simulations in green (phosphoryl radical **24**) and blue (unidentified radical, likely C-centered).

EPR Experimental:

All solutions for spin trapping experiments were prepared in Schlenk flasks to keep the solutions thoroughly degassed. Each flask contained 50 mM of PBN in 5.0 mL dichloroethane in addition to the other components. We prepared the following reaction mixtures as mentioned in the main text:

1) 4DPAIPN, **29**, **32**, **38**

2) 4DPAIPN, **29**, **38**

3) 4DPAIPN, **29**, **32**

4) 4DPAIPN alone.

In Figure S8, we report the spectra of the radicals trapped in these solutions, as stated above. For each set of experiments the solutions were kept in the dark or irradiated for 20 hours using blue LED light at 456 nm with the same setup used for the other reactions described in the main text. Spin trapping experiments with PBN were performed on 40.0 mL of solution taken using a gas purged syringe and quickly loaded into glass capillaries and sealed with wax. The EPR spectra of the trapped radicals were recorded on an X-band Elexsys E580 spectrometer (Bruker BioSpin GmbH, Rheinstetten, Germany) using a SHQ cavity (n_{mw} = 9.8634 GHz). All the EPR spectra were obtained using the following parameters: microwave power 5 mW (attenuation 15 dB), modulation amplitude 0.05 mT, modulation frequency 100 kHz, time constant 20 ms, conversion time 82 ms, scan width 15 mT, 1024 points, 4 scans, room temperature. The magnetic field axis was calibrated measuring a diphenylpicrylhydrazyl crystal (DPPH, g = 2.0036). Spectral simulations were performed using Easyspin version 6.0.11 using the *garlic* function for CW-EPR spectra.[17]

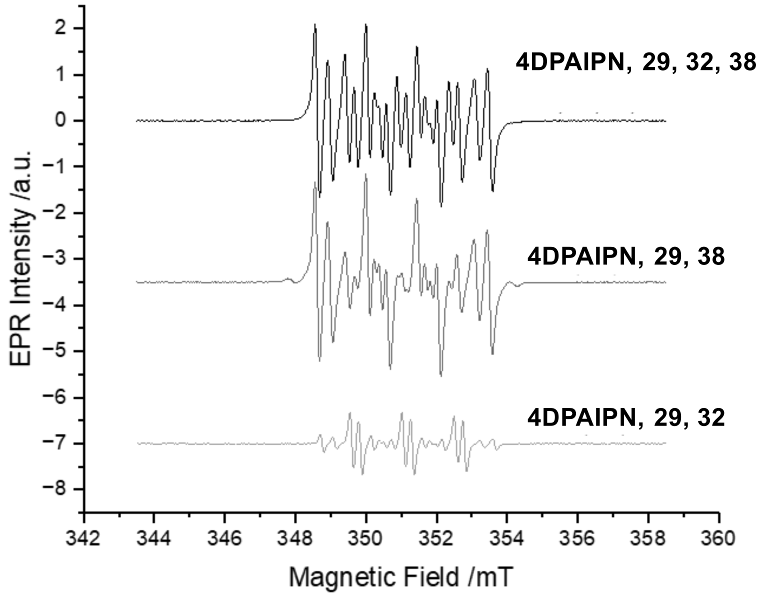

Figure S8: EPR spectra of the radicals trapped by PBN after 20 h LED irradiation at 456 nm. The solution composition is shown to the right of each spectrum.

4.7. Control experiments

Following confirmation of the formation of the key phosphoryl radical intermediate ($Ph_2P(=O)\cdot$) **24** in the reaction medium by EPR experiments, we sought to establish that this species originates specifically from iminyl phosphine **29** upon single-electron transfer (SET) oxidation to **29**$^{\bullet+}$. To exclude alternative sources, a series of control experiments (section 7.7.1–7.7.4) was performed in which iminyl phosphine **29** was replaced with phosphine-derived side products detected in the reaction media, namely diphenyl phosphine oxide ($Ph_2P(=O)H$, **36**), diphenylphosphine amide ($Ph_2P(=O)NH_2$, **37**) and diphenylphosphinic acid (Ph_2PO_2H, **38**).

4.7.1. Verifying the feasibility from diphenylphosphineoxide (DPPO, $Ph_2P(O)H$) 36.

During UPC^2 analysis of the crude reaction mixtures, diphenylphosphine oxide (DPPO, **36**) was identified and isolated as a byproduct of the photochemical transformation. **36** is known to undergo SET oxidation under photoredox conditions to generate the corresponding phosphoryl radical.[22] This prompted us to examine whether **36** could act as a viable precursor to the phosphoryl radical ($Ph_2P(=O)\cdot$) under photocatalytic conditions. Accordingly, iminyl phosphine **29** was replaced with **36** to evaluate the feasibility of phosphoryl radical **24** generation via a reductive-quenching pathway mediated by 4DPAIPN as shown in Figure S9.

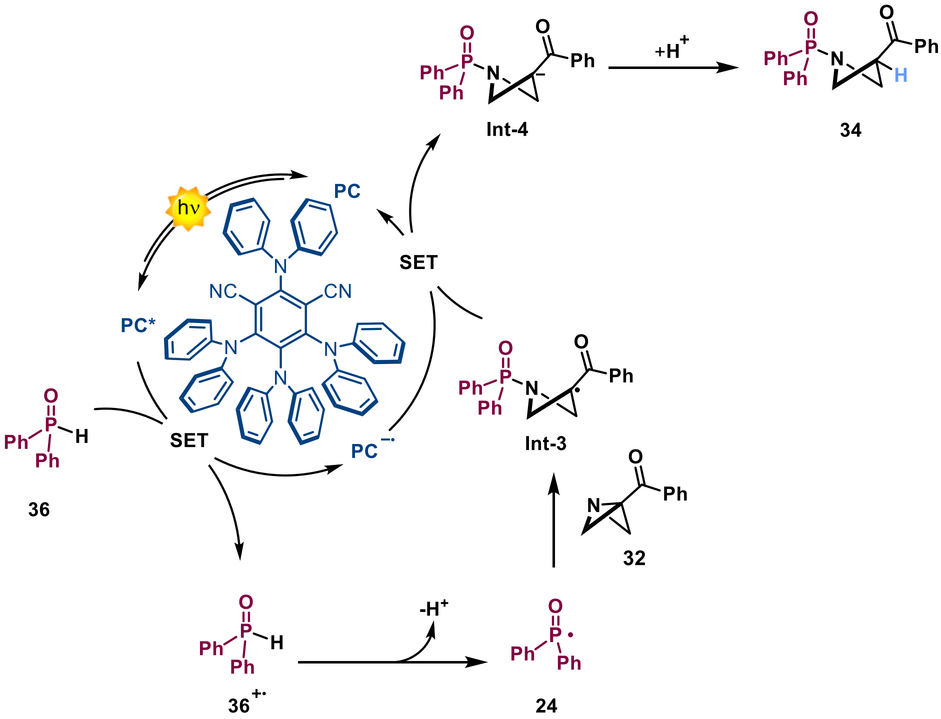

Figure S9: Verification of possible phosphoryl radical **24** generation from **36** via reductive quenching manifold.

As shown in table S9, control reactions conducted in the presence of other photocatalyst or in the dark failed to yield any detectable product. These results indicate that DPPO **36** does not participate in the formation of the active phosphoryl radical $Ph_2P(=O)\cdot$) **24** under the applied photocatalytic conditions, thereby excluding it as a direct precursor for the phosphoryl radical $Ph_2P(=O)\cdot$) **24** under the standard photochemical conditions.

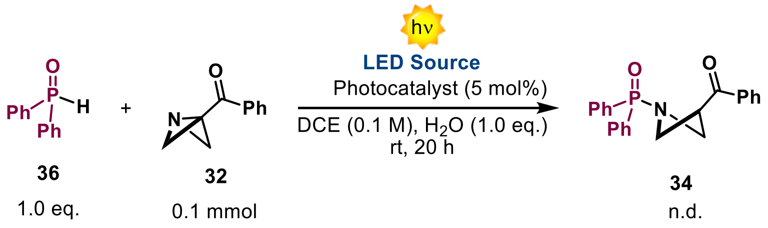

Entry[a]	Photocatalyst (5.0 mol%)	Light source	Yield 34 (%)[b]
1	4DPAIPN	456 nm	n.d.
2	TXO	400 nm	n.d.
3	-	456 nm	n.d.
4	-	-	n.d.

Table S9: [a]Reaction performed at 0.1 mmol scale, **36** (1.0 eq.), **32** (1.0 eq.), photocatalyst (5.0 mol%). [b]1H NMR yield using 1,3,5-trimethoxybenzene as internal standard.

Procedure: In an oven-dried 10.0 mL Schlenk tube containing an oven dried Teflon coated stirring bar was added with photocatalyst (5.0 mol%), diphenylphosphine oxide **36** (20.2 mg, 0.1 mmol, 1.0 eq.), azabicyclo[1.1.0]butane **32** (15.9 mg, 0.1 mmol, 1.0 eq.) and were dissolved using the anhydrous DCE (0.1 M, 1.0 mL, commercially available, 99.8% from Sigma-Aldrich).The Schlenk tubes were then closed with the proper greased glass stoppers. The resulting solution was subjected to three cycles of free-pump-thaw to remove the oxygen and in the last cycle, the vessel was filled with argon. The reaction was irradiated under blue LEDs for 20 hours with fan cooling. Afterwards, LED was removed and the solvent was evaporated under reduced pressure to afford the crude mixture and was analyzed by ^{1}H NMR spectroscopy using 1,3,5-trimethoxybenzene as an internal standard. No formation of the desired N-phosphoryl azetidine **34** was observed under these conditions.

4.7.2. Verifying the feasibility from diphenylphosphinic amide ($Ph_2P(O)NH_2$) 37.

During UPC² analysis of the crude reaction mixtures, diphenylphosphinic amide **37** was identified and isolated as a byproduct of the photochemical transformation. This observation prompted us to investigate the potential for direct generation of the phosphoryl radical **24** under the standard photocatalytic conditions. To probe this possibility, iminyl phosphine **29** was replaced with diphenylphosphinic amide **37**, and the reaction was conducted under identical conditions.

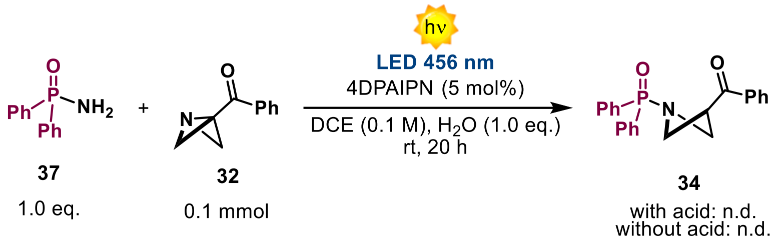

Procedure: In an oven-dried 10.0 mL Schlenk tube containing an oven dried Teflon coated stirring bar was added 4DPAIPN (4.0 mg, 5.0 mol%), diphenylphosphinic amide **37** (21.7 mg, 0.1 mmol, 1.0 eq.), azabicyclo[1.1.0]butane **32** (15.9 mg, 0.1 mmol, 1.0 eq.) and were dissolved using the anhydrous DCE (0.1 M, 1.0 mL, commercially available, 99.8% from Sigma-Aldrich).The Schlenk tubes were then closed with the proper greased glass stoppers. The resulting solution was subjected to three cycles of free-pump-thaw to remove the oxygen and in the last cycle, the vessel was filled with argon. The reaction was irradiated under 456 nm blue LEDs for 20 hours with fan cooling. Afterwards, LED was removed and the solvent was evaporated under reduced pressure to afford the crude mixture and was analyzed by ^{1}H and ^{31}P NMR spectroscopy using 1,3,5-trimethoxybenzene and triphenyl phosphite as an internal standard. No formation of the desired N-phosphoryl azetidine **34** was observed under these conditions.

4.7.3. Verifying the feasibility from diphenylphosphinic acid (Ph_2PO_2H) 38.

During UPC² analysis of the crude reaction mixtures, diphenylphosphinic acid (**38**) was detected as a byproduct of the photochemical transformation. Furthermore, the catalytic addition (20.0 mol%) of diphenylphosphinic acid **38** seems to enhance the generation of the desired product **34**. This observation prompted us to investigate the potential for direct generation of the phosphoryl radical **24** under the standard photocatalytic conditions. To probe this

possibility, iminyl phosphine **29** was replaced with diphenylphosphinic acid **38**, and the reaction was conducted under identical conditions.

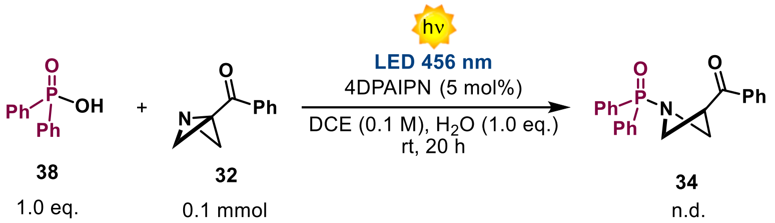

Procedure: In an oven-dried 10.0 mL Schlenk tube containing an oven dried Teflon coated stirring bar was added 4DPAIPN (4.0 mg, 5.0 mol%), diphenylphosphinic acid **38** (21.8 mg, 0.1 mmol, 1.0 eq.), azabicyclo[1.1.0]butane **32** (15.9 mg, 0.1 mmol, 1.0 eq.) and were dissolved using the anhydrous DCE (0.1 M, 1.0 mL, commercially available, 99.8% from Sigma-Aldrich).The Schlenk tubes were then closed with the proper greased glass stoppers. The resulting solution was subjected to three cycles of free-pump-thaw to remove the oxygen and in the last cycle, the vessel was filled with argon. The reaction was irradiated under 456 nm blue LEDs for 20 hours with fan cooling. Afterwards, LED was removed and the solvent was evaporated under reduced pressure to afford the crude mixture and was analyzed by ^{1}H and ^{31}P NMR spectroscopy using 1,3,5-trimethoxybenzene and triphenyl phosphite as an internal standard. No formation of the desired N-phosphoryl azetidine **34** was observed under these conditions.

4.7.4. Verifying the feasibility of generation of 24 from mixture of all reaction byproducts.

During UPC^2 analysis of the crude reaction mixtures, benzophenone **35**, diphenylphosphine oxide **36**, diphenylphosphinic amide **37**, diphenylphosphinic acid (**38**) was detected as a byproduct of the photochemical transformation. This observation prompted us to investigate the potential generation of the phosphoryl radical **24** from the mixture of rection byproducts under the standard photocatalytic conditions. To probe this possibility, iminyl phosphine **29** was replaced with a mixture of **35-38**, and the reaction was conducted under identical conditions. However, no formation of the desired N-phosphoryl azetidine **34** was observed under these conditions.

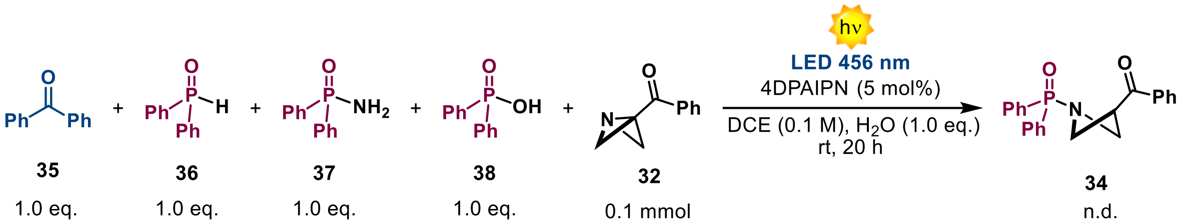

Procedure: In an oven-dried 10.0 mL Schlenk tube containing an oven dried Teflon coated stirring bar was added 4DPAIPN (4.0 mg, 5.0 mol%), benzophenone **35** (18.2 mg, 0.1 mmol, 1.0 eq.), diphenylphosphine oxide **36** (20.2 mg, 0.1 mmol, 1.0 eq.), diphenylphosphinic amide **37** (21.7 mg, 0.1 mmol, 1.0 eq.), diphenylphosphinic acid **38** (21.8 mg, 0.1 mmol, 1.0 eq.), azabicyclo[1.1.0]butane **32** (15.9 mg, 0.1 mmol, 1.0 eq.) and were dissolved using the anhydrous DCE (0.1 M, 1.0 mL, commercially available, 99.8% from Sigma-Aldrich).The Schlenk tubes were then closed with the proper greased glass stoppers. The resulting solution was subjected to three cycles of free-pump-thaw to remove the oxygen and in the last cycle, the vessel was filled with argon. The reaction was irradiated under 456 nm blue LEDs for 20 hours with fan cooling. Afterwards, LED was removed and the solvent was evaporated under reduced pressure to afford the crude mixture and was analyzed by ^{1}H and ^{31}P NMR spectroscopy using 1,3,5-trimethoxybenzene and triphenyl phosphite as an internal standard. No formation of the desired N-phosphoryl azetidine **34** was observed under these conditions.

Also reducing the amount of diphenylphosphinic acid **38** to 20 mol% didn't afford the N-phosphoryl azetidine **34**.

4.7.5. Reaction performed under completely dry condition.

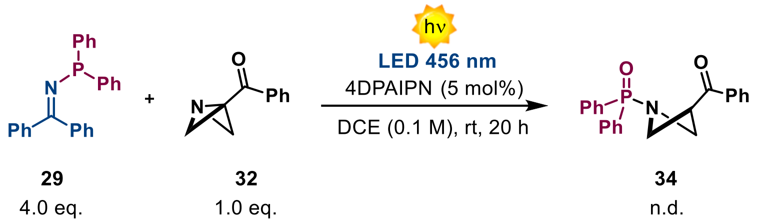

Procedure: In an oven-dried 10.0 mL Schlenk tube containing an oven dried Teflon coated stirring bar was added 4DPAIPN (4.0 mg, 5.0 mol%), iminyl phosphine **29** (0.4 mmol, 4.0 eq.), and azabicyclo[1.1.0]butane **32** (0.1 mmol, 1.0 eq.), inside a glovebox. The Schlenk tube was then closed with the proper septum. After removing the Schlenk tube from the glovebox, freshly distilled DCE (1.0 mL, 0.1 M, from commercially available, 99.8% from Sigma-Aldrich, and dried over CaH_2 under reflux) was added under argon atmosphere. The resulting solution was subjected to three cycles of freeze-pump-thaw (10 minutes each) to remove any traces of oxygen and in the last cycle, the vessel was filled with argon. The reaction was irradiated under 456 nm blue LEDs for 20 hours with fan cooling. Afterwards, LED was removed and the solvent was evaporated under reduced pressure to afford the crude mixture. ^{1}H NMR of the product was determined using 1,3,5-trimethoxybenzene as internal standard. No formation of the desired N-phosphoryl azetidine **34** was observed under these conditions.

4.8. Oxidation by sulfur experiments

To further strengthens the fact that whether radical phosphinyl radical ($Ph_2P\cdot$) **20** or phosphoryl radical $Ph_2P(=O)\cdot$) **24** is responsible for the strain release of compound **32**, sulfur was subsequently added to the reaction mixture after LED irradiation under argon atmosphere, which was then stirred for one hour at ambient temperature. If the phosphinyl radical **20** is responsible for the azabicyclo[1.1.0]butanes **32** opening, the formation of compound **33** via sulfur oxidation should be observed (Figure S10). The absence of **33** along with generation of 5% of **30** (formed from the oxidation of unreacted **29**) suggests that the phosphoryl radical **24** is the only species responsible for strain release of **32**.

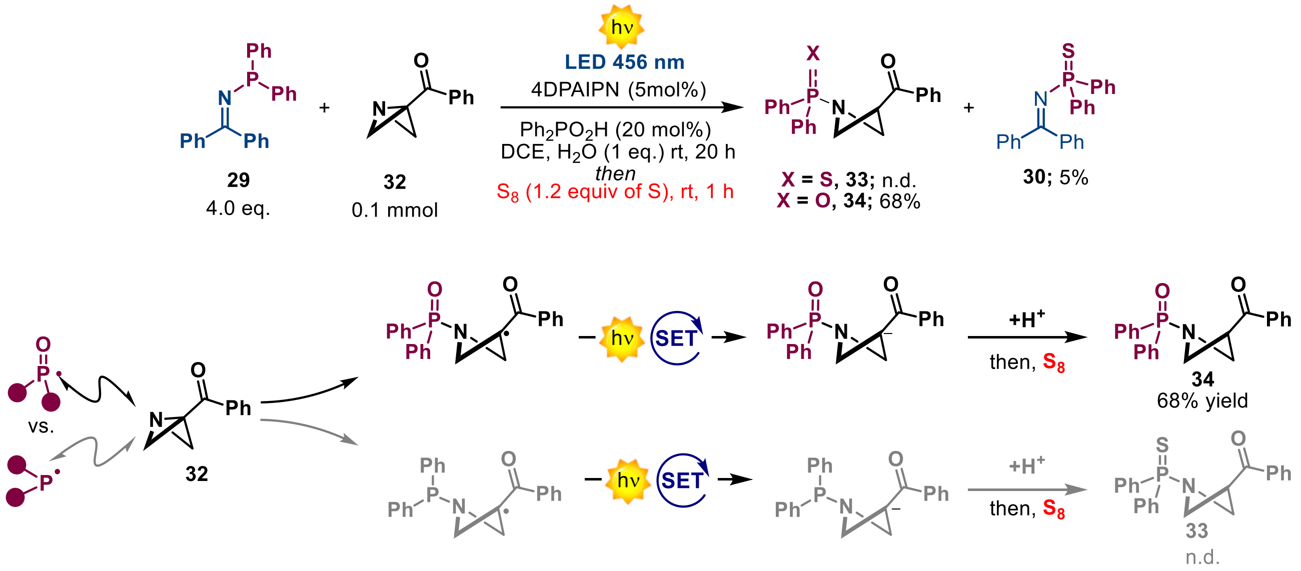

Figure S10: Oxidation by sulfur experiments.

4.9. Activation energy barriers for radical addition.

This finding is further supported by density functional theory (DFT) calculations performed at the UωB97X-D/Def2-SVP level of theory in dichloromethane (SMD solvation model). The addition of a phosphinyl radical ($Ph_2P\cdot$) **20** to azabicyclo[1.1.0]butane **32** exhibits a relatively high activation barrier ($\Delta E^\ddagger = 14.1$ kcal mol^{-1}; $\Delta G^\ddagger = 13.6$ kcal mol^{-1}). In contrast, the phosphoryl radical $Ph_2P(=O)\cdot$ **24** displays markedly enhanced reactivity toward

azabicyclo[1.1.0]butane **32** (Figure S11), with a substantially lower activation barrier ($\Delta E^{\ddagger}$ = 8.9 kcal mol^{-1}; $\Delta G^{\ddagger}$ = 10.4 kcal mol^{-1}) and a pronouncedly exergonic reaction profile (ΔE = -22.0 kcal/mol; ΔG = -21.8 kcal/mol *vs* ΔE = -43.2 kcal/mol; ΔG =-42.1 kcal/mol).

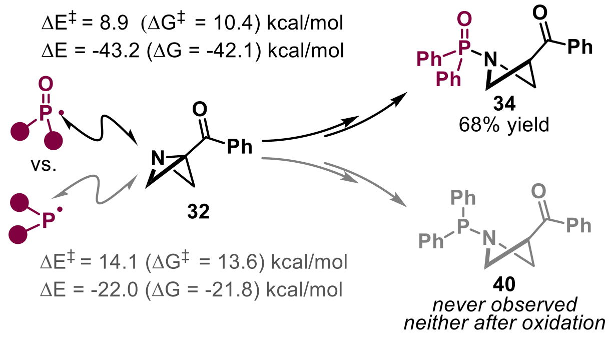

Figure S11: DFT calculations for comparison of activation energy barriers for strain release of **32** by **20** and **24**.

4.10. Deuterium incorporation studies

To shed light on the second part of the catalytic manifold, we performed a reaction using 1.0 eq. of D_2O, that resulted in 40% deuterium incorporation at C3 of the azetidine core. This finding suggests that upon radical opening of the azabicyclo[1.1.0]butanes **32**, the transiently generated tertiary radical is reduced into the corresponding carbanion **41** that reacts with H_2O, as a proton source (Figure S12).

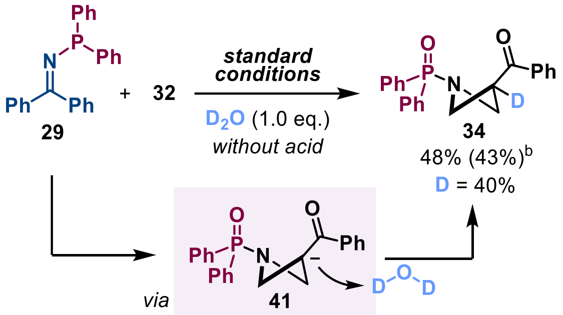

Figure S12: Deuterium incorporation studies to identify the source of hydrogen.

4.11. Experimental verification of the feasibility of β-scission pathway for the generation of 24.

The superior performance of diphenylphosphinic acid **38** in enhancing the reaction yield, relative to other acid additives (Table S4, entry 5 and 6) prompted us to consider its potential nucleophilic attack on **29**$^{\bullet+}$, followed by a β-scission pathway to generate the radical **24** (Figure S13). This process would be analogous to acyl radical generation from carboxylate anions and phosphine radical cations.[23]

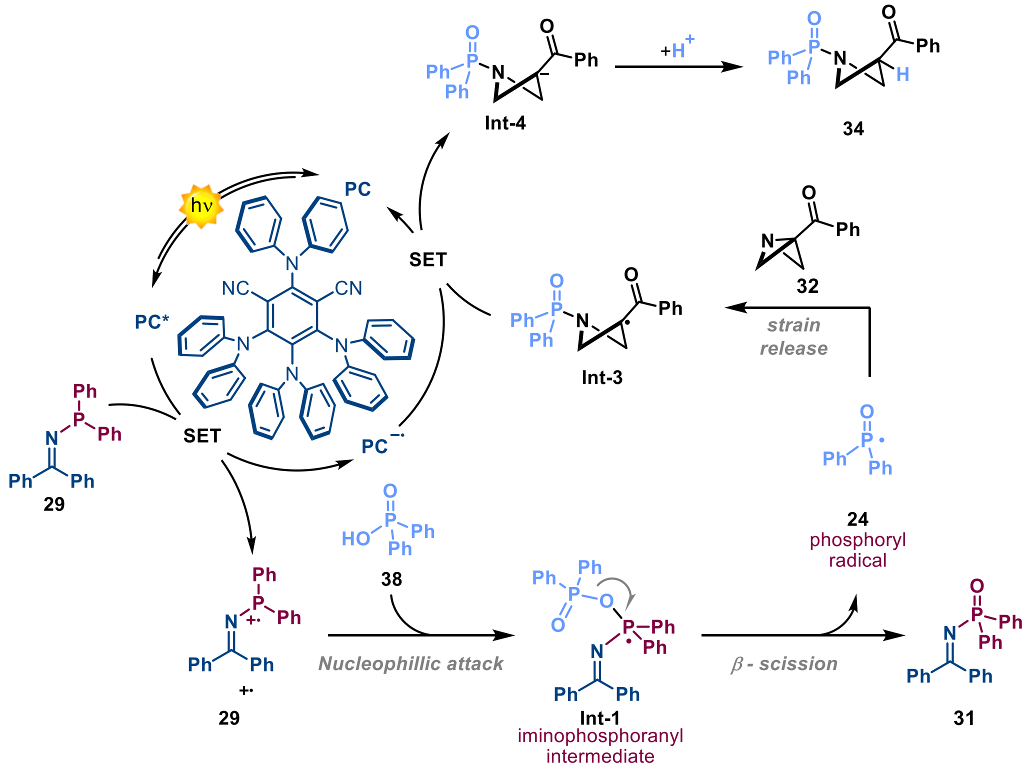

Figure S13: Alternative possible mechanistic route via a β- scission pathway for the generation of **24** from **29**$^{\bullet+}$.

To verify this mechanistic hypothesis, we replaced the model iminyl phosphine **29** with the corresponding phosphite derivative **42** and carried out the reaction under the standard photochemical conditions. It should be noted that the radical **63** generated from iminyl phosphine **42** exhibits comparable reactivity towards azabicyclo[1.1.0]butane **32** to that of radical **24** as predicted by DFT calculation (Figure S14).

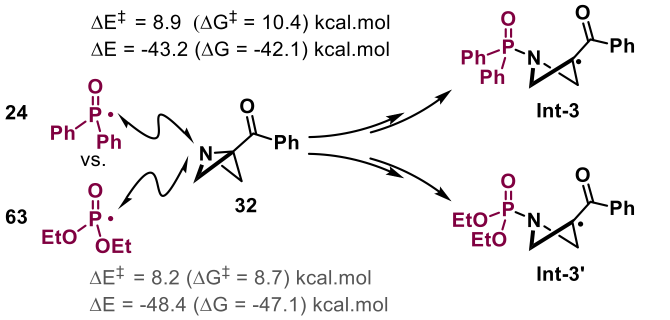

Figure S14: Calculated activation energy barriers for **24** and **63** towards the radical strain release of **32**.

As shown in Figure S15, generation of the phosphoryl radical ($Ph_2P(=O)\cdot$) **24** via β-scission of the phosphoranyl radical intermediate **44** would be expected to produce N-phosphoryl azetidine **34** following radical addition then single-electron transfer (SET) reduction and protonation of the anion. Thus, detection of **34** would provide clear evidence for the intermediary of this pathway. However, **34** was not observed under these conditions; instead, azetidine **45** was formed in 50% yield (Figure S15). The absence of **34** indicates that β-scission of intermediate **44** does not occur, thereby ruling out this pathway as a viable route for the formation of phosphoryl radical **24** from **29**$^{\bullet+}$. Moreover, the unfeasibility of β-scission pathway is also confirmed by computational studies (Refer Section 5.0).

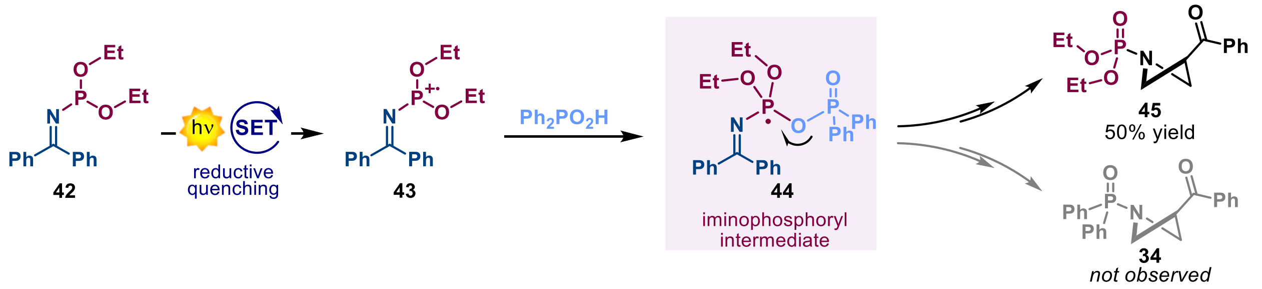

Figure S15: Mechanistic route employing iminyl phosphine **42** in presence of **38**.

4.12. Determination of Molar extinction coefficient

We determined the molar extinction coefficients of both the iminyl phosphine **29** and the photocatalyst **4DPAIPN** at the irradiation wavelength of 456 nm.

At 456 nm;

- **29**: ε = 85 M^{-1} cm^{-1}
- **4DPAIPN**: ε = 6630 M^{-1} cm^{-1}

Calculation of the relative light absorption contributions using the Beer-Lambert relationship:

Given that the catalyst has a much higher extinction coefficient (6630 M^{-1} cm^{-1}) and is present in catalytic amounts (5.0 mol%), while the iminyl phosphine **29** is present in higher equivalents, we evaluated the absorption competition constants (K_c).

Entry	4DPAIPN (mol%)	29 (eq.)	4DPAIPN:29	$f_{4DPAIPN}$	f_{29}	$f_{4DPAIPN}$ (%)	f_{29} (%)	K_c
1	5.0	4.0	1:80	0.494	0.506	49.4	50.6	1.03
2	5.0	3.0	1:60	0.565	0.435	56.5	43.5	0.77
3	5.0	2.0	1:40	0.661	0.339	66.1	33.9	0.51
4	5.0	1.0	1:20	0.796	0.204	79.6	20.4	0.26

Table S10: Determination of absorption competition constants (K_c), where f corresponds to 'fraction of absorption of light by each components'.

Method of calculation

For 4.0 equivalents of **29**,

[**29**]: [**4DPAIPN**] = 80:1

Let [**4DPAIPN**] = x

[**29**] = 80x

For **29**, $A = \varepsilon c \times l = 85 \times 80x = 6800x$

For **4DPAIPN**, $A = \varepsilon c \times l = 6630x$

Total absorption $= 6800x + 6630x = 13430x$

Fraction of absorption of light by **29** $= 6800/13430 = 0.506$

Fraction of absorption of light by **4DPAIPN** $= 6630/13430 = 0.494$

At 456 nm, the absorption competition analysis shows:

- **29** contributes ~50.6% of total photon absorption
- **4DPAIPN** contributes ~49.4% of total photon absorption

The relative absorption constant can therefore be estimated as:

$$K_c = \frac{\varepsilon\,(\mathbf{29}) \times c(\mathbf{29})}{\varepsilon\,(PC) \times c(PC)} = \frac{85 \times 80}{6630 \times 1} = 1.03$$

For 3.0 equivalents of **29**,

$$K_c = \frac{\varepsilon\,(\mathbf{29}) \times c(\mathbf{29})}{\varepsilon\,(PC) \times c(PC)} = \frac{85 \times 60}{6630 \times 1} = 0.77$$

For 2.0 equivalents of **29**,

$$K_c = \frac{\varepsilon\,(\mathbf{29}) \times c(\mathbf{29})}{\varepsilon\,(PC) \times c(PC)} = \frac{85 \times 40}{6630 \times 1} = 0.51$$

For 1.0 equivalents of **29**,

$$K_c = \frac{\varepsilon\,(\mathbf{29}) \times c(\mathbf{29})}{\varepsilon\,(PC) \times c(PC)} = \frac{85 \times 20}{6630 \times 1} = 0.26$$

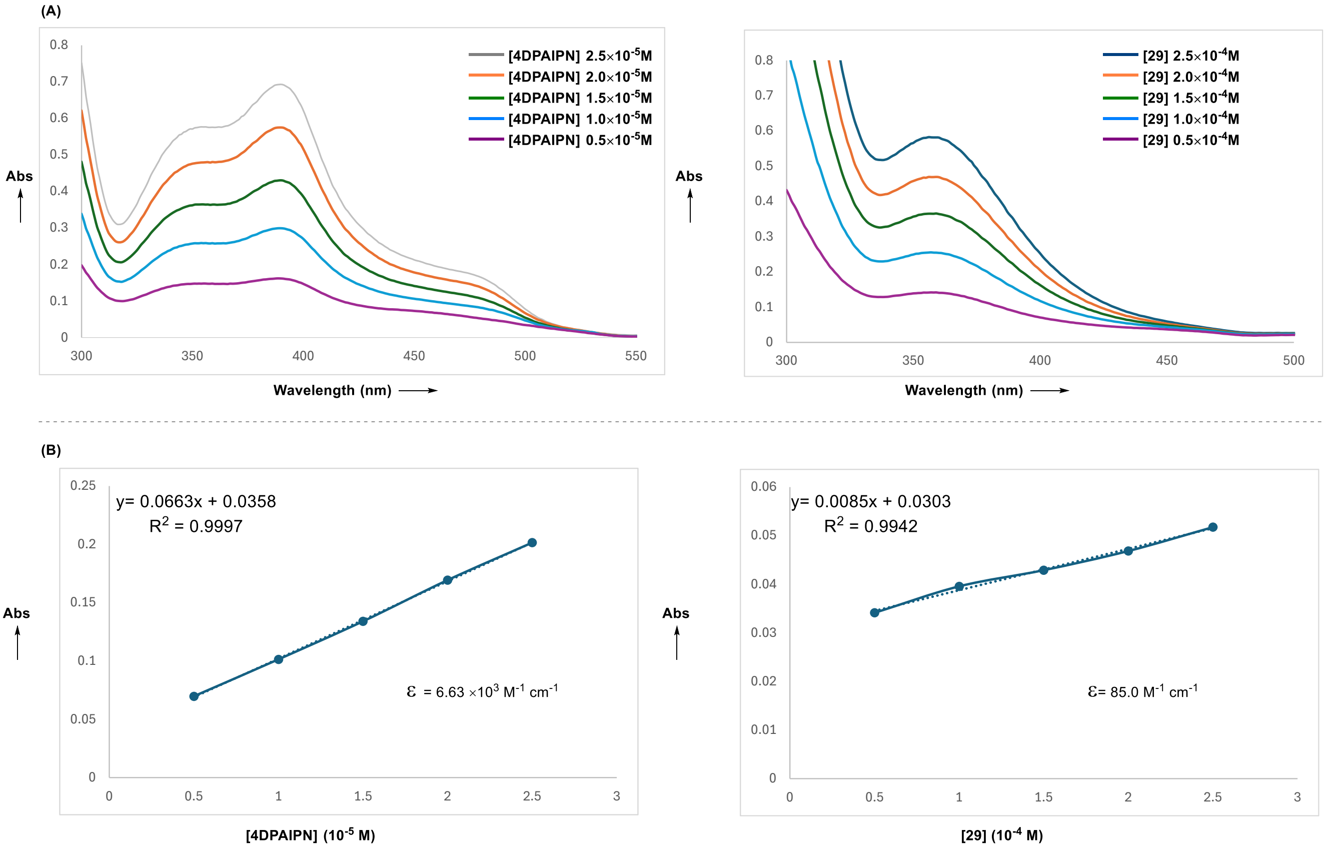

Figure S16: (A) UV-vis absorption studies for **4DPAIPN** and **29. (**B) Beer-Lambert plot for determining the extinction coefficients of **29** and the **4DPAIPN** at the 456 nm wavelength.

Despite the higher molar extinction coefficient of **4DPAIPN**, the large excess of substrate **29** results in comparable light absorption by both species under the reaction conditions. Therefore, based on absorption considerations alone, direct excitation of **29** cannot be completely excluded.

However, several additional experimental and mechanistic factors strongly disfavour a productive role of direct excitation of **29**. First, **29** is not fully stable under the reaction conditions and undergoes a competing hydrolysis pathway leading to the formation of byproducts **31** and **35–38**. Consequently, the effective steady-state concentration of **29** available for photoexcitation is lower than the nominal 4 equivalents used in the absorption analysis. Second, the calculated bond dissociation enthalpy (BDE) of **29** (52.3 kcal·mol⁻¹) suggests that radical species generated upon direct excitation of **29** can undergo a rapid radical recombination. Third, control experiments demonstrate that direct irradiation of **29** in the absence of photocatalyst, even at 370 nm, does not lead to formation of the desired product,

indicating that excitation of **29** alone is not productive. Finally, variation of the **29** loading (1–3 equivalents of **29**) still affords the desired product in moderate to good yields, further indicating that substrate excitation is not the determining factor for reactivity. Taken together with the results from cyclic voltammetry and Stern-Volmer quenching studies, support the fact that the reaction proceeds predominantly via excitation of the photocatalyst **4DPAIPN**, while direct excitation of **29** does not contribute significantly to the desired reaction pathway.

4.13. Quantum yield measurement

A ferrioxalate actinometry solution was prepared by following the Hammond variation of the Hatchard and Parker procedure outlined in Handbook of Photochemistry.[24] Ferrioxalate actinometer solution measures the decomposition of ferric ions to ferrous ions, which are complexed by 1,10-phenanthroline (complete complexation takes about an hour), and monitored by UV/Vis absorbance at 510 nm.[25] The moles of iron-phenanthroline complex formed are related to moles of photons absorbed.

Actinometry

The following solutions were prepared and stored in the dark:

Solution A

20 mL of 1,10-phenantroline 0.2% by weight in water

Solution B (Buffer)

a. 8.2 g $NaOAc{\cdot}H_2O$

b. 1 mL concentrated H_2SO_4 (98%)

c. Diluted to 100 mL with water

Solution C ($Fe_2(SO_4)_3$ solution)

a. 10 g $Fe_2(SO_4)_3{\cdot}nH_2O$ (approximately 20% Fe by weight)

b. 5.5 mL concentrated H_2SO_4 (98%)

c. Diluted to 100 mL with water

Solution D

100 mL of $K_2C_2O_4$ 1.2 M in water

The actinometric solution ($K_3Fe(C_2O_4)_3$ solution) was prepared in the dark by mixing 0.5 mL of $Fe_2(SO_4)_3$ solution with 0.5 mL of $K_2C_2O_4$ solution in a volumetric flask and diluting to 10 mL. The $K_3Fe(C_2O_4)_3$ solution was stored in the dark. The actinometric measurements were done as follows:

Step 1. 1.5 mL of the $K_3Fe(C_2O_4)_3$ solution was added to the Schlenk tube with a magnetic stirrer bar.

Step 2. The tube was irradiated under stirring with a PR160L 456 nm Kessil lamp set at 25% of its maximum output power placed at a distance of 20 cm for increasing amount of time (5, 10, 20, 30 and 45 seconds).
Note: Since the absorbance of the $K_3Fe(C_2O_4)_3$ solution at 456 nm is greater than 2, all the incident photons are absorbed, and no correction factors shall be applied in the following calculations.

Step 3. 1.0 mL of the irradiated solution was transferred to a 10 mL volumetric flask, to which 2 mL of the 0.2% 1,10-phenantroline solution and 0.5 mL of the buffer solution were added. The flask was filled to the mark with water and the content thoroughly mixed.

Step 4. A blank sample was prepared repeating **step 3** with 1 mL $K_3Fe(C_2O_4)_3$ solution kept in the dark without irradiation.

Step 5. The absorbances at 510 nm of the solutions prepared according to **step 3** at increasing time intervals were measured and their difference with the blank ΔA was calculated (Figure S17). The absorbance at 510 nm of the blank was lower than A = 0.6 as recommended.

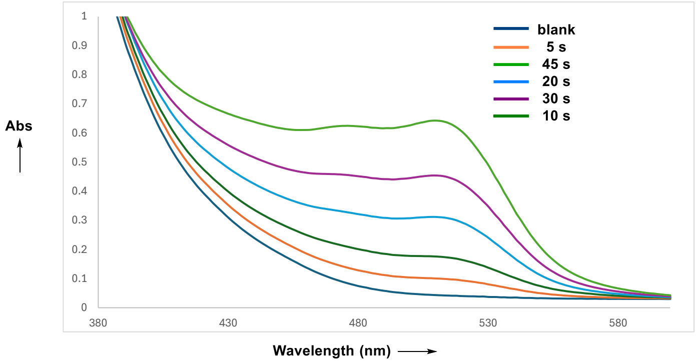

Figure S17: Absorbance spectra of the solutions prepared according to step 3 at increasing time intervals.

The values of ΔA obtained were plotted as a function of the irradiation time (5, 10, 20, 30 and 45 seconds) and linearly interpolated with the following function:

$$\Delta A = \frac{\varepsilon b \varphi V1I}{V2V3} t$$

where: ΔA: are the values obtained at each time interval in Step 5
b: is the path length of the cuvette
ε: is the extinction coefficient of Fe-1,10-phenantroline complex at 510 nm ($1.11 \cdot 10^4$ M^{-1} cm^{-1})
φ: is the quantum yield of ferrous production at 456 nm (0.90)
V1: is the volume in mL of irradiated $K_3Fe(C_2O_4)_3$ solution transferred to the 10 mL flask (1 mL)
V2: is the volume in L of irradiated $K_3Fe(C_2O_4)_3$ solution (0.001 L)
V3: is the volume in mL of the volumetric flask used for workup of irradiated aliquots (10 mL)
I: is the photon flux in einsteins s^{-1} (moles of photons s^{-1})
t: is the irradiation time in seconds

From the slope of the interpolation (Figure S18), the light intensity was thus calculated to be I = $1.37 \cdot 10^{-8}$ mol s^{-1}

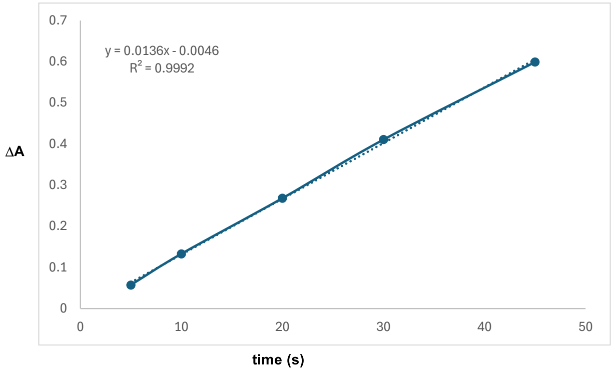

Figure S18. Plot and linear interpolation of the values of ΔA obtained according to step 5 and their linear interpolation.

Quantum yield of the model reaction

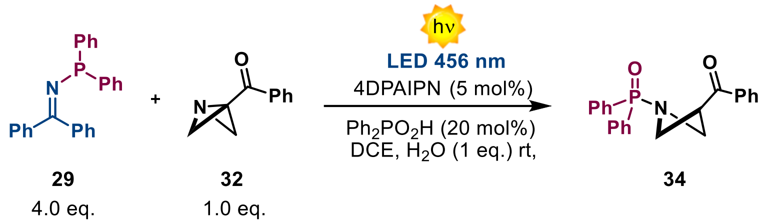

The same Schlenk tube used for actinometry was charged with 4DPAIPN (4.0 mg, 5.0 mol%), diphenylphosphinic acid **38** (4.4 mg, 20.0 mol%), iminyl phosphine **29** (0.4 mmol, 4.0 eq.), the azabicyclo[1.1.0]butane **32** (15.9 mg, 0.1 mmol, 1.0 eq.) and were dissolved using the anhydrous DCE (0.05 M, 2.0 mL, commercially available, 99.8% from Sigma-Aldrich). The absorbance of the reaction mixture was then measured.

The absorbance of the reaction mixture for our photocatalytic reaction at 456 nm is greater than 2. This means that all the incident photons are absorbed, and no correction factors shall be applied in the following calculations.

The fraction of light absorbed (f) can be calculated according to $f=1-10^{-A}\approx 1$. Hence, the reaction mixture absorbs 100% of the incident photons, and the effective photon flux is calculated correcting the photon flux obtained from actinometry, according to

Effective photon flux;

$I_{eff} = f \times I = I_{eff} = f \cdot I$

$\approx 1 \cdot 1.37\times10^{-8}$ mols^{-1}

$= 1.37\times10^{-8}$ mols^{-1}.

The same Schlenk tube used or actinometry containing an oven dried Teflon coated stirring bar was added 4DPAIPN (4.0 mg, 5.0 mol%), diphenylphosphinic acid **38** (4.4 mg, 20.0 mol%), iminyl phosphine **29** (0.4 mmol, 4.0 eq.), the azabicyclo[1.1.0]butane **32** (15.9 mg, 0.1 mmol, 1.0 eq.) and were dissolved using the anhydrous DCE (0.05 M, 2.0 mL, commercially available, 99.8% from Sigma-Aldrich). The Schlenk tubes were then closed with the proper greased glass stoppers. The resulting solution was subjected to three cycles of freeze-pump-thaw to remove the oxygen and in the last cycle, the vessel was filled with argon. The reaction was irradiated under 456 nm blue LEDs with fan cooling. 0.2 mL aliquots of the reaction were sampled after 30, 60, 120, 240 and 360 minutes of irradiation, diluted with 500 µL of $CDCl_3$ and analyzed by ^1H NMR to determine the yield using 1,3,5-trimethoxybenzene as internal standard.

The yield values (mmols of product formed) at given time intervals were plotted against the mmols of photons absorbed by the reaction mixture (obtained by multiplication of the effective photon flux with the irradiation time in seconds). The plot was linearly interpolated and the slope of the interpolation is, by definition, the quantum yield of the reaction (Figure S19). The value of quantum yield is then 0.03.

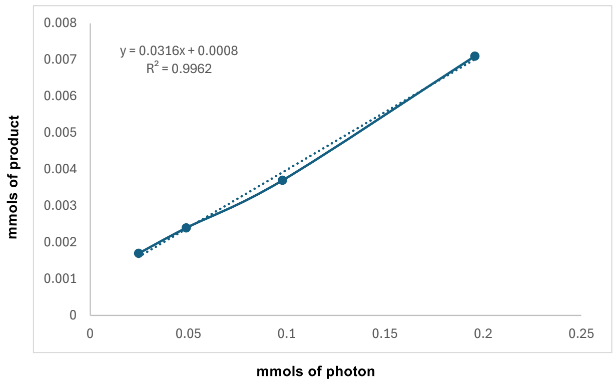

Figure S19: Plot of the mmols of product formed vs mmols of photons absorbed and linear interpolation.

4.14. Comparative study of reported phosphoryl radical precursor 36 in the aza-BCB system

To further highlight the efficiency and advantages of our method for accessing this class of compounds, we investigated the reported phosphoryl radical precursor diphenylphosphine oxide **36** under literature conditions,[22] using azabicyclo[1.1.0]butane **32** as a model substrate. Specifically, we evaluated both a photoredox protocol (condition 1) and a thermal approach (condition 2) previously reported for the generation of phosphoryl radical **24** from **36**. Pleasingly, our photoredox strategy demonstrated clear superiority over the reported phosphoryl radical source in promoting the radical ring-opening of aza-BCBs, leading to the formation of N-phosphoryl azetidine scaffolds.

Experimental procedure for photoredox protocol (Condition 1)

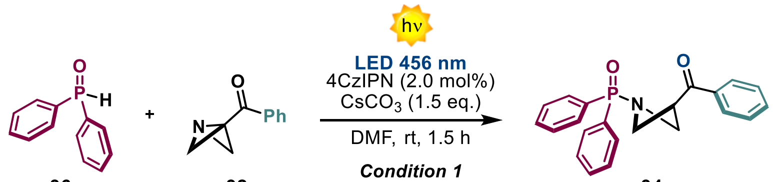

36 1.0 eq. | **32** 1.0 eq. | **34** 7% yield

An oven-dried Schlenk tube (10.0 mL) containing a stirring bar was charged with the **32** (31.8 mg, 0.2 mmol, 1.0 eq.) and 4CzIPN (3.2 mg, 2.0 mol %) under N_2 atmosphere. The Schlenk tube was then introduced in a glovebox, where it was charged with Cs_2CO_3 (97.5 mg ,0.3 mmol, 1.5 eq.) and **36** (40.4 mg, 0.2 mmol, 1.0 eq.). The tube was taken out of the glovebox and connected to a vacuum line where it was evacuated and back-filled with N_2 for 3 times. Then DMF (2 mL) were added under N_2 flow. Finally, the reaction mixture in sealed tube was placed at a distance of 2 ~ 4 cm from a 30 W blue LED and stirred at room temperature (25 °C) for 1.5 h. Then, the mixture was quenched with 1.0 mL of H_2O, extracted with EtOAc, then concentrated in vacuo. ^{1}H NMR yield of the product **34** was determined using 1,3,5-trimethoxybenzene as internal standard.

Experimental procedure for thermal protocol (Condition 2)

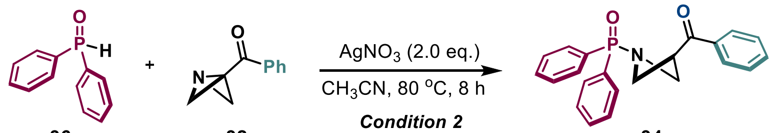

36 2.0 eq. | **32** 1.0 eq. | **34** 0% yield

A mixture of **32** (79.5 mg, 0.5 mmol, 1.0 eq.), **36** (202.0 mg, 1.0 mmol, 2.0 eq.), $AgNO_3$ (169.8 mg, 1.0 mmol, 2.0 eq.) were sequentially added in a 10-mL reaction vial. Then, CH_3CN (3 mL) was added into this reaction system. The reaction vial was sealed and stirred at 80 °Cfor 8 h. Next, the solvent was quenched with water (10.0 mL), extracted with ethyl acetate (3 × 10.0 mL). The combined organic layers were washed with brine (25.0 mL) and dried over anhydrous Na_2SO_4 and the solvent was evaporated in vacuo. ^{1}H NMR yield of the product **34** was determined using 1,3,5-trimethoxybenzene as internal standard.

5. Computational studies.

Refer to section 1.3 for the computational methods.

We opted not to perform extensive conformational exploration of **29**$^{•+}$, but we did perform a (partial) relaxed potential energy surface (PES) scan of the C–P–N–C dihedral in the gas phase, and optimized the lowest-energy minimum found in implicit dichloromethane. We thereafter retained the four aromatic rings in the conformations they adopted after this optimization. Similarly, we also explored and verified: the preferred orientation of the benzoyl group in strained azabicyclo[1.1.0]butanes **32**; perpendicularity of the P=O and C=O bonds in **Int-3** and **Int-4**; and *trans* orientation of the P=O bond with respect to the N lone pair in **Int-3** and **Int-4**.

Unfeasibility of β-scission in **Int-1** was confirmed *via* a rigid and a relaxed potential energy surface scan of the elongation of the outermost P–O bond. The rigid scan encounters a plateau at an electronic energy value (ΔE) of +39.6 kcal.mol^{-1} above **Int-1**. The relaxed scan proceeds smoothly from a P–O length of 1.61 Å to 1.91 Å (not a maximum), after which the unpaired electron migrates from the N-bound P to the diphenyl substituted C, with consequent tightening of the P=N bond (such undesirable situation also significantly complicated the identification of **TS2**): this makes it impossible to compare energies beyond 1.91 Å. However, a Hessian calculation on the structure at P–O = 1.91 Å already points to a free energy difference of +22.5 kcal mol^{-1} with respect to **Int-1**. This is +2.7 kcal mol^{-1} higher than the ΔG‡ for **TS2**, estimated at +11.3 kcal mol^{-1} with respect to **Int-1**.

The nature of all minima and first-order transition states (TSs) was confirmed *via* full Hessian calculations, expecting no imaginary vibrational frequencies in the first case, and only one in the latter. The imaginary vibrational frequency in each TS was always inspected visually to make sure it made chemical sense with respect to the chemical species on either side. Starting from each TS, Intrinsic Reaction Coordinate (IRC) calculations were initiated for a few steps on either side, to make sure the TS really did fall back to the intended chemical species. IRC calculations were either run with the older GS2 algorithm[26] or more recent HPC algorithm[27] available in *Gaussian16* (see individual inputs for details).[3]

Detection of **TS1** proceeded *via* the STQN algorithm[28] inputting the optimized **29**$^{•+}$**–34** complex and **Int-1** (generated from the latter). **TS3** was located starting from **Int-3** and performing a relaxed PES scan synchronously elongating N–C and shortening P–N, followed by simple optimization of the highest-energy structure to a saddle point.

Identification of **TS2** through P–N elongation in **Int-1** was again marred by the migration of the unpaired electron to the diphenyl-substituted C (*cf.* β-scission); the STQN method proved similarly unviable, even when forcing biorthogonalization of unpaired electrons. We thus first generated and optimized the **Int-2–19** complex by further manually elongating P–N in the highest-energy structure prior to electron migration. The complex was then reoptimized along with **Int-1** in the gas phase, at the UωB97X-D/Def2-SVP level,[6,7] using ORCA (v. 5.0.4).[2] Next, to provide a guess for the structure of **TS2**, we again used ORCA at the same level of theory to carry out a CI-NEB calculation[1] with 16 intermediate nodes, using the ORCA-optimized **Int-1** and the **Int-2–19** complex as the reactant and product, respectively. The CI-NEB calculation is not in the online repository, but is provided separately as a zipped folder. The resulting climbing image was successfully optimized with *Gaussian16* to a first-order saddle point (the final **TS2**), resuming the original UωB97X-D/Def2-SVP level of theory[6,7] and implicit dichloromethane. An IRC was sufficient to make sure **TS2** fell back to **Int-1**; to reach the **Int-2–19** complex, it was necessary to displace **TS2** by 0.5 Bohr along the imaginary frequency before reoptimizing.

Atomic charge fitting on optimized **Int-2** was performed using the Merz-Kollman-Singh method.[29]

5.1. Triplet excited energies and BDEs of iminyl phosphine 29-31 and 42.

We have determined the triplet energy (T_1) and spin distribution for compounds **29**, **30**, **31** and **42** utilizing the freely accessible EnT decker machine learning-based (ML) platform as well as density functional theory (DFT) calculation at the UωB97X-D/Def2-SVP level of theory in DCM, employing the SMD solvation model. Triplet energies for **29**, **30**, **31**, and **42** were calculated by switching the multiplicity to 3 on the optimised singlet, conducting a single point calculation, and subtracting the energy of the optimised singlet from the energy of the reoptimised triplet. The bond dissociation energies (BDEs) were calculated using the same level of theory. Energies for the dissociation of the P-N bond were obtained by subtracting the energy of the optimised singlet from the sum of the energies of the optimised iminyl and phosphoryl radical moieties. All calculations are available online.[4]

**Substrate**	**BDE_{DFT} (kcal·mol^{-1})**	T_{1ML} **(kcal·mol^{-1})**	T_{1DFT} **(kcal·mol^{-1})**
**29**	52.3	45.0	40.8
**30**	58.1	43.7	51.4
**31**	67.0	60.3	51.7
**42**	57.6	48.9	44.6

Table S11: T_{1ML}: Triplet energies determined using freely accessible EnT decker machine learning-based (ML) platform. BDE_{DFT} and T_{1DFT} (kcal·mol^{-1}: Calculated using density functional theory (DFT) at the UωB97X-D/Def2-SVP level of theory in DCM, employing the SMD solvation model

6. Crytal structures.

Compound	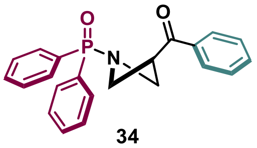 34 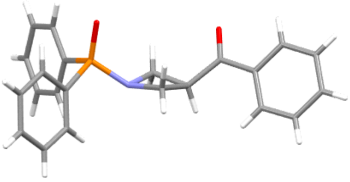
CCDC number	2527691
Empirical formula	$C_{22}H_{20}NO_2P$
Formula weight	361.37
Temperature [K]	298.00
Crystal system	orthorhombic
Space group (number)	$P2_12_12_1$ (19)
a [Å]	6.2453(3)
b [Å]	16.8571(7)
c [Å]	17.6796(7)
α [°]	90
β [°]	90
γ [°]	90
Volume [Å3]	1861.27(14)
Z	4.0
ρcalc [gcm−3]	1.290
μ [mm−1]	1.430
F(000)	760
Crystal size [mm3]	0.07×0.124×0.24
Crystal colour	translucent light colourless
Crystal shape	Fragment
Radiation	Cu$K\alpha$ (λ=1.54178 Å)
2θ range [°]	7.25 to 137.20 (0.83 Å)
Index ranges	$-7 \leq h \leq 7$<br>$-20 \leq k \leq 20$<br>$-21 \leq l \leq 21$
Reflections collected	73233
Independent reflections	3410<br>Rint = 0.0623<br>Rsigma = 0.0231
Completeness to θ = 67.679°	100.0
Data / Restraints / Parameters	3410 / 144 / 236
Goodness-of-fit on F2	1.046
Final R indexes [I≥2σ(I)]	R1 = 0.0240<br>wR2 = 0.0643
Final R indexes [all data]	R1 = 0.0246<br>wR2 = 0.0649
Largest peak/hole [eÅ−3]	0.15/−0.20

Compound	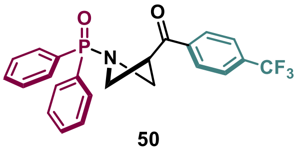 50 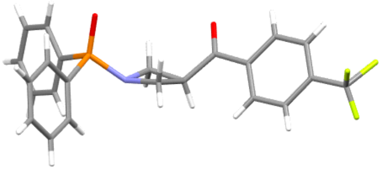
CCDC number	2527692
Empirical formula	$C_{23}H_{19}F_3NO_2P$
Formula weight	429.38
Temperature [K]	298.15
Crystal system	monoclinic
Space group (number)	$P2_1/n$ (14)
a [Å]	16.7817(5)
b [Å]	6.2535(2)
c [Å]	24.3810(7)
α [°]	90
β [°]	90.776(2)
γ [°]	90
Volume [Å3]	2558.41(13)
Z	4.0
*ρ*calc [gcm−3]	1.170
μ [mm−1]	1.779
F(000)	930
Crystal size [mm3]	0.03×0.16×0.5
Crystal colour	translucent light colourless
Crystal shape	prism
Radiation	Cu*Kα* (λ=1.54178 Å)
2θ range [°]	6.35 to 149.99 (0.80 Å)
Index ranges	−20 ≤ h ≤ 20<br>−7 ≤ k ≤ 6<br>−30 ≤ l ≤ 30
Reflections collected	124673
Independent reflections	5231<br>*R*int = 0.0795<br>*R*sigma = 0.0314
Completeness to θ = 67.679°	99.9
Data / Restraints / Parameters	5231 / 87 / 298
Goodness-of-fit on *F*2	1.063
Final *R* indexes [*I*≥2σ(*I*)]	*R*1 = 0.0503<br>w*R*2 = 0.1349
Final *R* indexes [all data]	*R*1 = 0.0589<br>w*R*2 = 0.1422
Largest peak/hole [eÅ−3]	0.40/−0.27

Compound	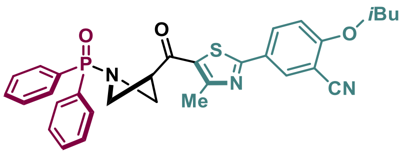 **60** 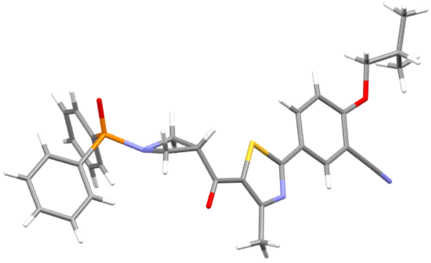
CCDC number	2527693
Empirical formula	$C_{31}H_{30}N_3O_3PS$
Formula weight	555.63
Temperature [K]	298.00
Crystal system	monoclinic
Space group (number)	$P2_1/c$ (14)
a [Å]	17.9494(5)
b [Å]	18.5593(6)
c [Å]	8.9236(3)
α [°]	90
β [°]	99.845(2)
γ [°]	90
Volume [Å3]	2928.93(16)
Z	4.0
*ρ*calc [gcm−3]	1.260
μ [mm−1]	1.787
F(000)	1168
Crystal size [mm3]	0.03×0.05×0.14
Crystal colour	translucent light colourless
Crystal shape	Fragment (Prism)
Radiation	Cu*Kα* (λ=1.54178 Å)
2θ range [°]	5.00 to 150.08 (0.80 Å)
Index ranges	$-22 \leq h \leq 20$<br>$-23 \leq k \leq 23$<br>$-10 \leq l \leq 11$
Reflections collected	21649
Independent reflections	5899<br>*R*int = 0.1028<br>*R*sigma = 0.0792
Completeness to θ = 67.679°	99.6
Data / Restraints / Parameters	5899 / 12 / 340
Goodness-of-fit on *F*2	1.065
Final *R* indexes [*I*≥2σ(*I*)]	*R*1 = 0.0818<br>w*R*2 = 0.2213
Final *R* indexes [all data]	*R*1 = 0.1367<br>w*R*2 = 0.2667
Largest peak/hole [eÅ−3]	1.07/−0.72
Extinction coefficient	0.0030(7)

7. NMR spectra

^{1}H NMR spectra of compound **29** in CD_2Cl_2

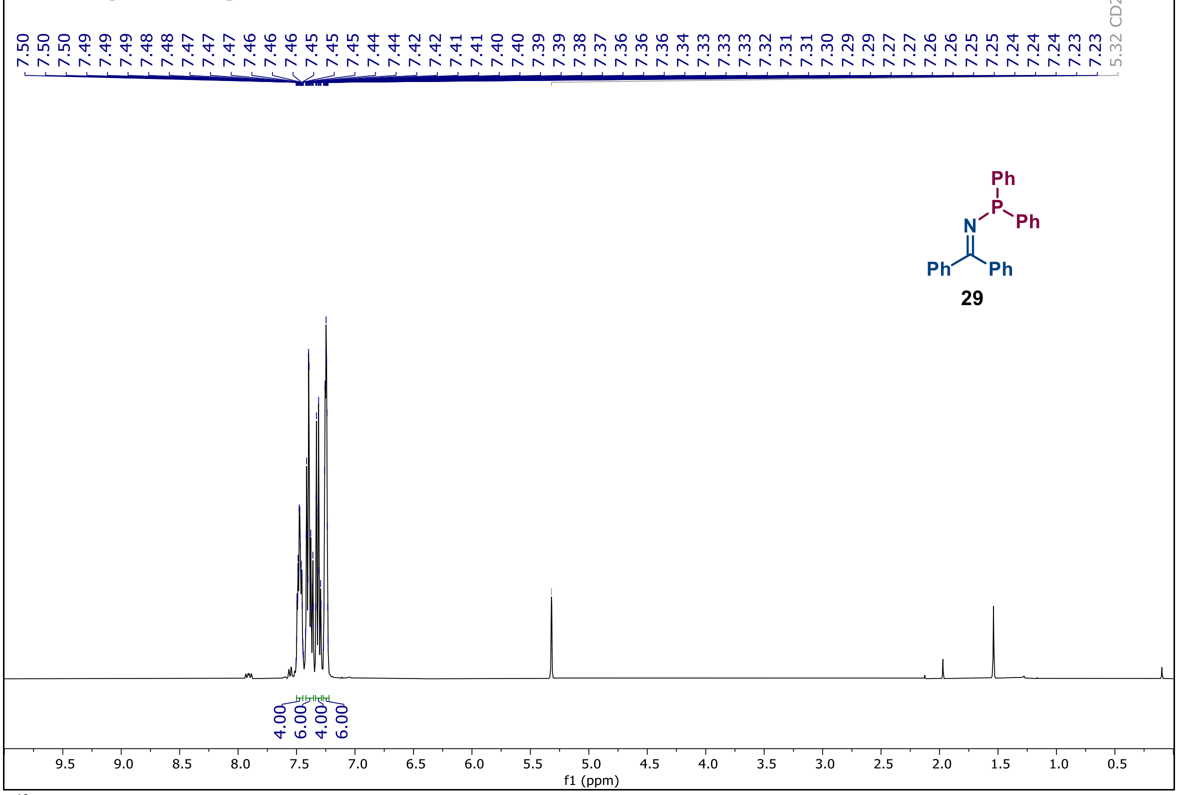

^{13}C NMR spectra of compound **29** in CD_2Cl_2

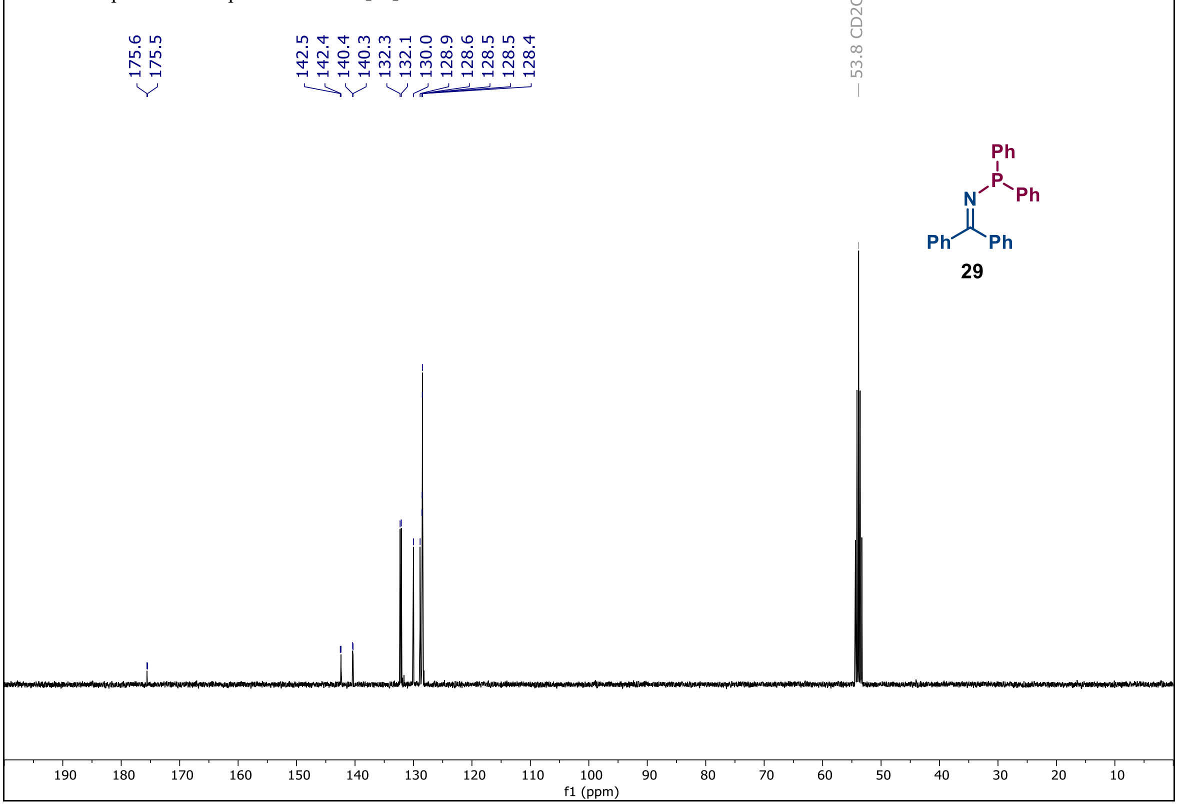

^{31}P NMR spectra of compound **29** in CD_2Cl_2

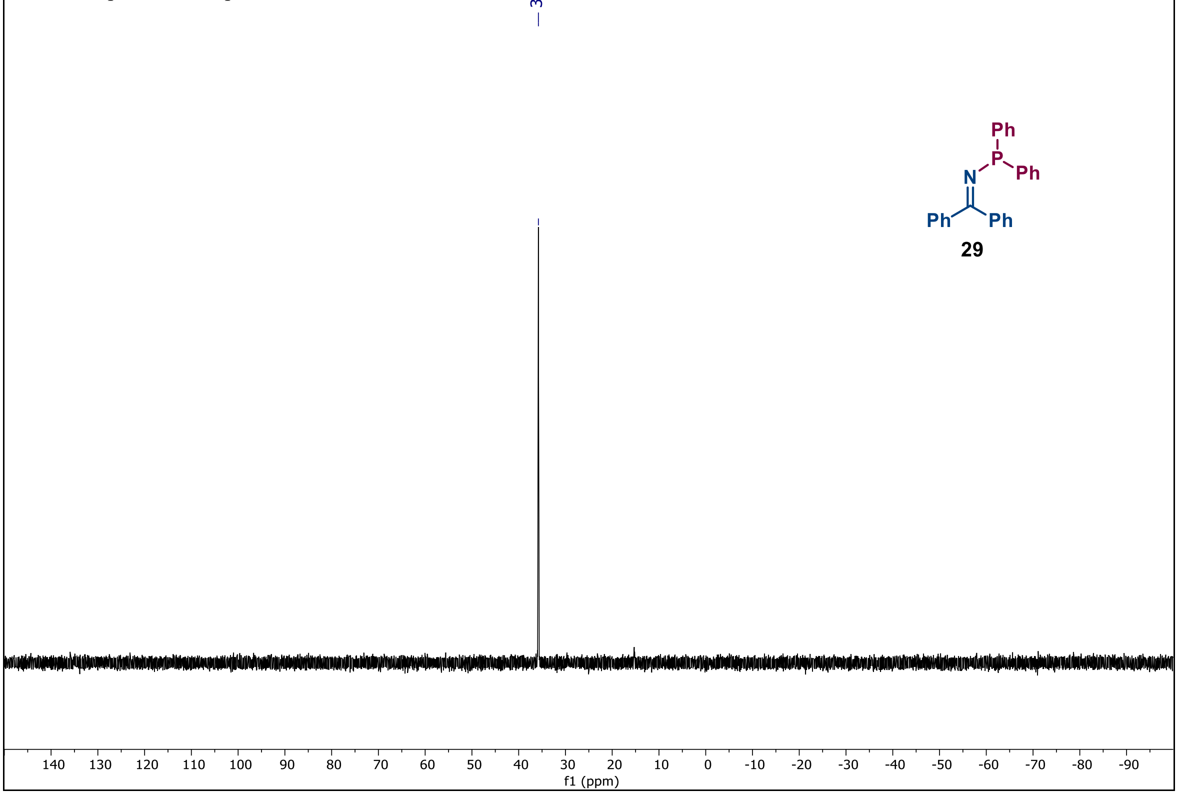

^{1}H NMR spectra of compound **68** in $CDCl_3$

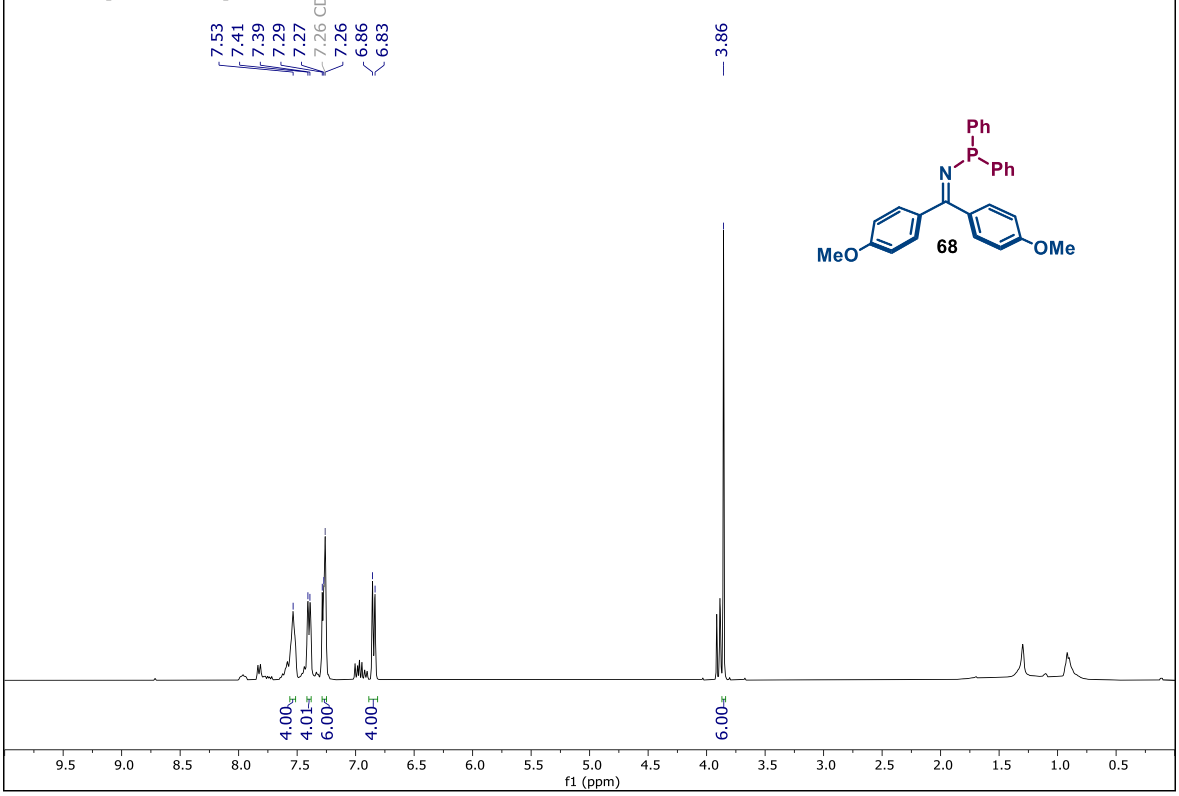

^{13}C NMR spectra of compound **68** in $CDCl_3$

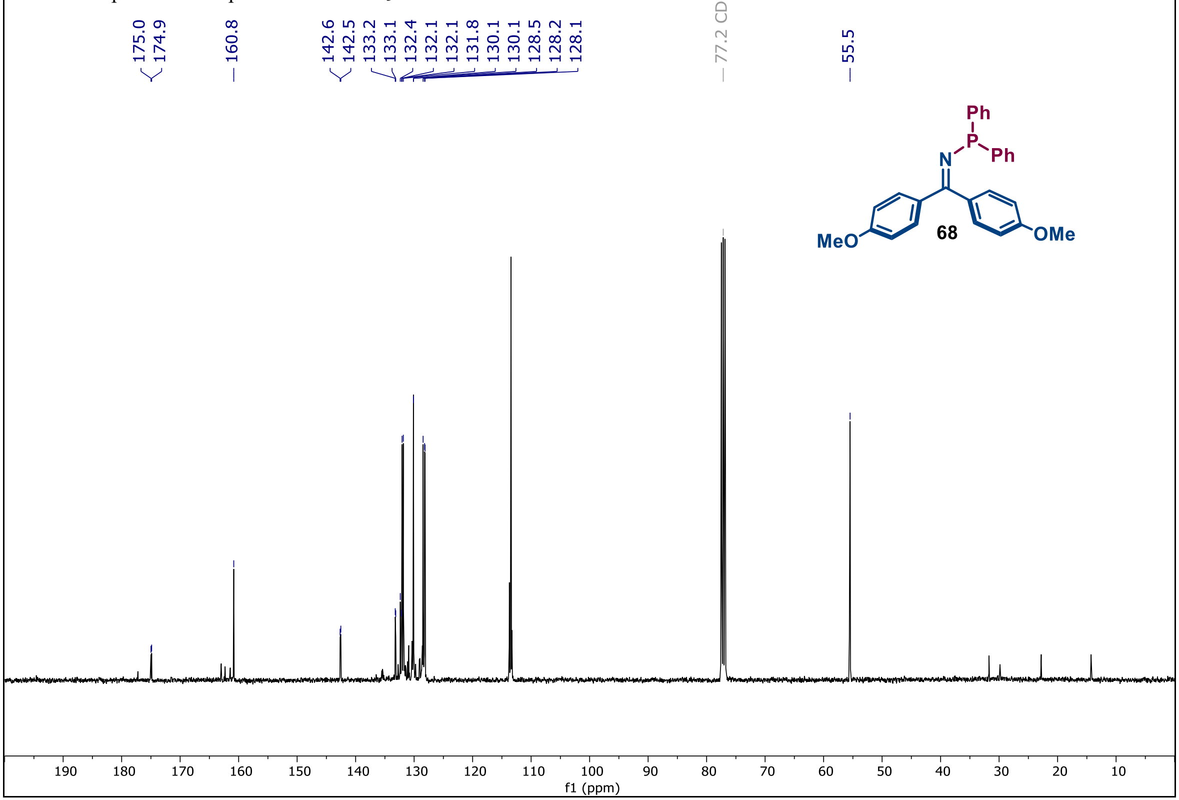

^{31}P NMR spectra of compound **68** in $CDCl_3$

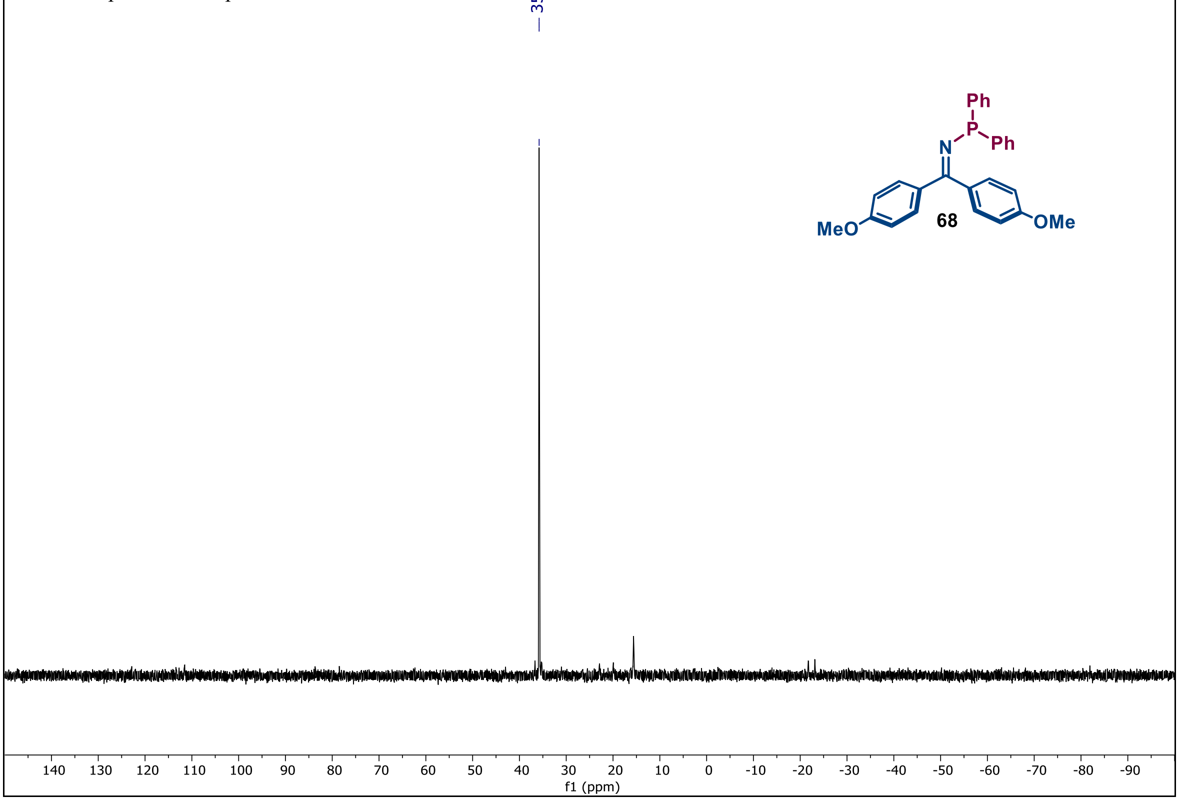

^{1}H NMR spectra of compound **69** in $CDCl_3$

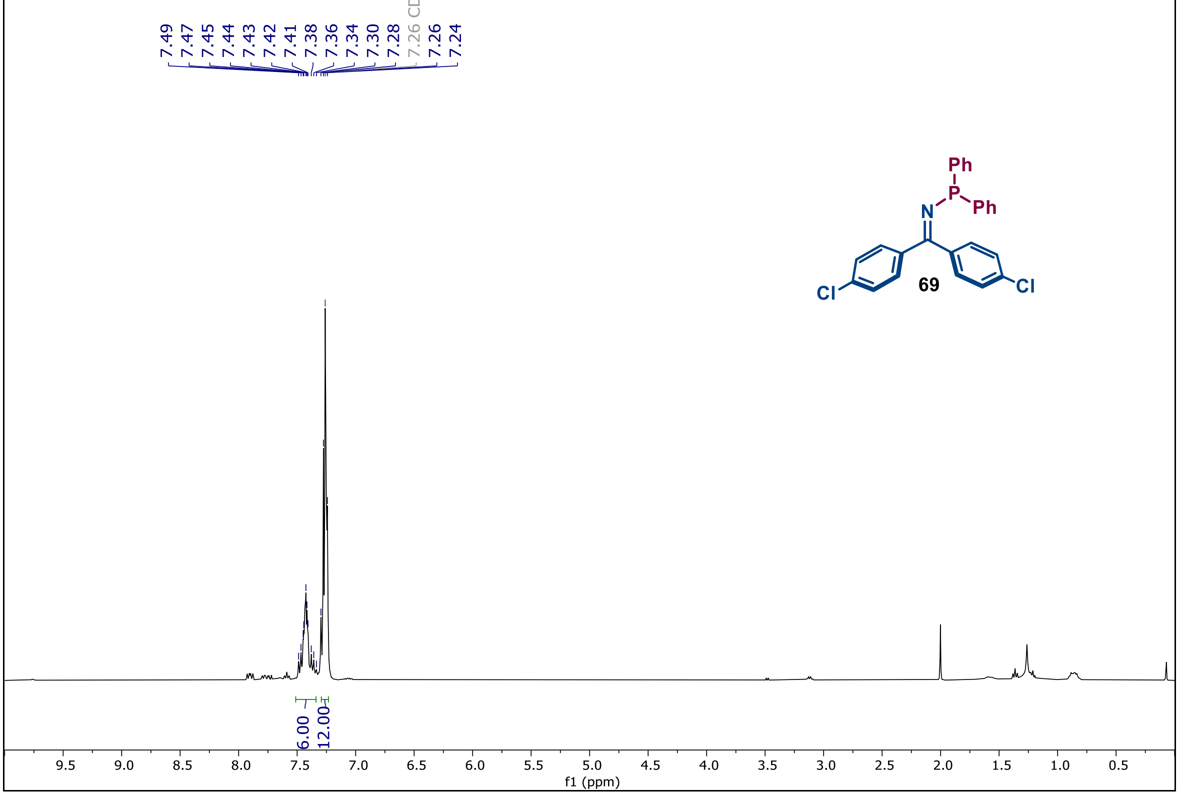

^{13}C NMR spectra of compound **69** in $CDCl_3$

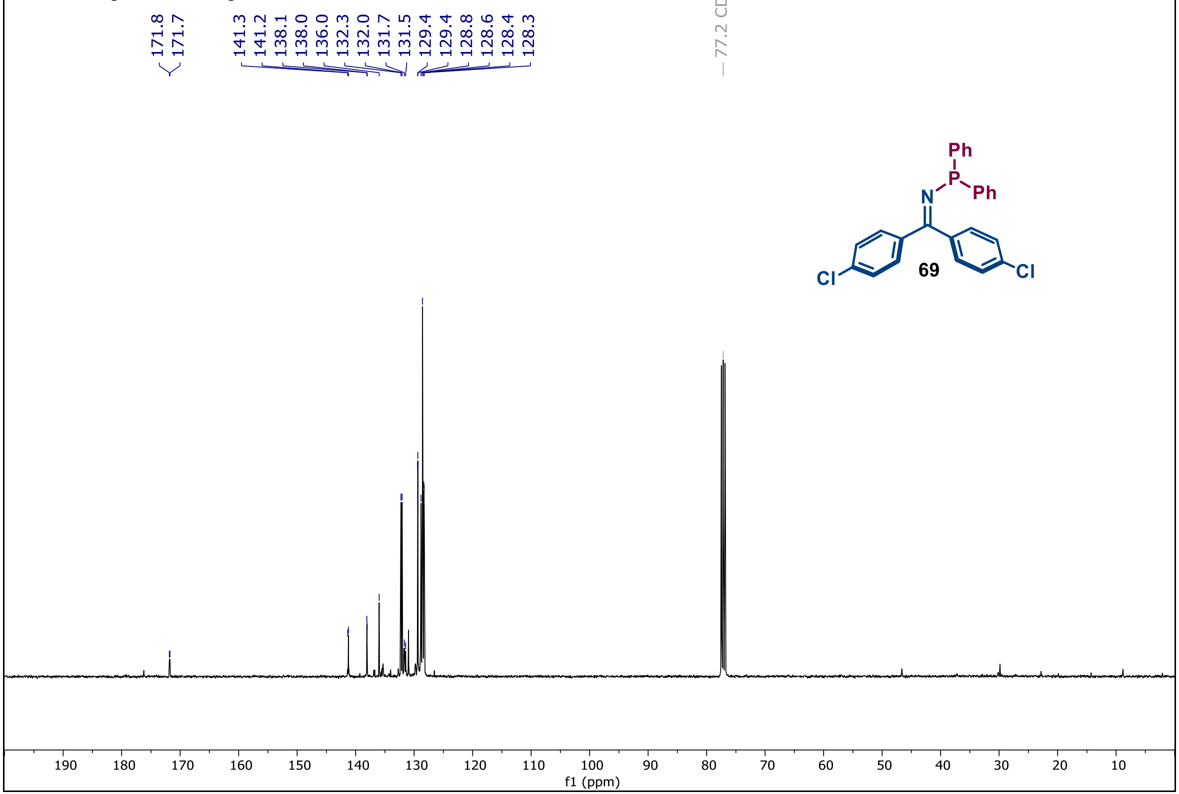

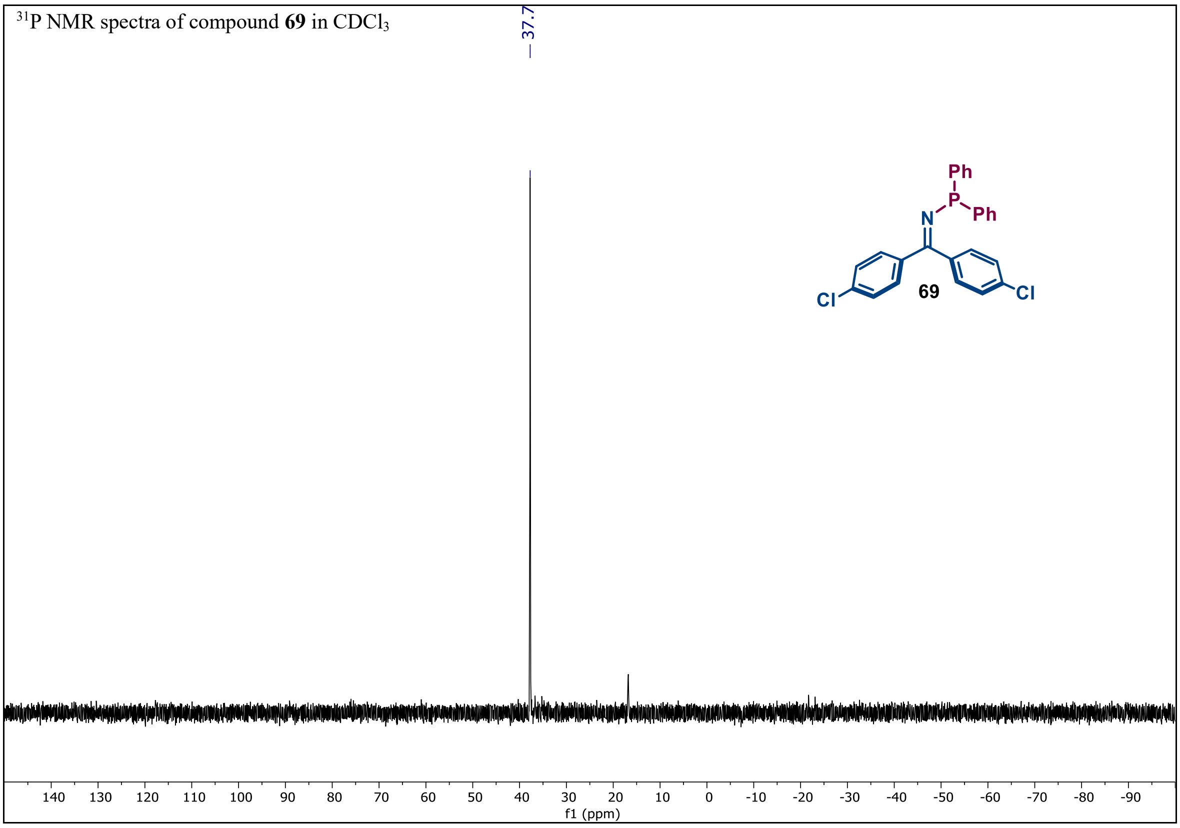

^{31}P NMR spectra of compound **69** in $CDCl_3$

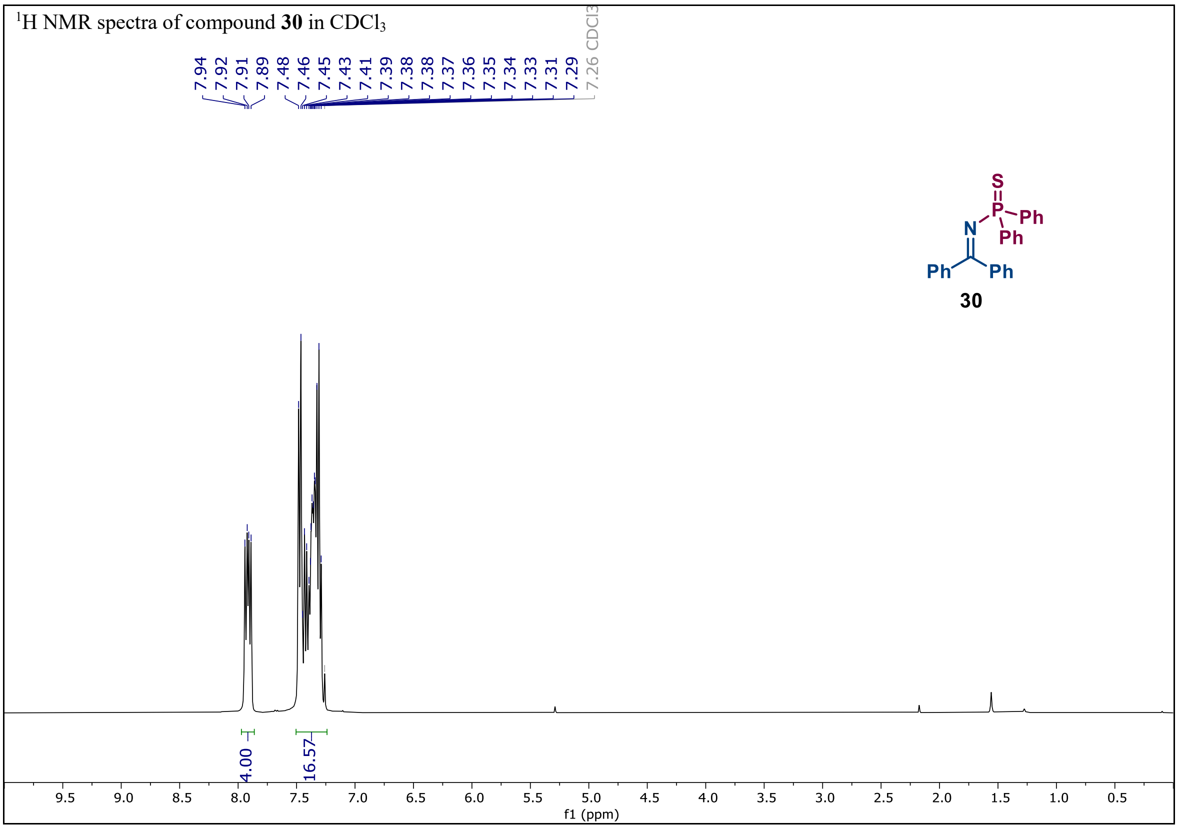

^{1}H NMR spectra of compound **30** in $CDCl_3$

^{13}C NMR spectra of compound **30** in $CDCl_3$

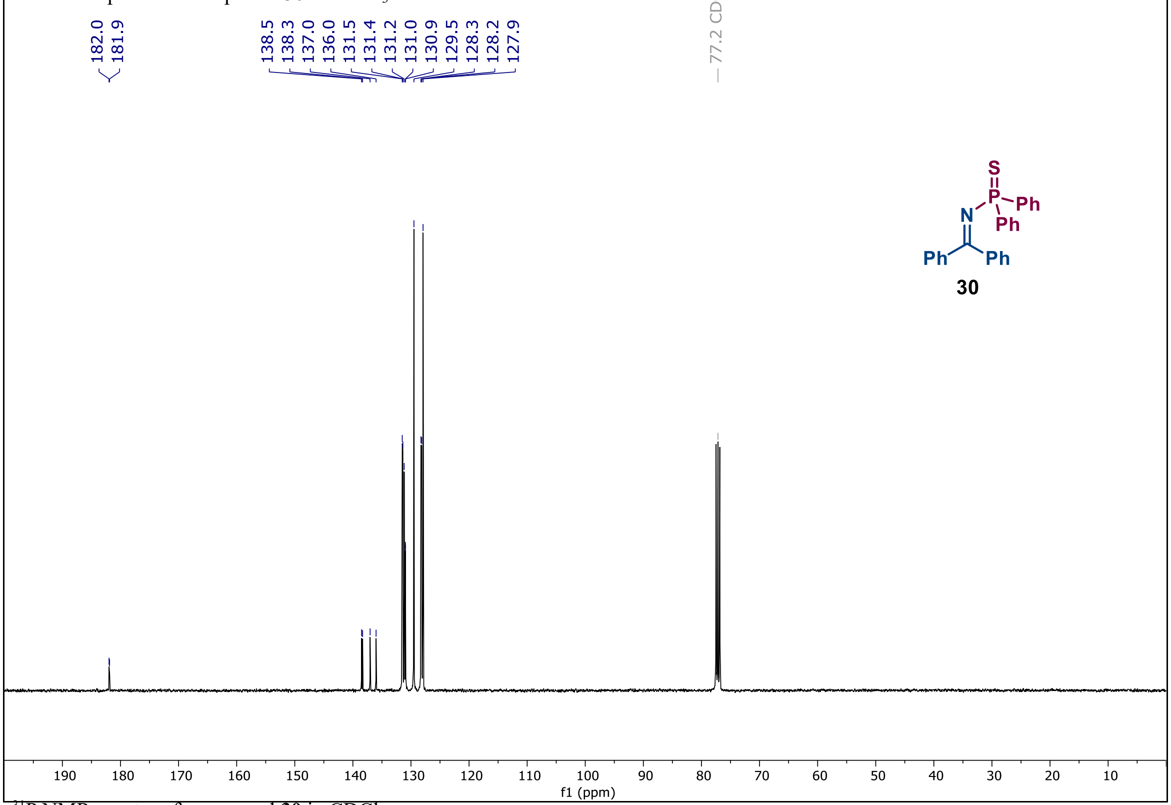

^{31}P NMR spectra of compound **30** in $CDCl_3$

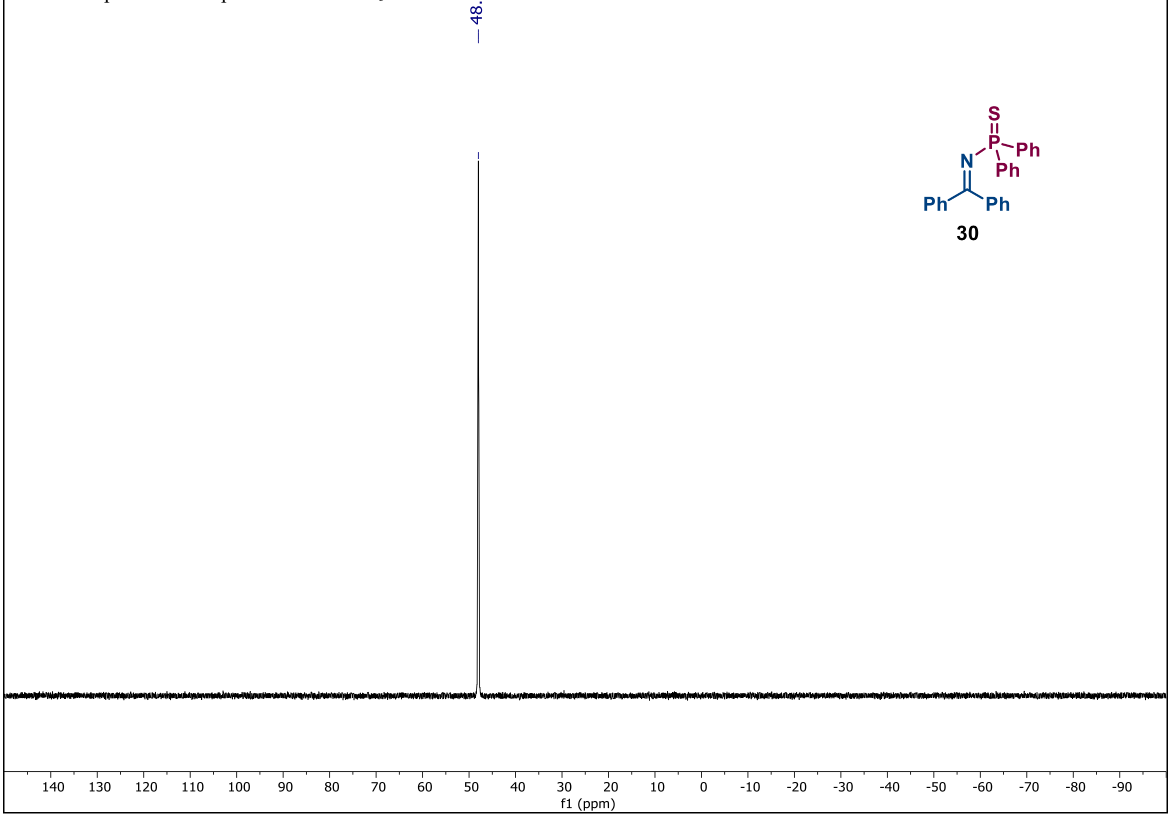

^{1}H NMR spectra of compound **30** in $CDCl_3$

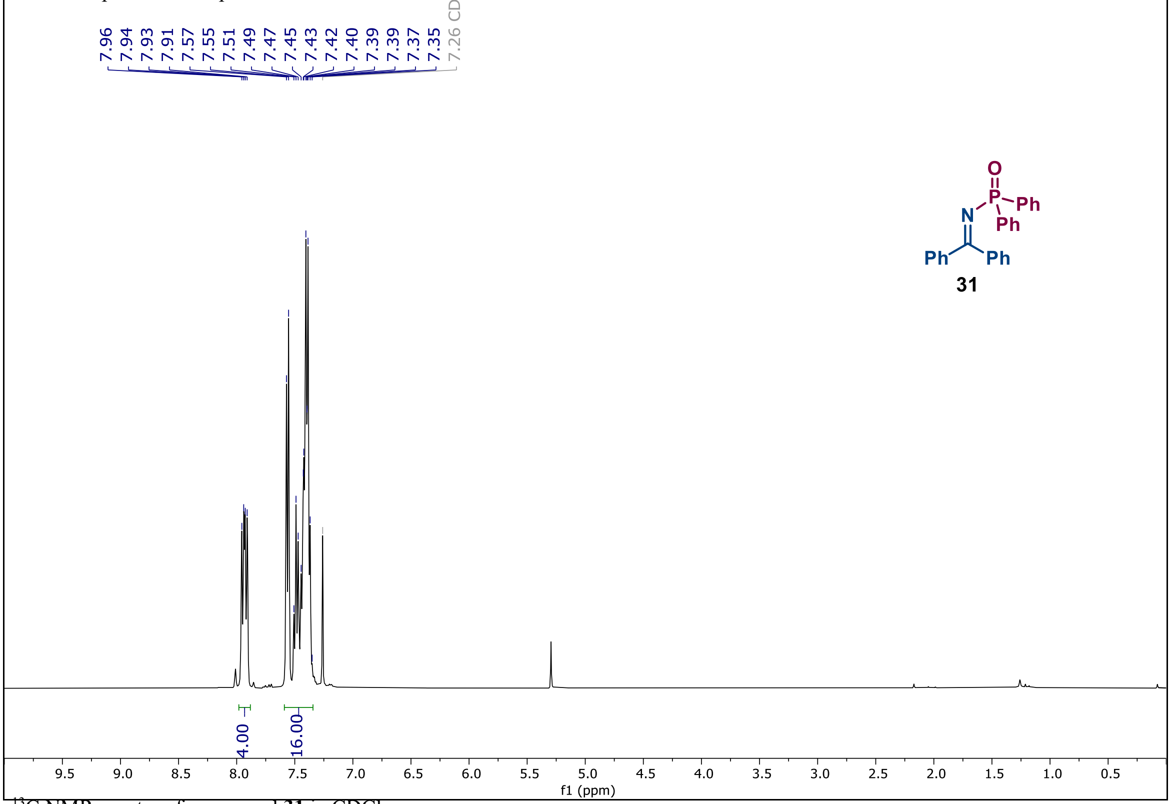

^{13}C NMR spectra of compound **31** in $CDCl_3$

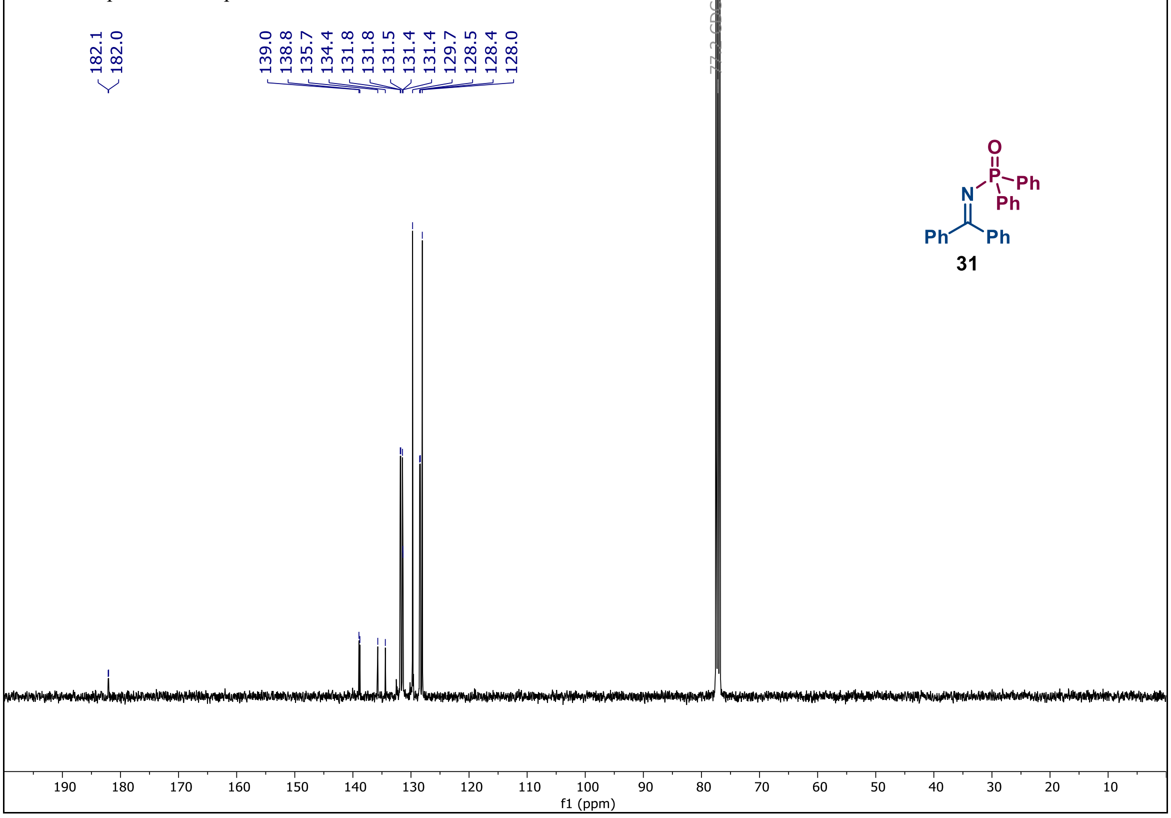

^{31}P NMR spectra of compound **31** in $CDCl_3$

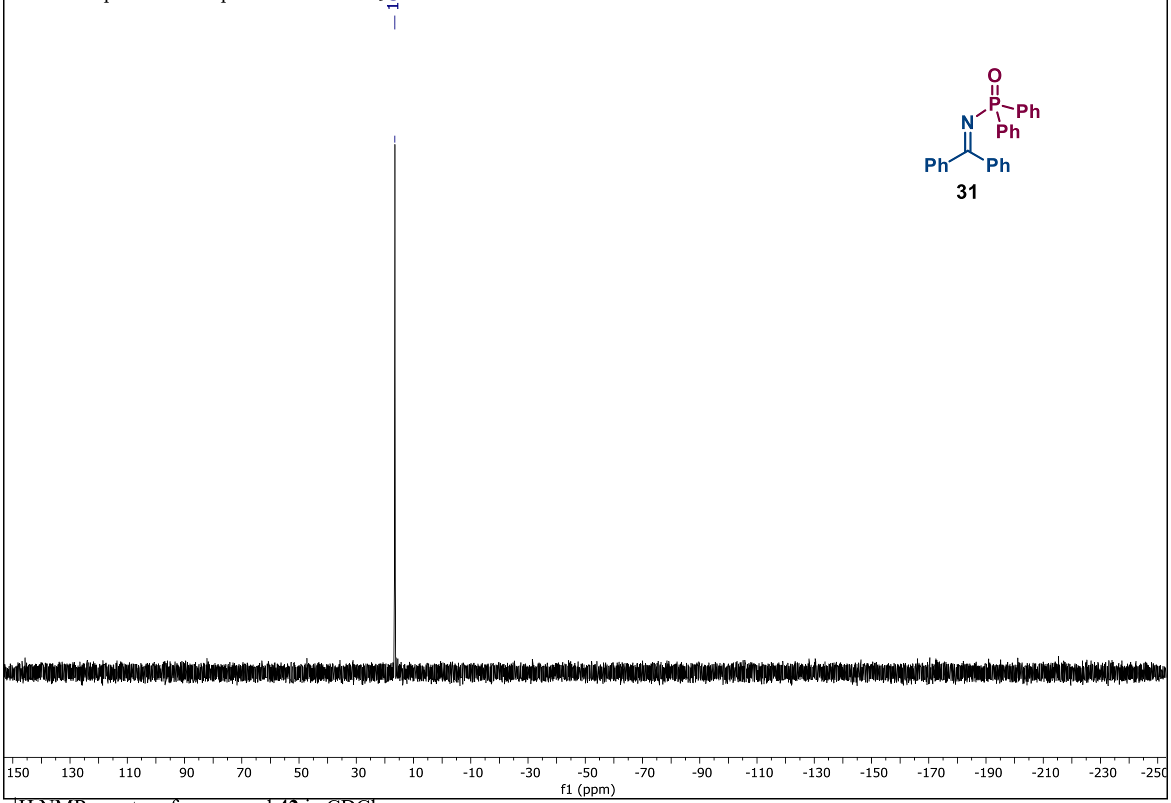

^{1}H NMR spectra of compound **42** in $CDCl_3$

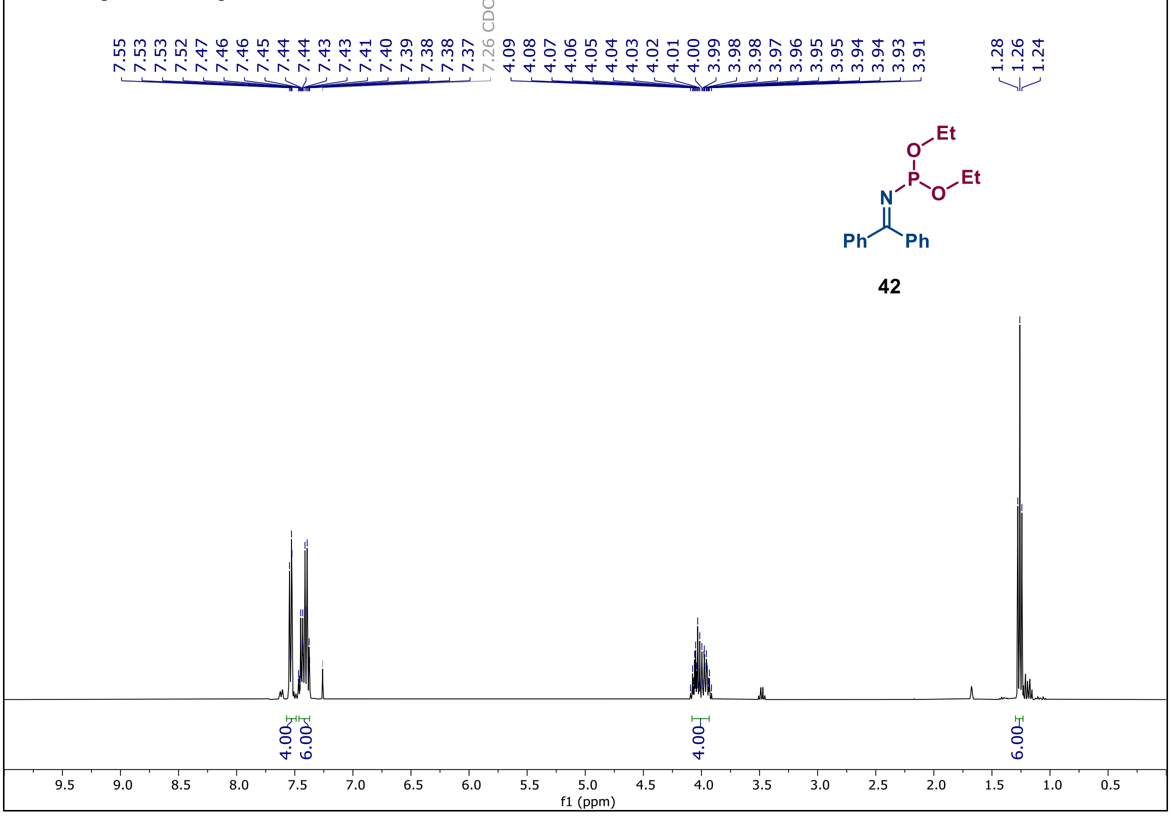

^{13}C NMR spectra of compound **42** in $CDCl_3$

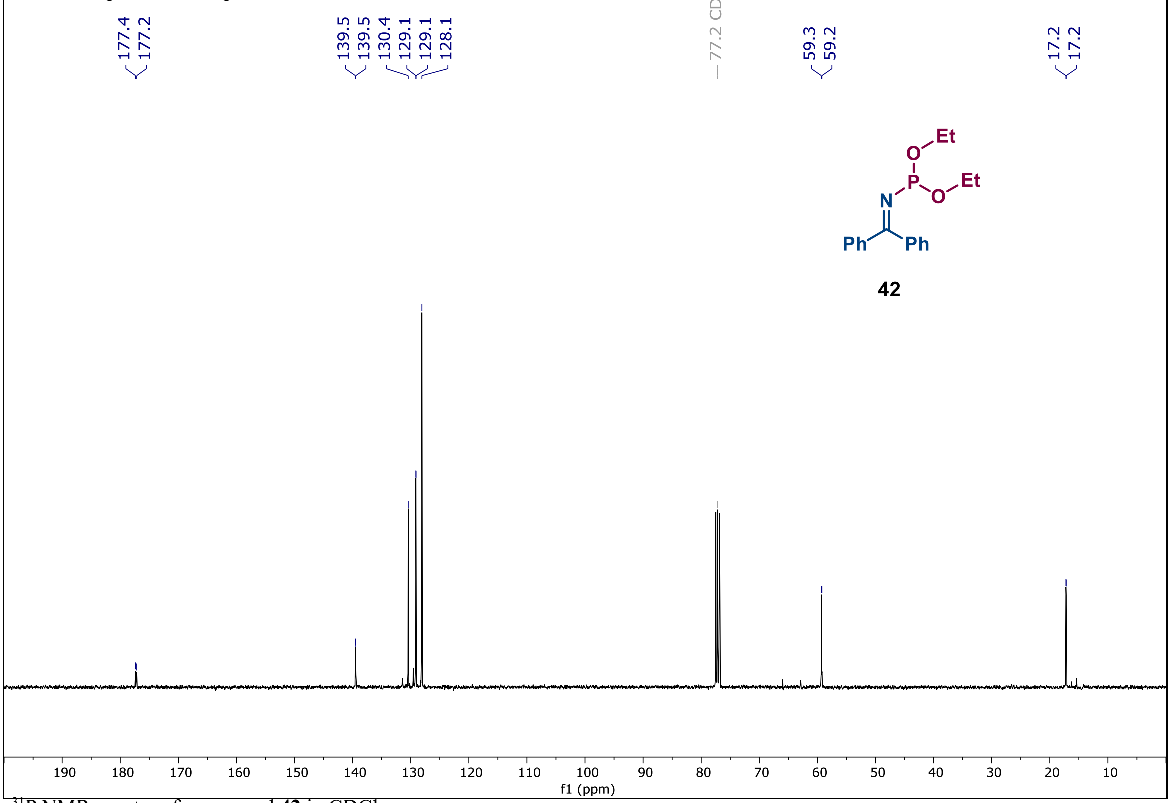

^{31}P NMR spectra of compound **42** in $CDCl_3$

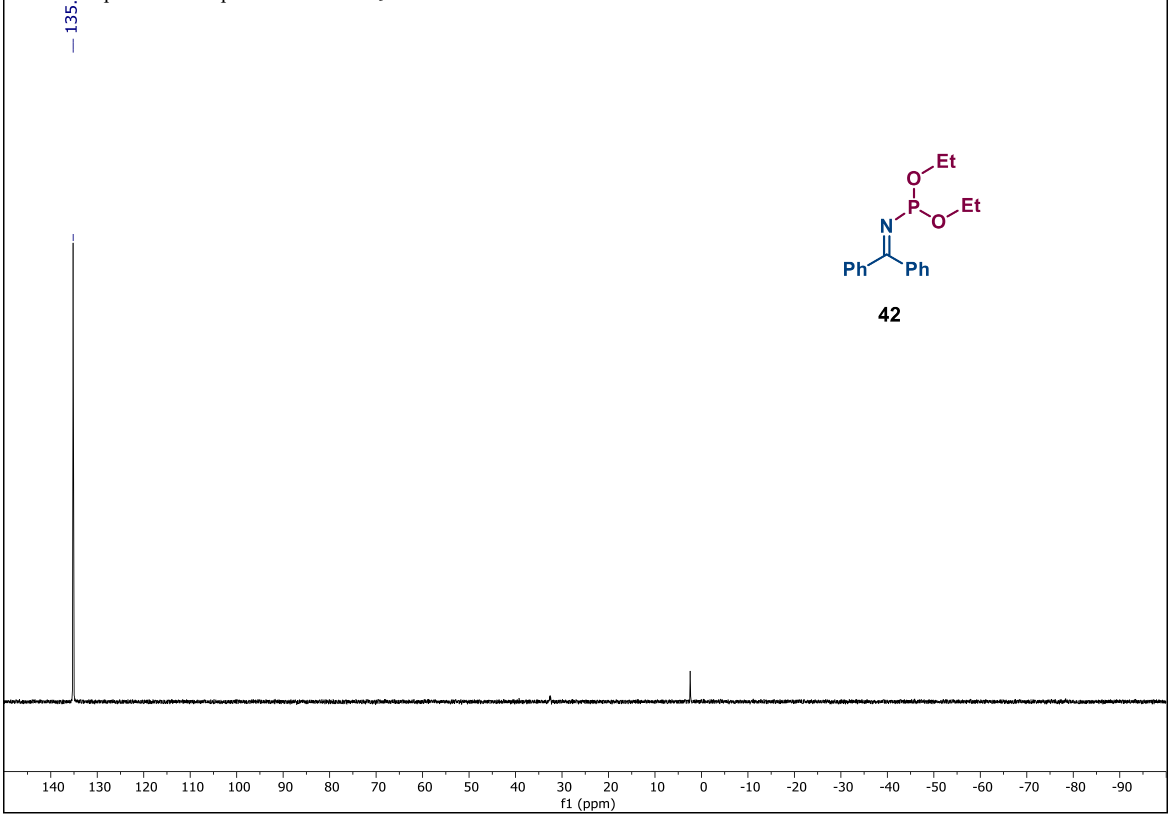

^{1}H NMR spectra of compound **S7** in $CDCl_3$

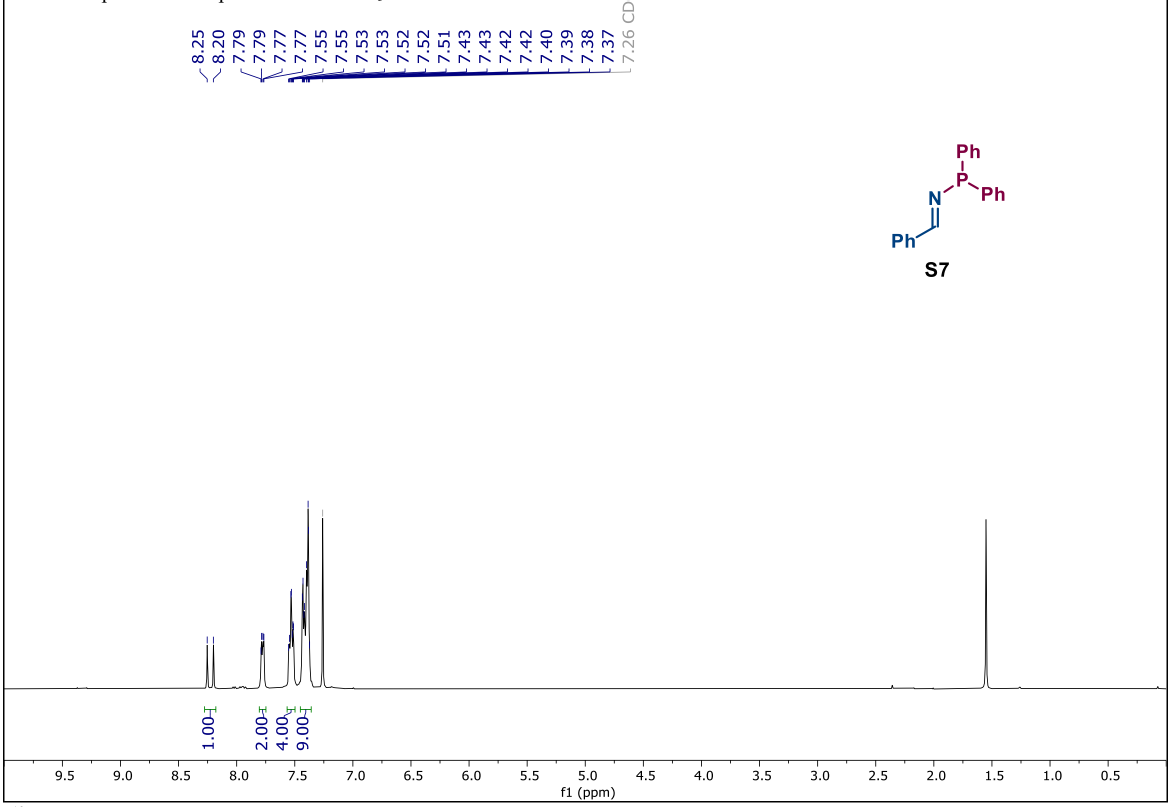

^{13}C NMR spectra of compound **S7** in $CDCl_3$

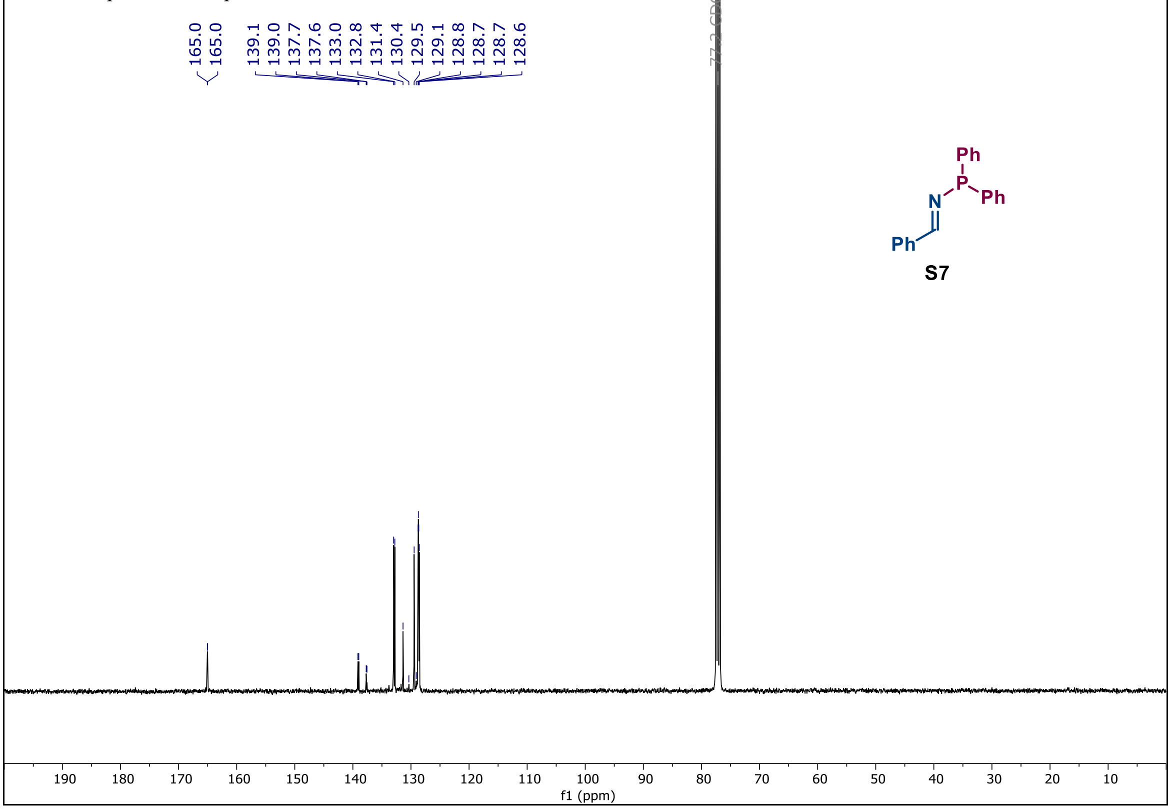

^{31}P NMR spectra of compound **S7** in $CDCl_3$

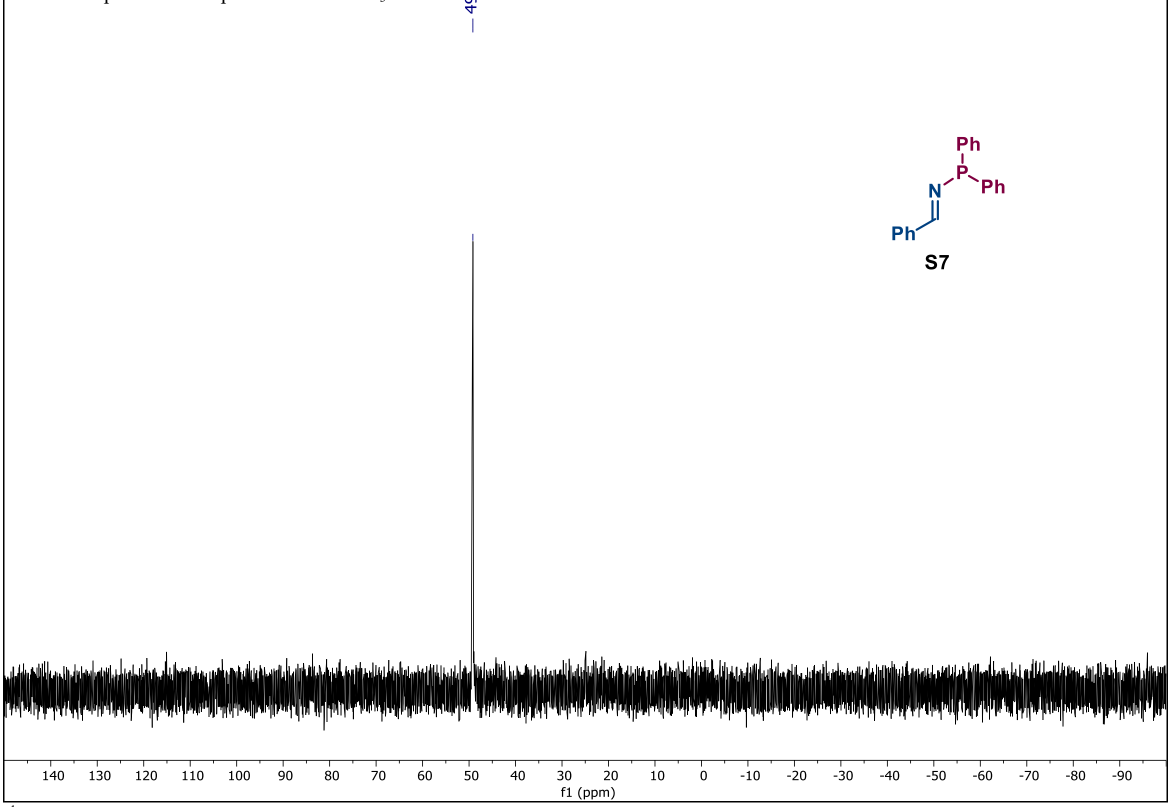

^{1}H NMR spectra of compound **S10** in $CDCl_3$

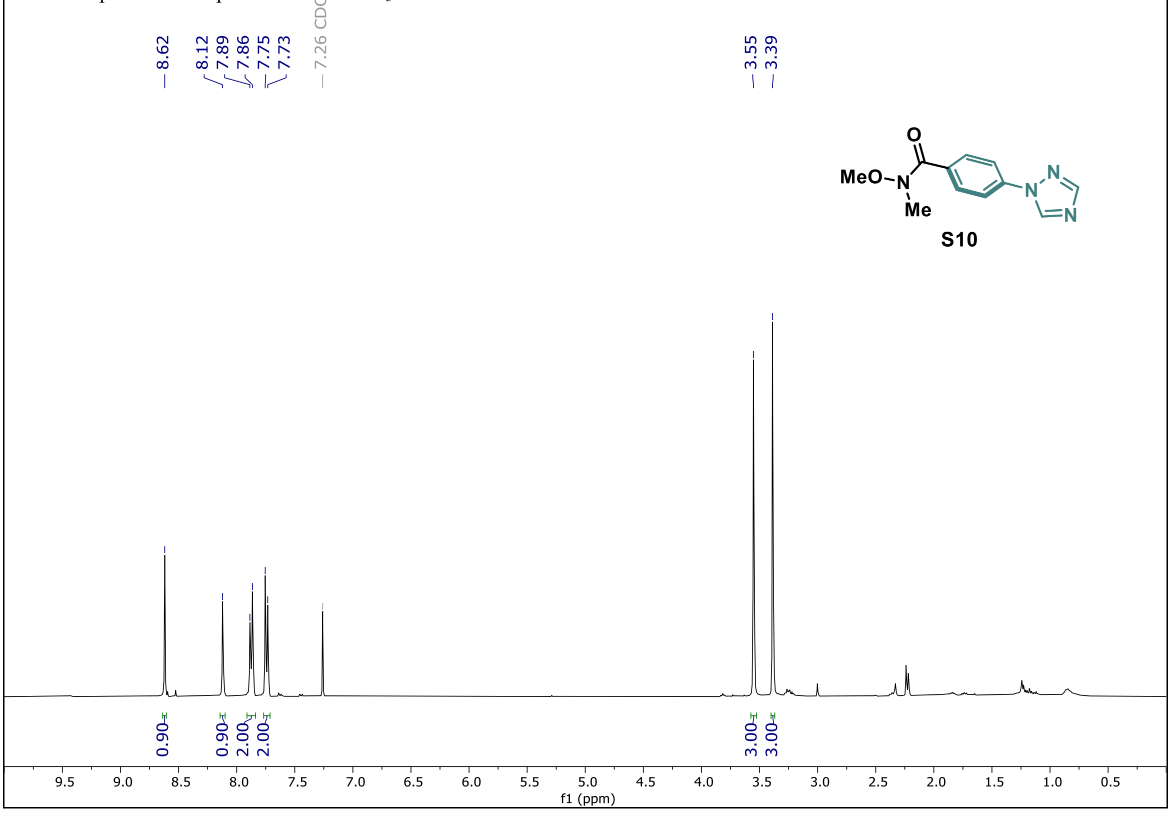

^{13}C NMR spectra of compound **S10** in $CDCl_3$

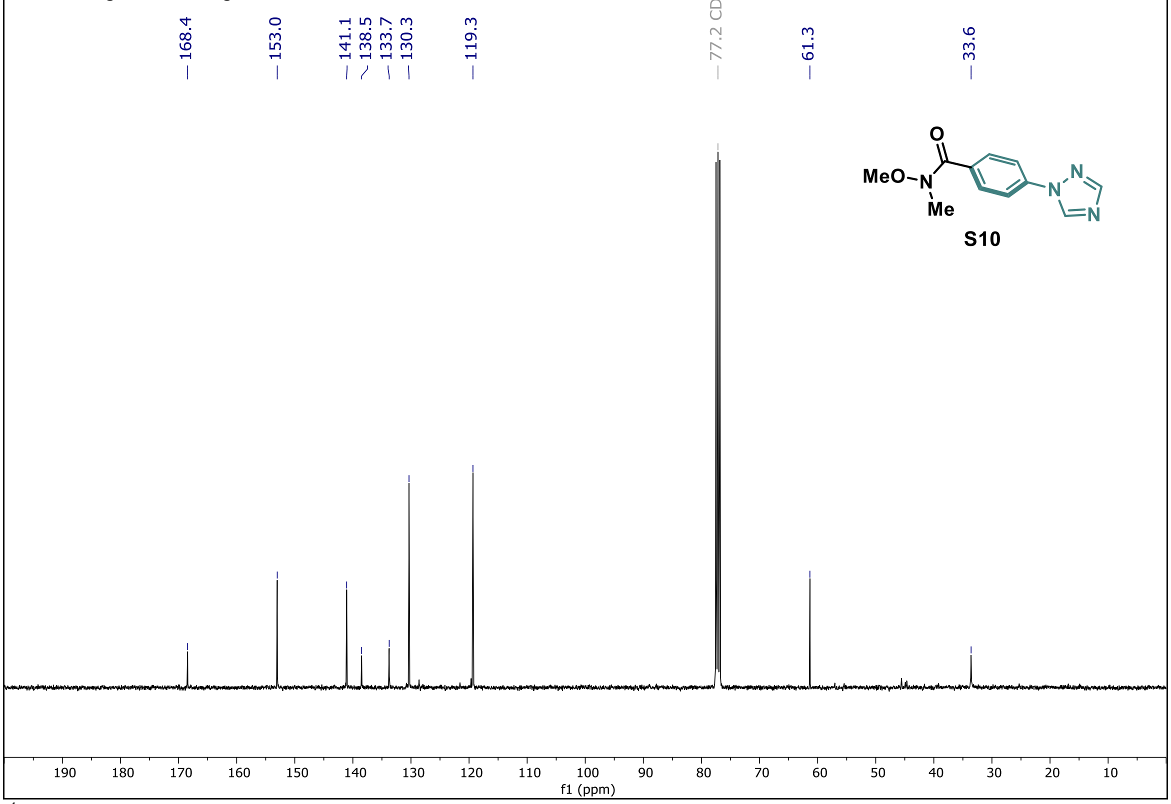

^{1}H NMR spectra of compound **S11** in $CDCl_3$

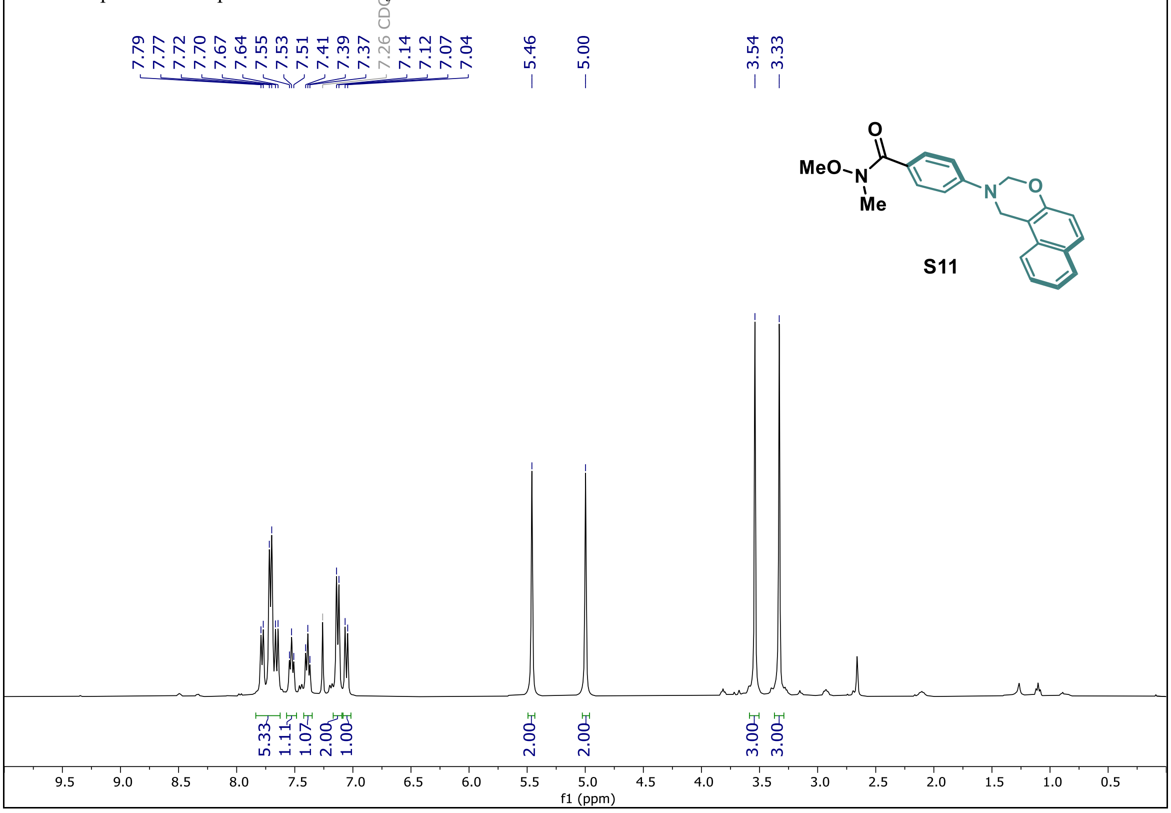

^{13}C NMR spectra of compound **S11** in $CDCl_3$

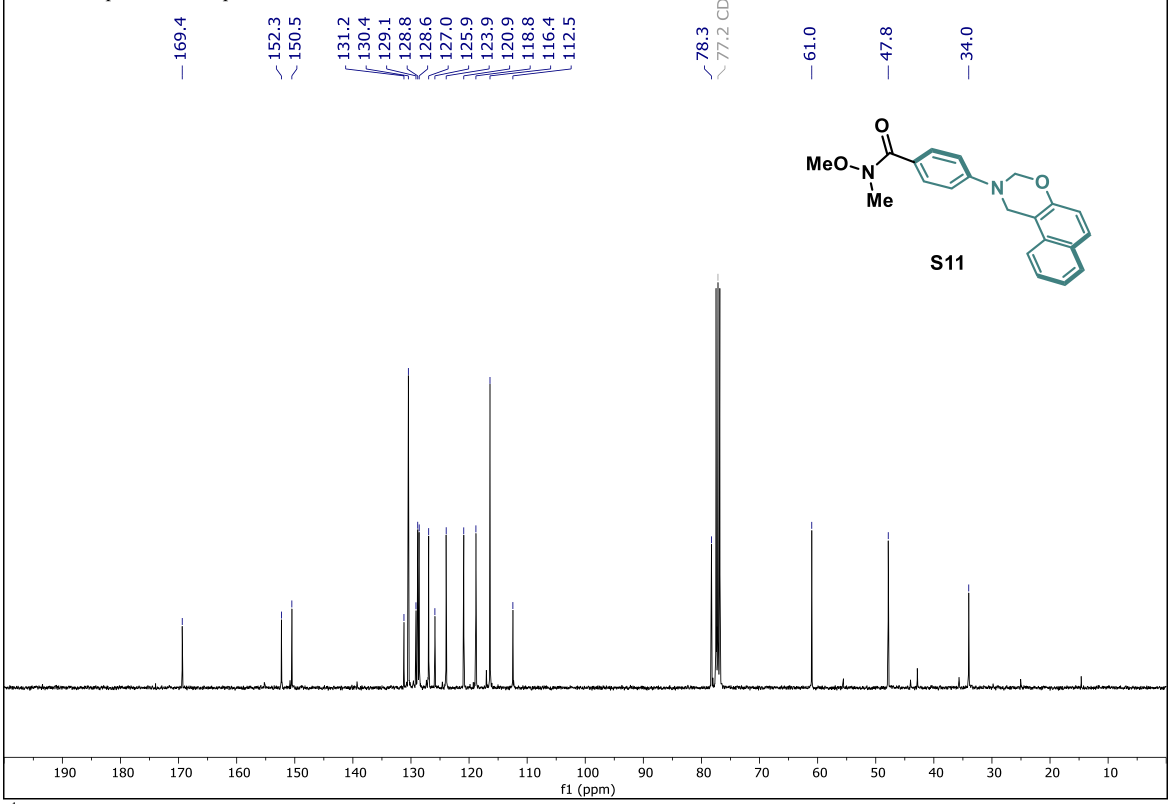

^{1}H NMR spectra of compound **S12** in $CDCl_3$

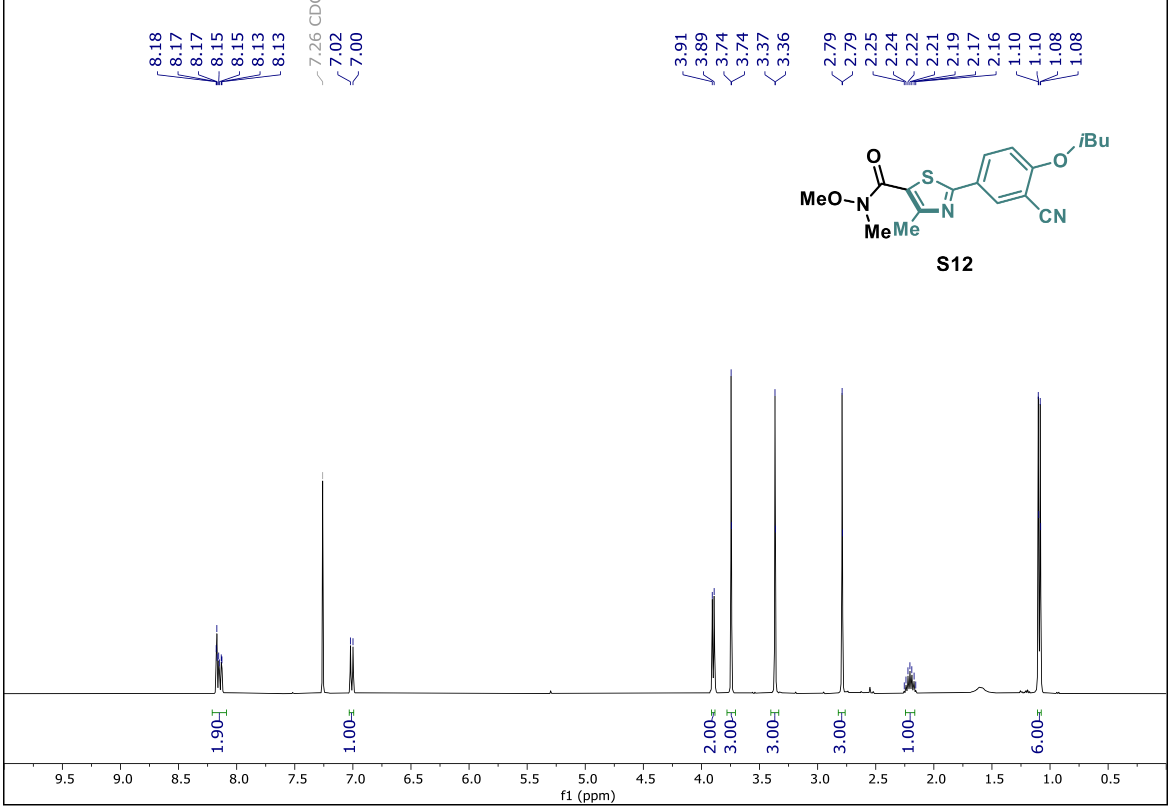

^{13}C NMR spectra of compound **S12** in $CDCl_3$

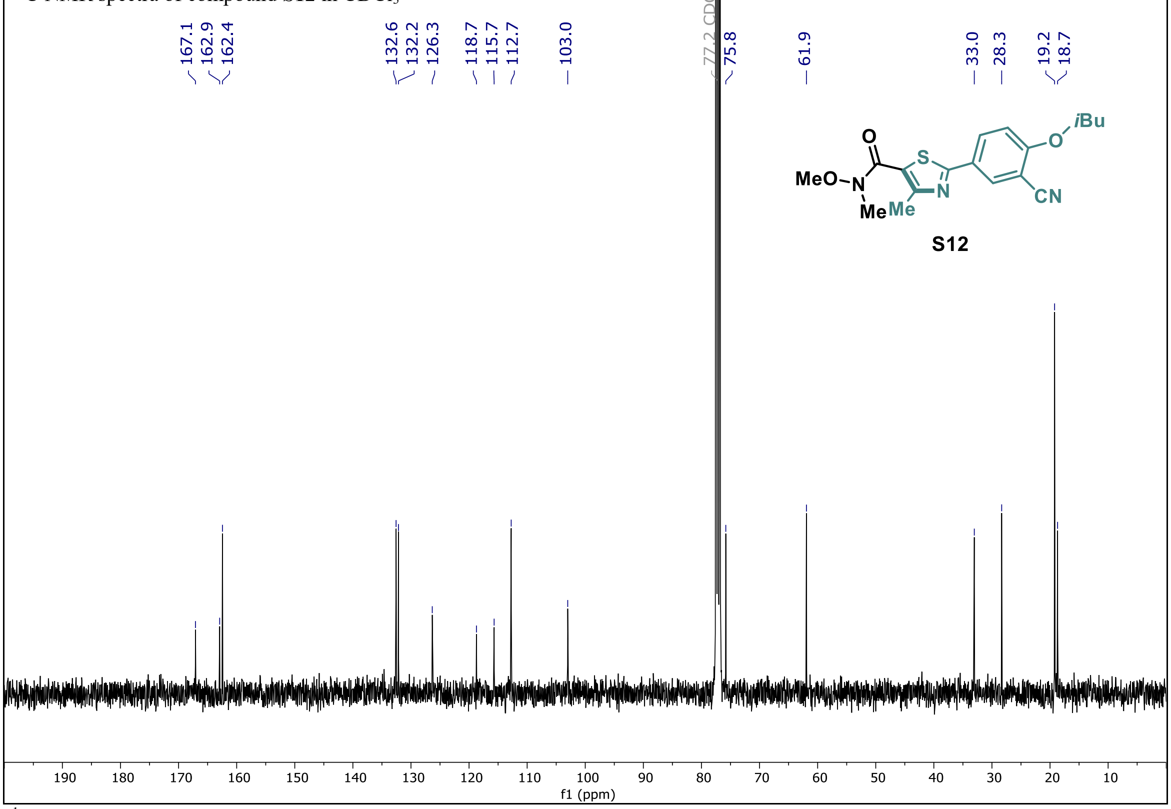

^{1}H NMR spectra of compound **S13** in $CDCl_3$

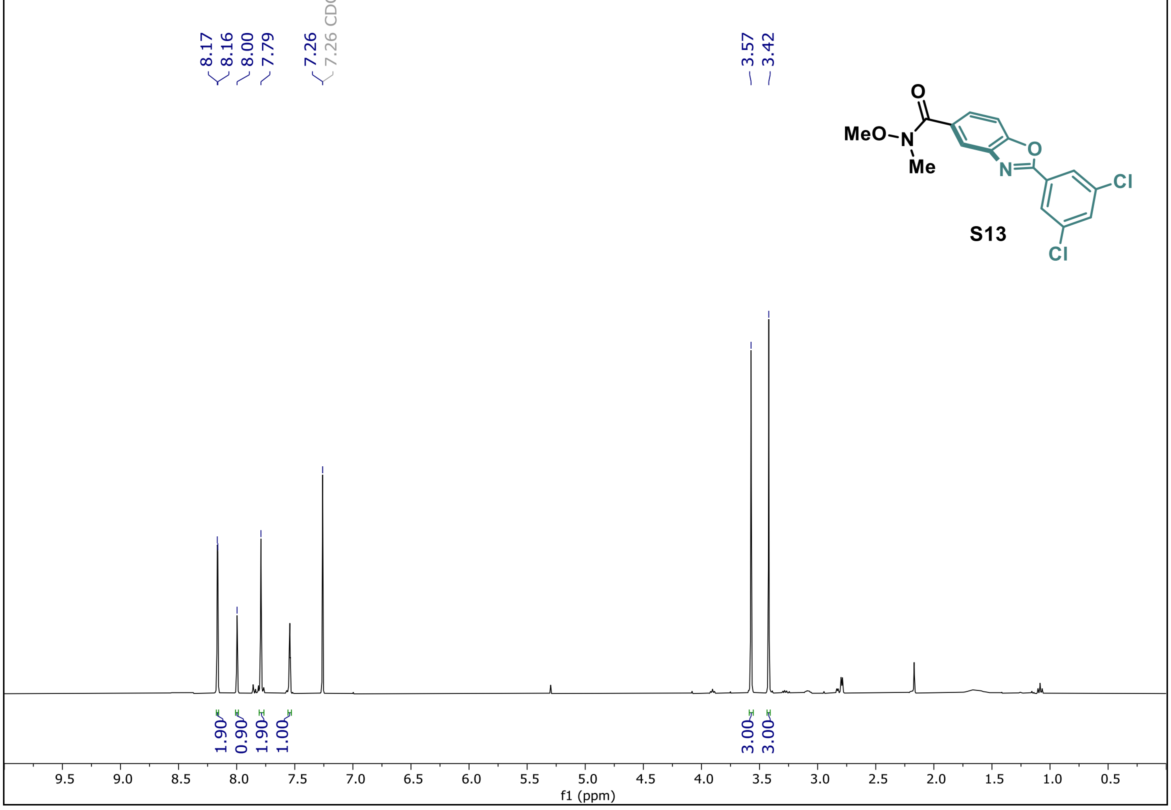

^{13}C NMR spectra of compound **S13** in $CDCl_3$

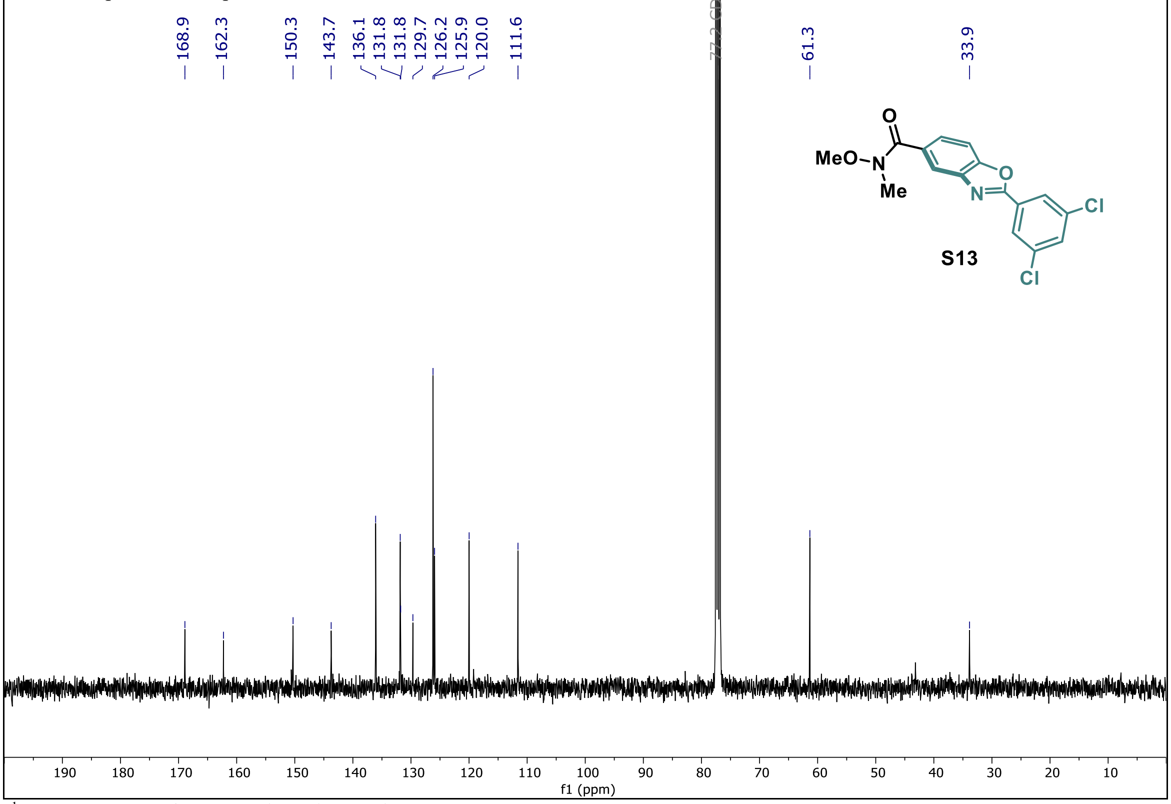

^{1}H NMR spectra of compound **S14** in $CDCl_3$

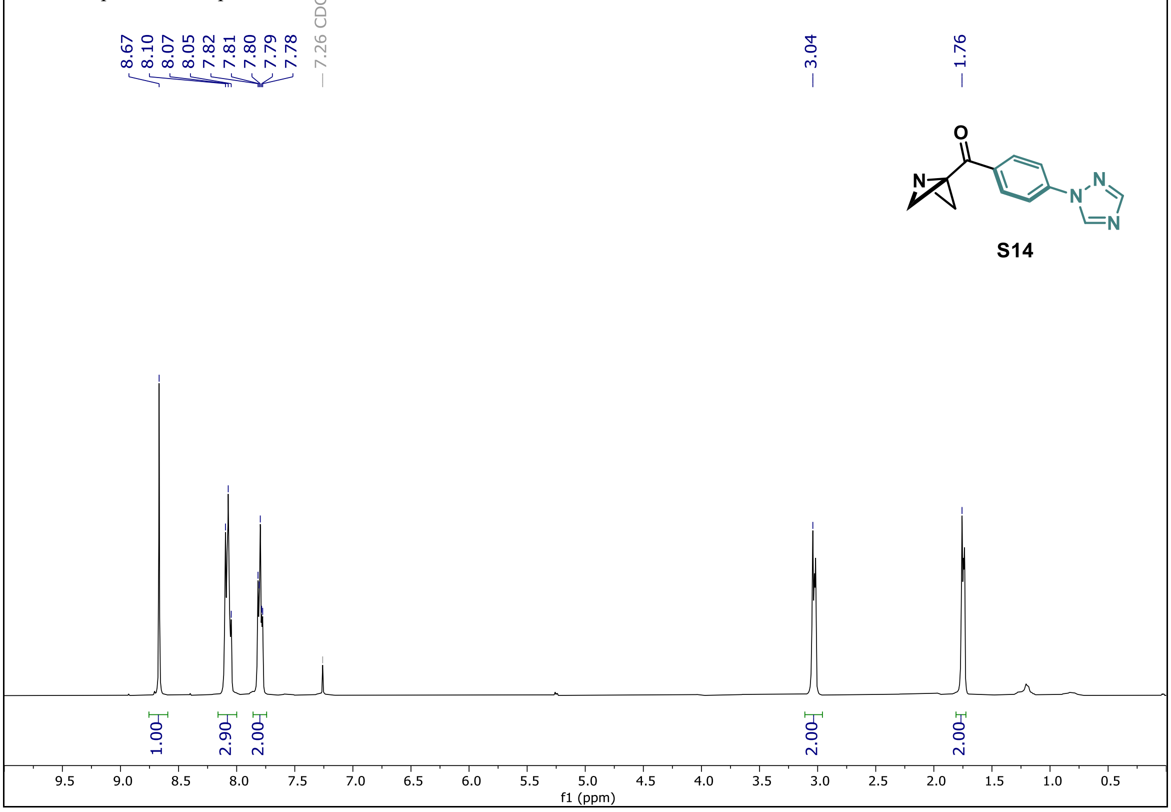

^{13}C NMR spectra of compound **S14** in $CDCl_3$

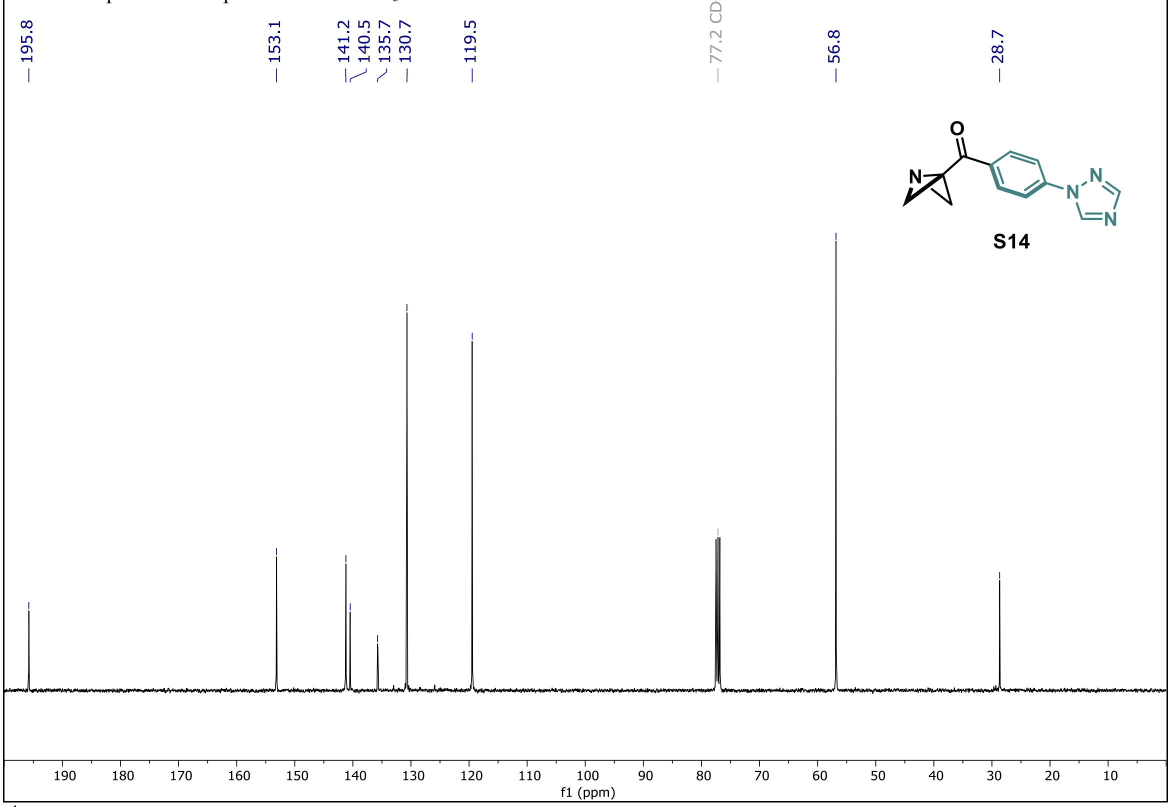

^{1}H NMR spectra of compound **S15** in $CDCl_3$

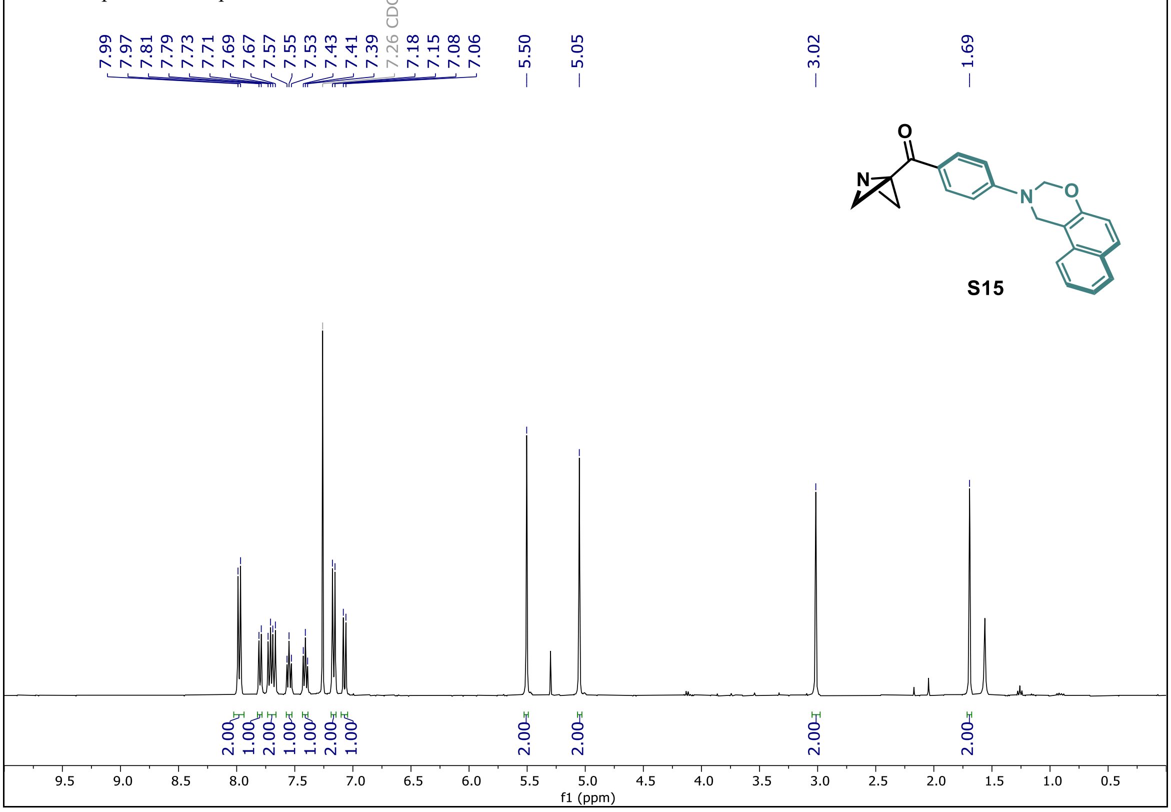

^{13}C NMR spectra of compound **S15** in CDCl$_3$

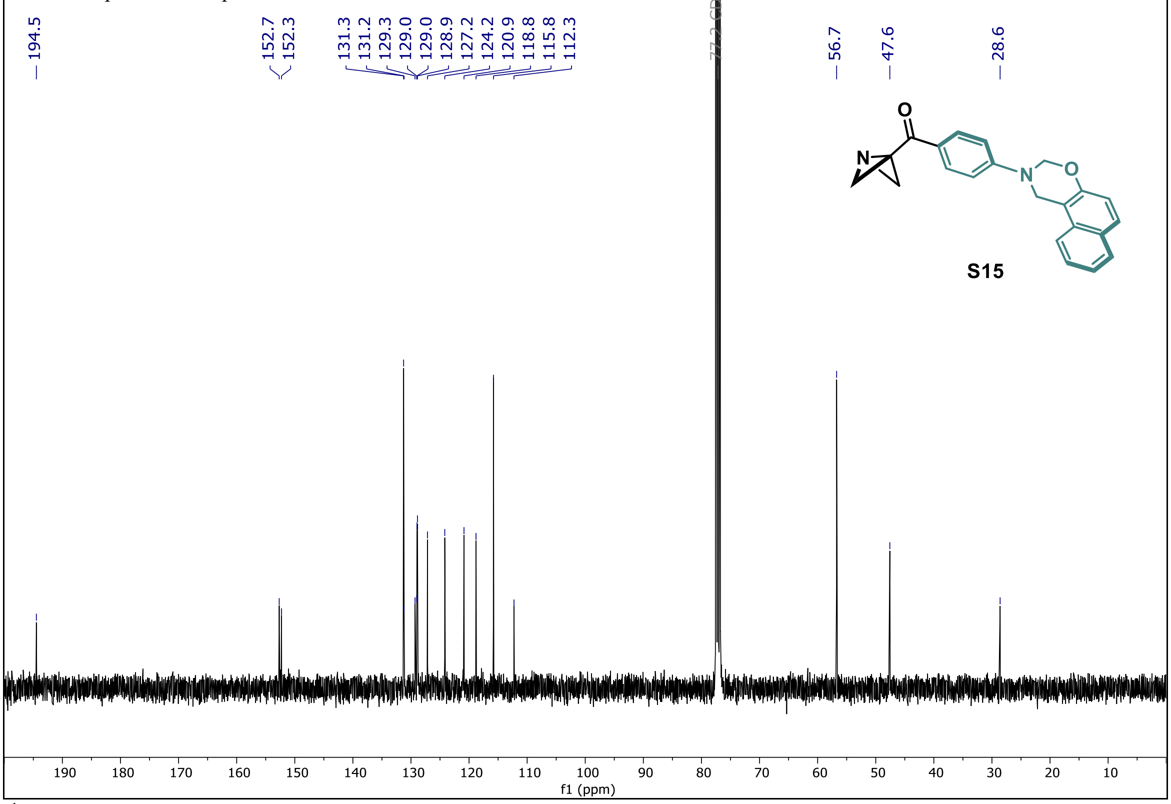

^{1}H NMR spectra of compound **S16** in CDCl$_3$

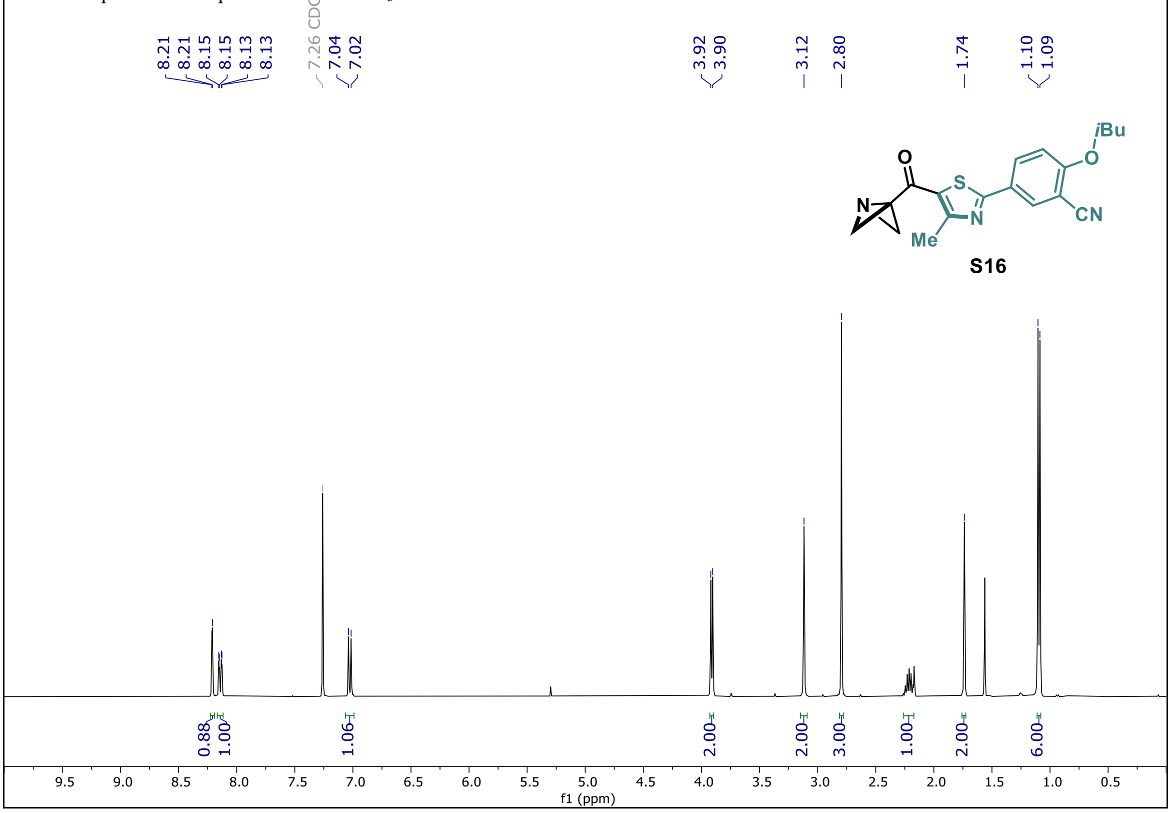

^{13}C NMR spectra of compound **S16** in $CDCl_3$

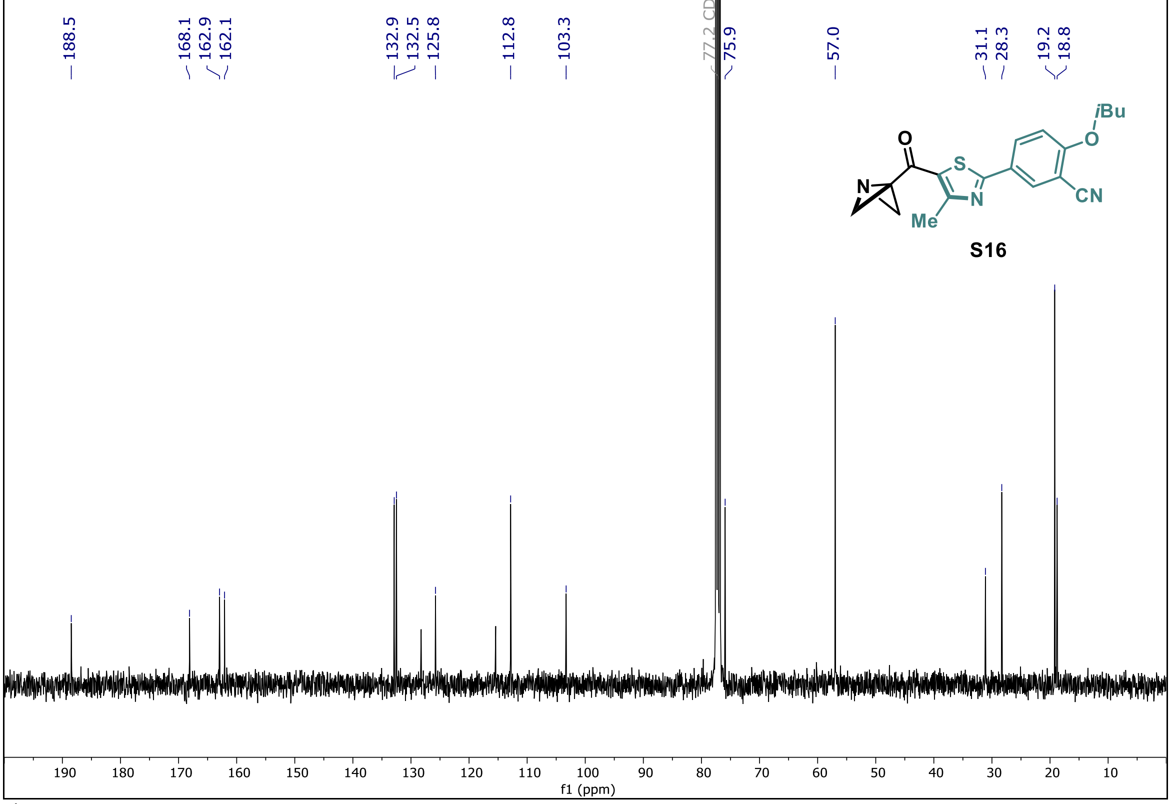

^{1}H NMR spectra of compound **S17** in $CDCl_3$

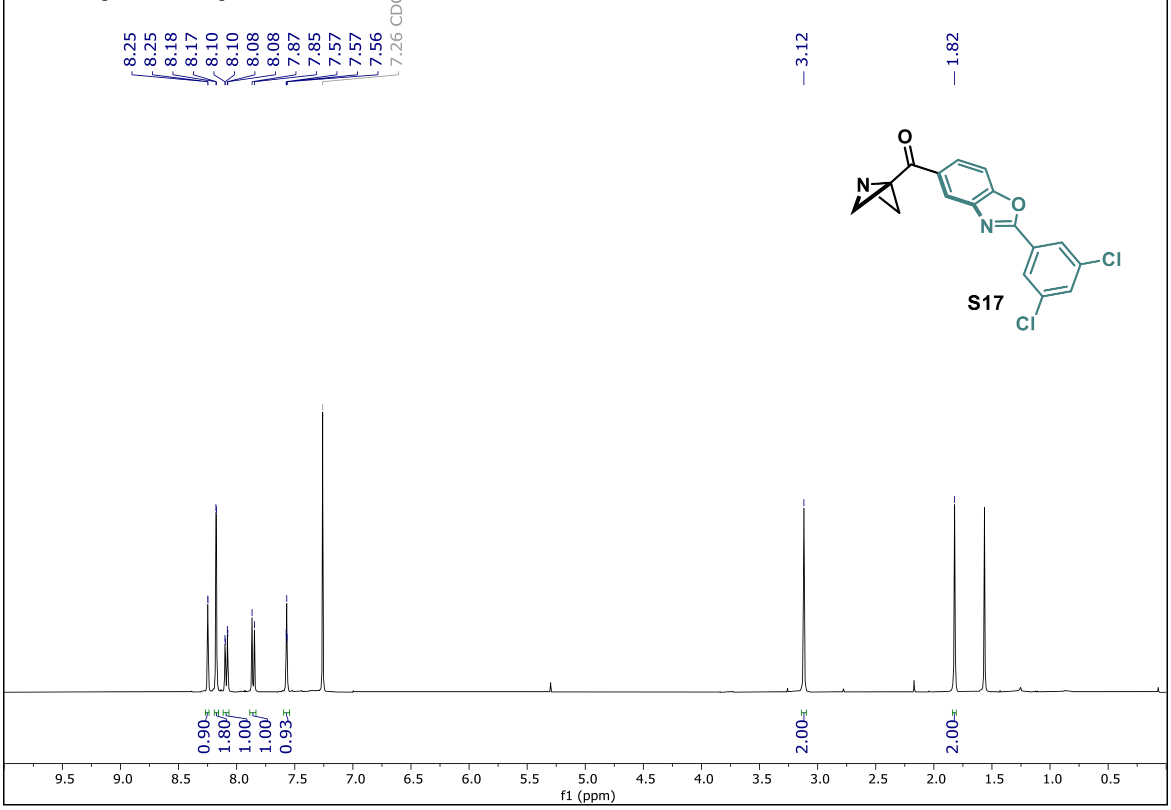

^{13}C NMR spectra of compound **S17** in $CDCl_3$

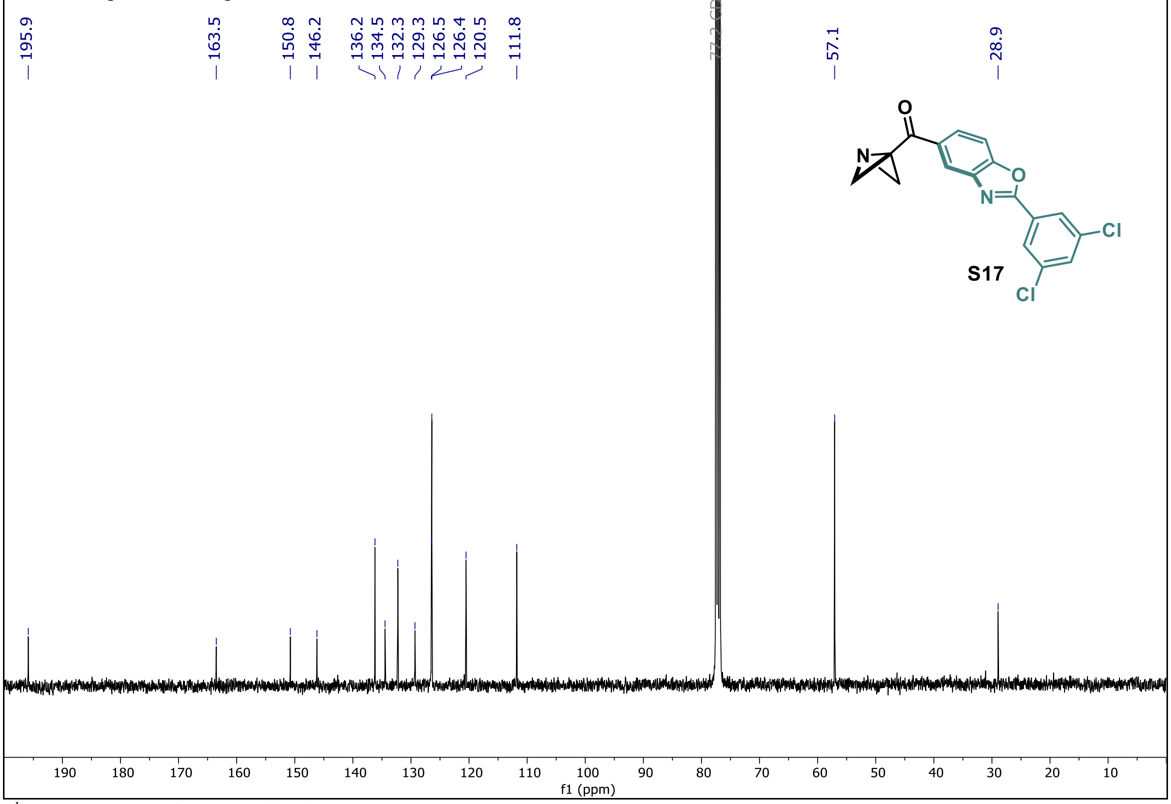

^{1}H NMR spectra of compound **34** in $CDCl_3$

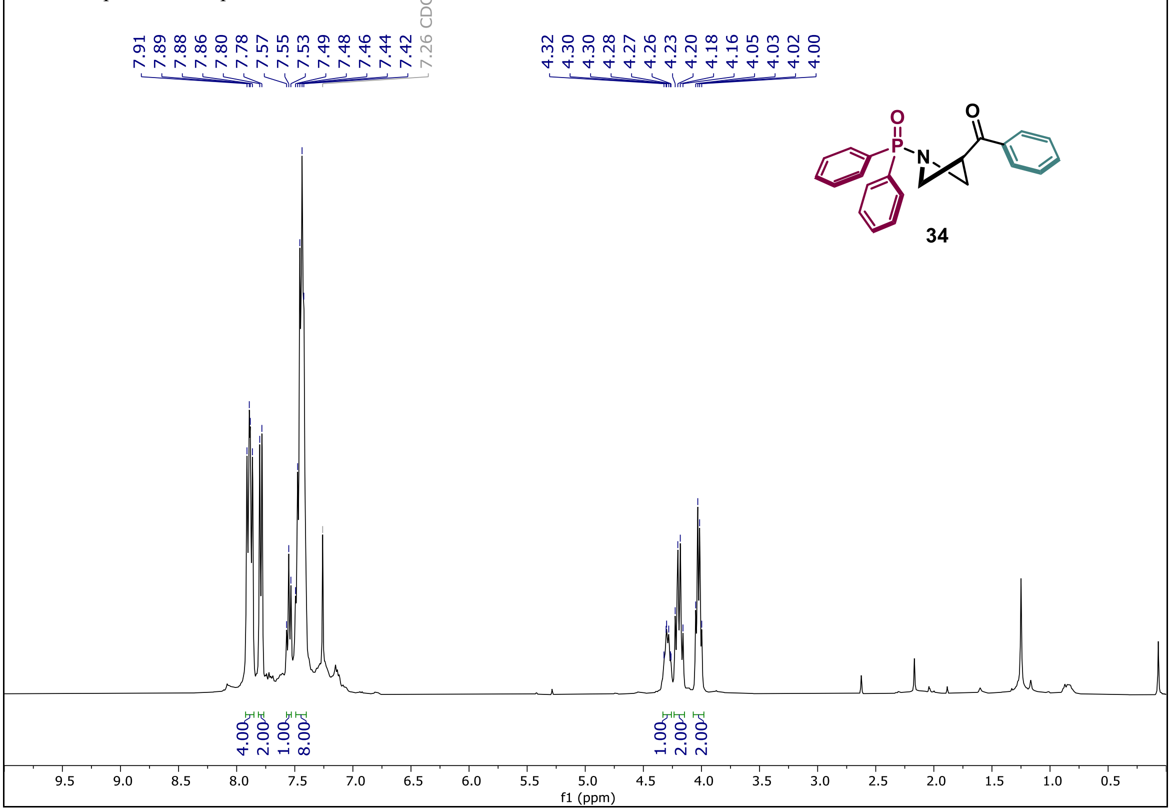

^{1}H NMR spectra of compound **34** in $CDCl_3$

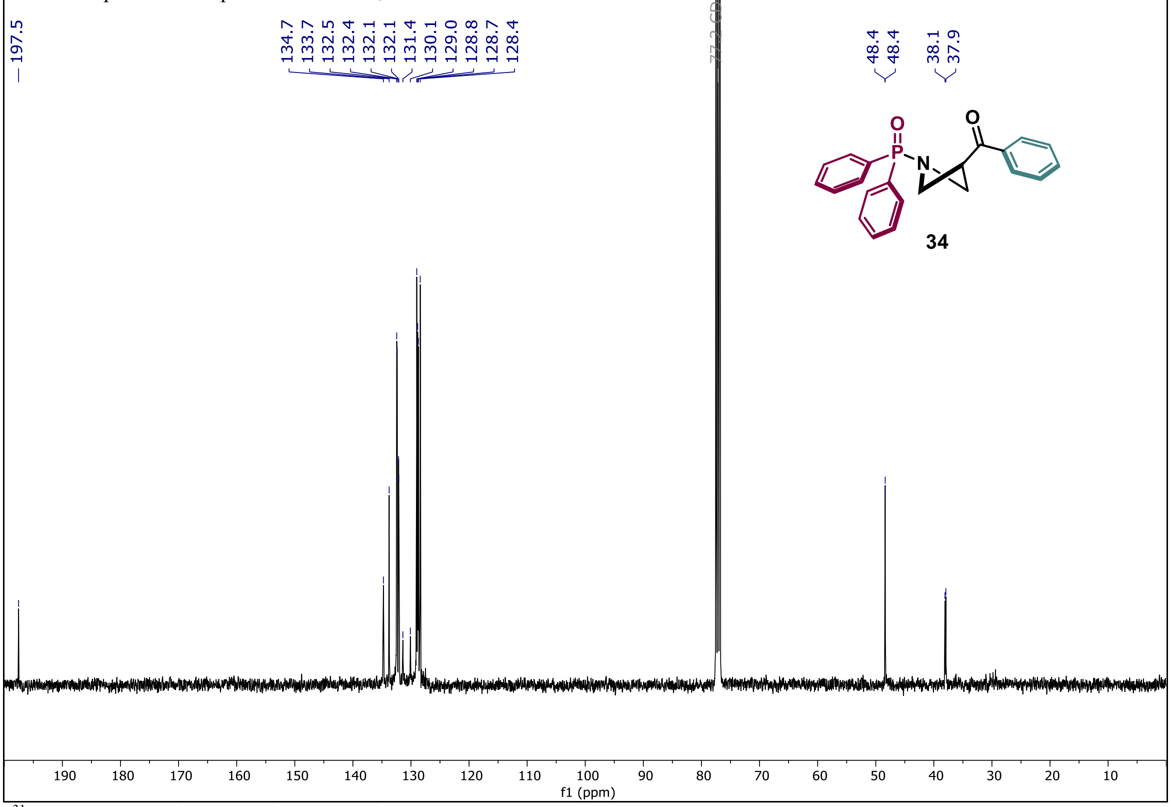

^{31}P NMR spectra of compound **34** in $CDCl_3$

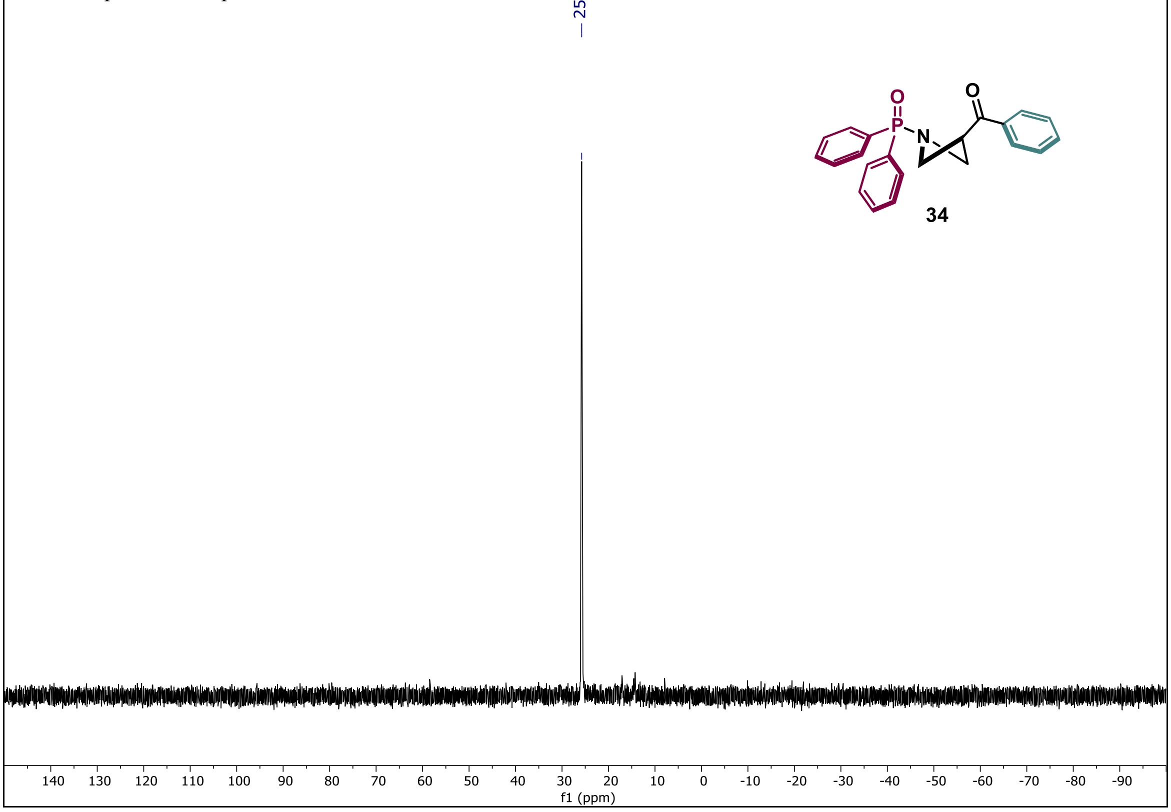

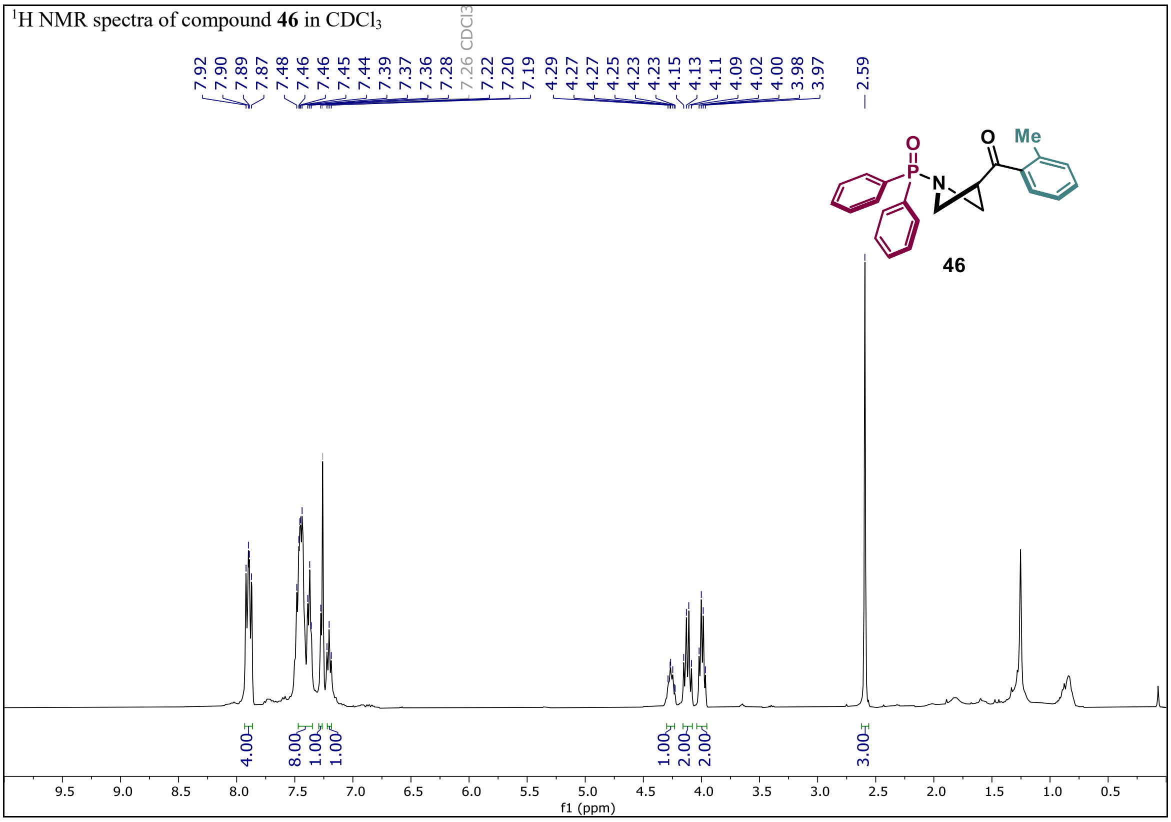

^{1}H NMR spectra of compound **46** in $CDCl_3$
7.92 7.90 7.89 7.87 7.48 7.46 7.46 7.45 7.44 7.39 7.37 7.36 7.28 7.26 CDCl3 7.22 7.20 7.19 4.29 4.27 4.27 4.25 4.23 4.23 4.15 4.13 4.11 4.09 4.02 4.00 3.98 3.97 2.59
O
P
N
O
Me
46
4.00 8.00 1.00 1.00 1.00 2.00 2.00 3.00
9.5 9.0 8.5 8.0 7.5 7.0 6.5 6.0 5.5 5.0 4.5 4.0 3.5 3.0 2.5 2.0 1.5 1.0 0.5
f1 (ppm)

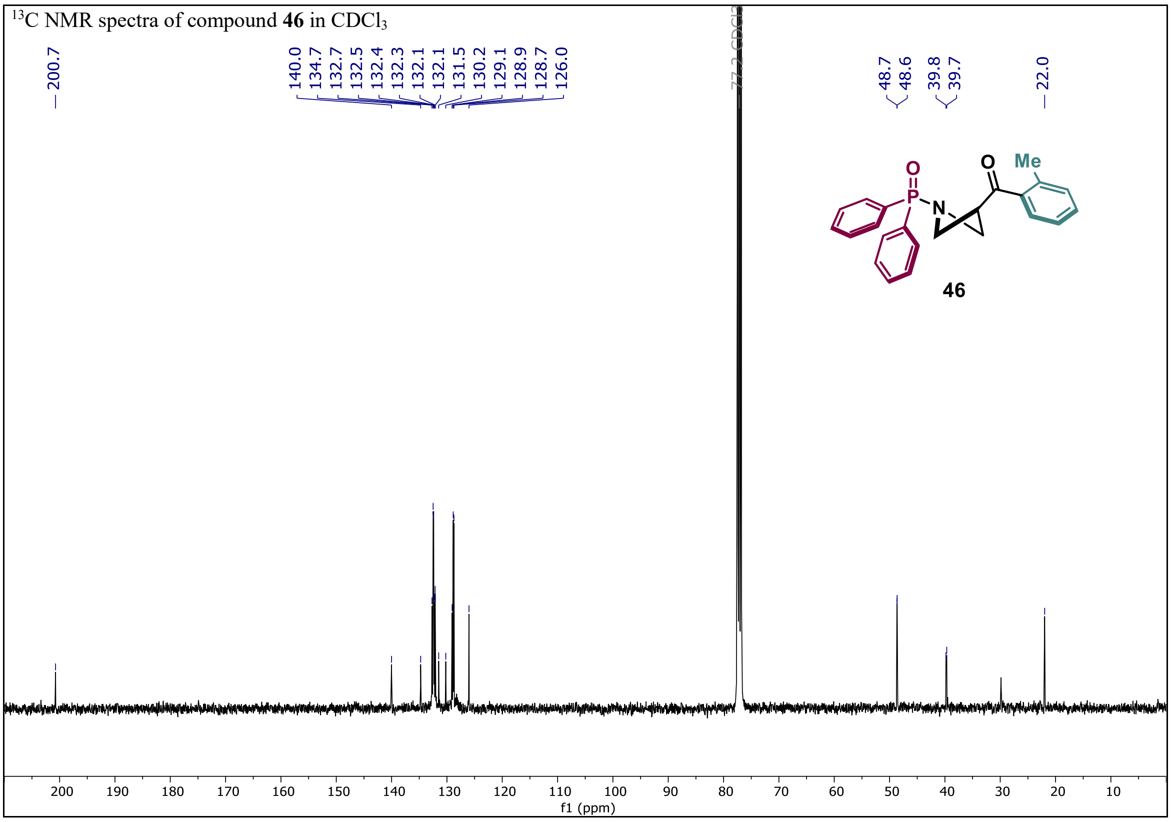

^{13}C NMR spectra of compound **46** in $CDCl_3$
200.7
140.0 134.7 132.7 132.5 132.4 132.3 132.1 132.1 131.5 130.2 129.1 128.9 128.7 126.0
77.2 CDCl3
48.7 48.6 39.8 39.7
22.0
O
P
N
O
Me
46
200 190 180 170 160 150 140 130 120 110 100 90 80 70 60 50 40 30 20 10
f1 (ppm)

^{31}P NMR spectra of compound **46** in $CDCl_3$

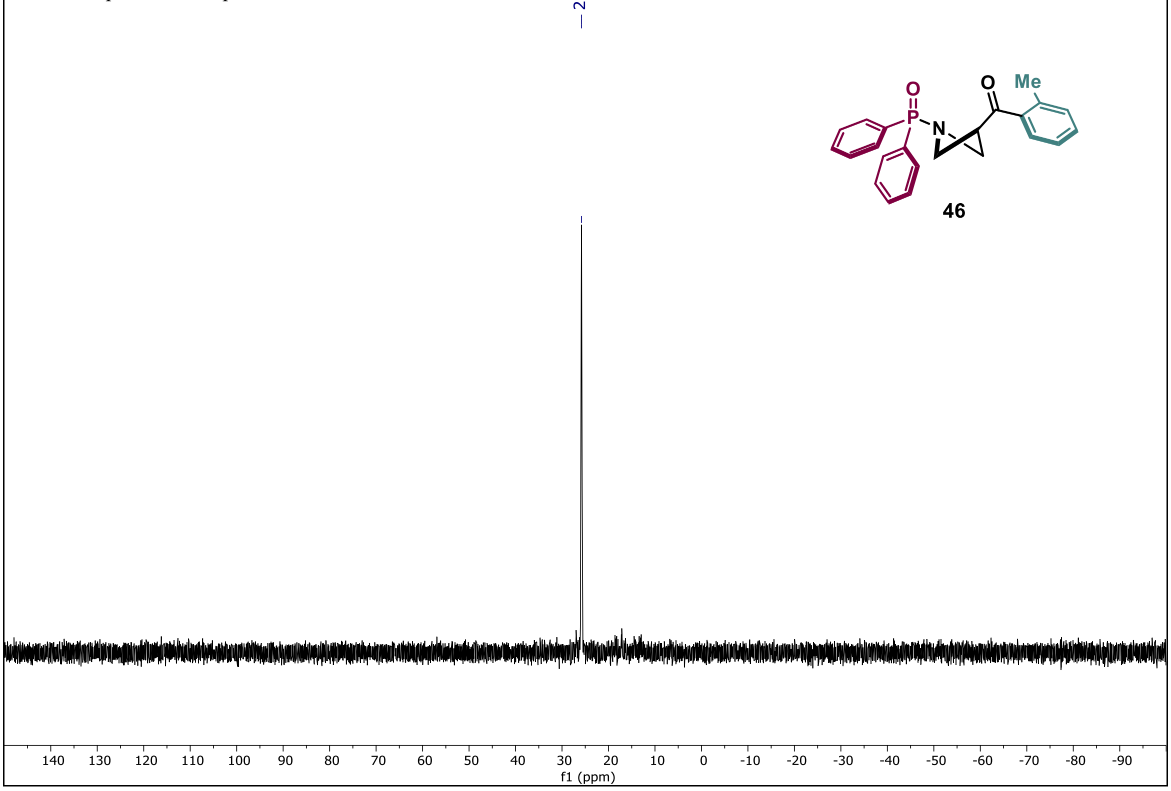

^{1}H NMR spectra of compound **47** in $CDCl_3$

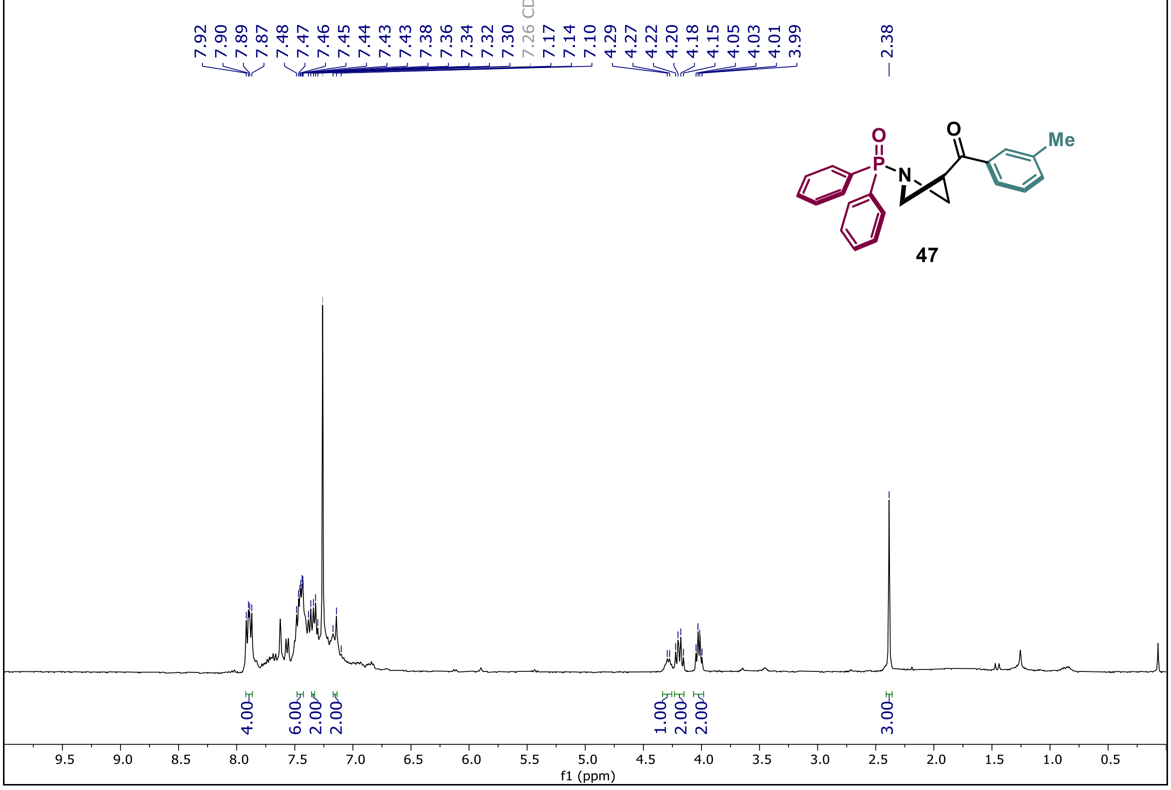

^{13}C NMR spectra of compound **47** in $CDCl_3$

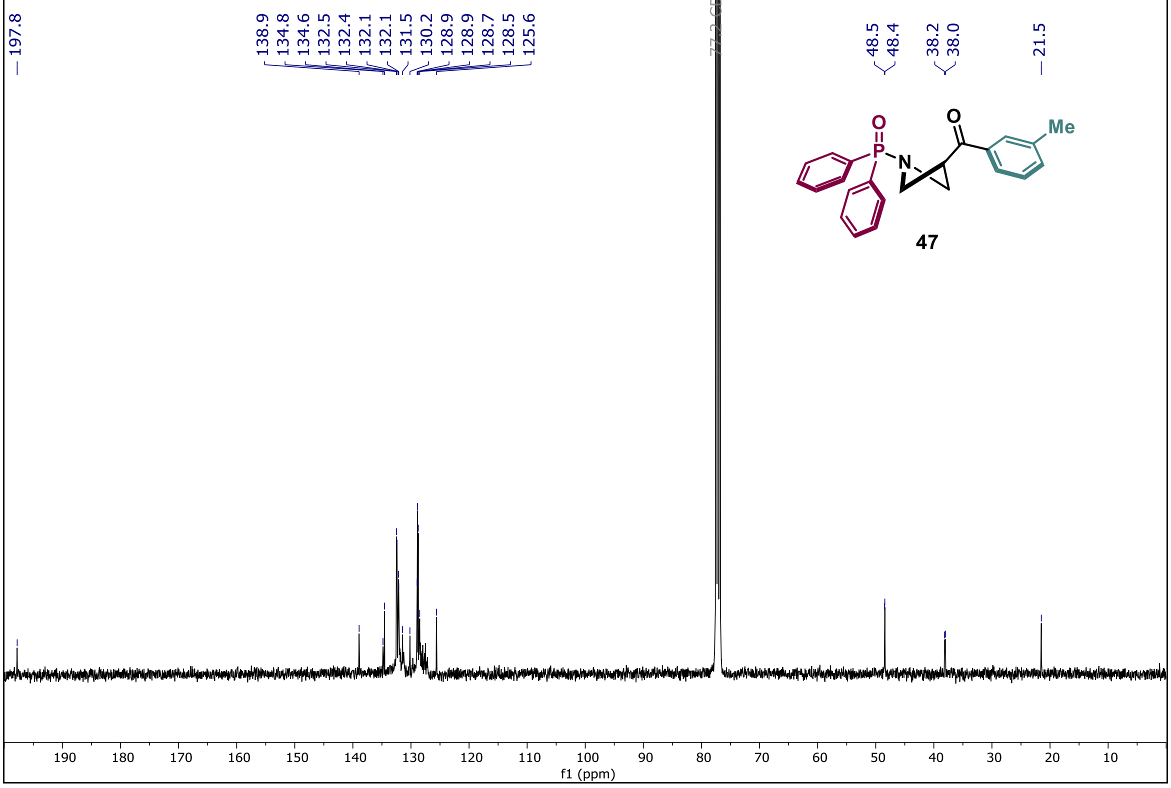

^{31}P NMR spectra of compound **47** in $CDCl_3$

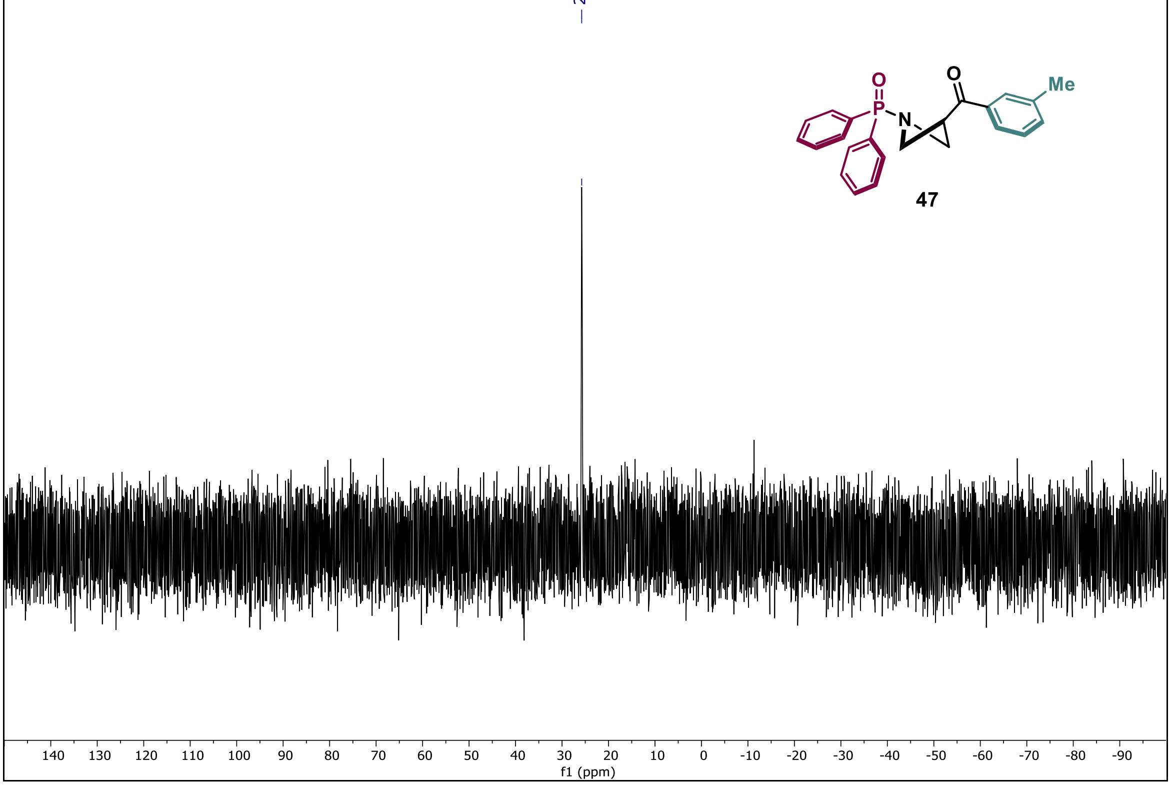

^{1}H NMR spectra of compound **48** in $CDCl_3$

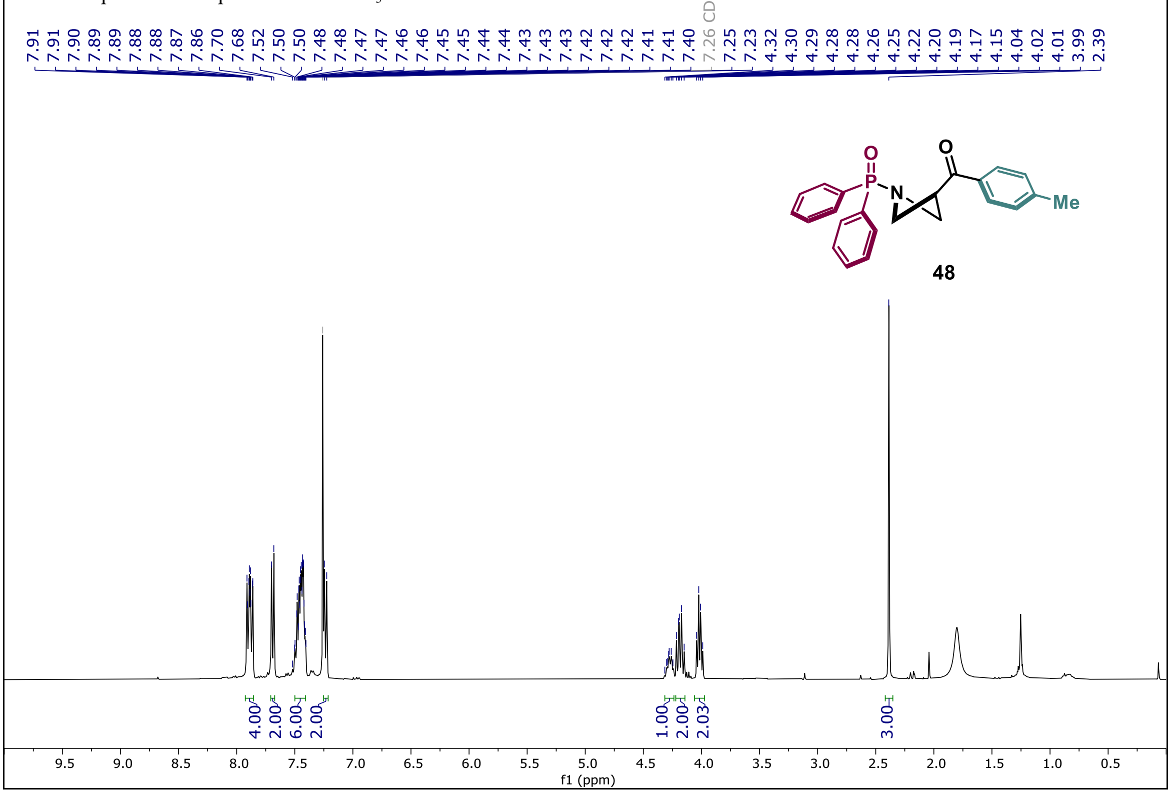

^{13}C NMR spectra of compound **48** in $CDCl_3$

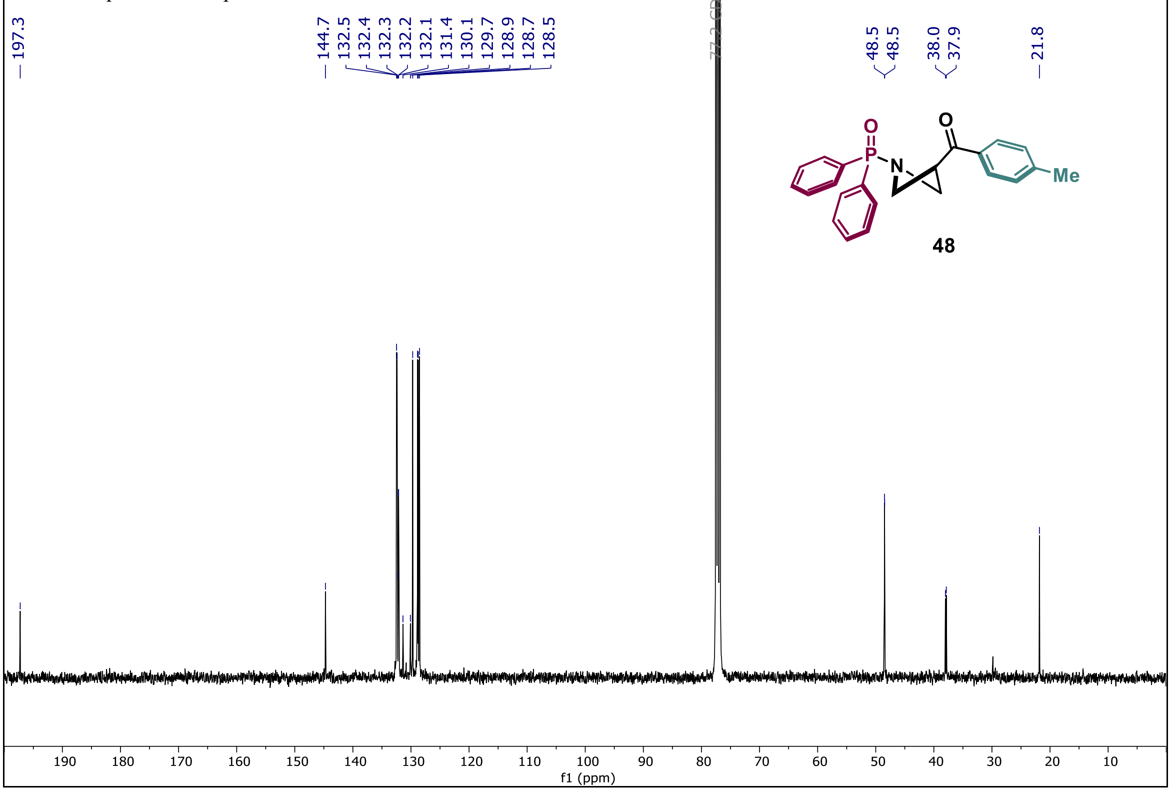

^{31}P NMR spectra of compound **48** in $CDCl_3$

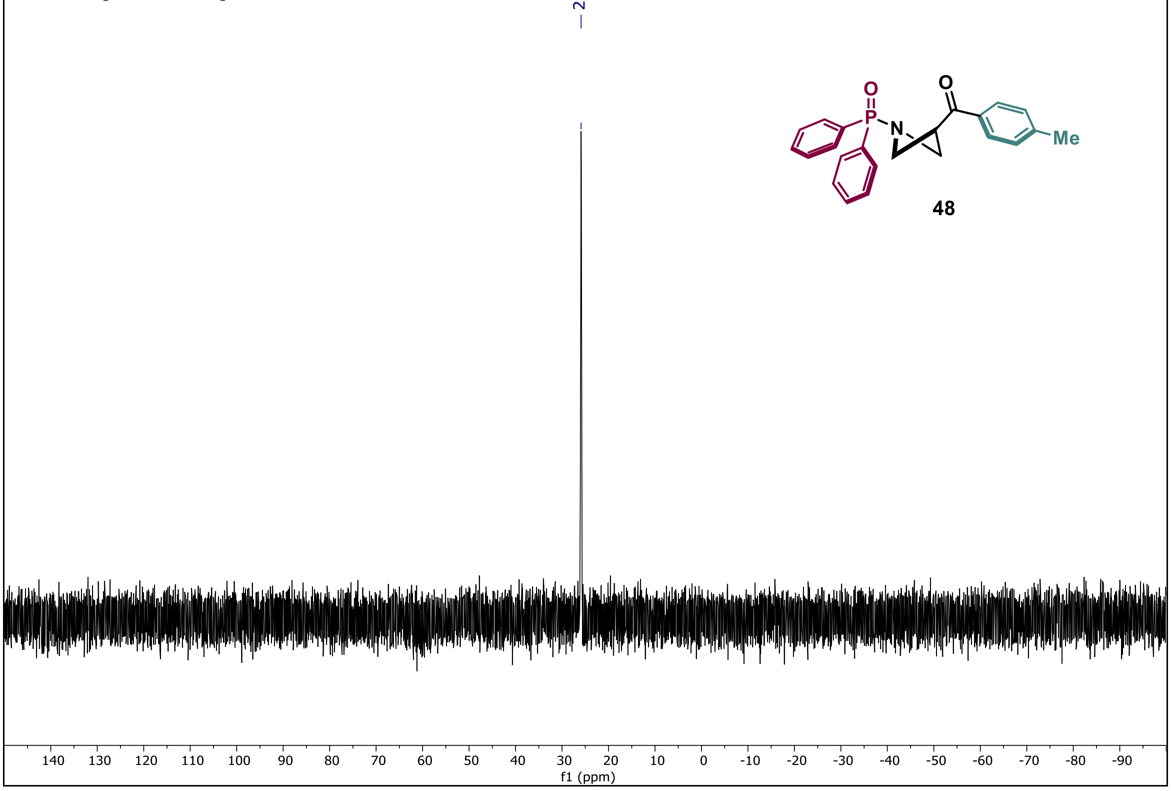

^{1}H NMR spectra of compound **49** in $CDCl_3$

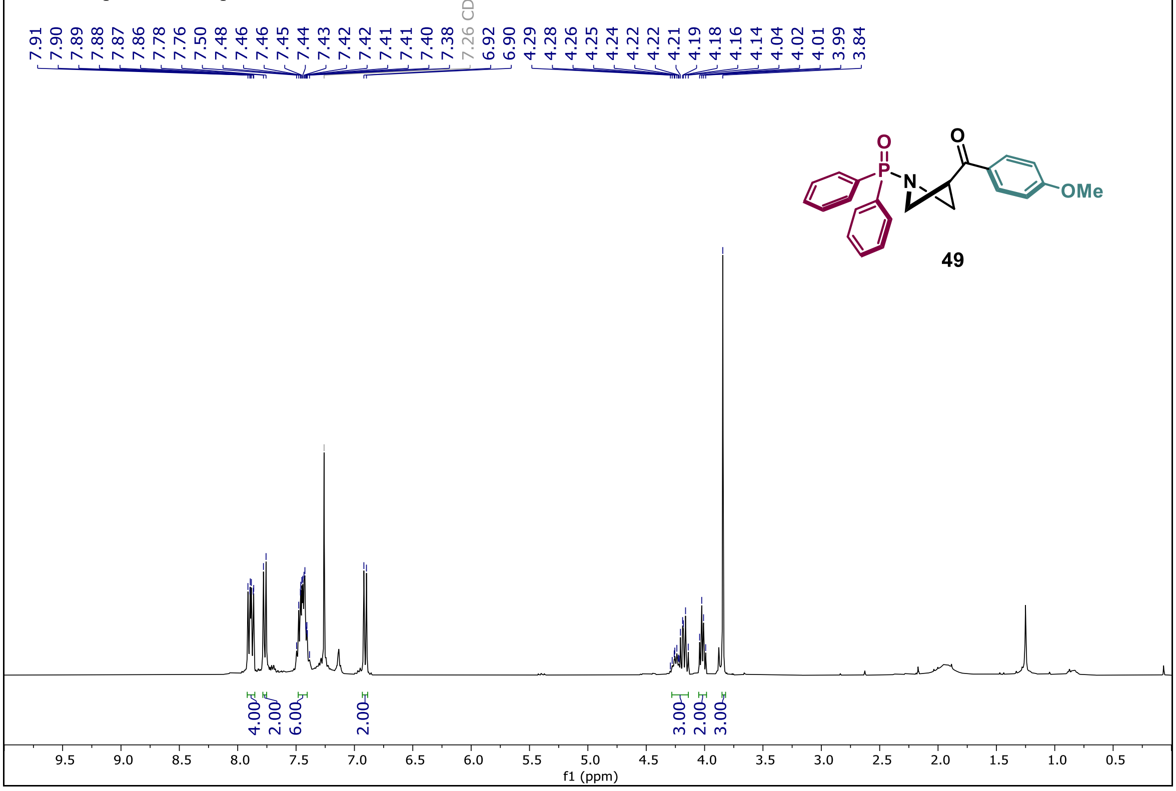

^{13}C NMR spectra of compound **49** in $CDCl_3$

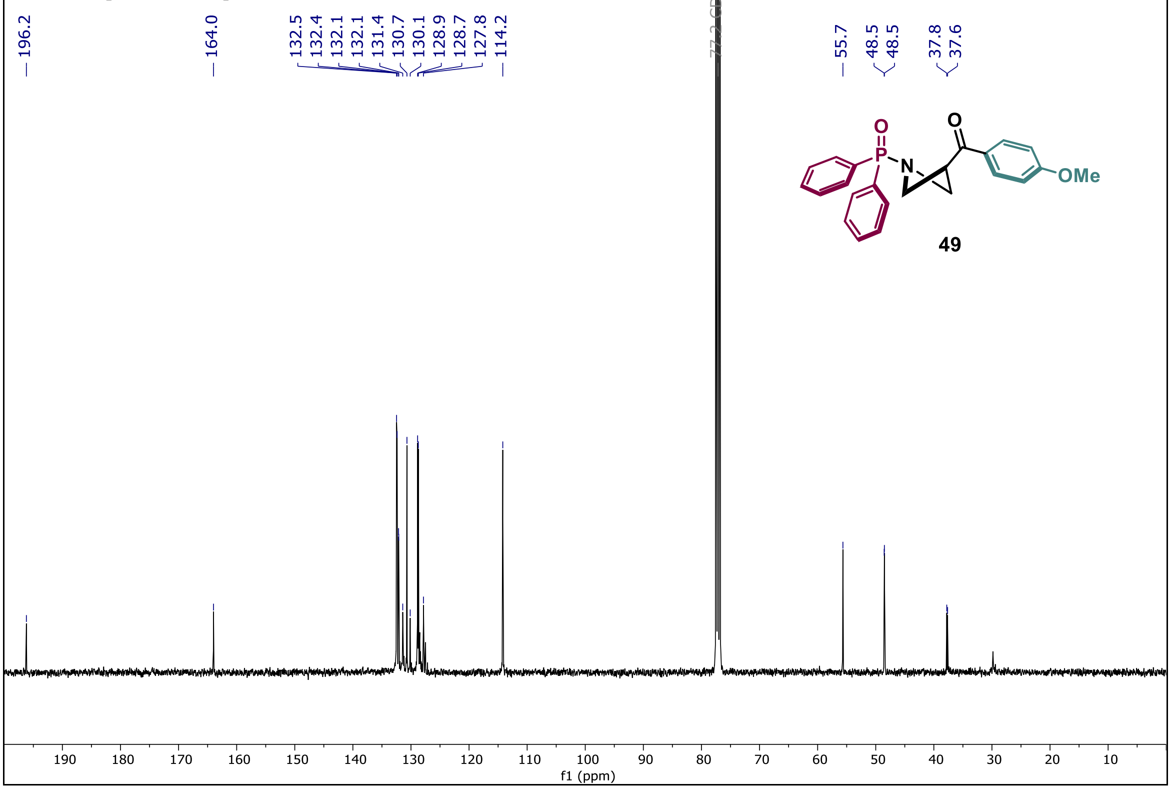

^{31}P NMR spectra of compound **49** in $CDCl_3$

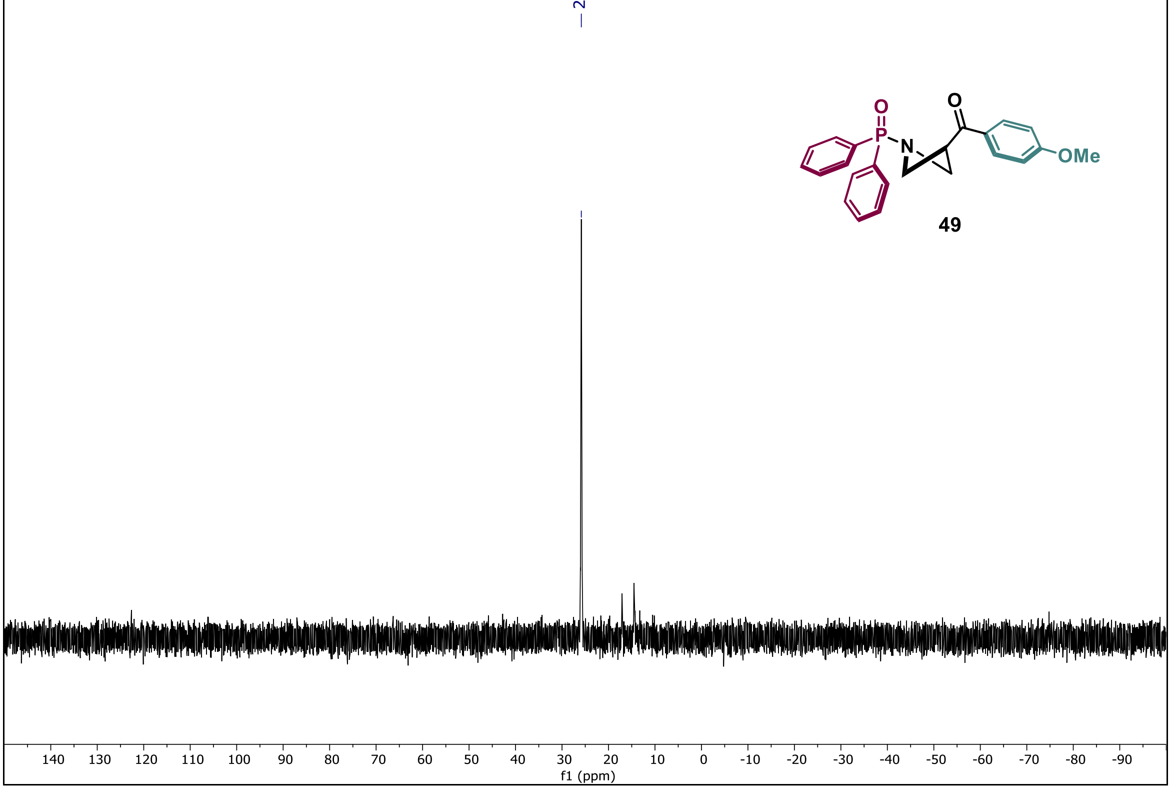

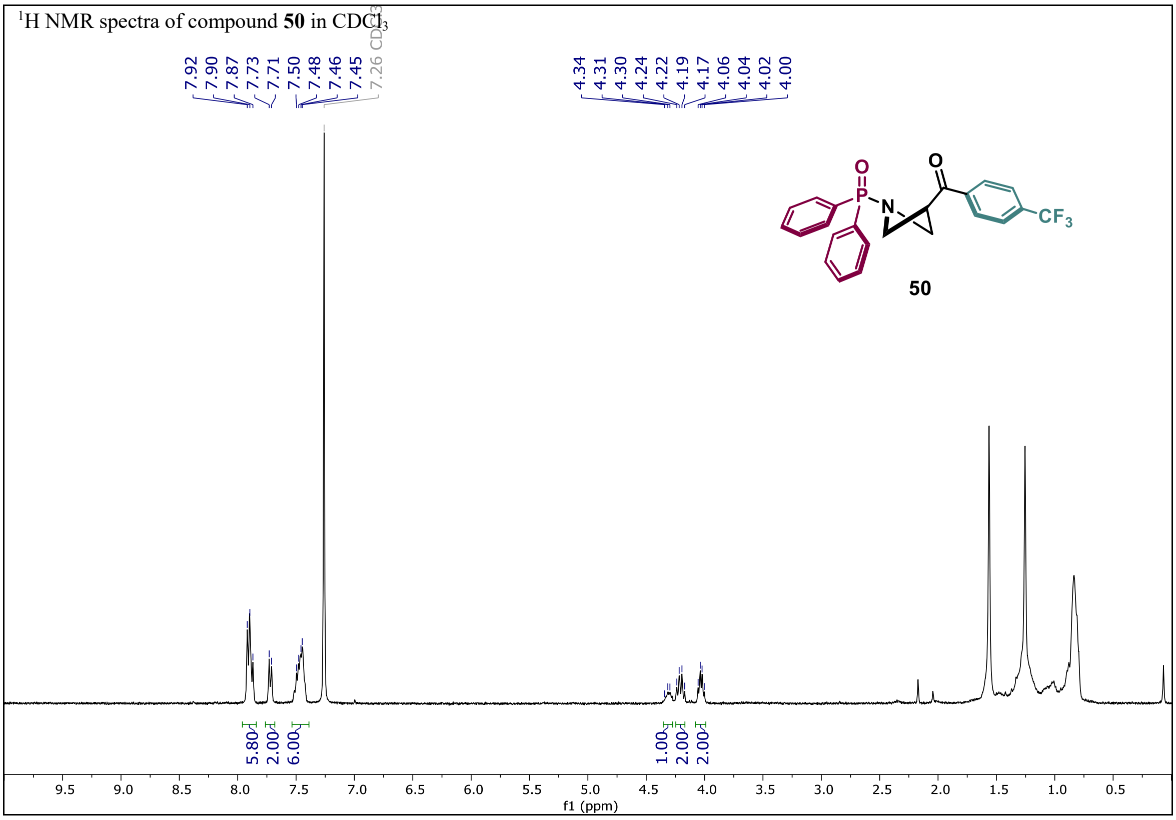

^{1}H NMR spectra of compound **50** in $CDCl_3$
7.92 7.90 7.87 7.73 7.71 7.50 7.48 7.46 7.45 7.26 CDCl3
4.34 4.31 4.30 4.24 4.22 4.19 4.17 4.06 4.04 4.02 4.00
50
5.80 2.00 6.00 1.00 2.00 2.00
f1 (ppm)

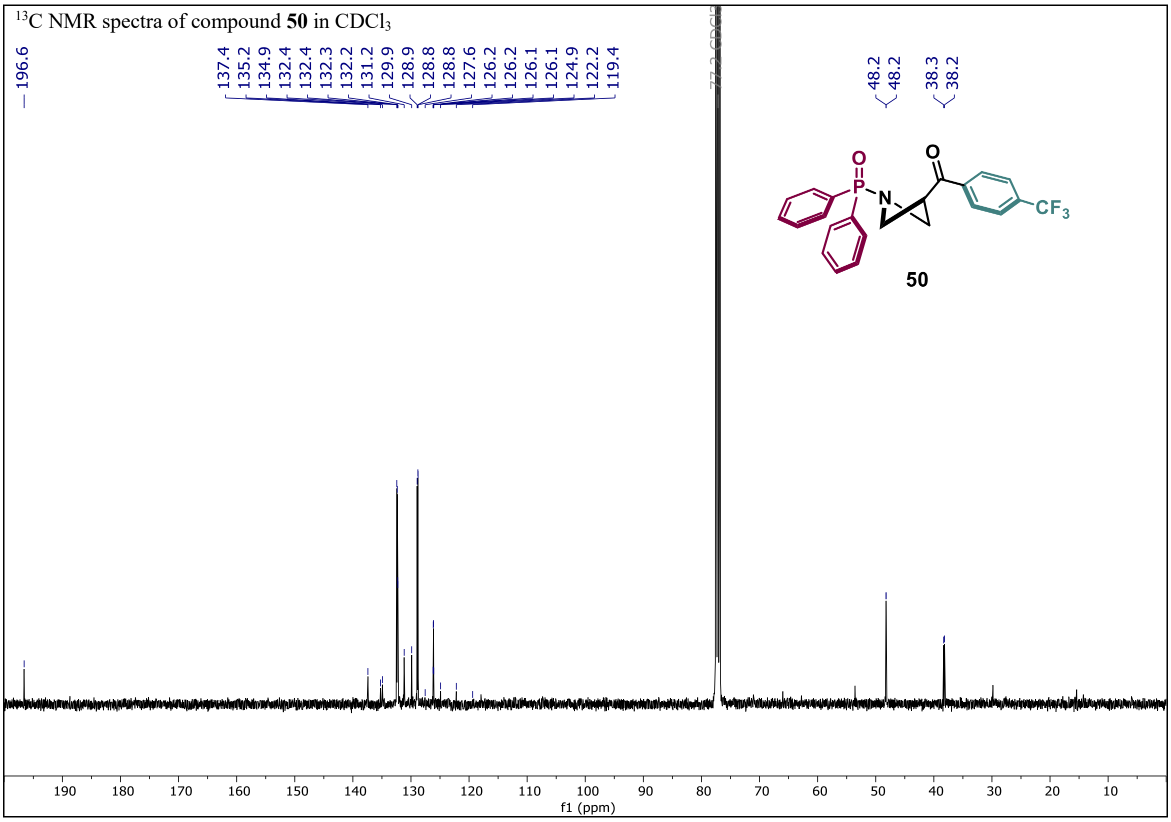

^{13}C NMR spectra of compound **50** in $CDCl_3$
196.6
137.4 135.2 134.9 132.4 132.4 132.3 132.2 131.2 129.9 128.9 128.8 128.8 127.6 126.2 126.2 126.1 124.9 122.2 119.4
77.2 CDCl3
48.2 48.2 38.3 38.2
50
f1 (ppm)

^{31}P NMR spectra of compound **50** in $CDCl_3$

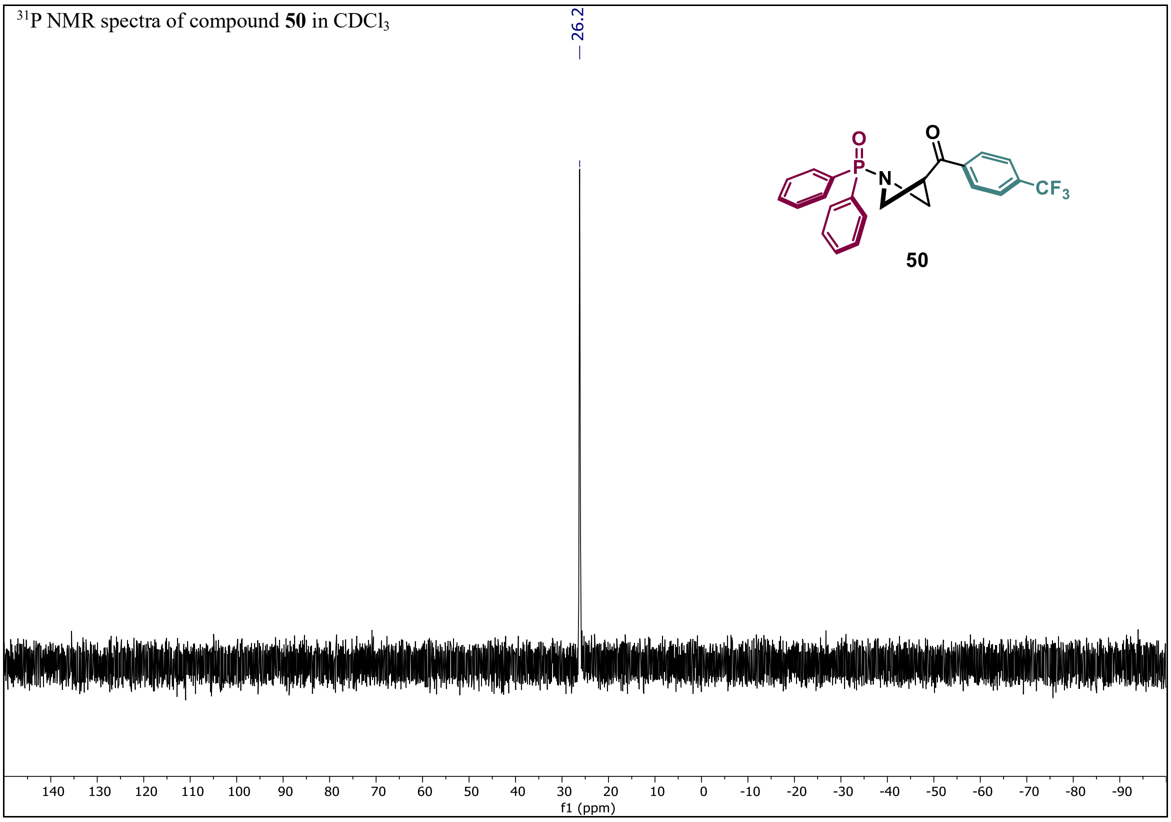

50

^{19}F NMR spectra of compound **50** in $CDCl_3$

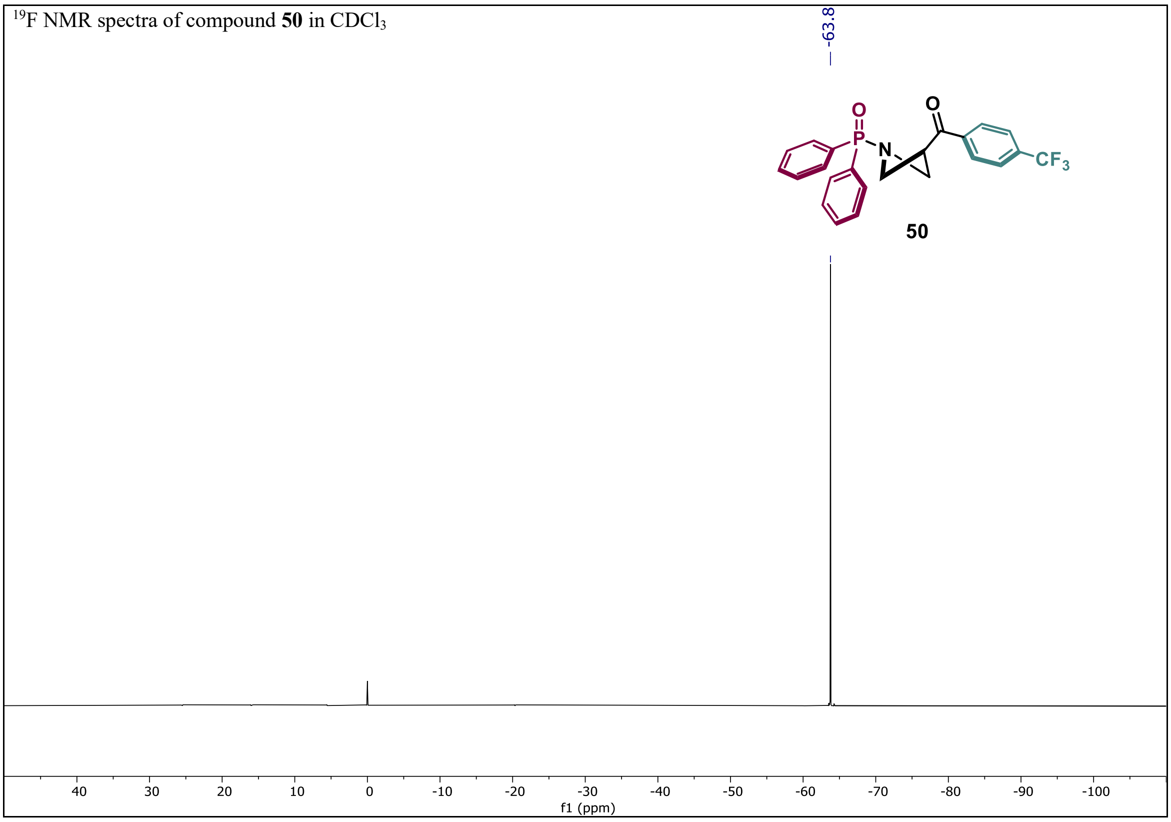

50

^{1}H NMR spectra of compound **51** in $CDCl_3$

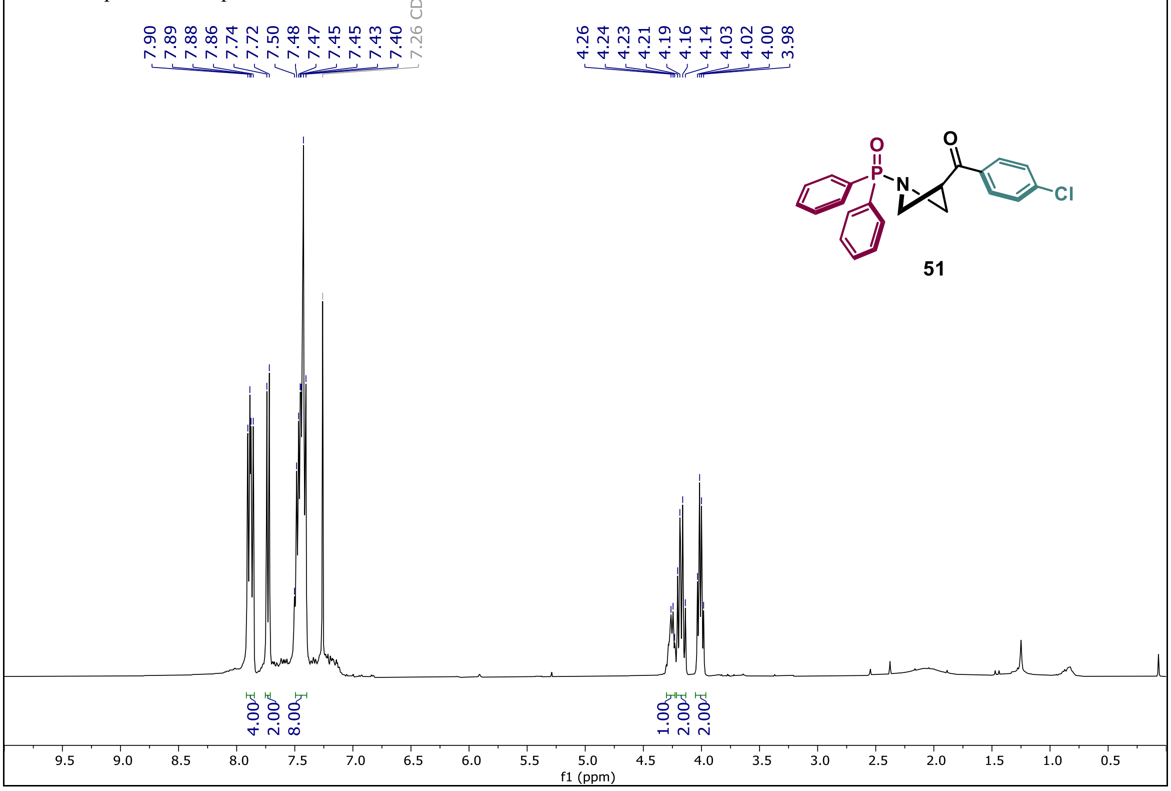

^{13}C NMR spectra of compound **51** in $CDCl_3$

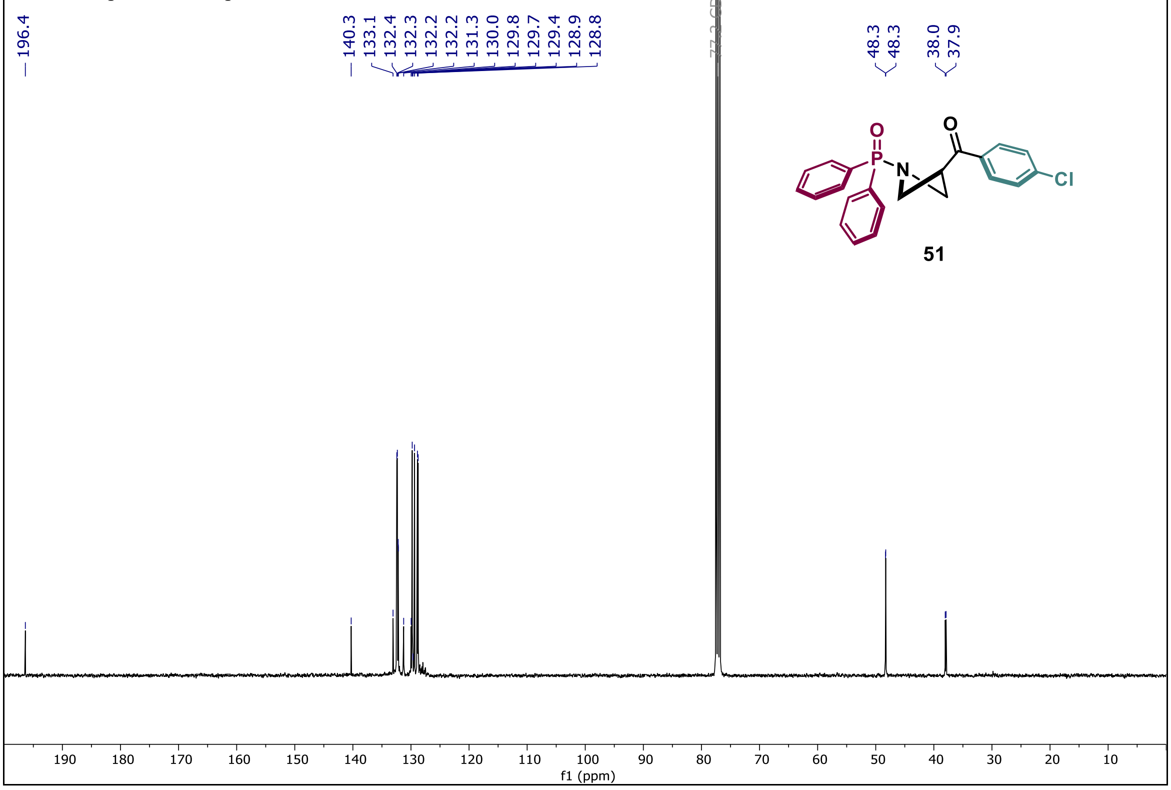

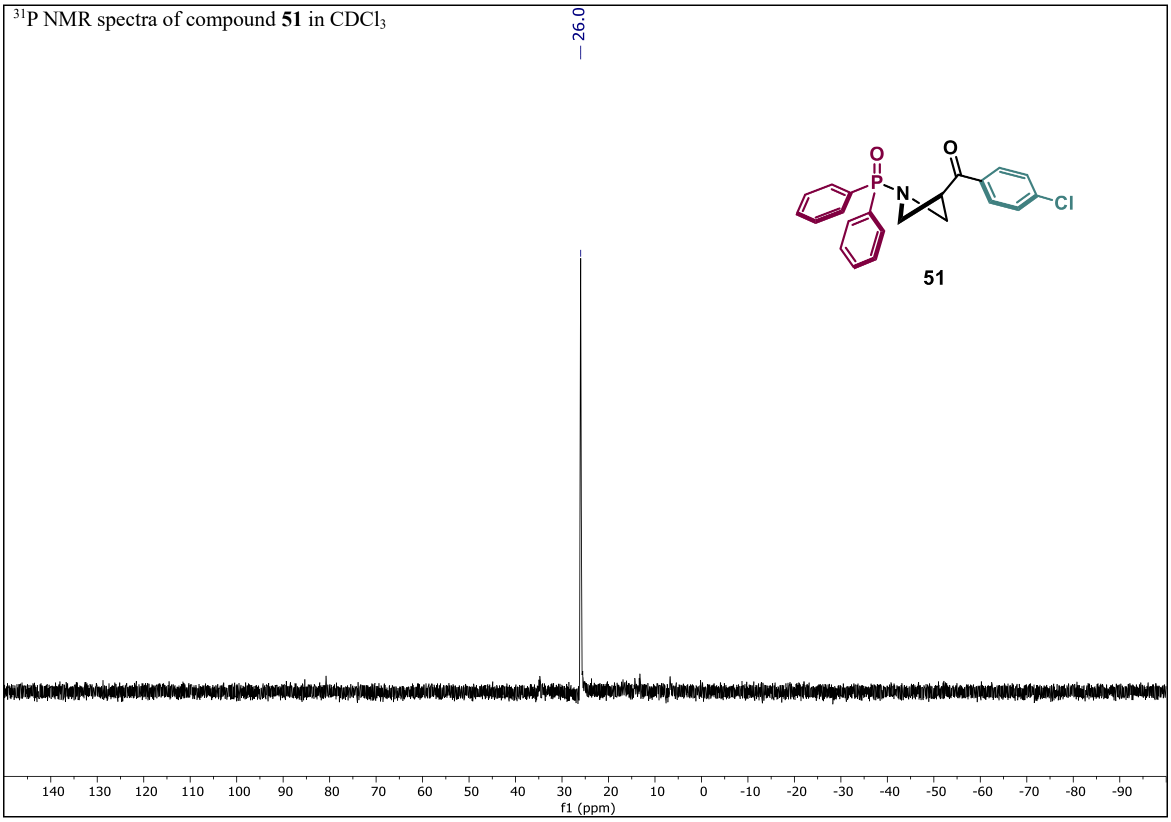
^{31}P NMR spectra of compound **51** in $CDCl_3$
26.0
51
f1 (ppm)

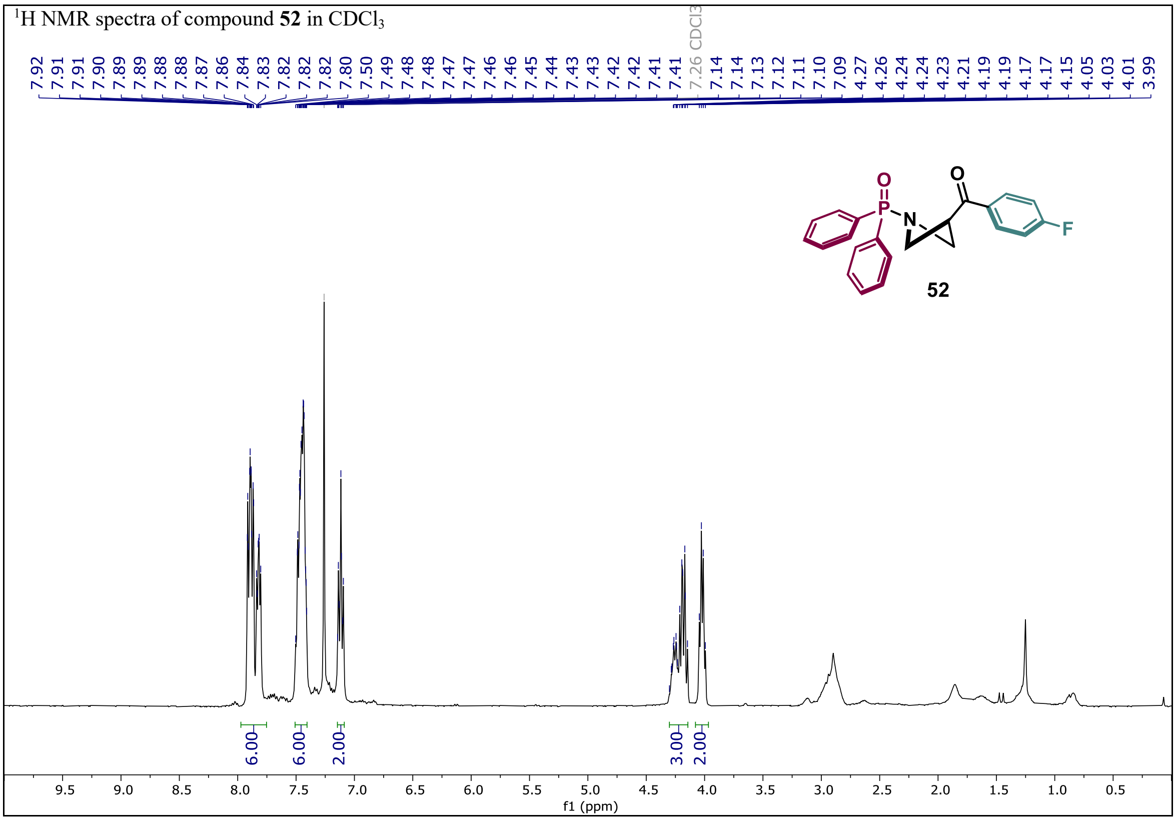
^{1}H NMR spectra of compound **52** in $CDCl_3$
7.92 7.91 7.91 7.90 7.89 7.88 7.88 7.87 7.86 7.84 7.83 7.82 7.82 7.82 7.80 7.50 7.49 7.48 7.48 7.47 7.47 7.46 7.46 7.45 7.44 7.43 7.43 7.42 7.42 7.41 7.41 7.26 CDCl3 7.14 7.13 7.12 7.11 7.10 7.09 4.27 4.26 4.24 4.24 4.23 4.21 4.19 4.19 4.17 4.15 4.05 4.03 4.01 3.99
52
6.00 6.00 2.00 3.00 2.00
f1 (ppm)

^{13}C NMR spectra of compound **52** in $CDCl_3$

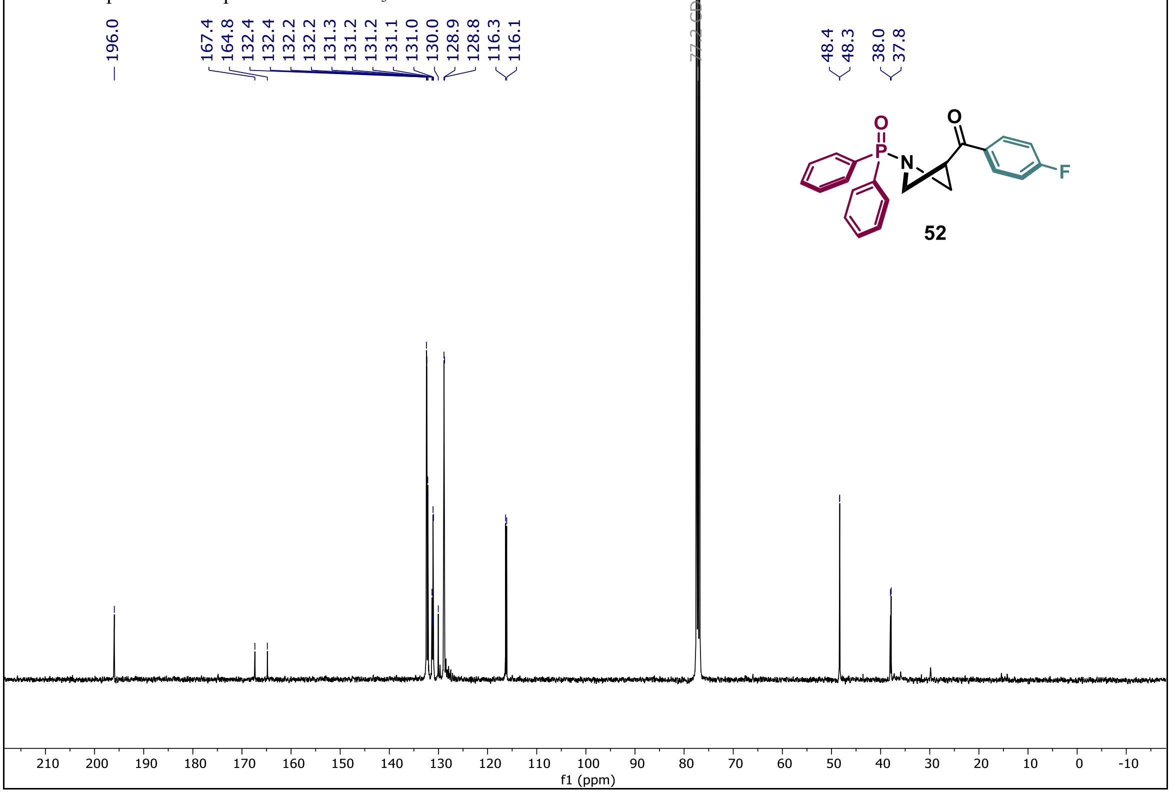

^{31}P NMR spectra of compound **52** in $CDCl_3$

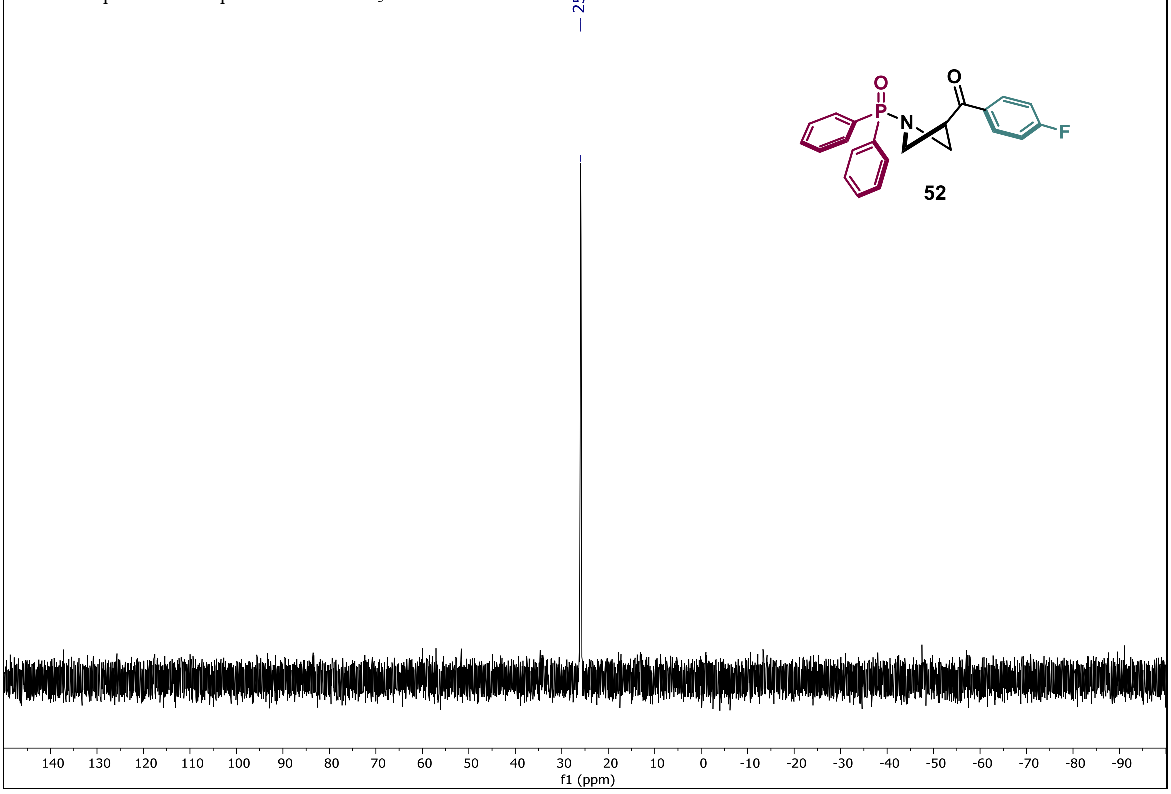

^{19}F NMR spectra of compound **52** in $CDCl_3$

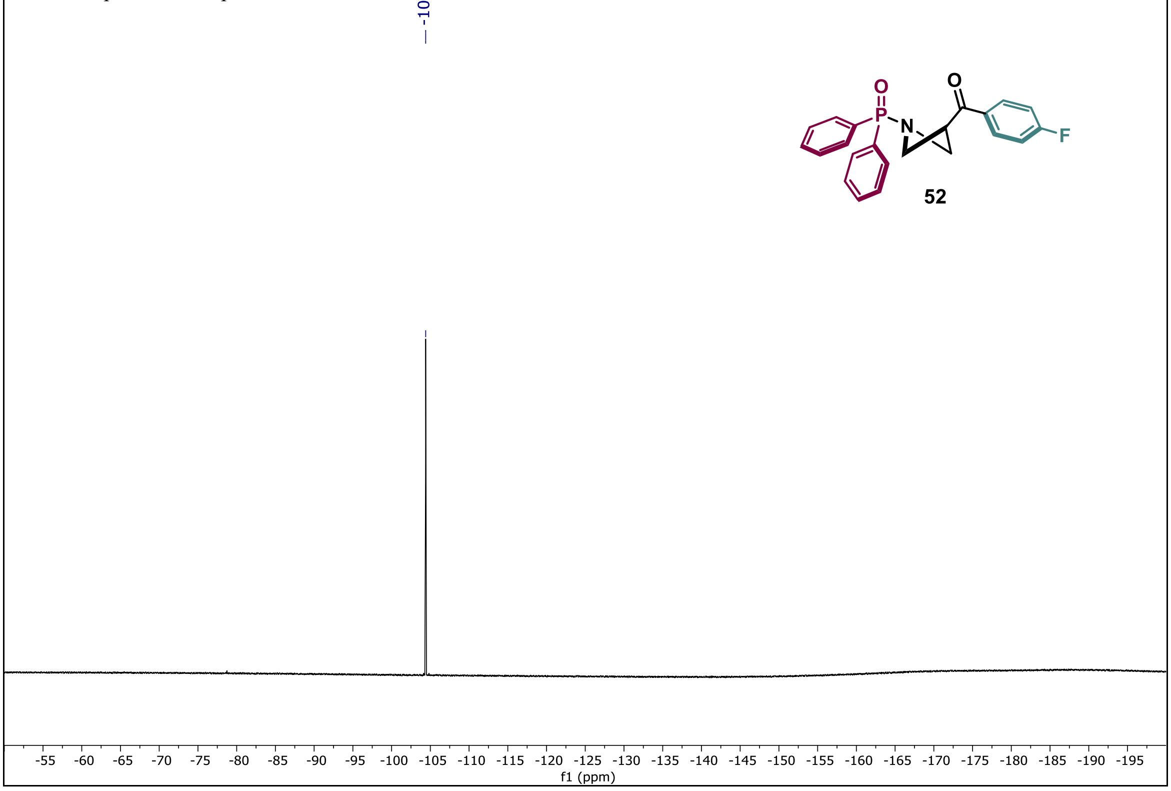

^{1}H NMR spectra of compound **53** in $CDCl_3$

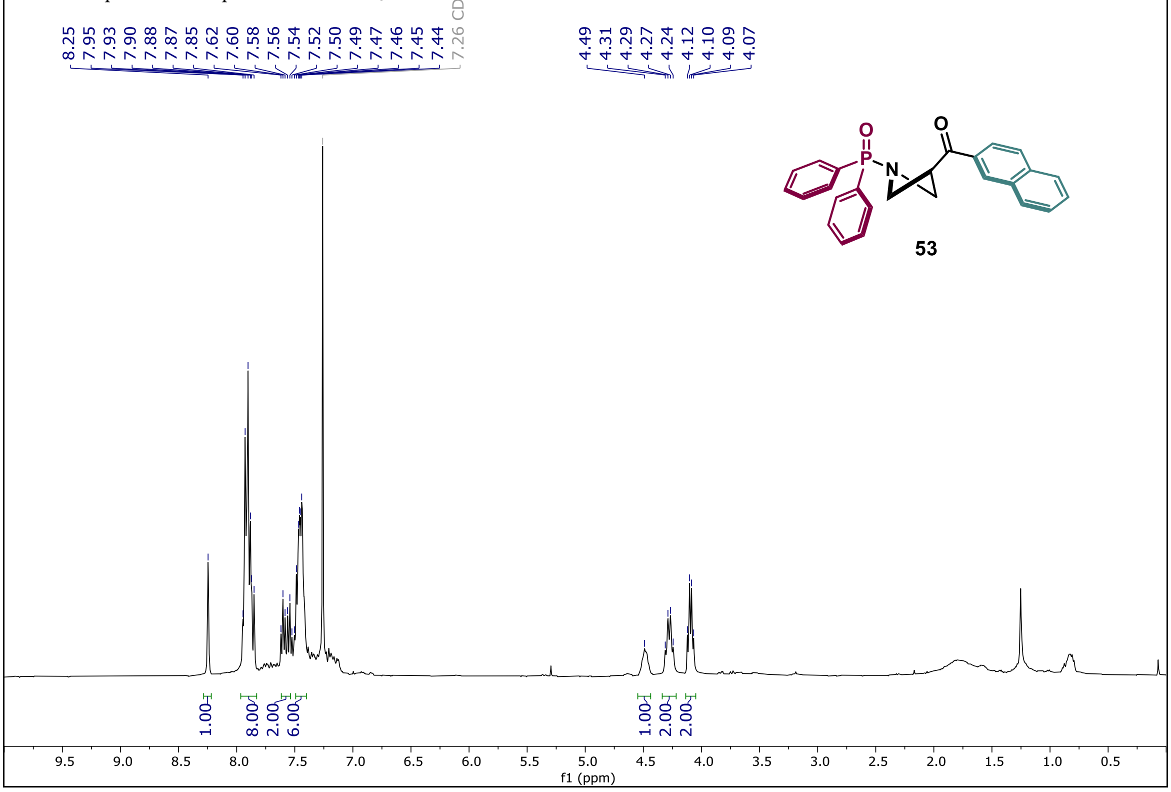

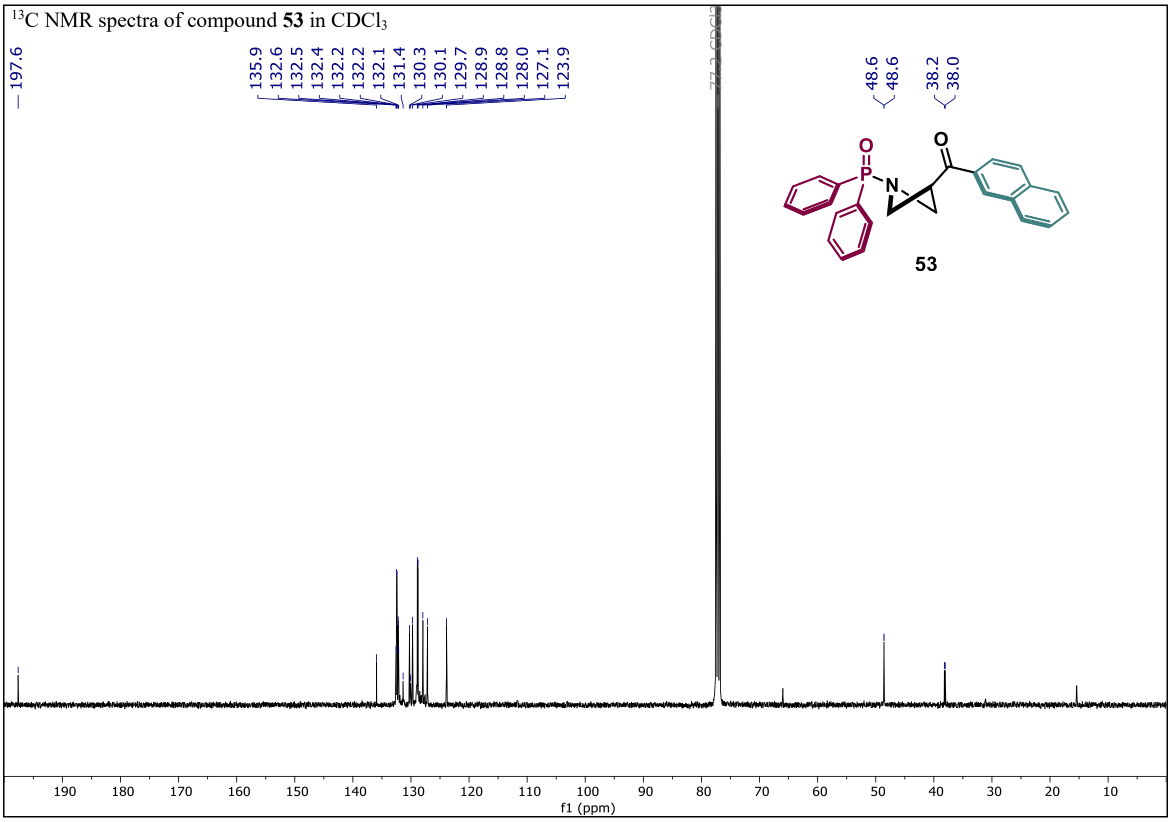

^{13}C NMR spectra of compound **53** in $CDCl_3$

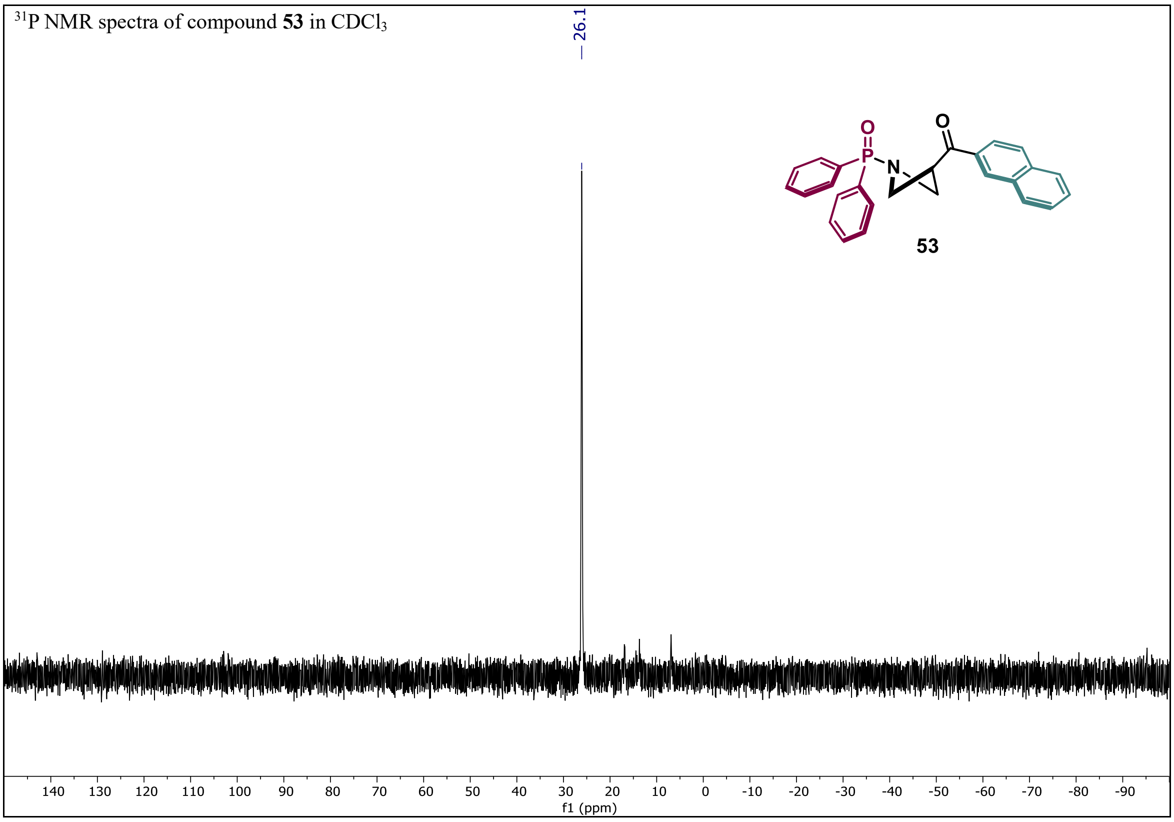

^{31}P NMR spectra of compound **53** in $CDCl_3$

^{1}H NMR spectra of compound **54** in $CDCl_3$

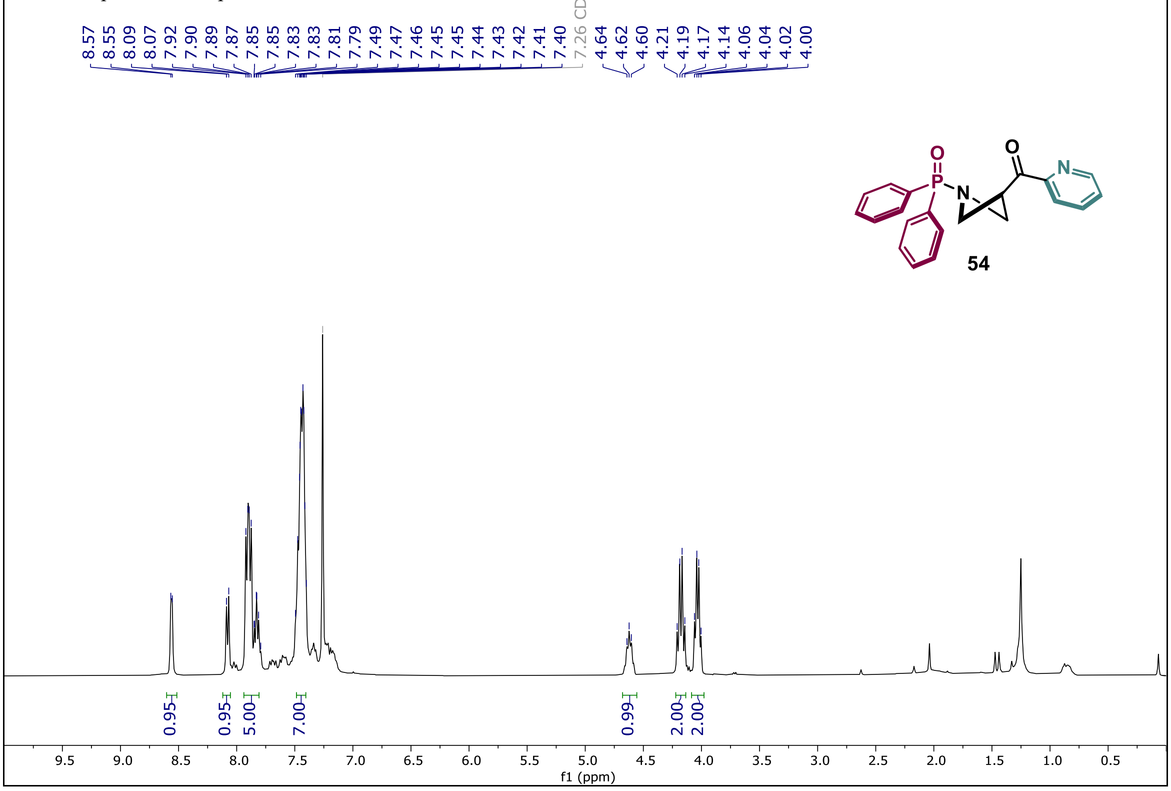

^{13}C NMR spectra of compound **54** in $CDCl_3$

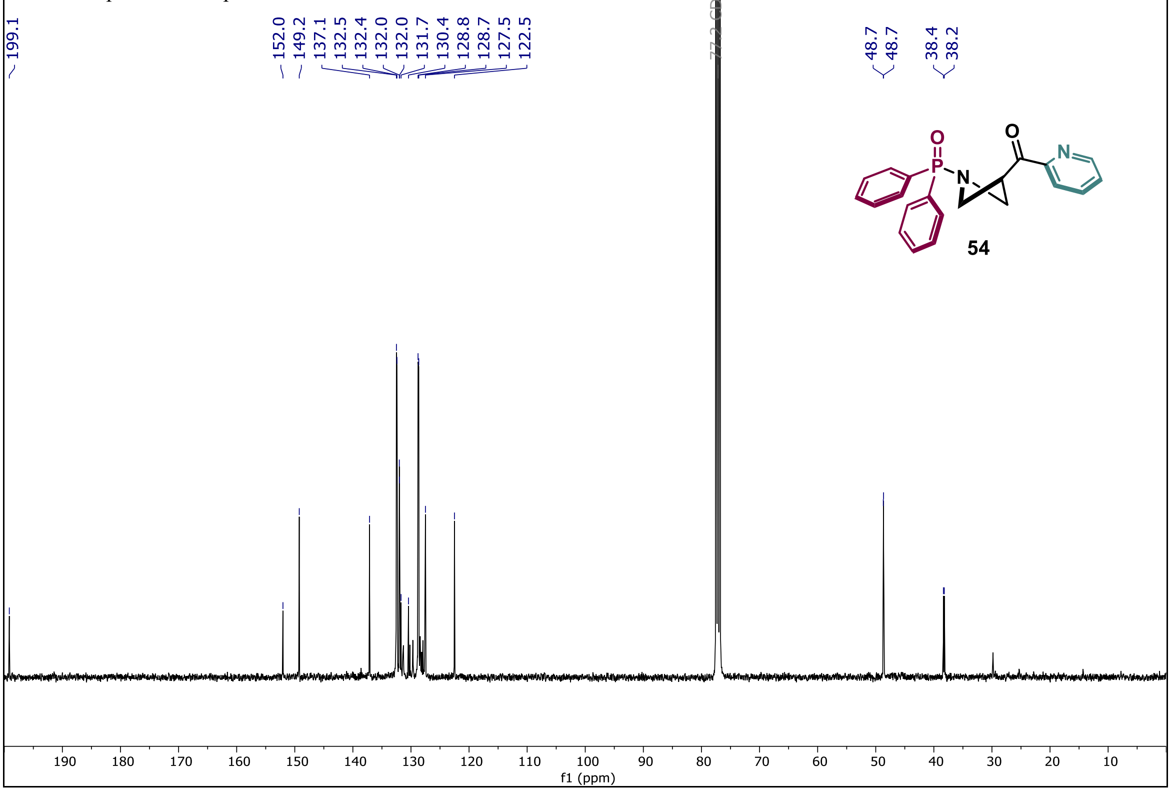

^{31}P NMR spectra of compound **54** in $CDCl_3$

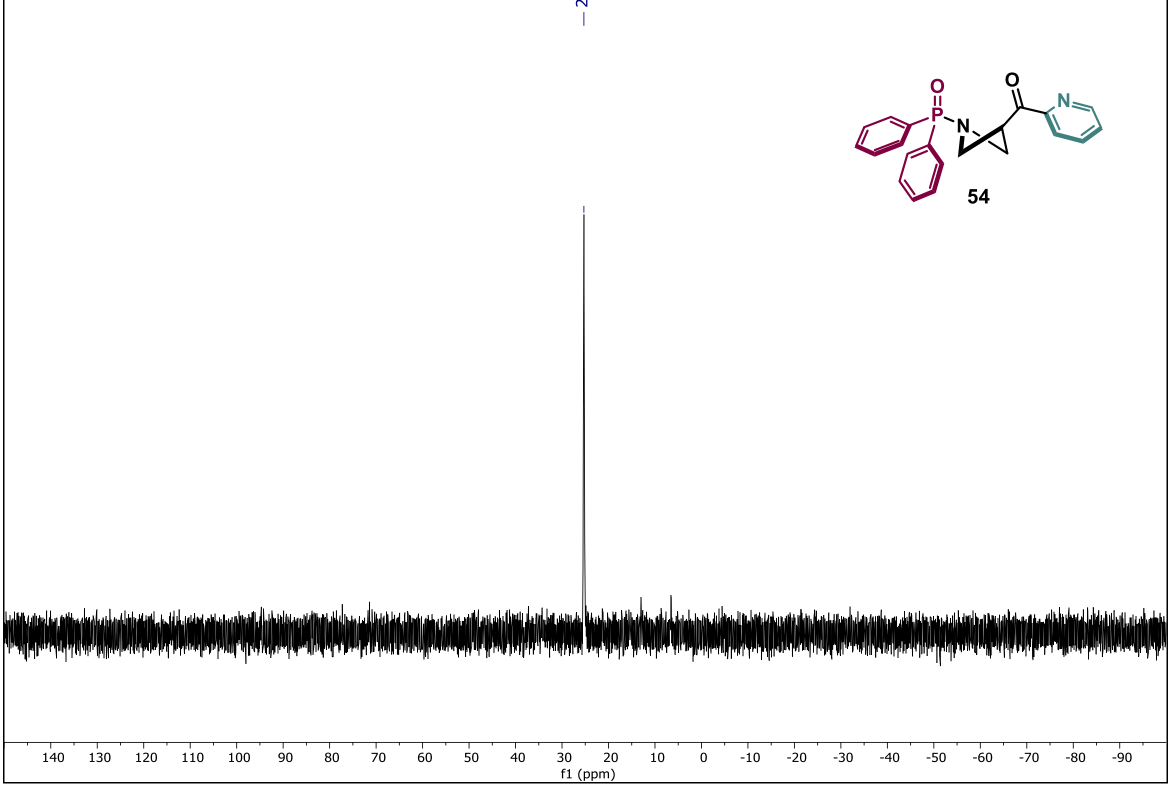

^{1}H NMR spectra of compound **55** in $CDCl_3$

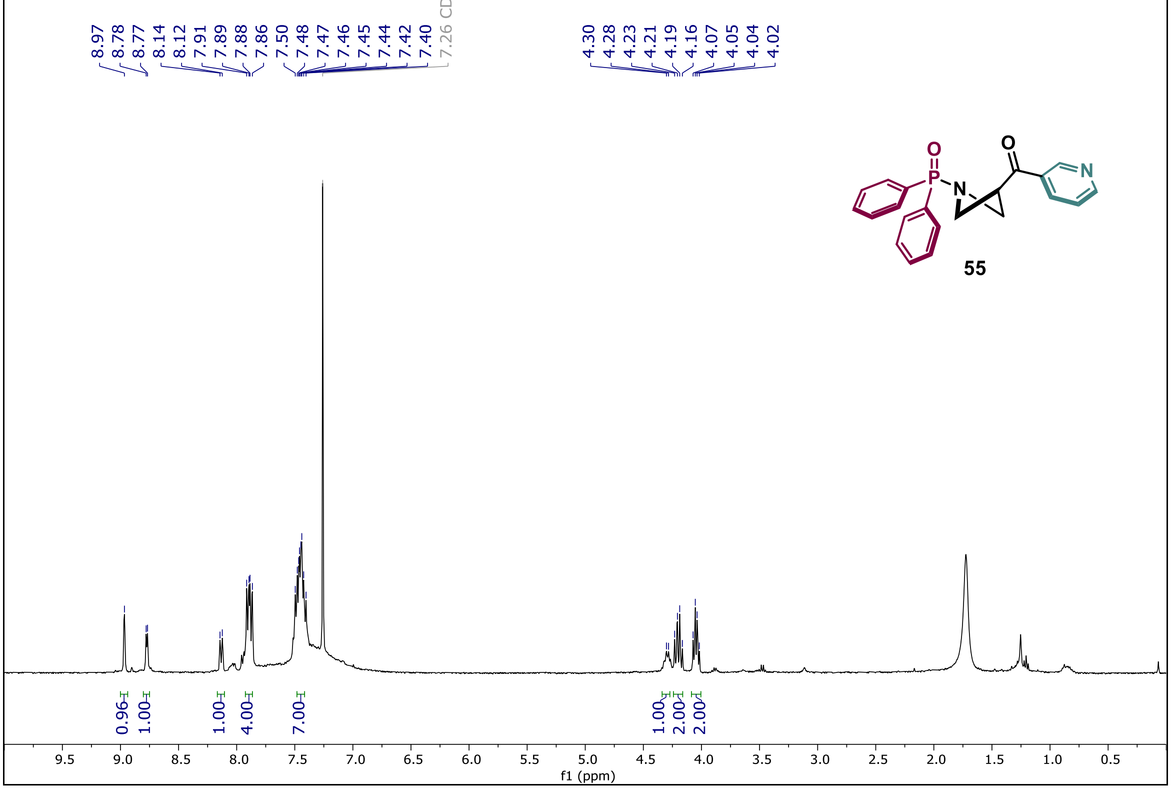

^{13}C NMR spectra of compound **55** in $CDCl_3$

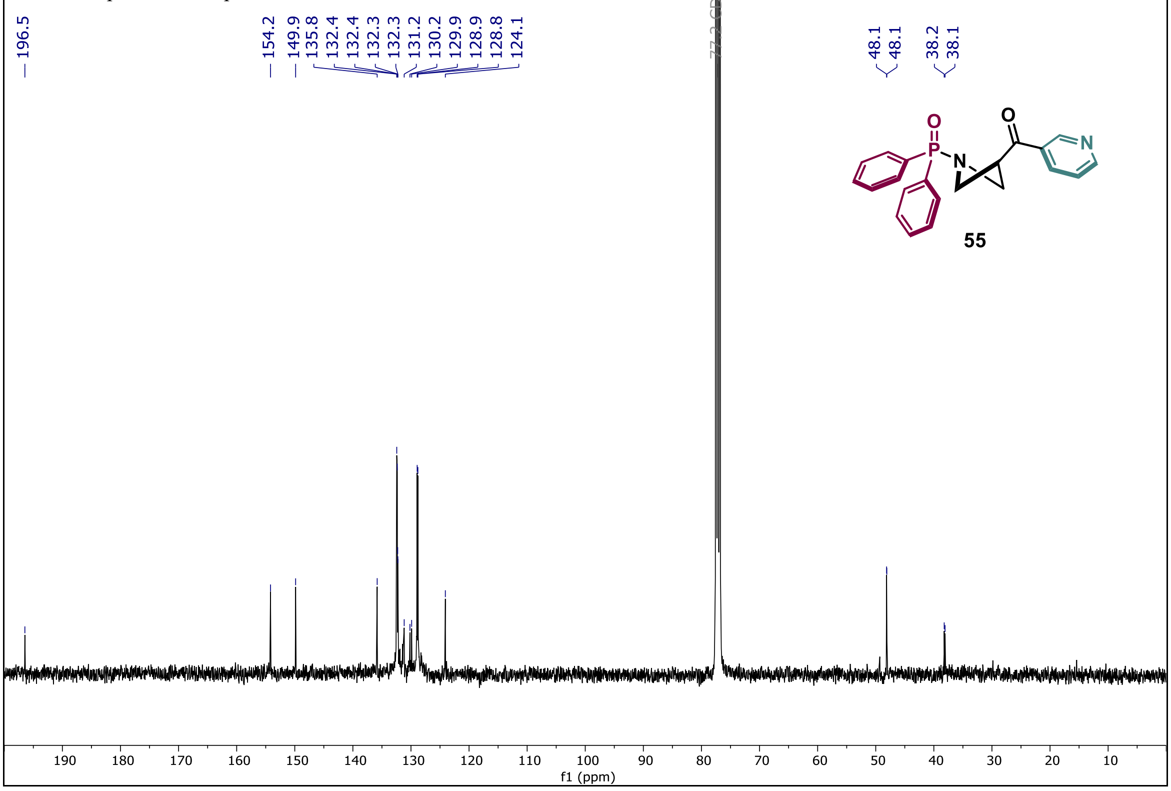

^{31}P NMR spectra of compound **55** in $CDCl_3$

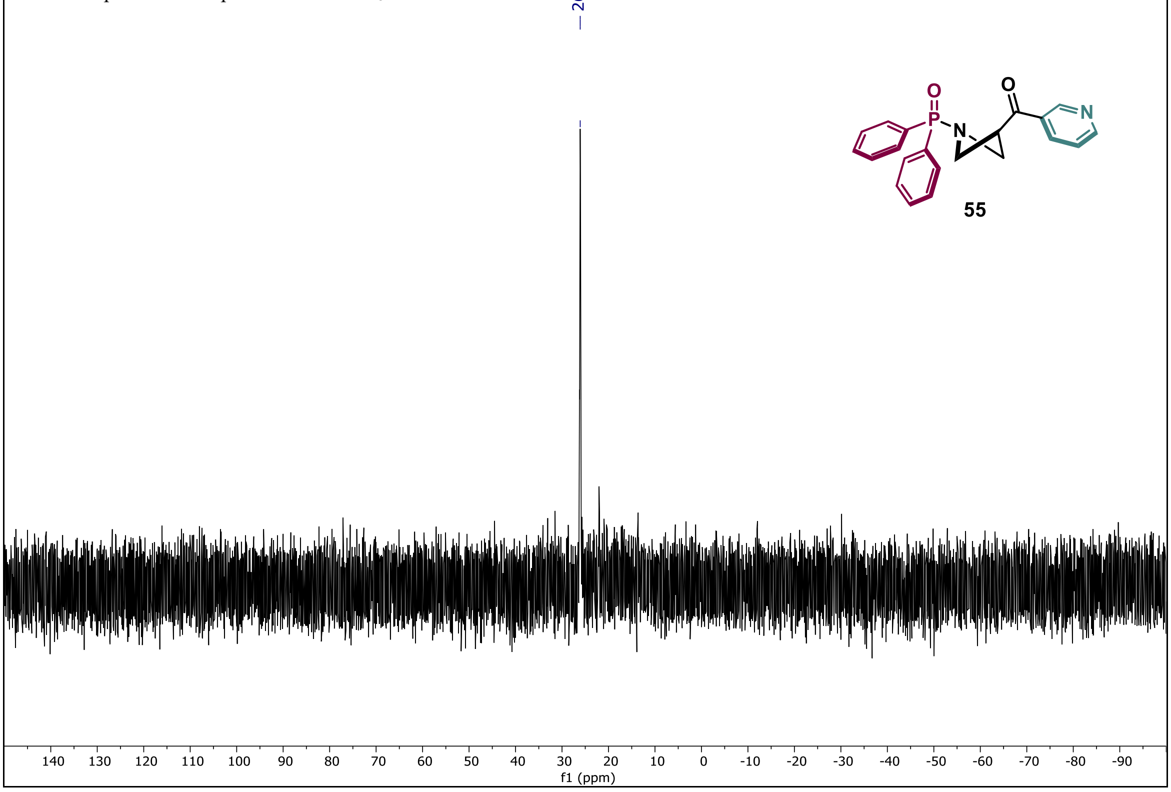

^{1}H NMR spectra of compound **56** in $CDCl_3$

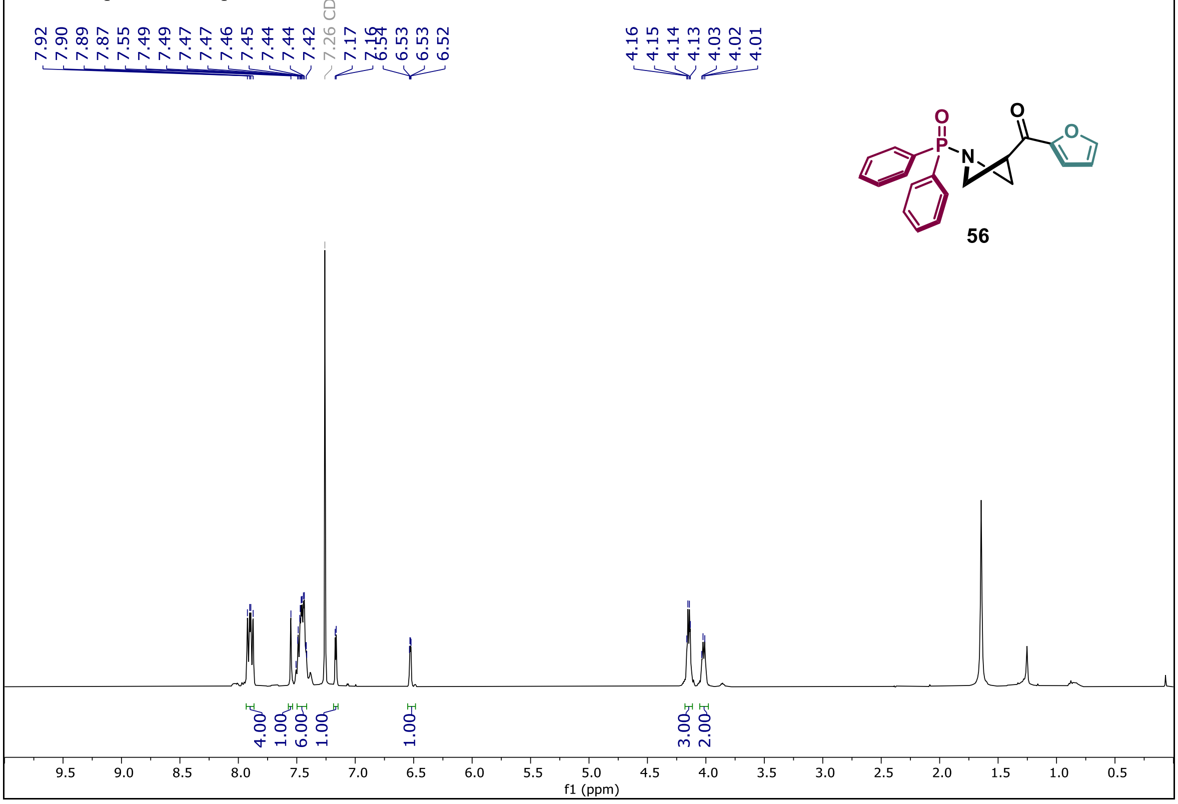

^{13}C NMR spectra of compound **56** in $CDCl_3$

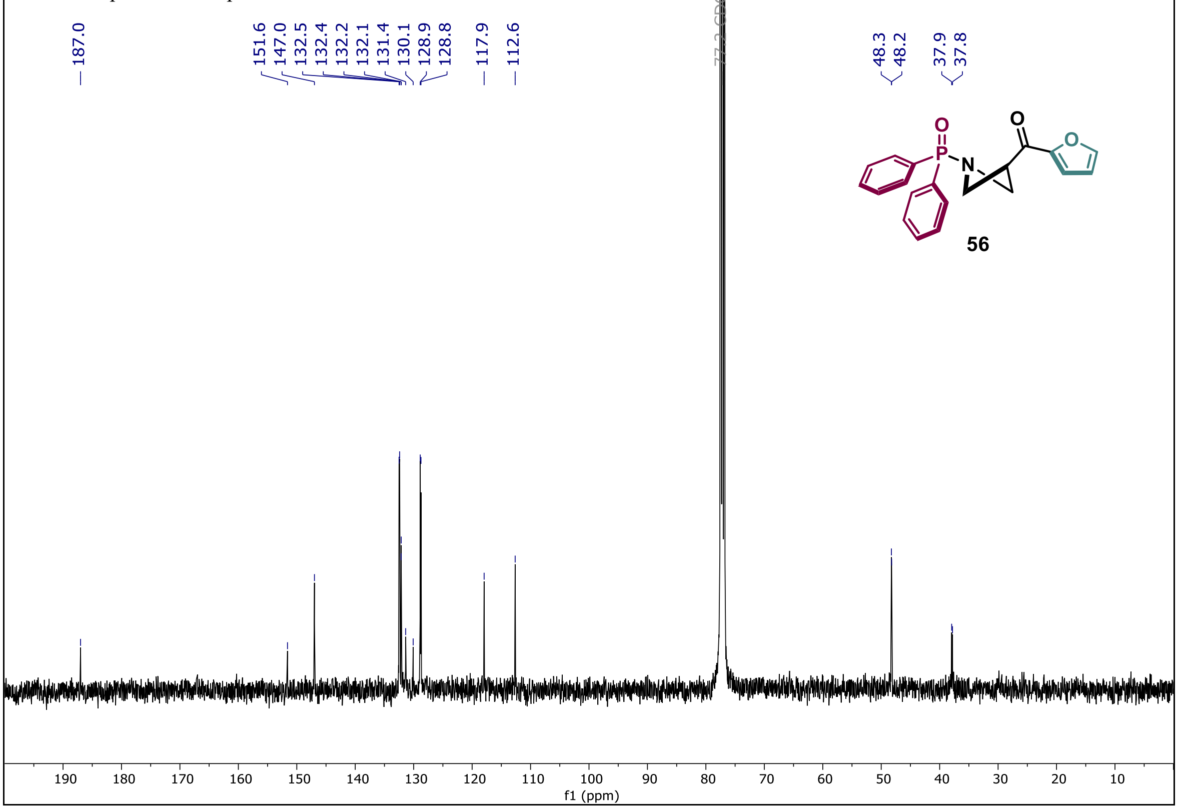

^{31}P NMR spectra of compound **56** in $CDCl_3$

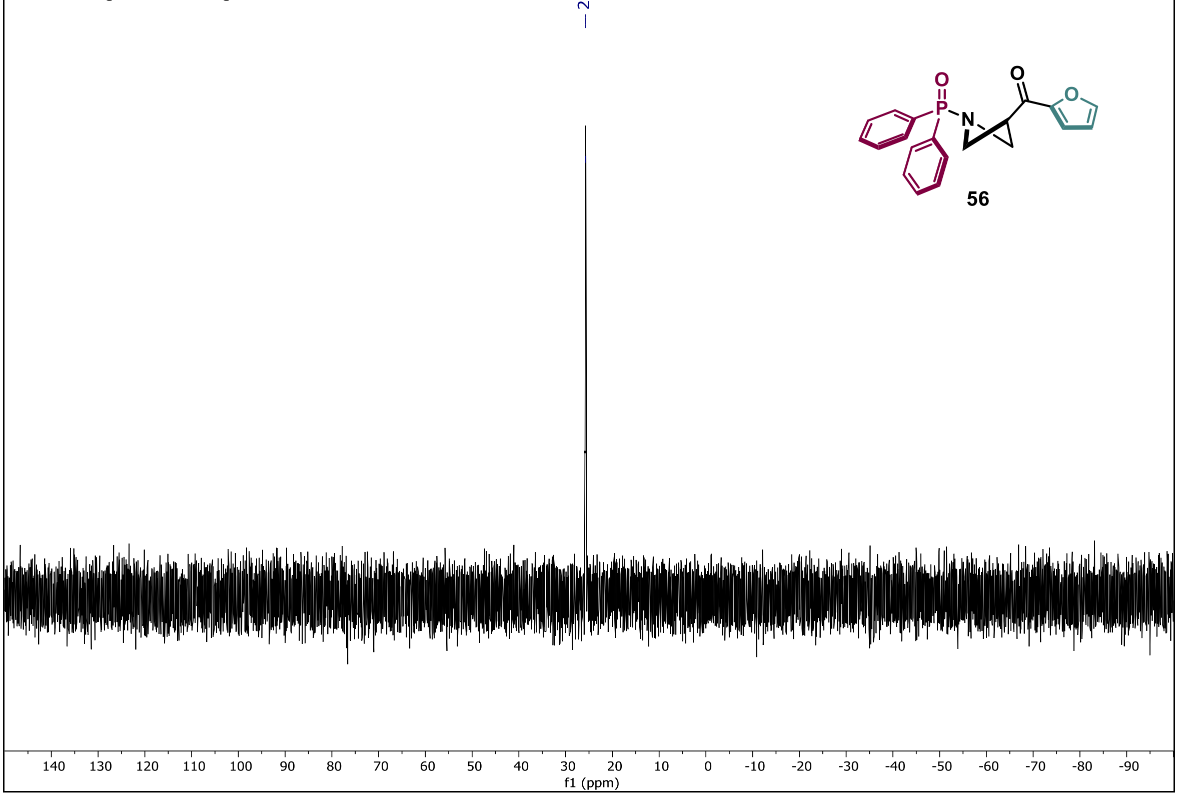

^{1}H NMR spectra of compound **57** in $CDCl_3$

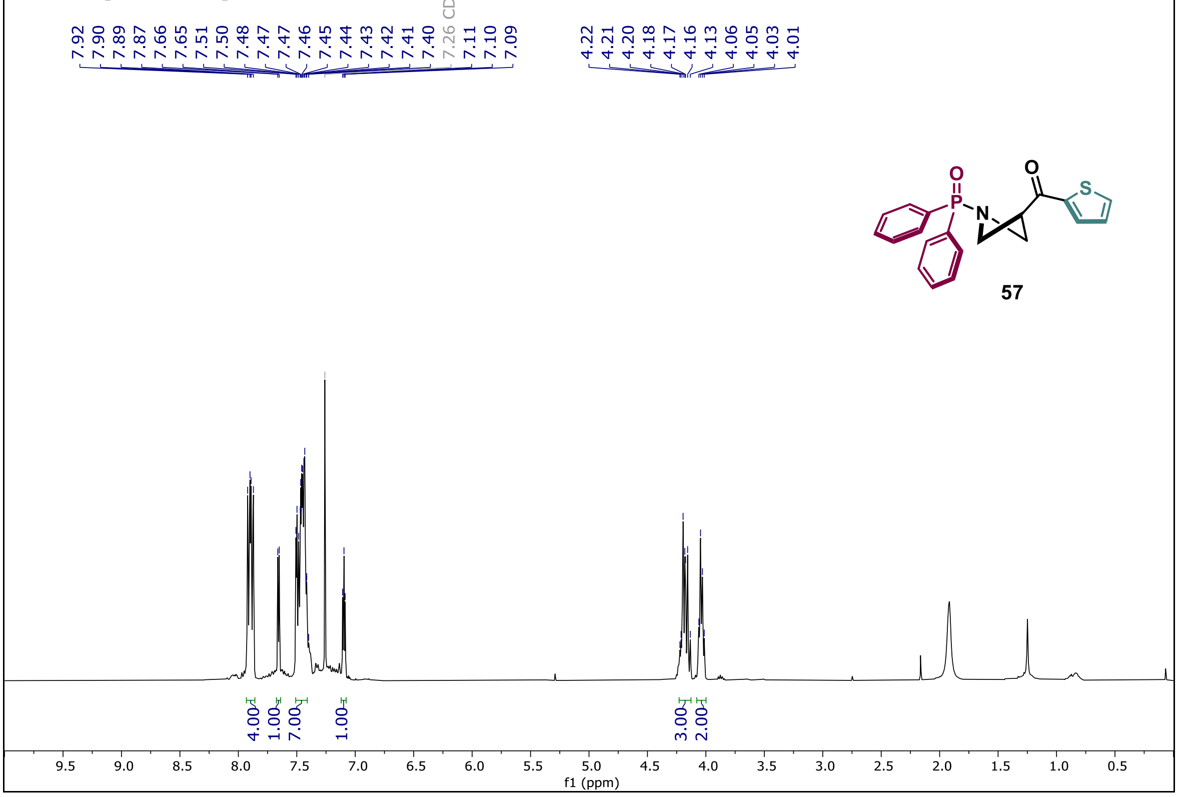

^{13}C NMR spectra of compound **57** in $CDCl_3$

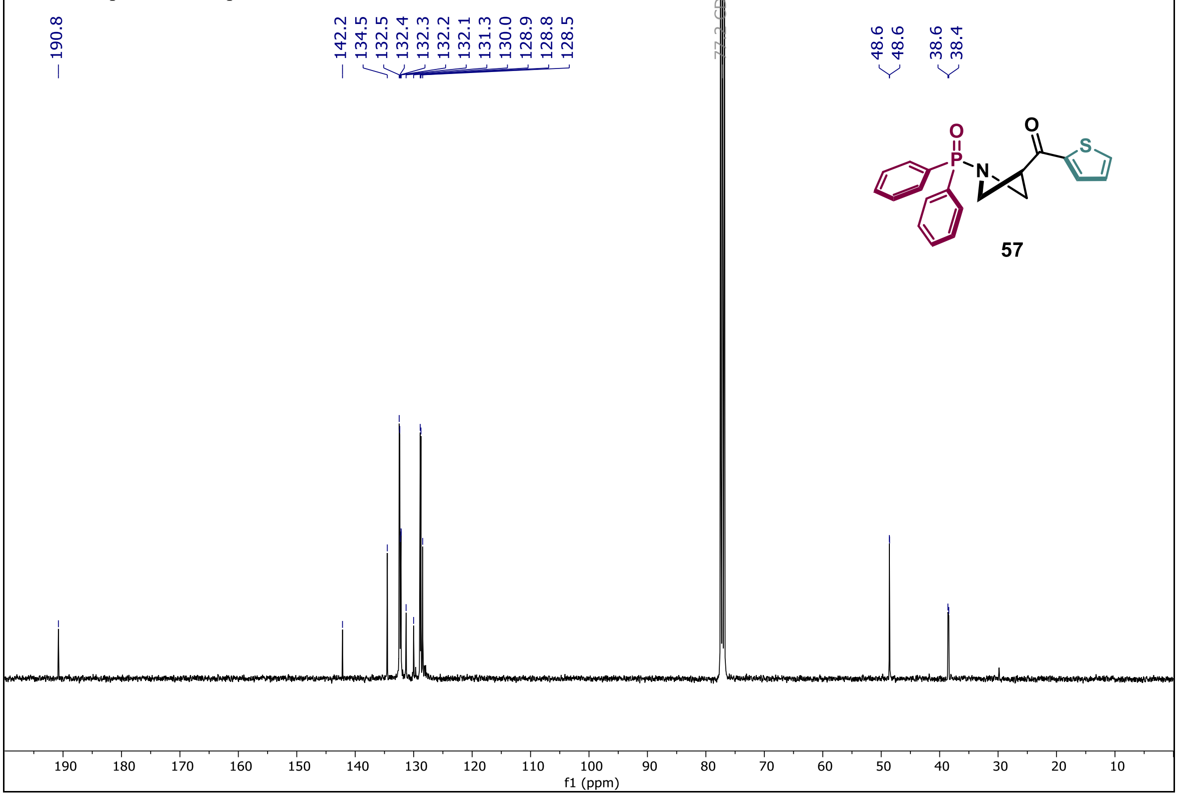

^{31}P NMR spectra of compound **57** in $CDCl_3$

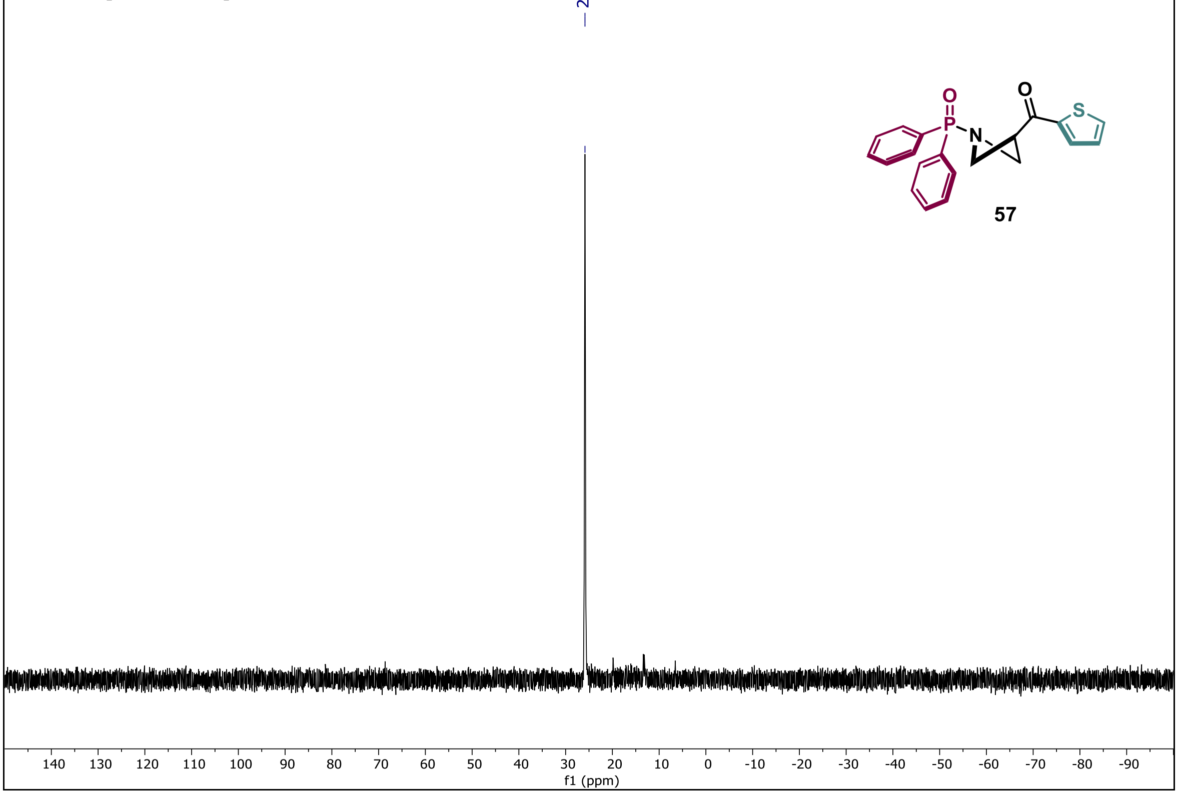

^{1}H NMR spectra of compound **58** in $CDCl_3$

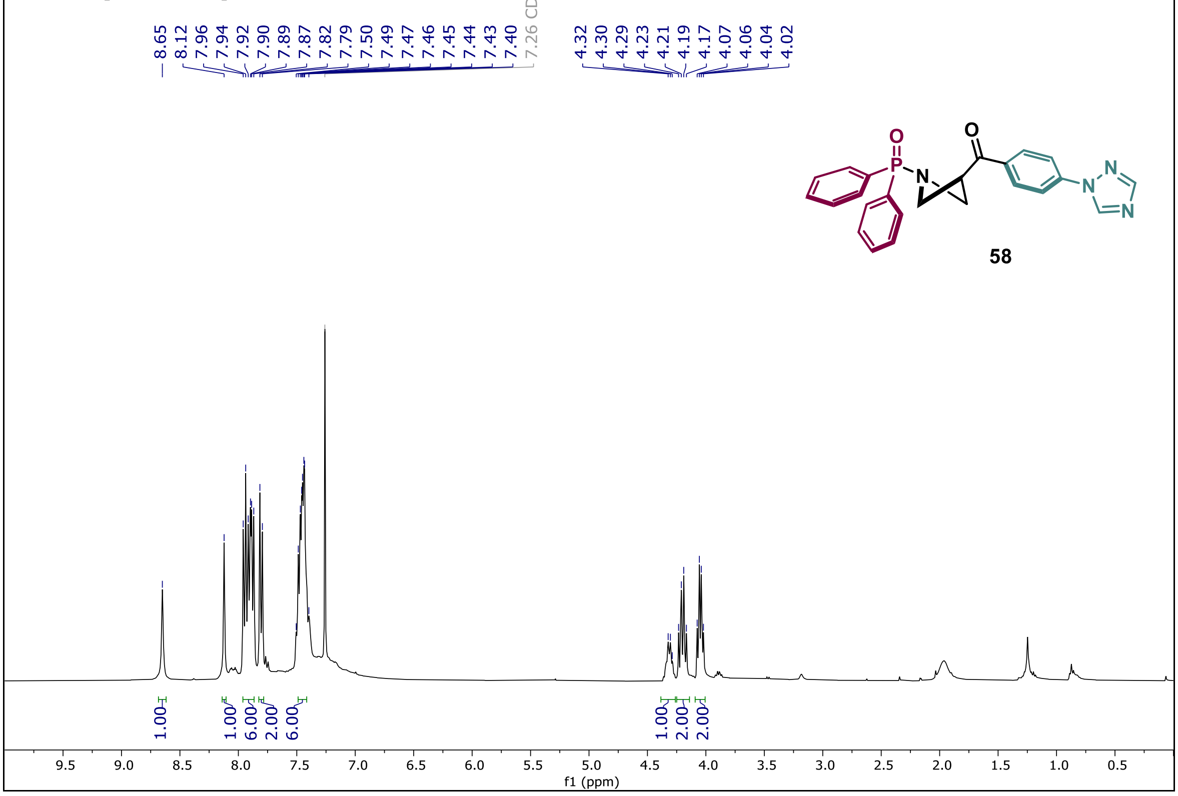

^{13}C NMR spectra of compound **58** in $CDCl_3$

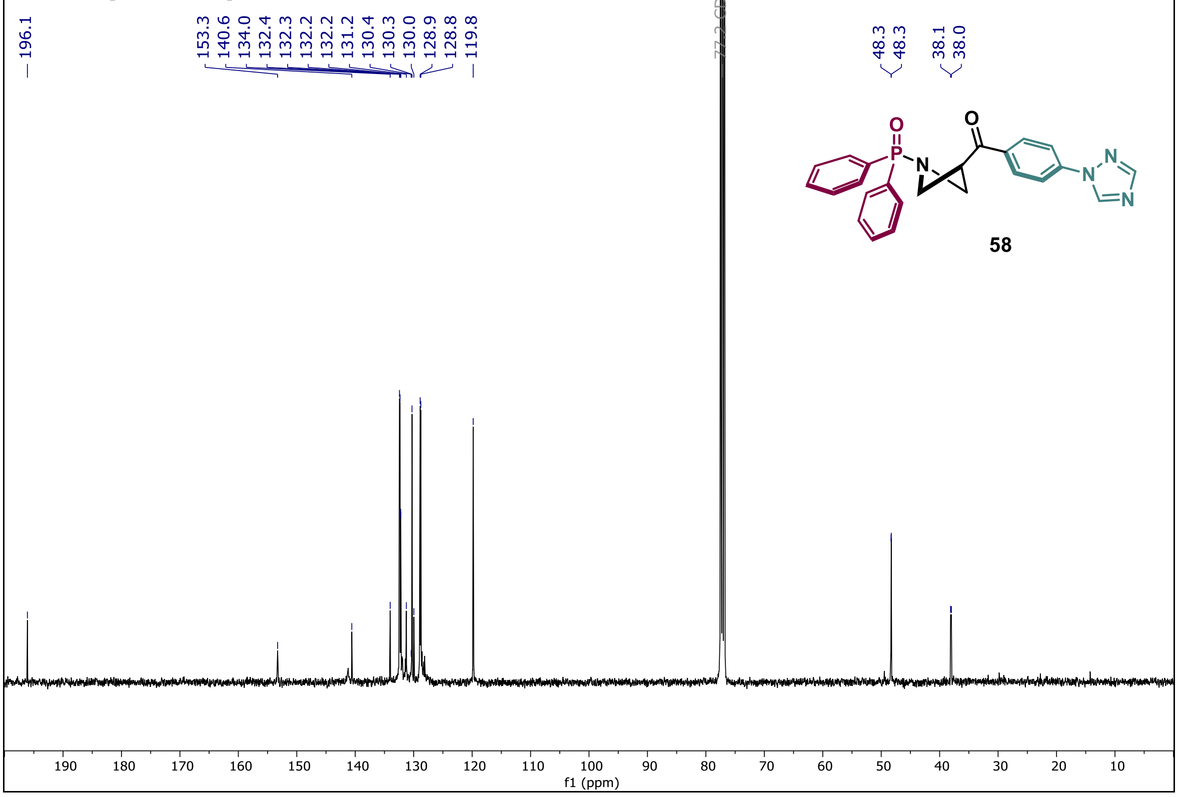

^{31}P NMR spectra of compound **58** in $CDCl_3$

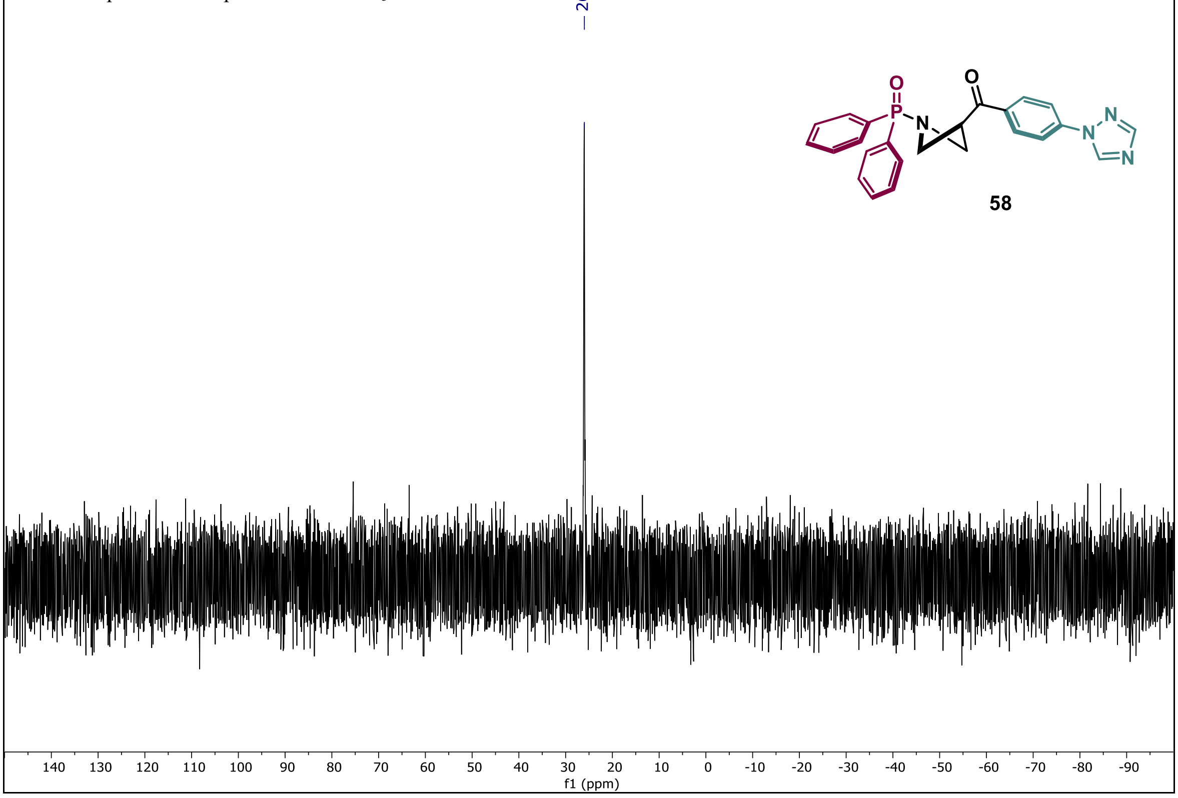

^{1}H NMR spectra of compound **59** in $CDCl_3$

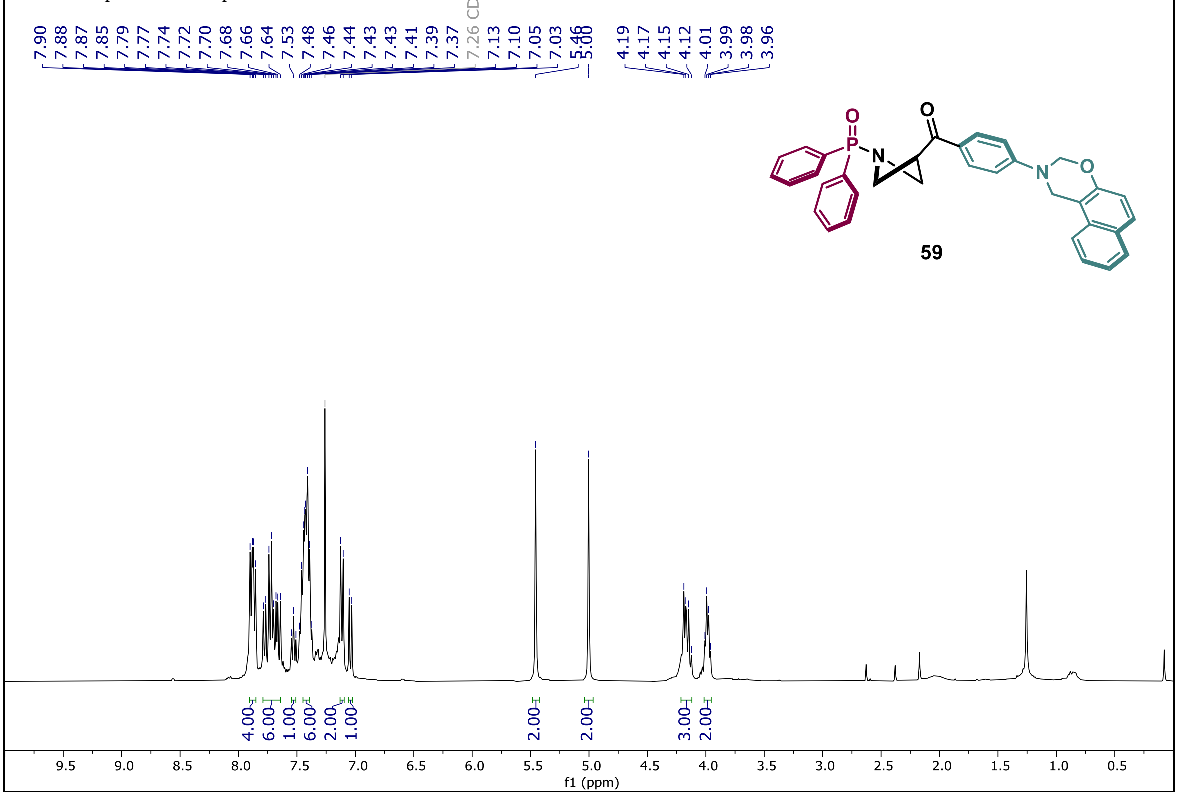

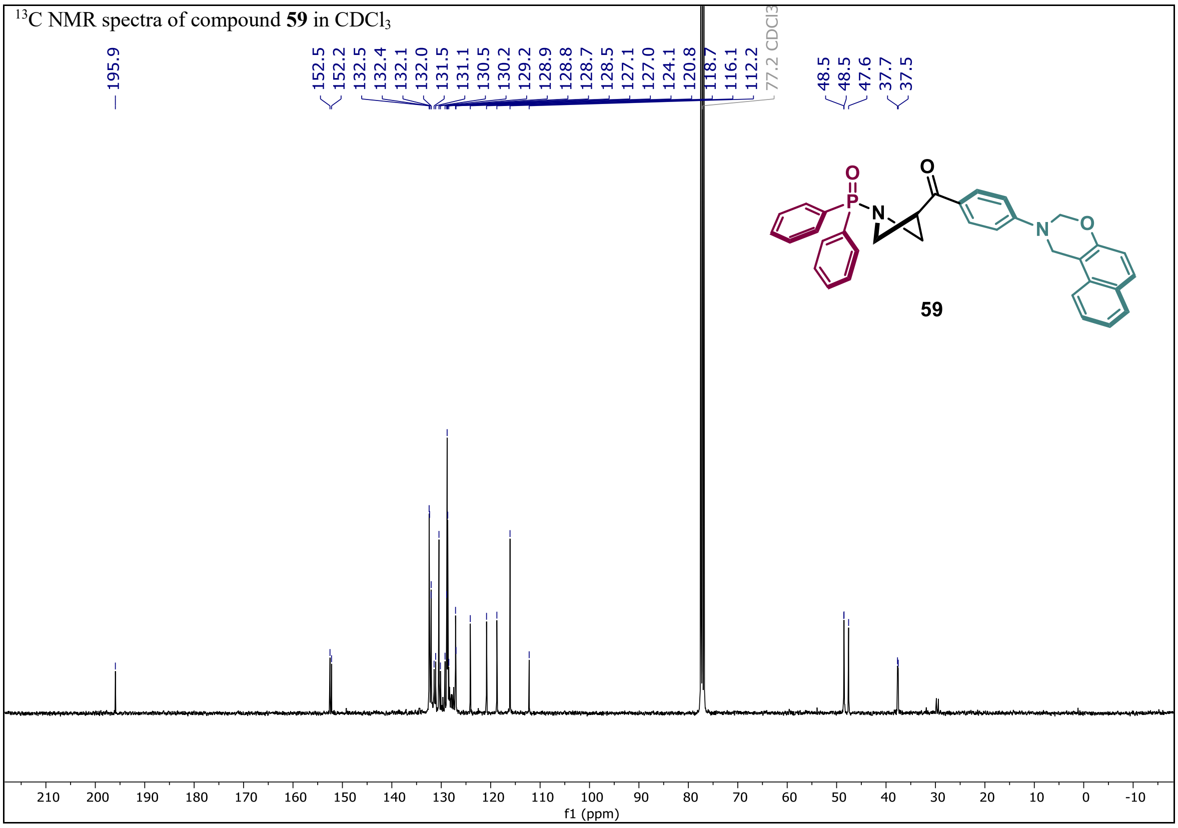

^{13}C NMR spectra of compound **59** in $CDCl_3$

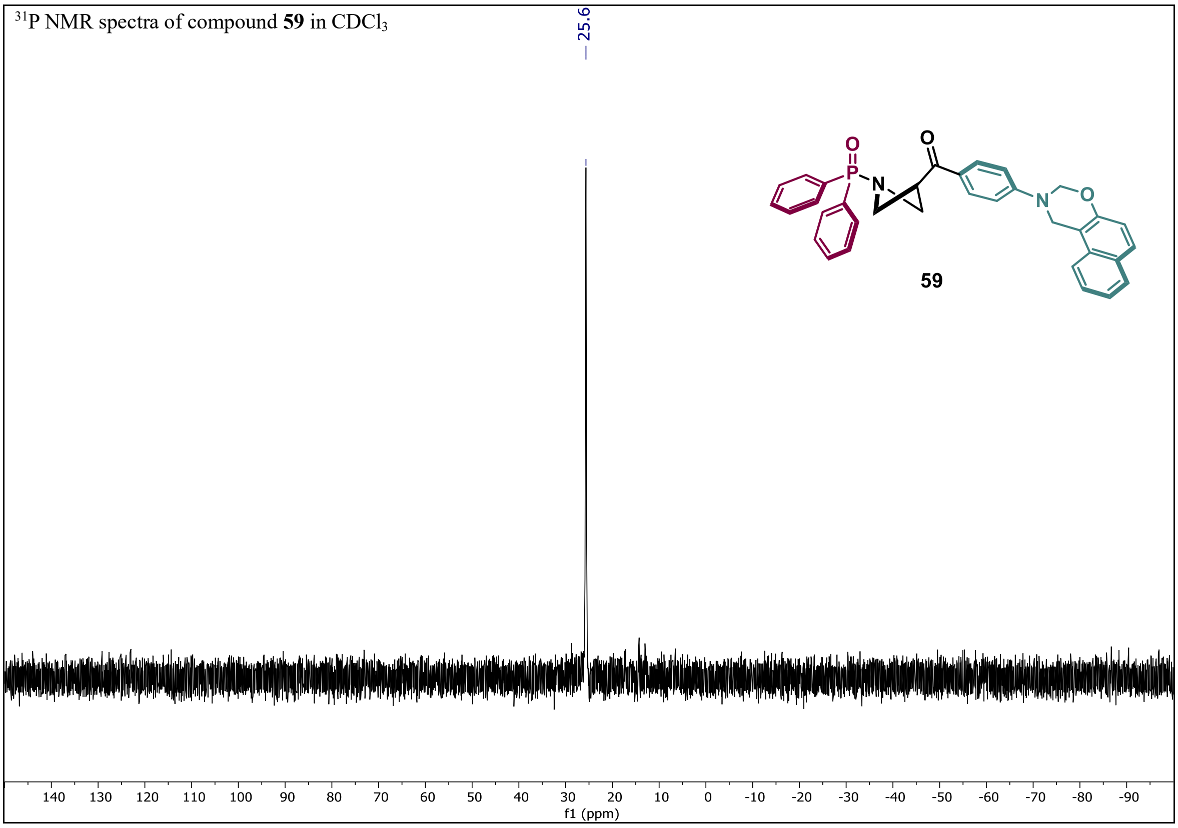

^{31}P NMR spectra of compound **59** in $CDCl_3$

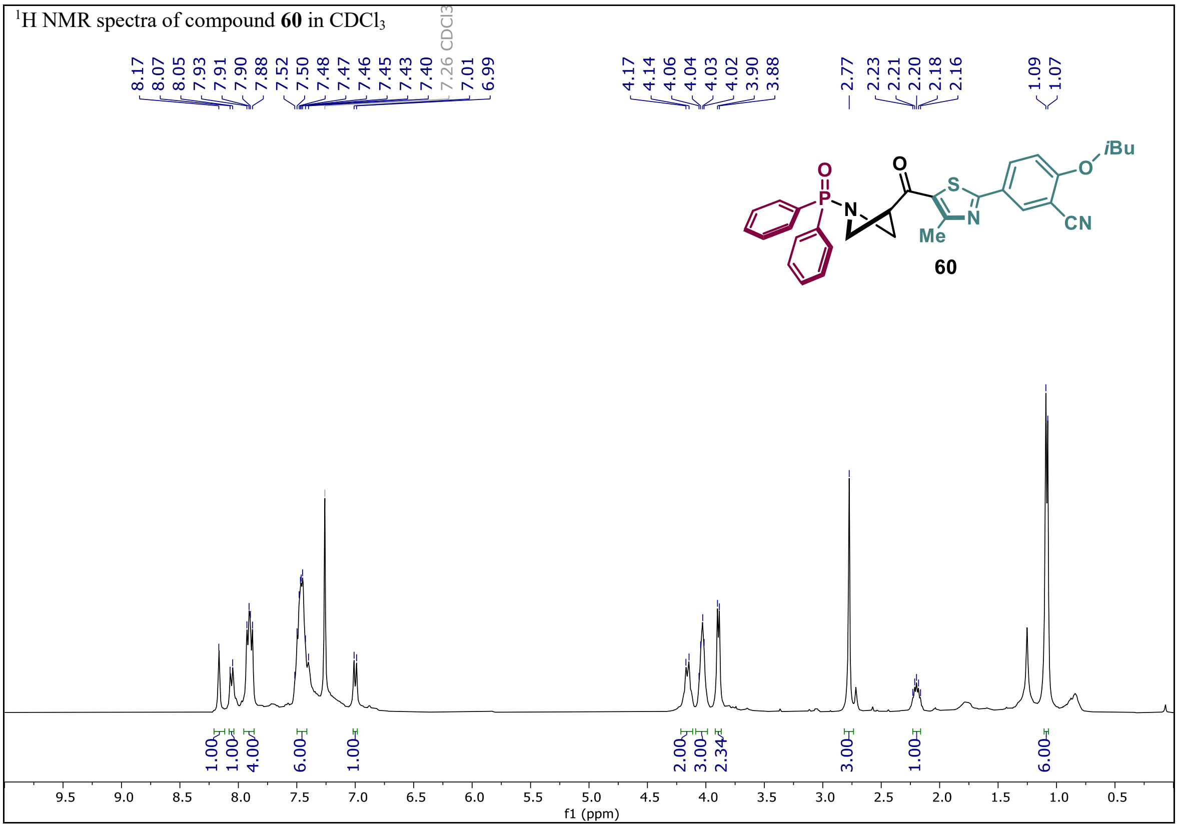
^{1}H NMR spectra of compound **60** in $CDCl_3$
8.17 8.07 8.05 7.93 7.91 7.90 7.88 7.52 7.50 7.48 7.47 7.46 7.45 7.43 7.40 7.26 CDCl3 7.01 6.99
4.17 4.14 4.06 4.04 4.03 4.02 3.90 3.88
2.77 2.23 2.21 2.20 2.18 2.16
1.09 1.07
O
P
N
O
S
N
Me
O
iBu
CN
60
1.00 1.00 4.00 6.00 1.00
2.00 3.00 2.34
3.00
1.00
6.00
9.5 9.0 8.5 8.0 7.5 7.0 6.5 6.0 5.5 5.0 4.5 4.0 3.5 3.0 2.5 2.0 1.5 1.0 0.5
f1 (ppm)

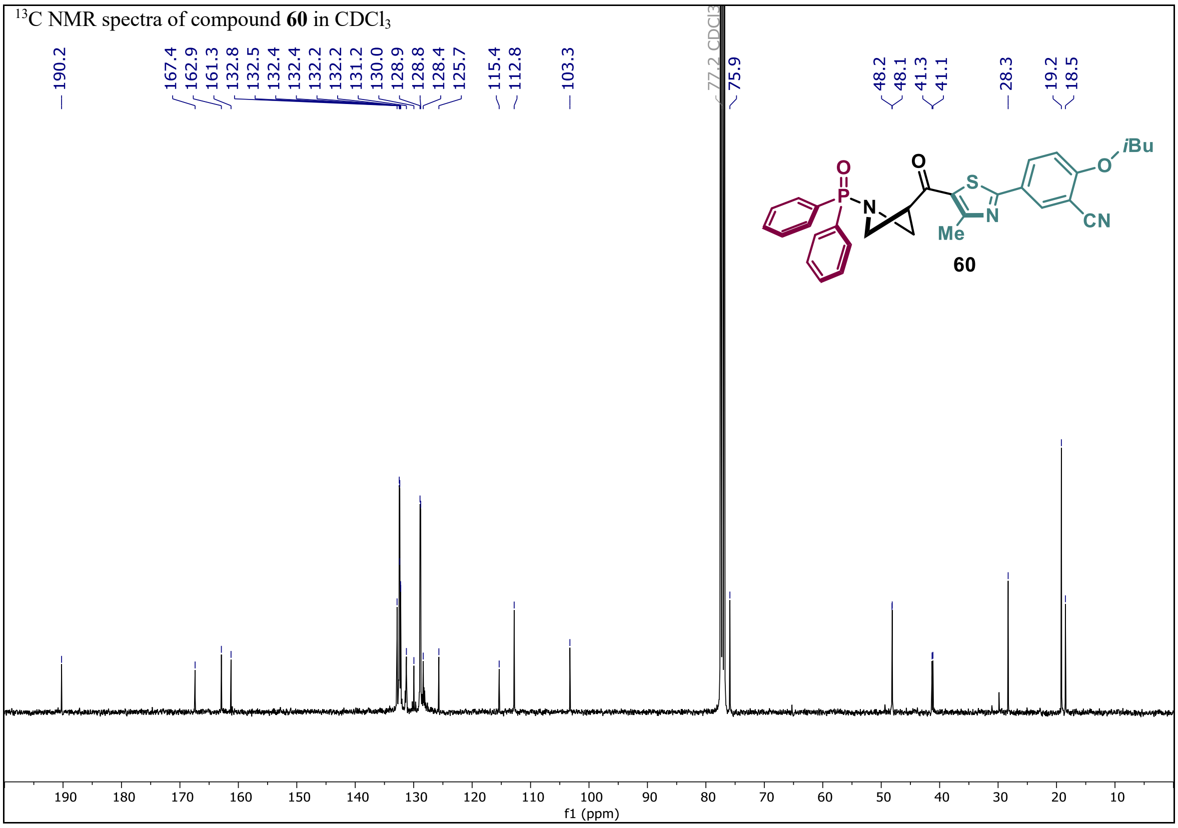
^{13}C NMR spectra of compound **60** in $CDCl_3$
190.2
167.4 162.9 161.3 132.8 132.5 132.4 132.4 132.2 132.2 131.2 130.0 128.9 128.8 128.4 125.7
115.4 112.8
103.3
77.2 CDCl3 75.9
48.2 48.1 41.3 41.1
28.3
19.2 18.5
O
P
N
O
S
N
Me
O
iBu
CN
60
190 180 170 160 150 140 130 120 110 100 90 80 70 60 50 40 30 20 10
f1 (ppm)

^{31}P NMR spectra of compound **60** in $CDCl_3$

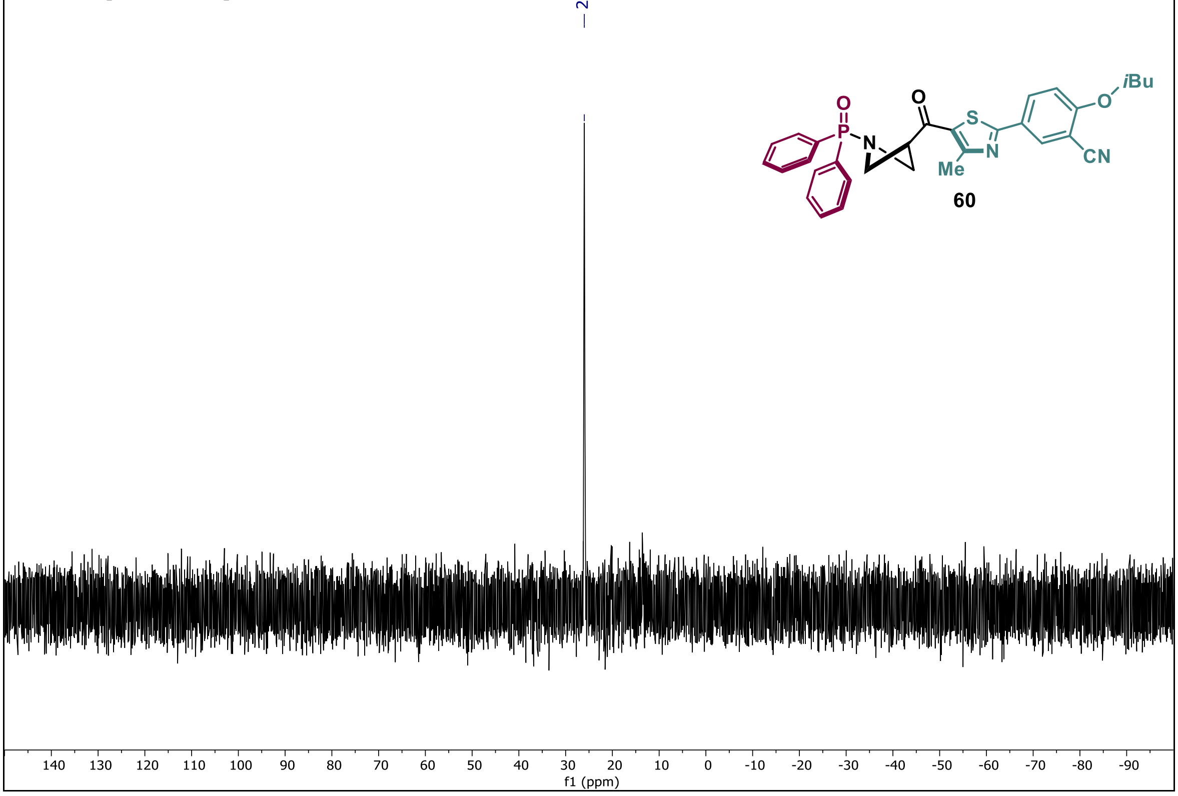

^{1}H NMR spectra of compound **61** in $CDCl_3$

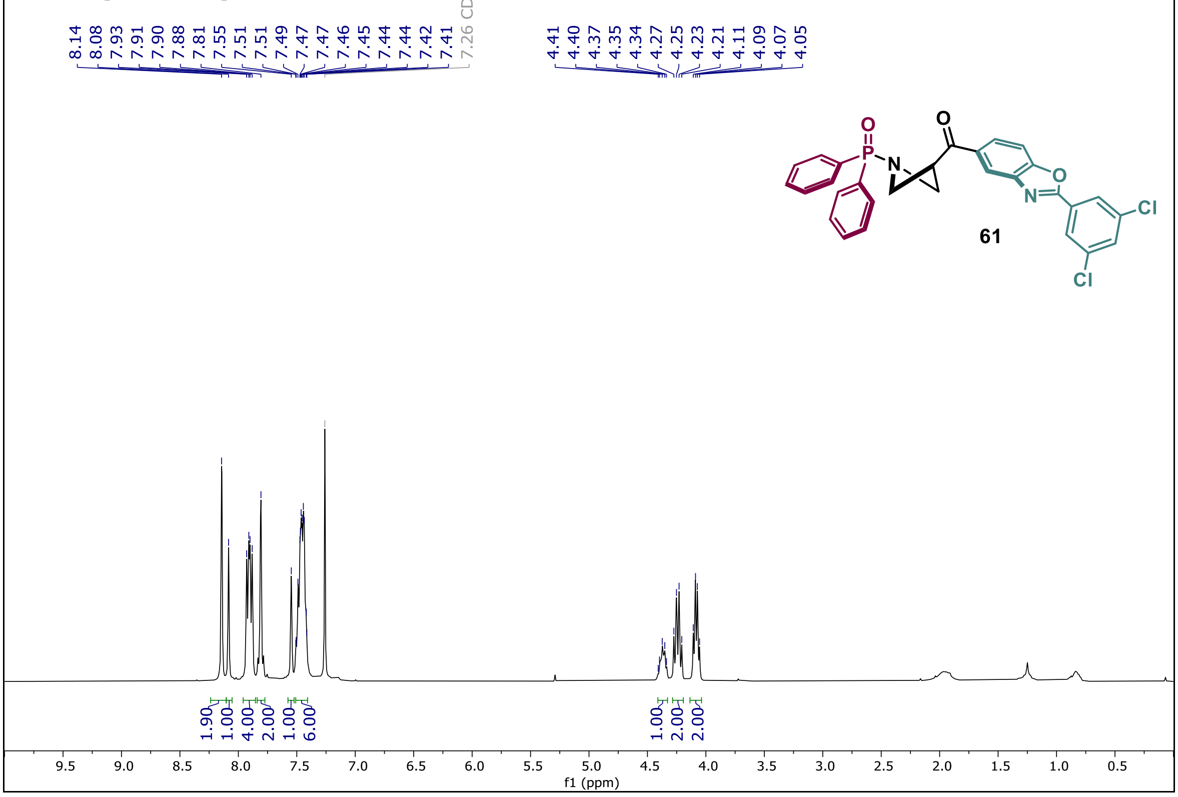

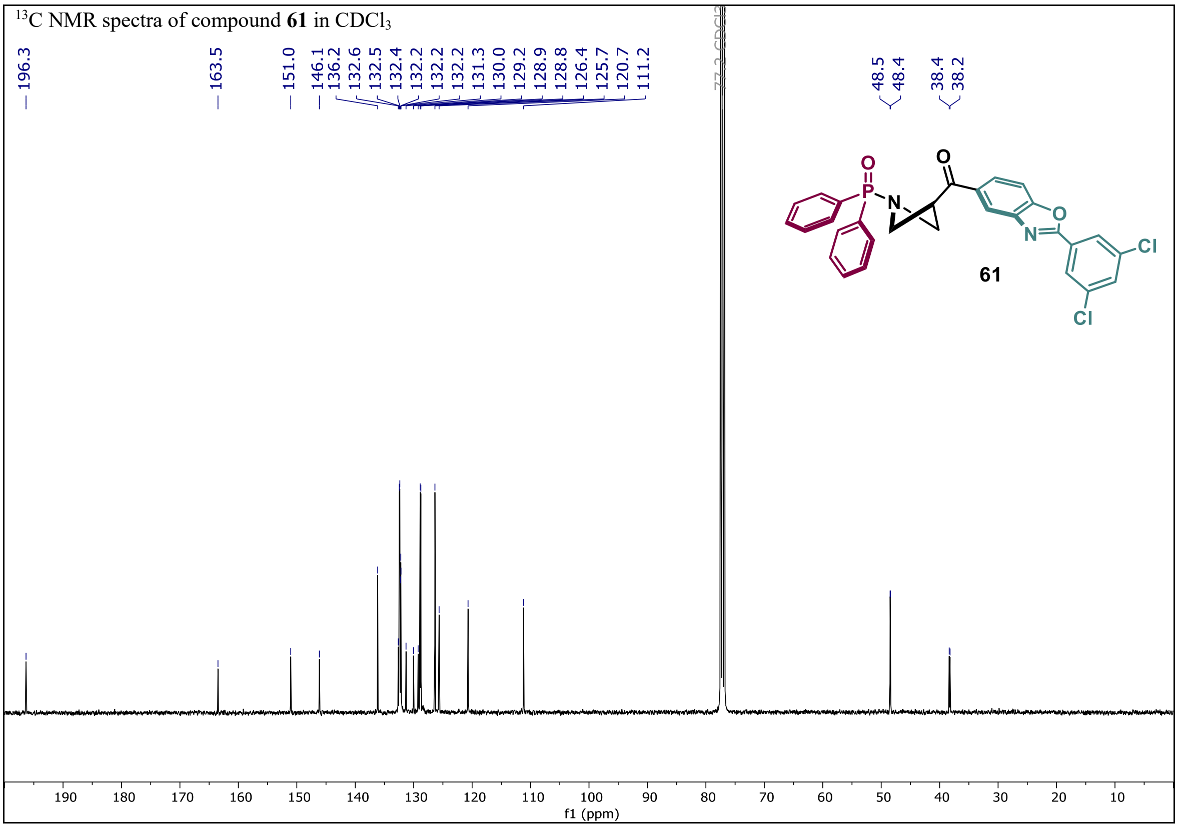

^{13}C NMR spectra of compound **61** in $CDCl_3$
196.3
163.5
151.0
146.1
136.2
132.6
132.5
132.4
132.2
132.2
132.2
131.3
130.0
129.2
128.9
128.8
126.4
125.7
120.7
111.2
48.5
48.4
38.4
38.2
61
f1 (ppm)

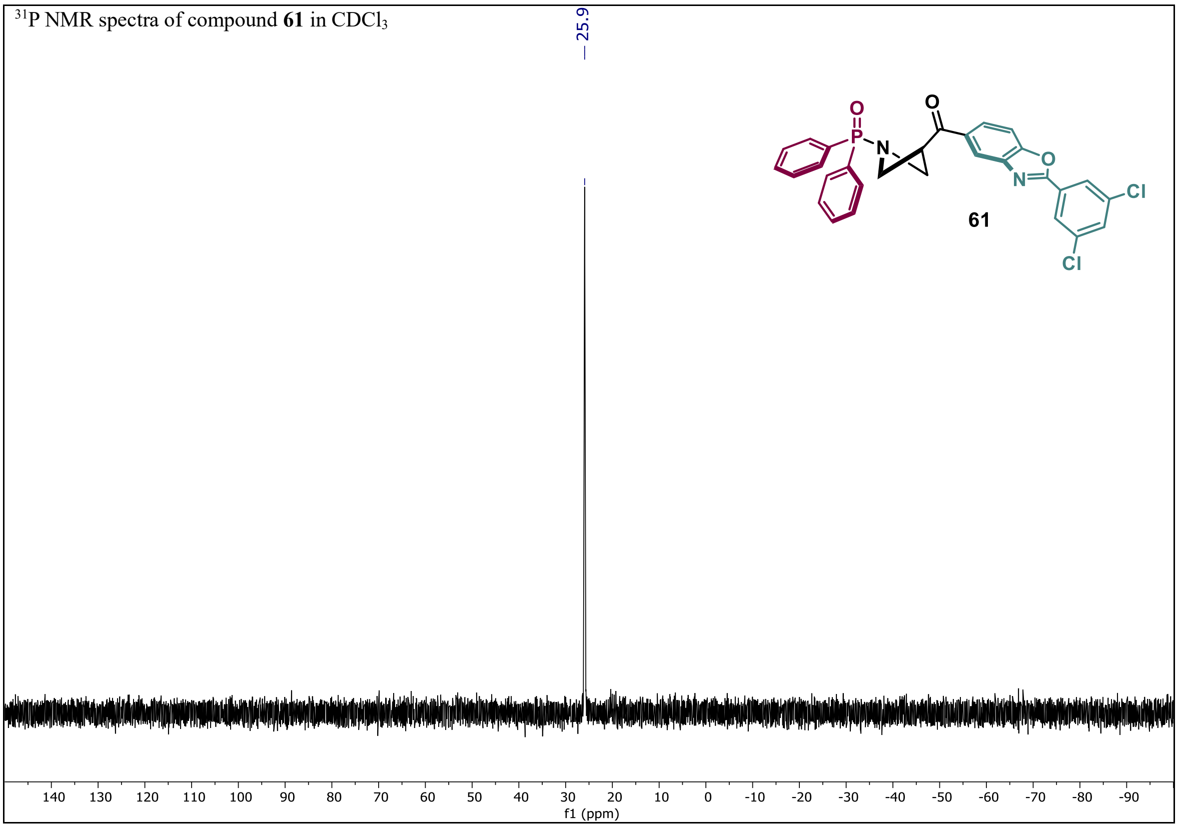

^{31}P NMR spectra of compound **61** in $CDCl_3$
25.9
61
f1 (ppm)

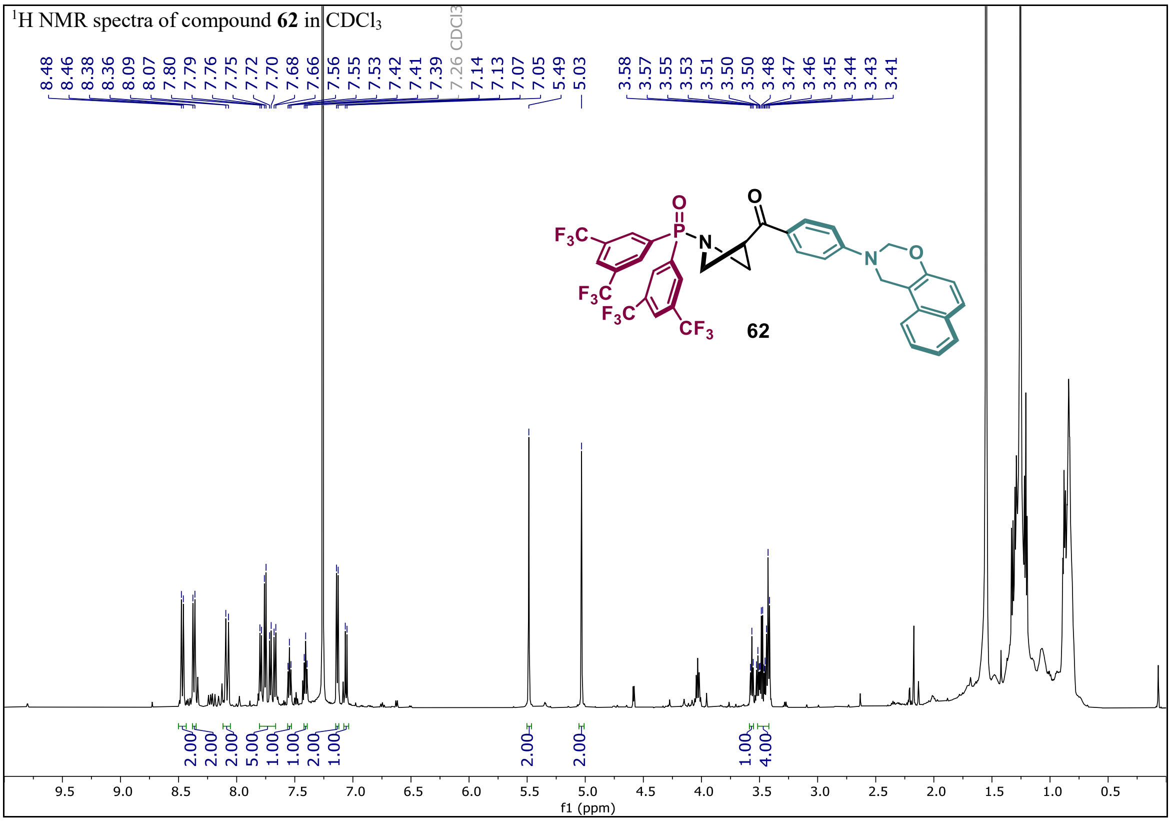

^{1}H NMR spectra of compound 62 in CDCl3
8.48 8.46 8.38 8.36 8.09 8.07 7.80 7.79 7.76 7.75 7.72 7.70 7.68 7.66 7.56 7.55 7.53 7.42 7.41 7.39 7.26 CDCl3 7.14 7.13 7.07 7.05 5.49 5.03
3.58 3.57 3.55 3.53 3.51 3.50 3.50 3.48 3.47 3.46 3.45 3.44 3.43 3.41
2.00 2.00 2.00 5.00 1.00 1.00 2.00 1.00 2.00 2.00 1.00 4.00
62
f1 (ppm)

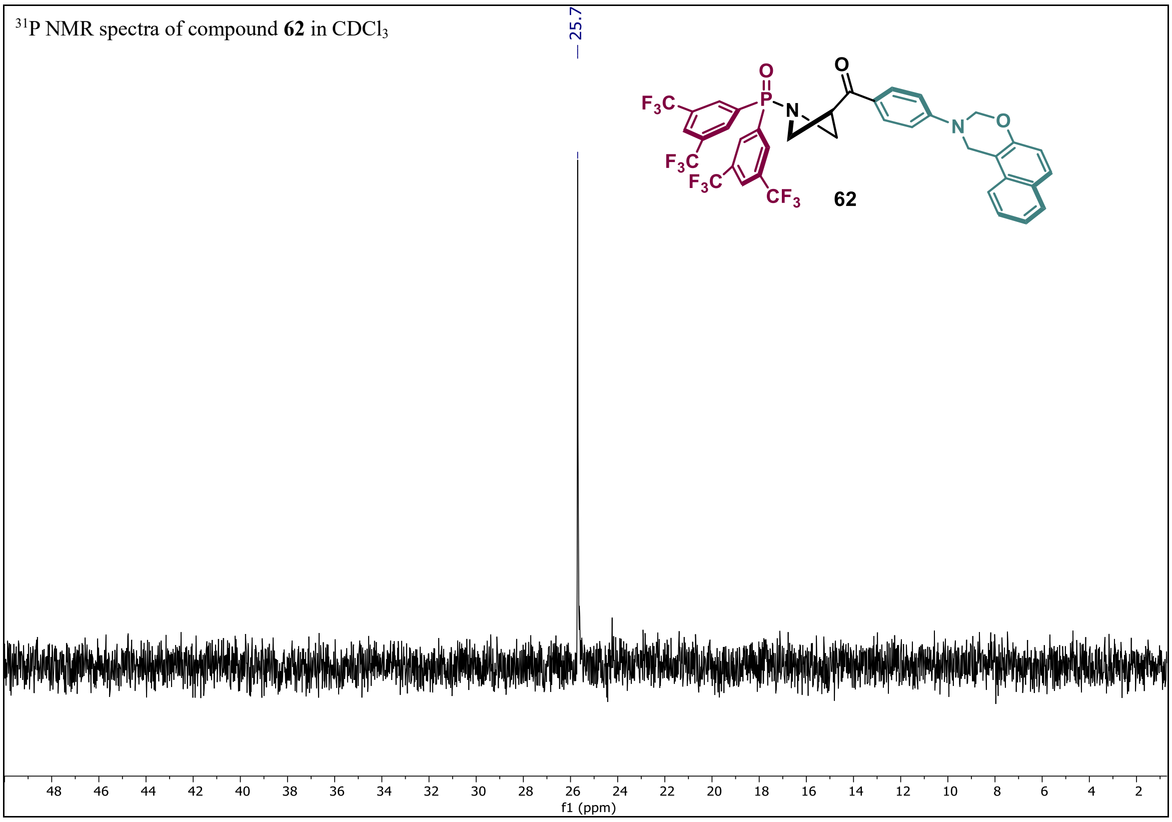

^{31}P NMR spectra of compound 62 in CDCl3
25.7
62
f1 (ppm)

^{19}F NMR spectra of compound **62** in $CDCl_3$

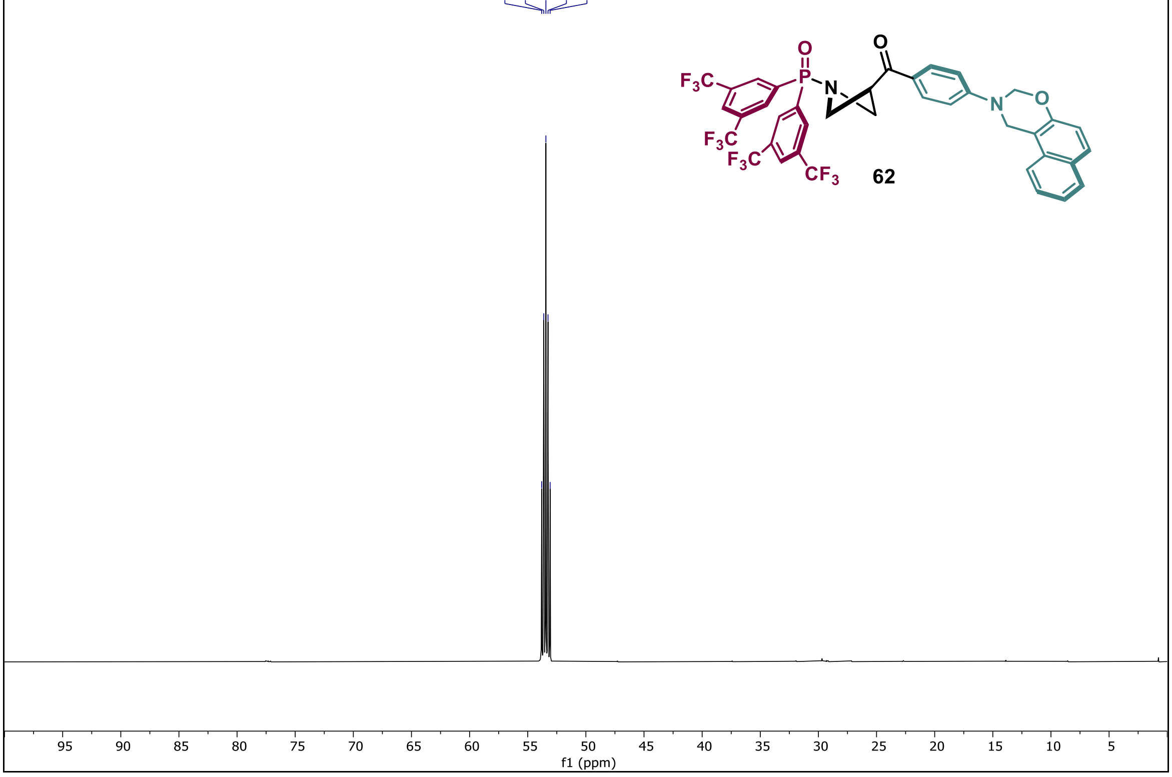

^{1}H NMR spectra of compound **63** in $CDCl_3$

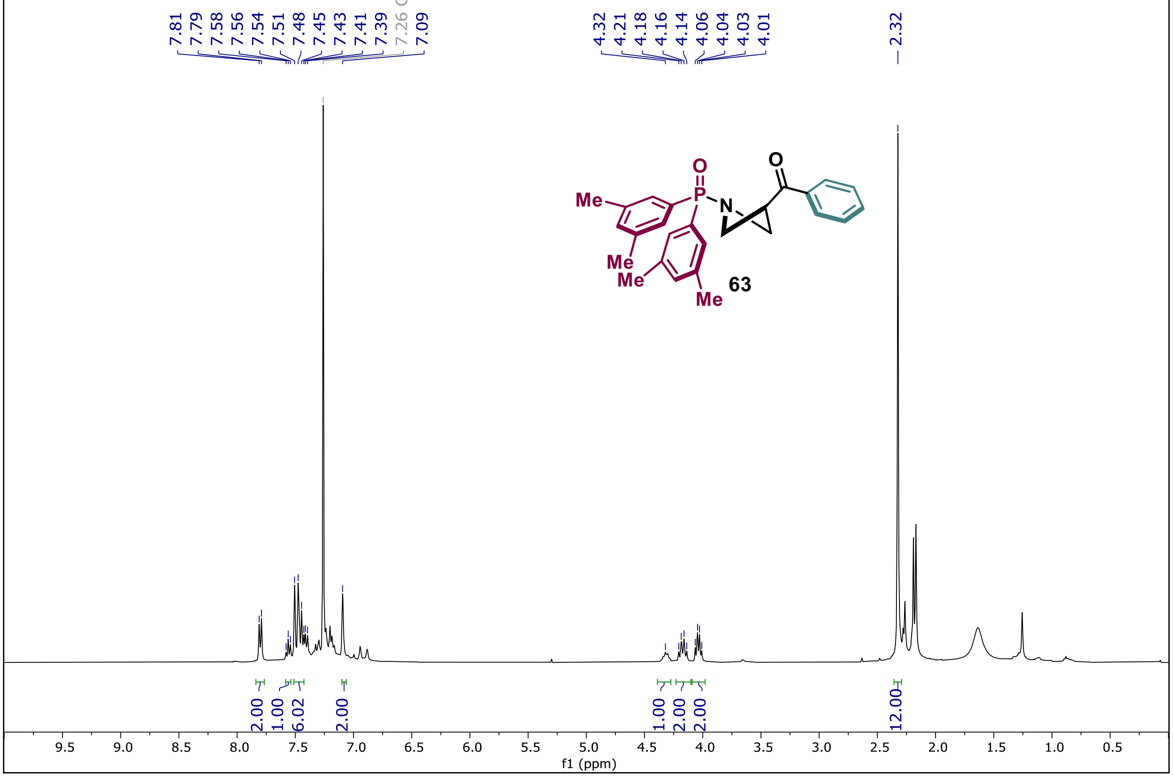

^{13}C NMR spectra of compound **63** in $CDCl_3$

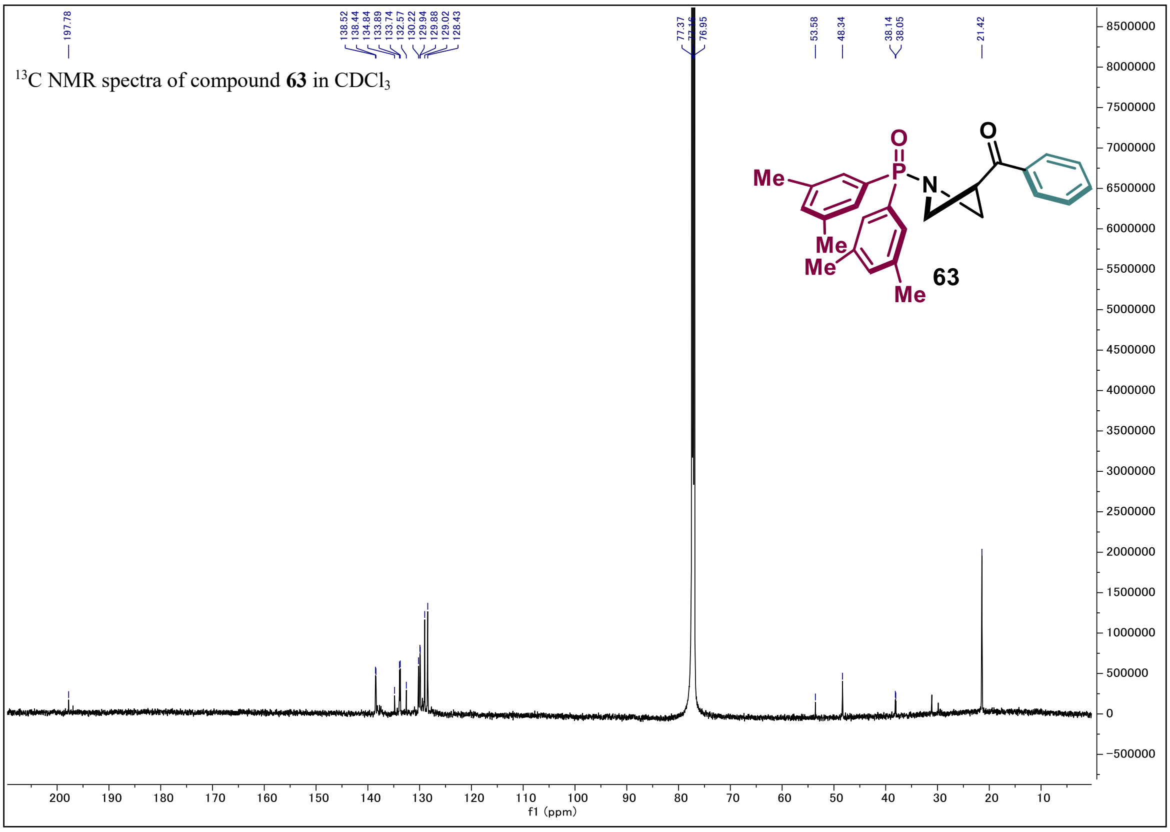

^{31}P NMR spectra of compound **63** in $CDCl_3$

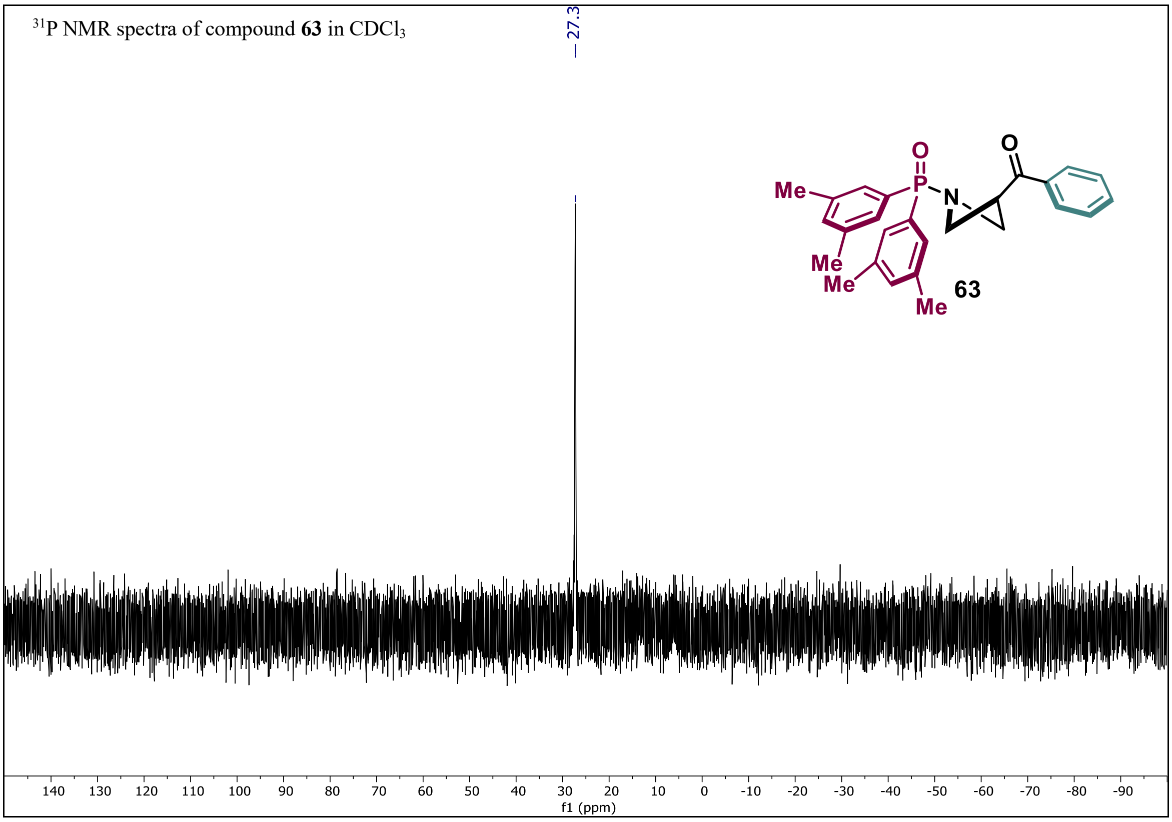

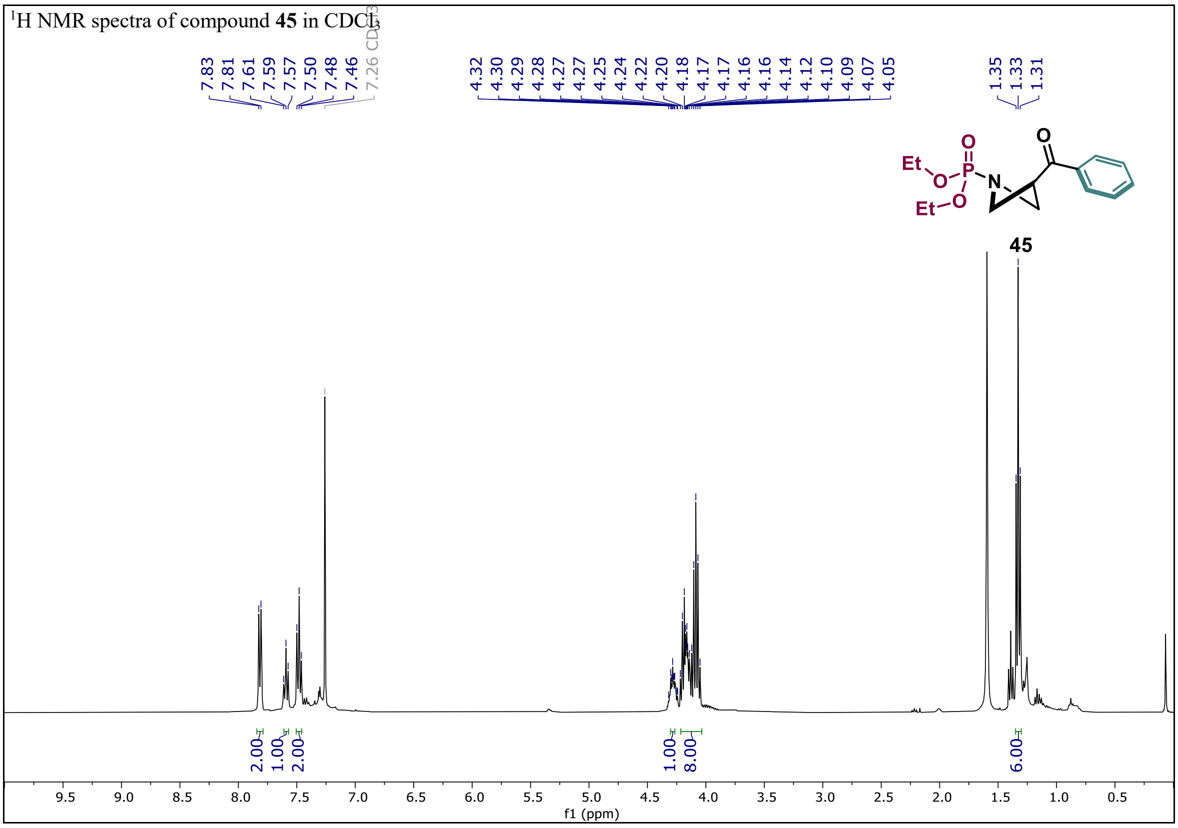

^{1}H NMR spectra of compound 45 in CDCl3
7.83
7.81
7.61
7.59
7.57
7.50
7.48
7.46
7.26 CDCl3
4.32
4.30
4.29
4.28
4.27
4.27
4.25
4.24
4.22
4.20
4.18
4.17
4.17
4.16
4.16
4.14
4.12
4.10
4.09
4.07
4.05
1.35
1.33
1.31
Et
O
P
O
N
O
Et
O
45
2.00
1.00
2.00
1.00
8.00
6.00
9.5
9.0
8.5
8.0
7.5
7.0
6.5
6.0
5.5
5.0
4.5
4.0
3.5
3.0
2.5
2.0
1.5
1.0
0.5
f1 (ppm)

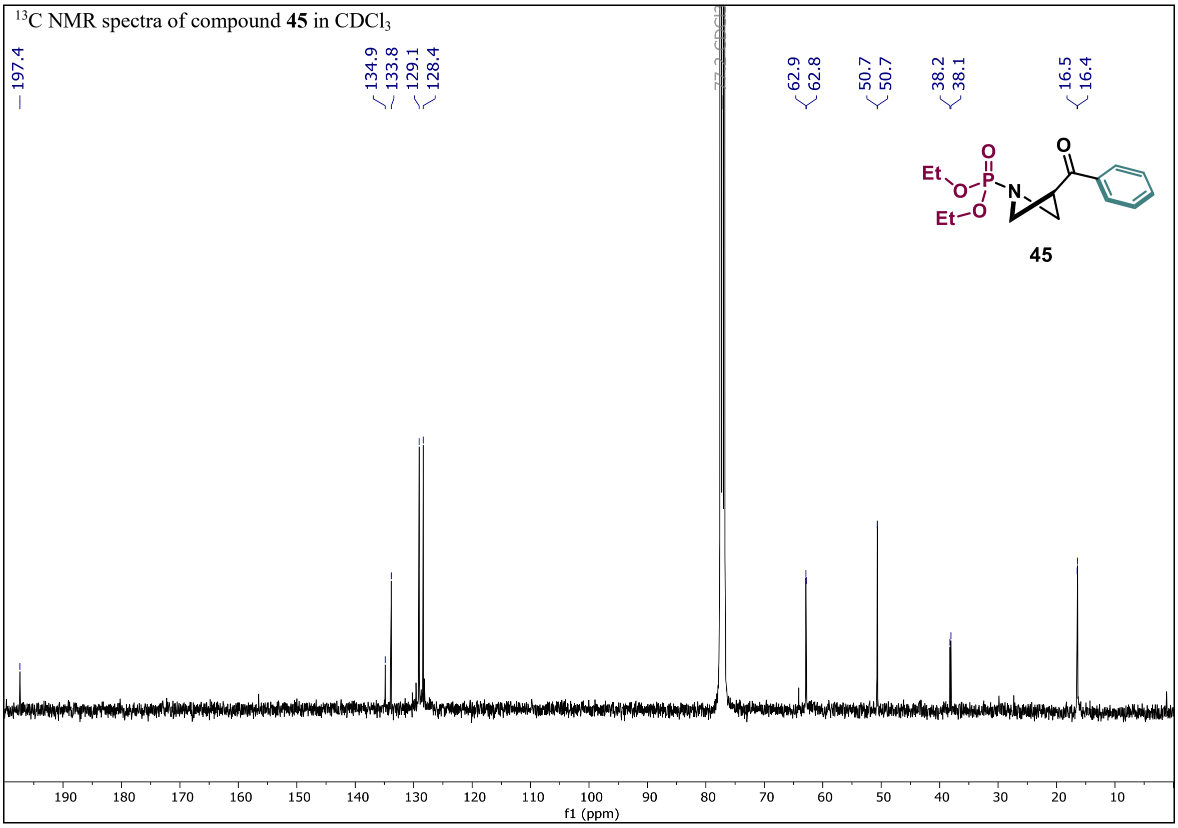

^{13}C NMR spectra of compound 45 in CDCl3
197.4
134.9
133.8
129.1
128.4
62.9
62.8
50.7
50.7
38.2
38.1
16.5
16.4
Et
O
P
O
N
O
Et
O
45
190
180
170
160
150
140
130
120
110
100
90
80
70
60
50
40
30
20
10
f1 (ppm)

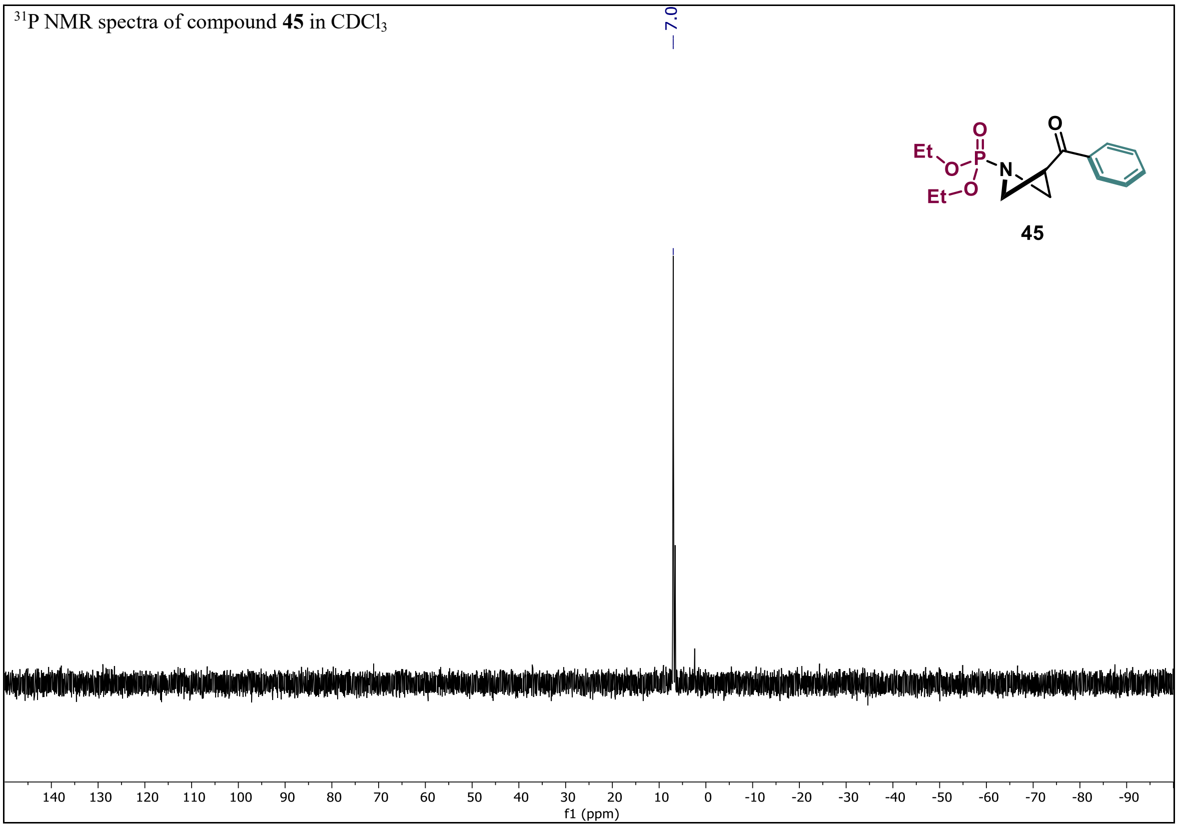

^{31}P NMR spectra of compound **45** in $CDCl_3$

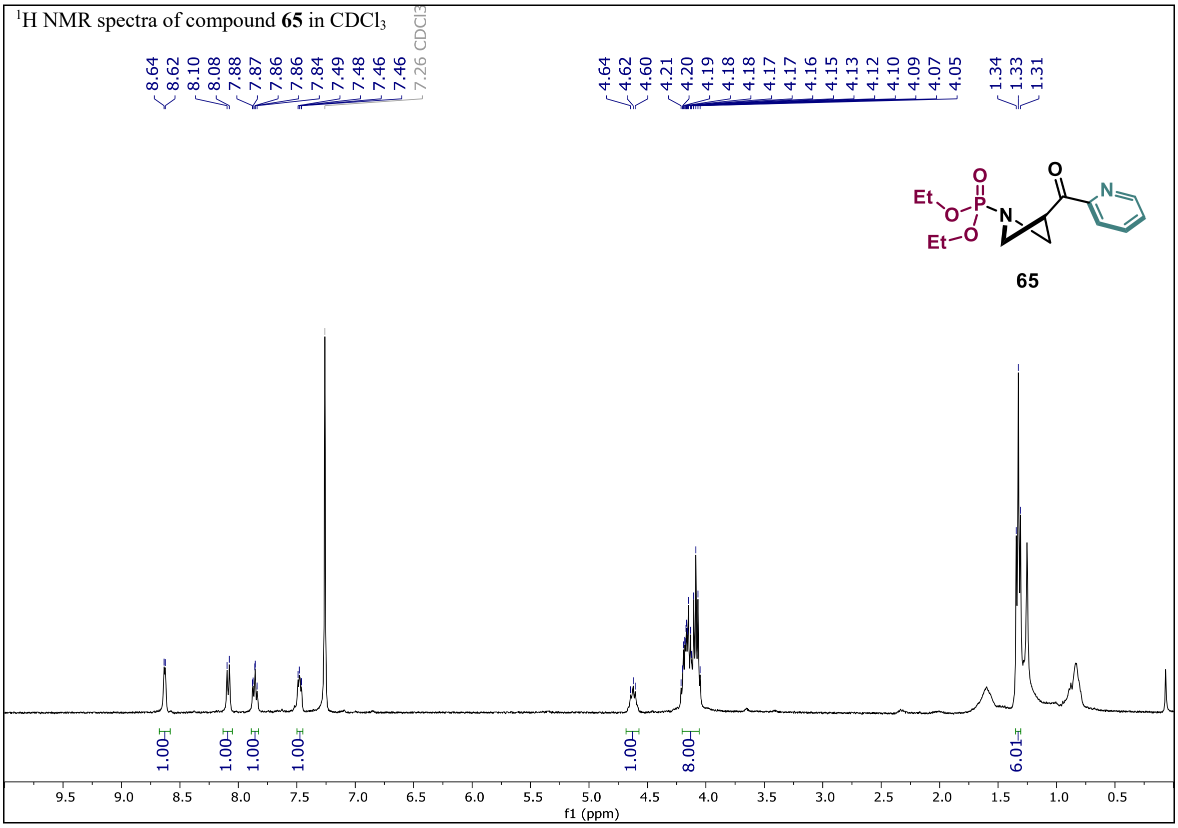

^{1}H NMR spectra of compound **65** in $CDCl_3$

^{13}C NMR spectra of compound **65** in $CDCl_3$

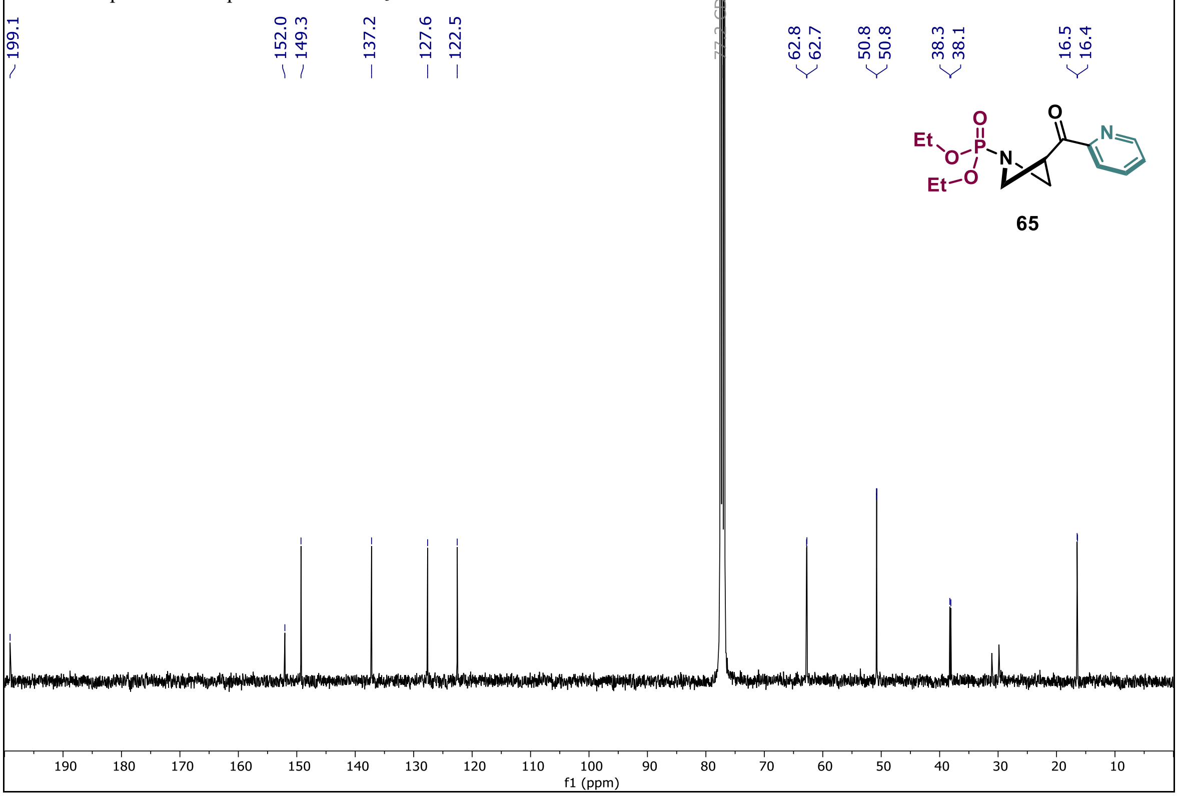

^{31}P NMR spectra of compound **65** in $CDCl_3$

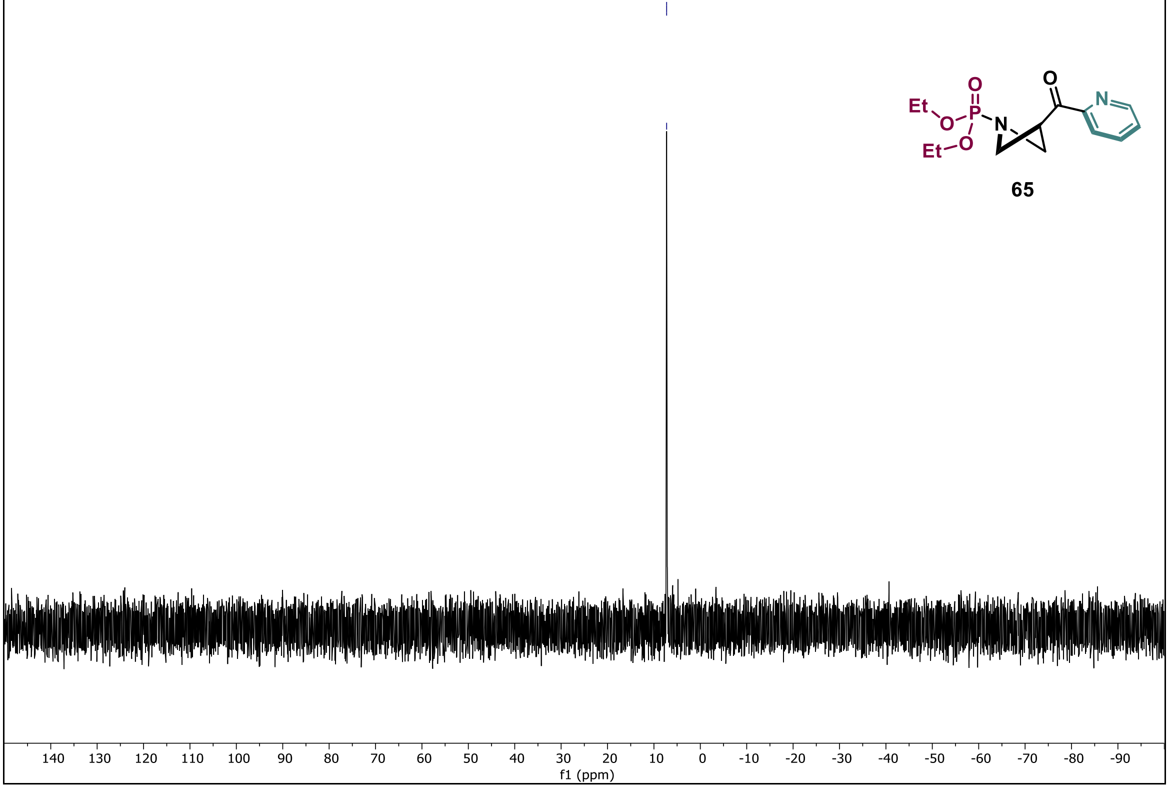

^1H NMR spectra of compound **66** in $CDCl_3$

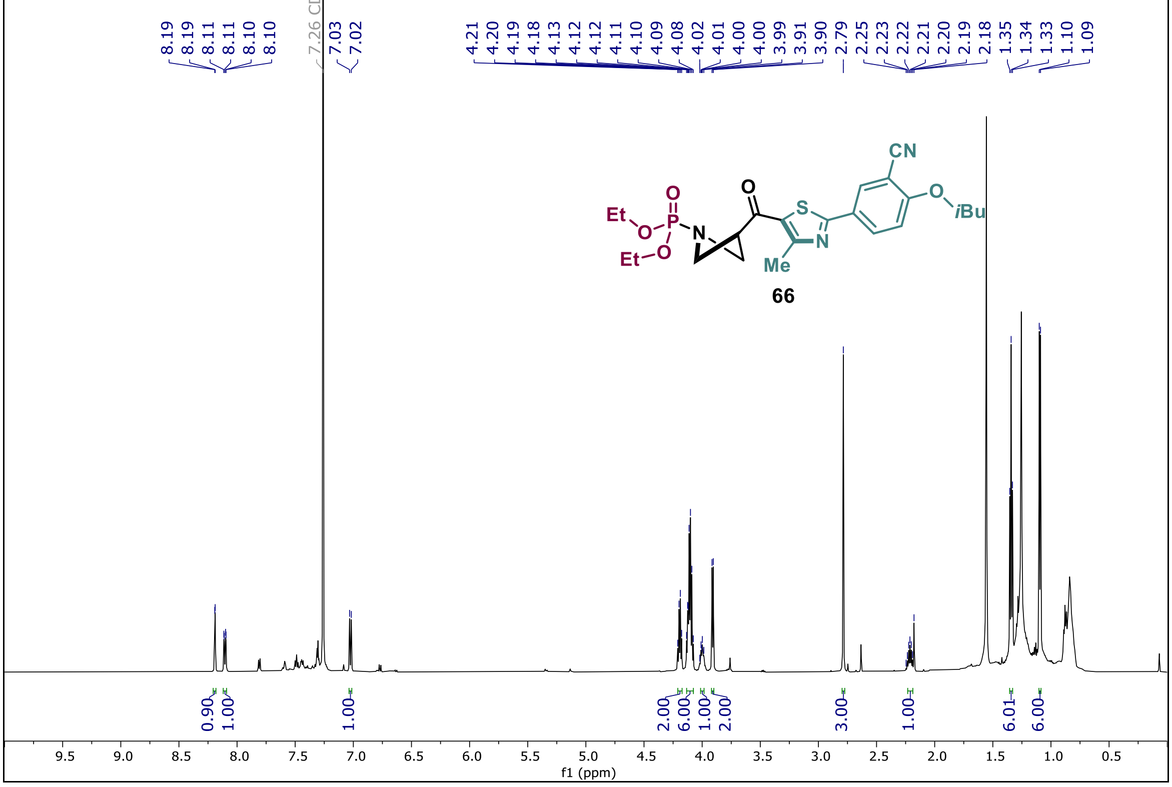

^{13}C NMR spectra of compound **66** in $CDCl_3$

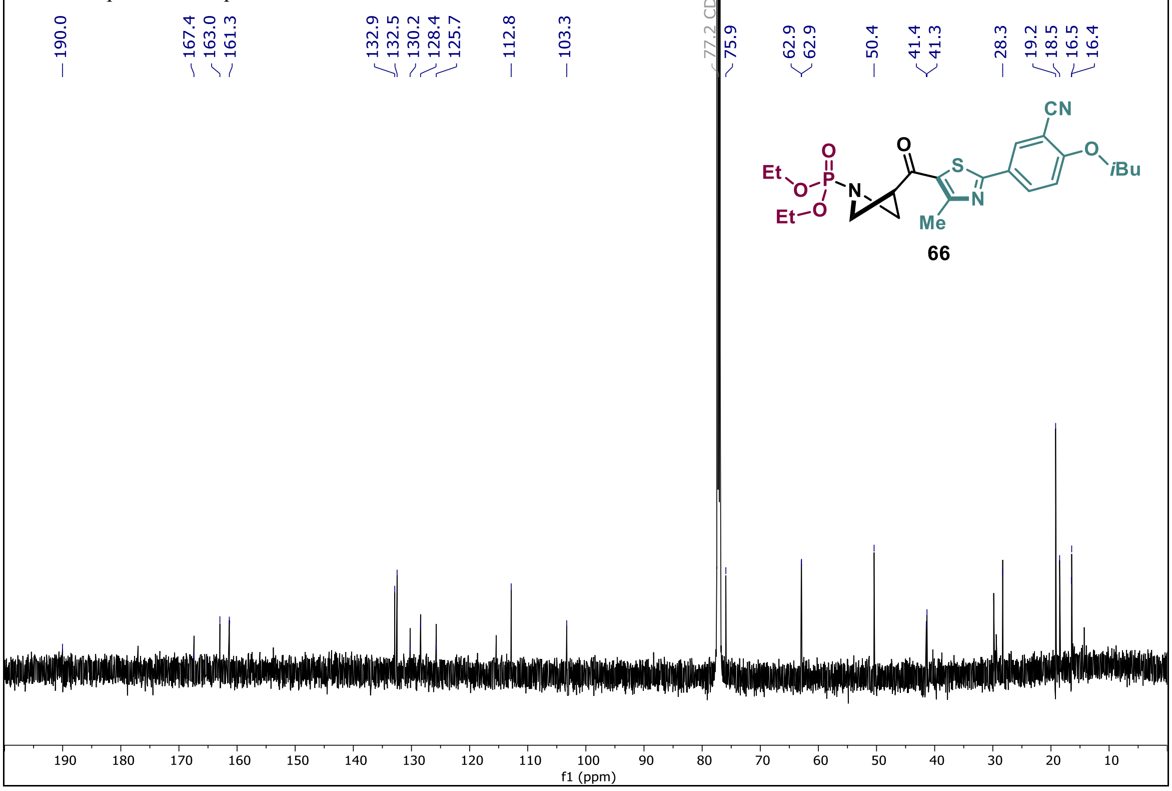

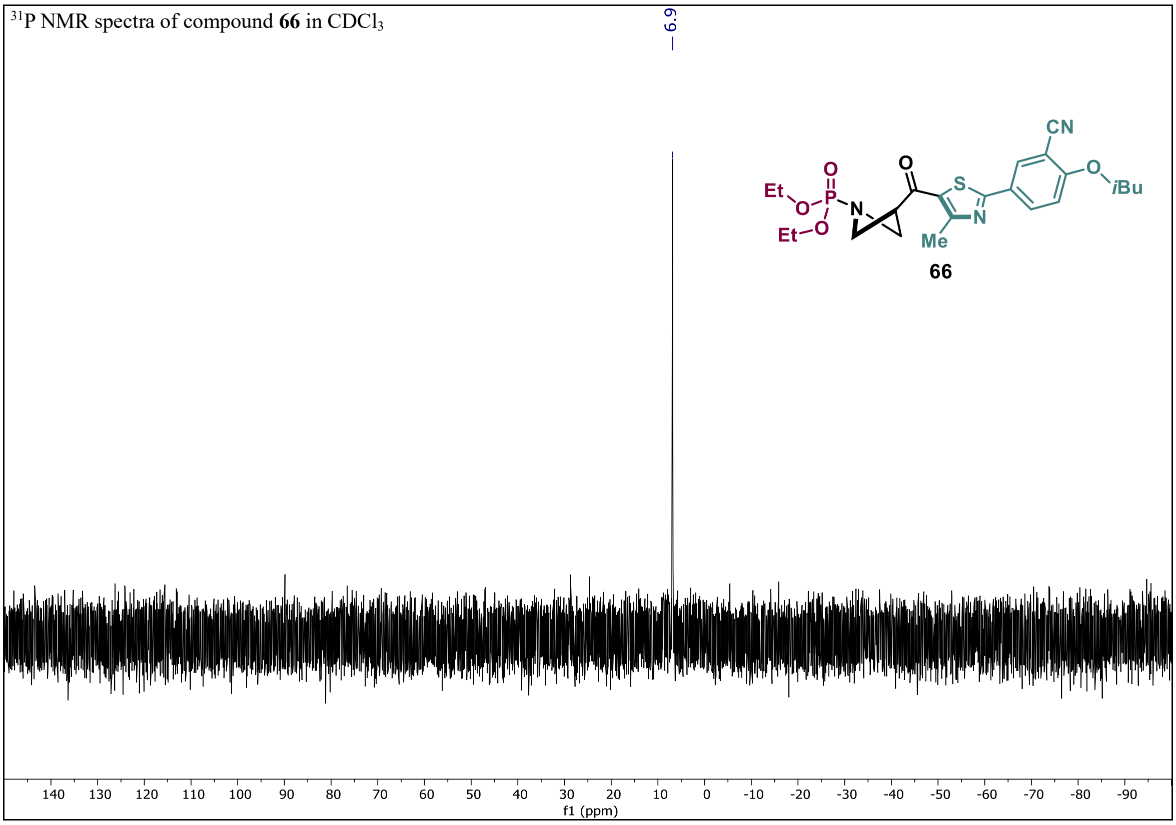

^{31}P NMR spectra of compound **66** in $CDCl_3$
6.9
Et
O
O
P
N
O
S
CN
O
iBu
Et
O
N
Me
66
140 130 120 110 100 90 80 70 60 50 40 30 20 10 0 -10 -20 -30 -40 -50 -60 -70 -80 -90
f1 (ppm)

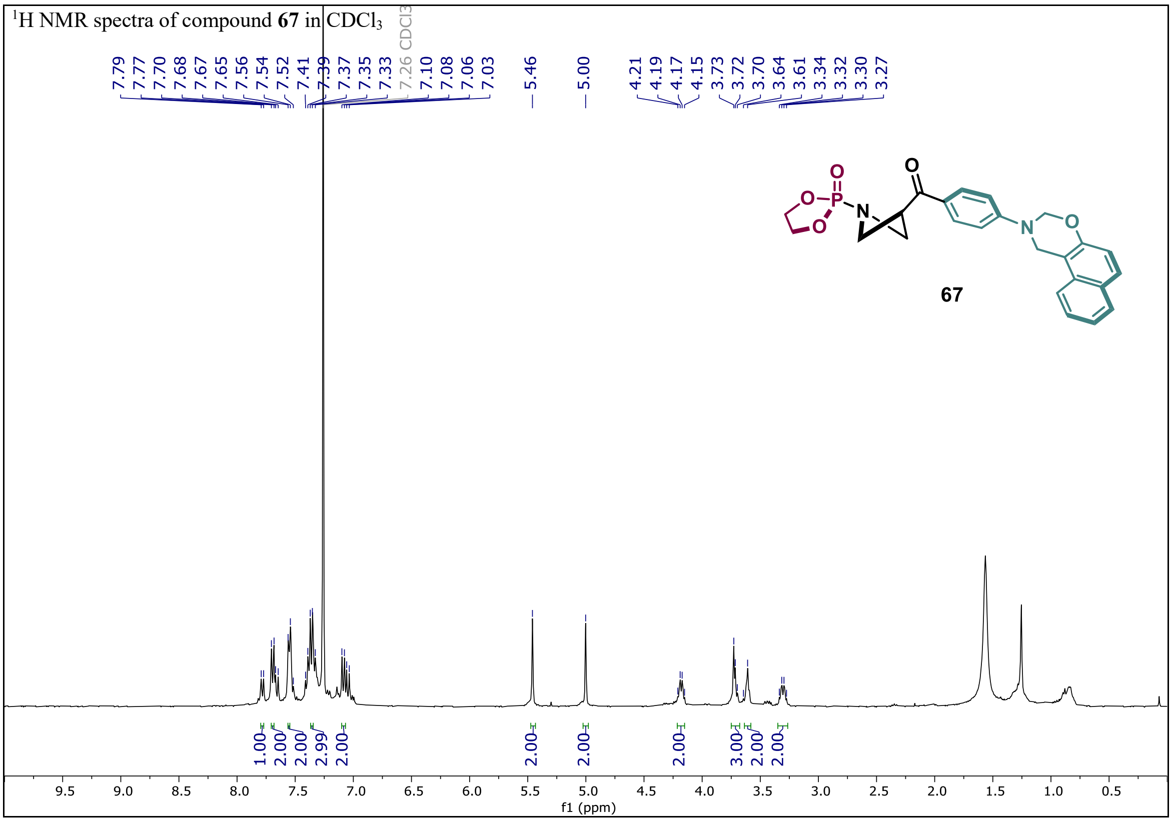

^{1}H NMR spectra of compound **67** in $CDCl_3$
7.79 7.77 7.70 7.68 7.67 7.65 7.56 7.54 7.52 7.41 7.39 7.37 7.35 7.33 7.26 CDCl3 7.10 7.08 7.06 7.03
5.46
5.00
4.21 4.19 4.17 4.15 3.73 3.72 3.70 3.64 3.61 3.34 3.32 3.30 3.27
O
O
P
N
O
N
O
67
1.00 2.00 2.00 2.99 2.00
2.00
2.00
2.00
3.00 2.00 2.00
9.5 9.0 8.5 8.0 7.5 7.0 6.5 6.0 5.5 5.0 4.5 4.0 3.5 3.0 2.5 2.0 1.5 1.0 0.5
f1 (ppm)

^{13}C NMR spectra of compound **67** in $CDCl_3$

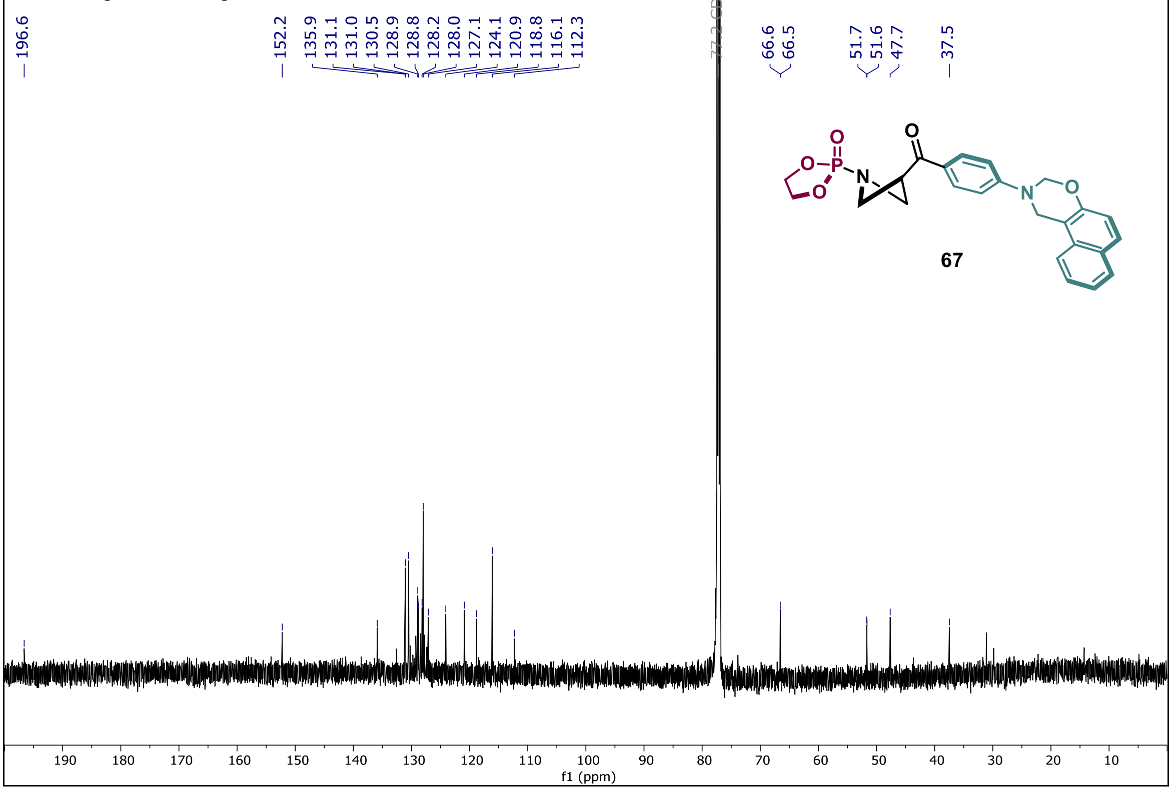

^{31}P NMR spectra of compound **67** in $CDCl_3$

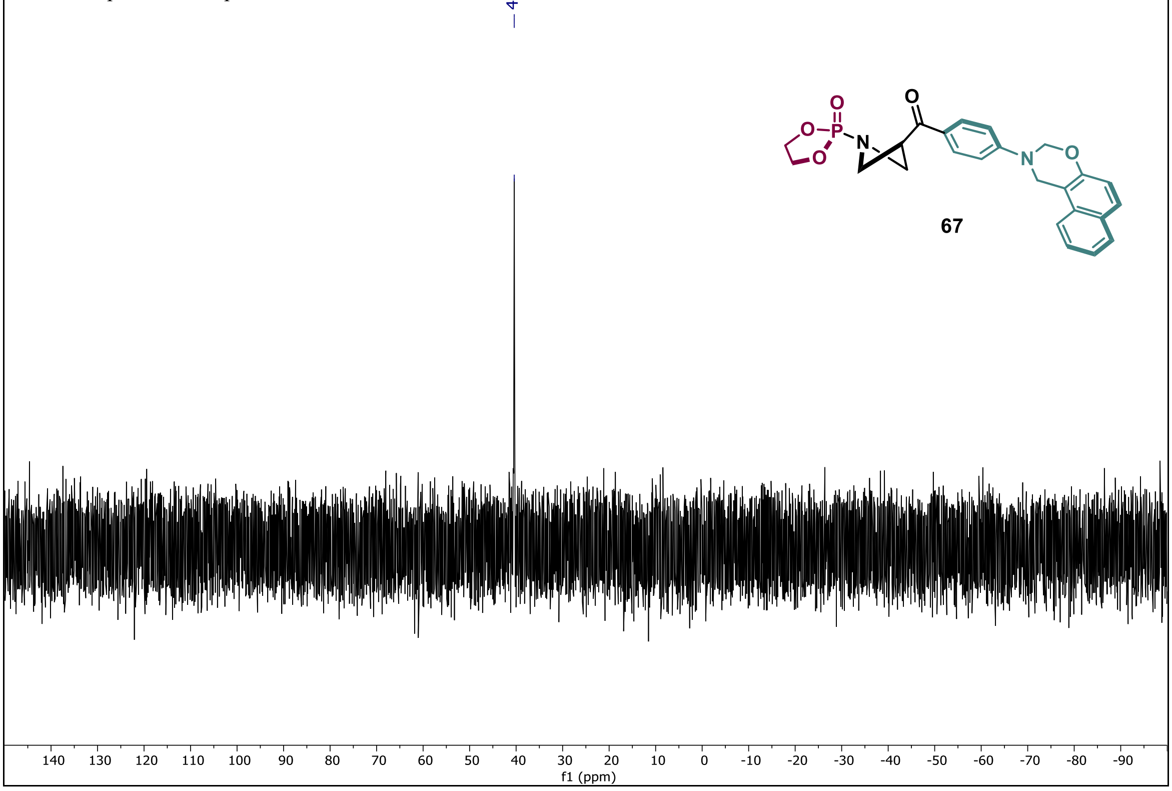

^{1}H NMR spectra of compound **70** in $CDCl_3$

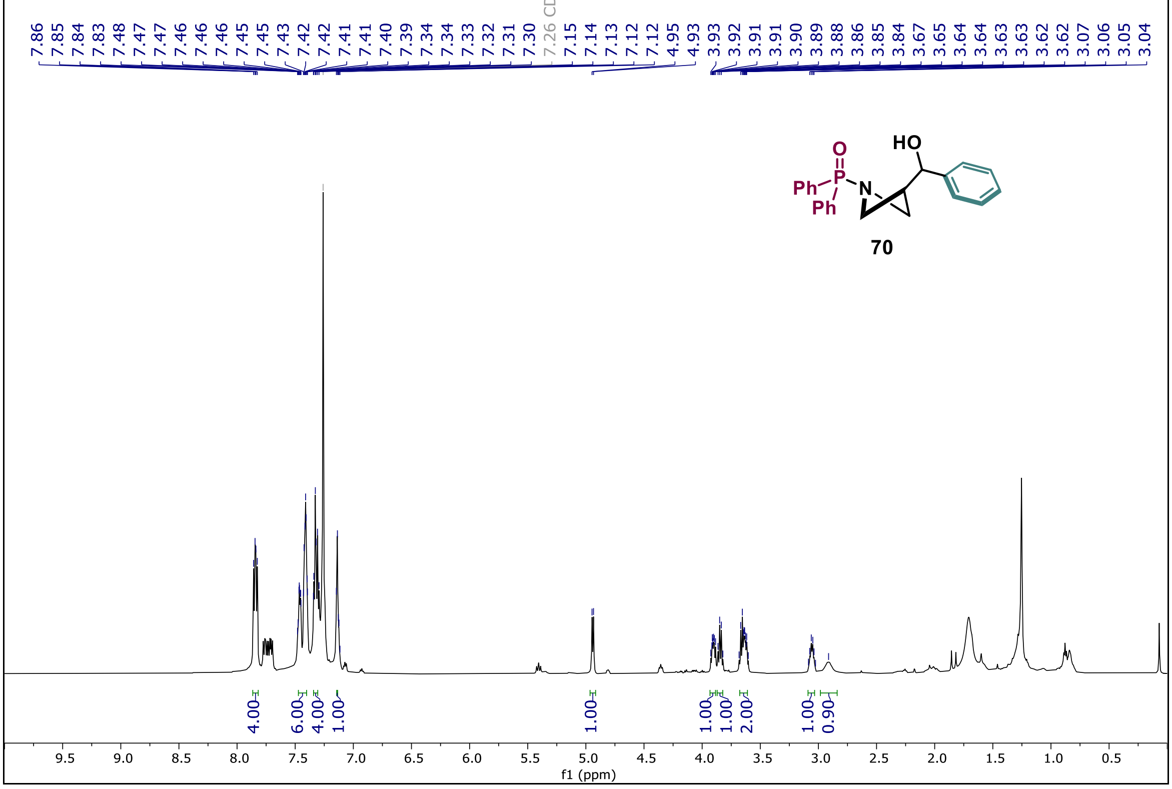

^{13}C NMR spectra of compound **70** in $CDCl_3$

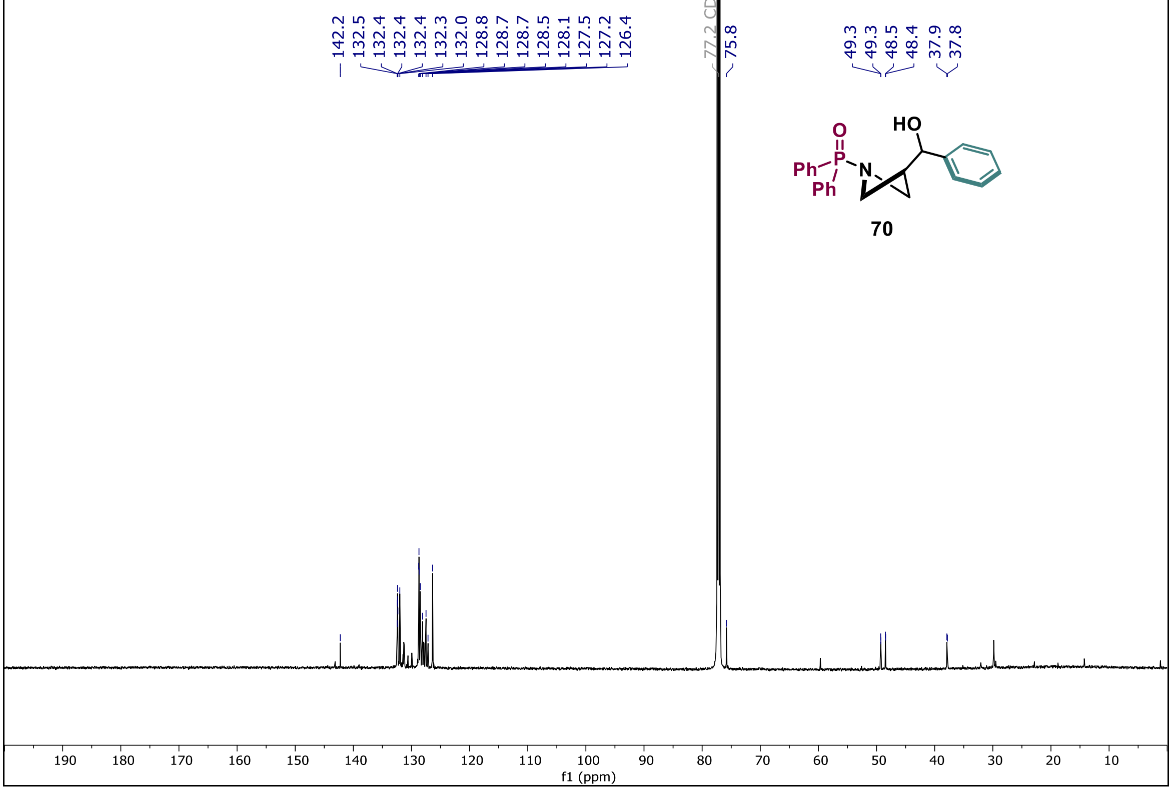

^{31}P NMR spectra of compound **70** in $CDCl_3$

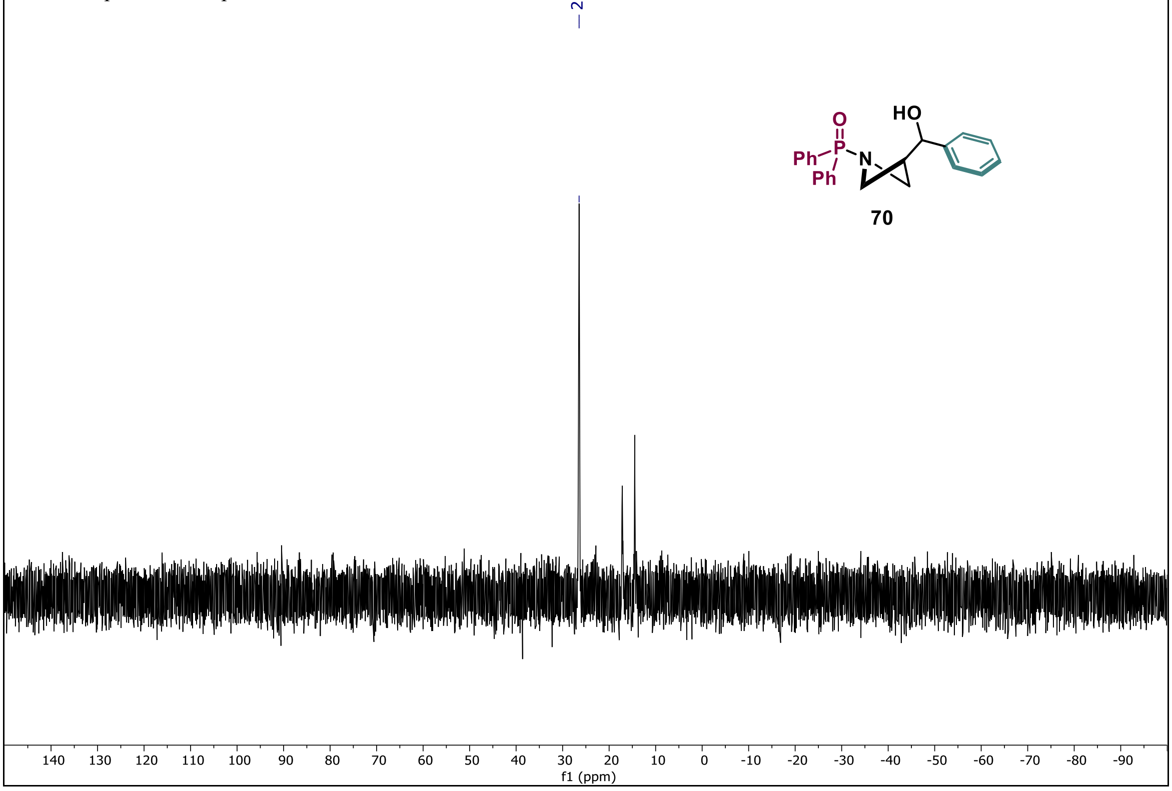

^{1}H NMR spectra of compound **72** in $CDCl_3$

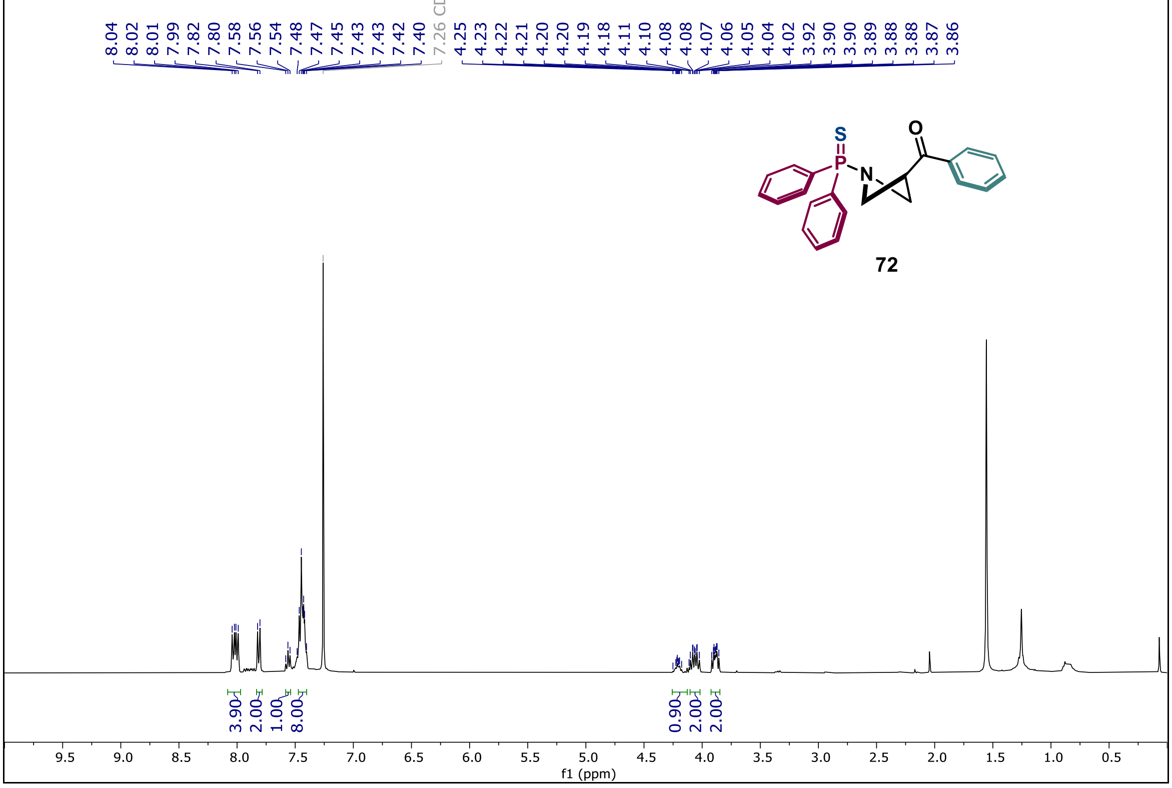

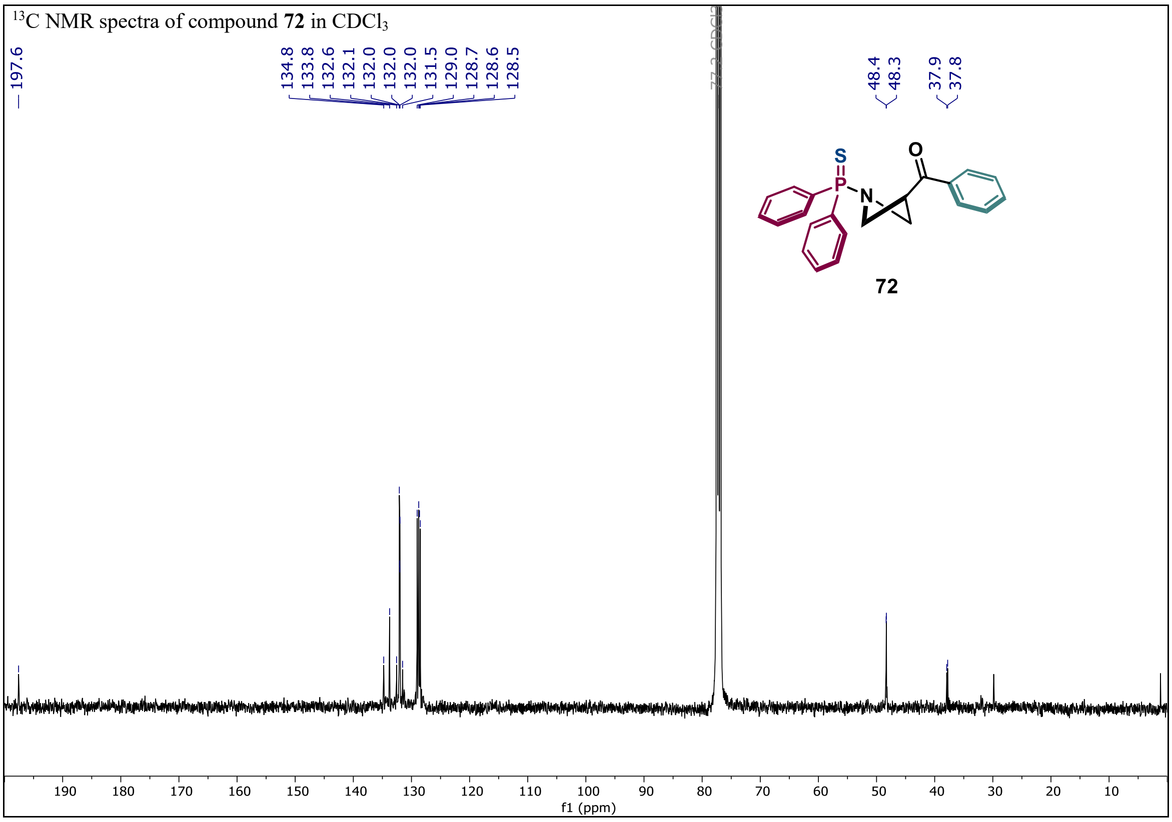

^{13}C NMR spectra of compound **72** in $CDCl_3$

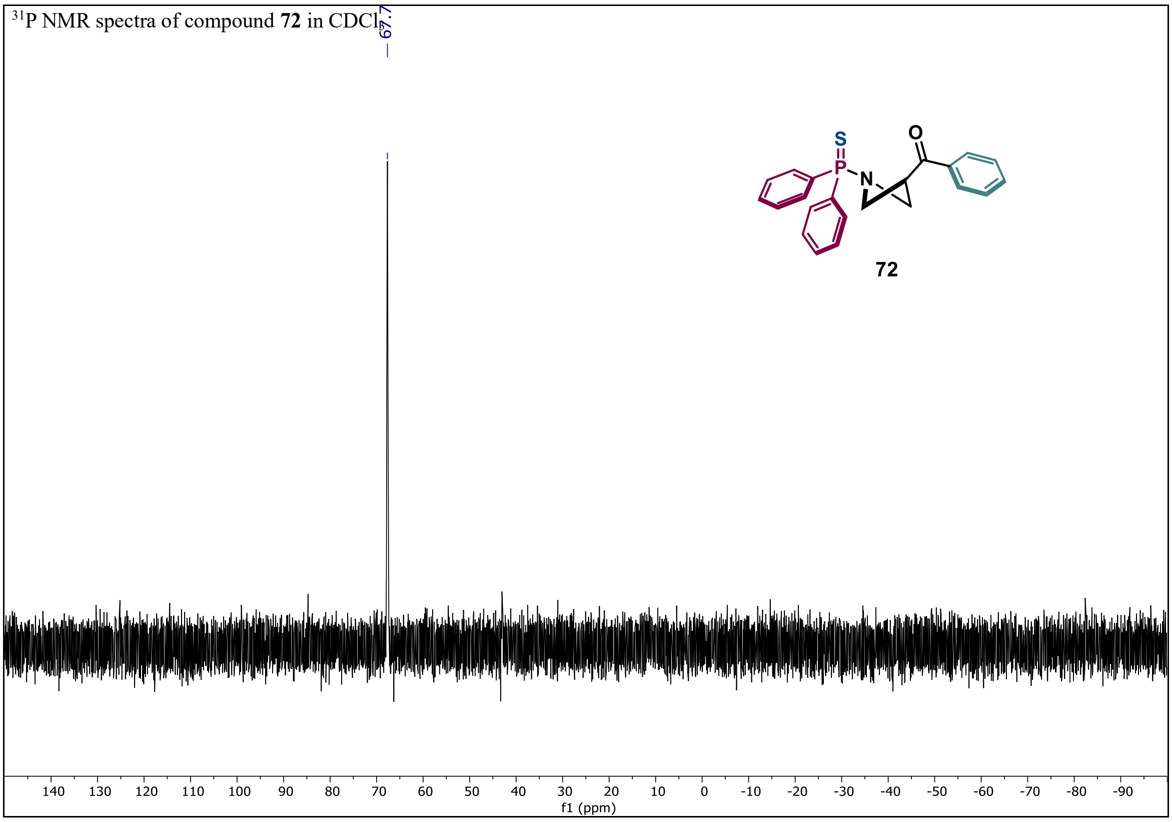

^{31}P NMR spectra of compound **72** in $CDCl_3$

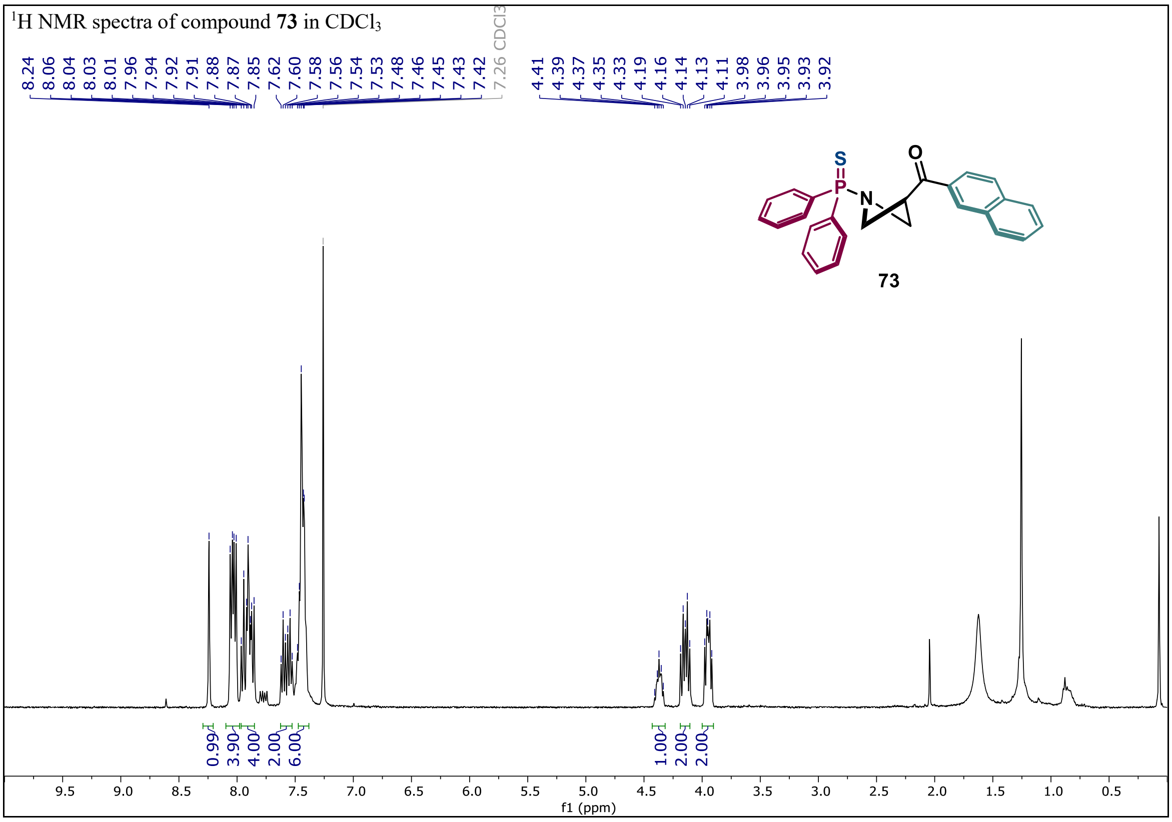

^{1}H NMR spectra of compound **73** in $CDCl_3$

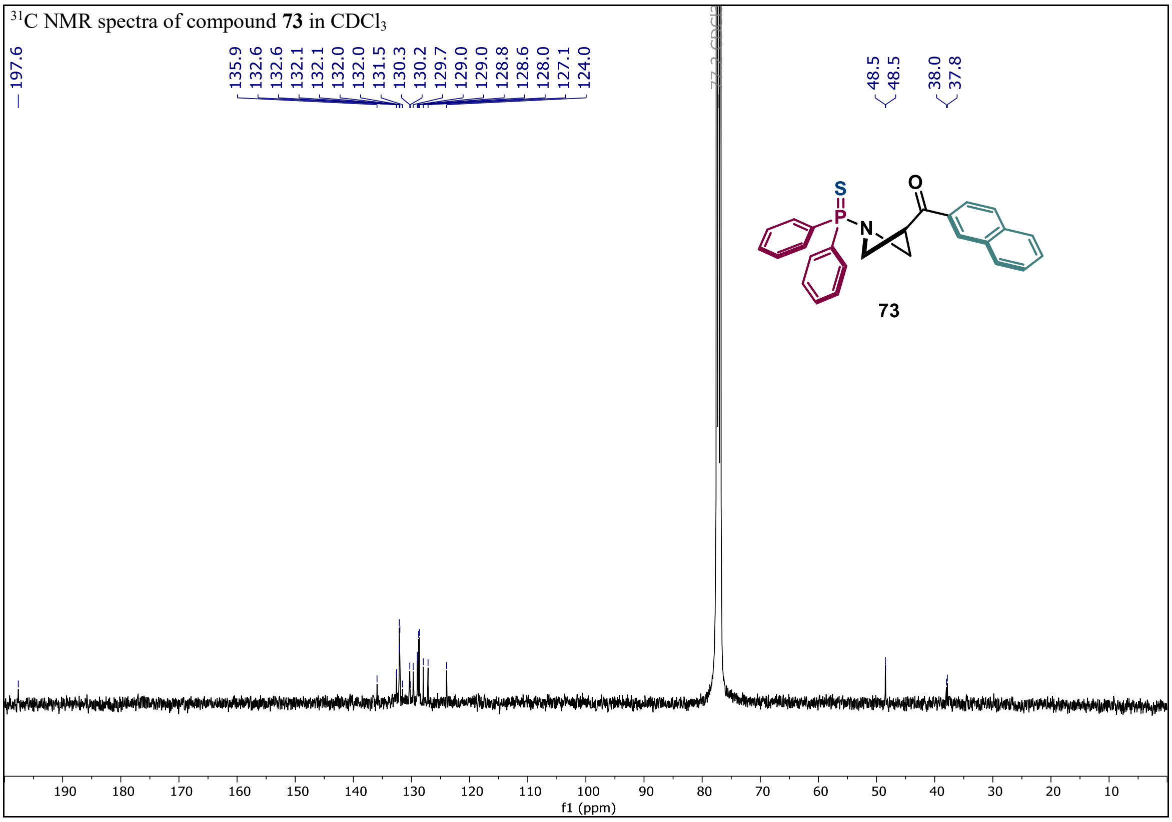

[31]C NMR spectra of compound **73** in $CDCl_3$

^{31}P NMR spectra of compound **73** in CDCl$_3$

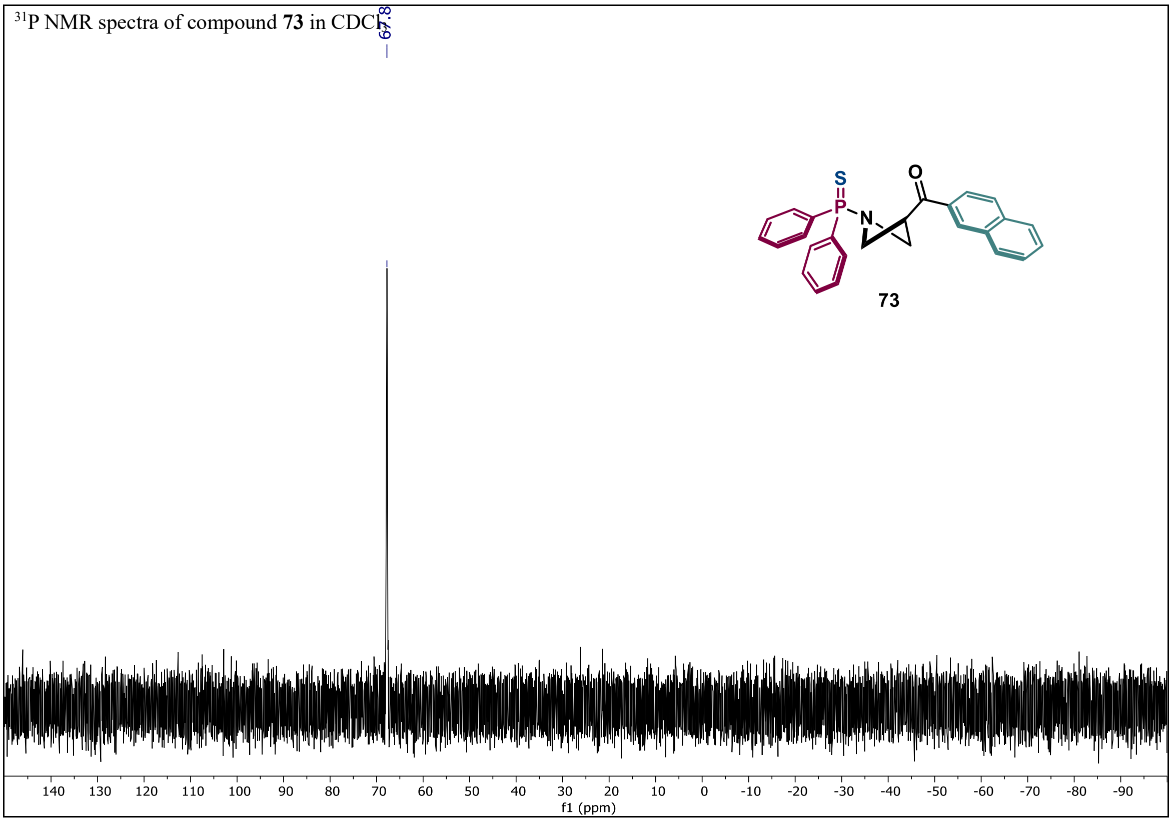

8. References

1. Henkelman, G.; Uberuaga, P. B.; Jónsson, H. A climbing image nudged elastic band method for finding saddle points and minimum energy paths. *J. Chem. Phys.* **2000**, *113*, 9901–9904.
2. Neese, F. Software update: The ORCA program system–version 5.0. *WIREs Comput Mol Sci*. **2022**, *12*, e1606.
3. Lopez, N.; Maseras, F.; Bo, C. The role of computational results databases in accelerating the discovery of catalysts. *Nat. Cat.* **2018**, *1*, 809–810.
4. A data set collection of computational results is available in the ioChem-BD repository [ioChem - BD References] and can be accessed via https://doi.org/10.19061/iochem-bd-6-627.
5. Gaussian 16, Revision C.02, Frisch, J. M.; Trucks, W. G.; Schlegel, B. H.; Scuseria, E. G.; Robb, A. M.; Cheeseman, R. J.; Scalmani, G.; Barone, V.; Petersson, A. G.; Nakatsuji, H.; Li, X.; Caricato, M.; Marenich, V. A.; Bloino, J.; Janesko, G. B.; Gomperts, R.; Mennucci, B.; Hratchian, P. H.; Ortiz, J. V.; Izmaylov, F. A.; Sonnenberg, J. L.; Williams-Young, D.; Ding, F.; Lipparini, F.; Egidi, F.; Goings, J.; Peng, B.; Petrone, A.; Henderson, T.; Ranasinghe, D.; Zakrzewski, G. V.; Gao, J.; Rega, N.; Zheng, G.; Liang, W.; Hada, M.; Ehara, M.; Toyota, K.; Fukuda, R.; Hasegawa, J.; Ishida, M.; Nakajima, T.; Honda, Y.; Kitao, O.; Nakai, H.; Vreven, T.; Throssell, K.; Montgomery, A.J.; Peralta, E. J. Jr.; Ogliaro, F.; Bearpark, J. M.; Heyd, J. J.; Brothers, N. E.; Kudin, N. K.; Staroverov, N. V.; Keith, A. T.; Kobayashi, R.; Normand, J.; Raghavachari, K.; Rendell, P. A.; Burant, C. J.; Iyengar, S. S.; Tomasi, J.; Cossi, M.; Millam, M. J.; Klene, M.; Adamo, C.; Cammi, R.; Ochterski, W. J.; Martin, L. R.; Morokuma, K.; Farkas, O.; Foresman, B. J.; and Fox, J. D. Gaussian, Inc., Wallingford CT, **2016**.
6. Chai, -D. J.; Gordon, H. M. Long-range corrected hybrid density functionals with damped atom–atom dispersion corrections. *Phys. Chem. Chem. Phys.*, **2008**, *10,* 6615–6620.
7. Weigend, F.; Ahlrichs, R. Balanced basis sets of split valence, triple zeta valence and quadruple zeta valence quality for H to Rn: design and Assessment of accuracy. *Phys. Chem. Chem. Phys.*, **2005**, *7*, 3297–3305.
8. Marenich, V. A.; Cramer, J. C.; Truhlar, G. D. Universal solvation model based on solute electron density and on a continuum model of the solvent defined by the bulk dielectric constant and atomic surface tensions. *J. Phys. Chem. B* **2009**, *113*, *18*, 6378–6396.
9. GaussView, Version 6.1.1, Dennington, R.; Keith, A. T.; Millam, M. J. Semichem Inc., Shawnee Mission, KS, **2016**.
10. Brown, C. C.; Glotzbach, C.; Stephan, W. D. Ag(I) and Au(I) complexes of sterically crowded cyclic phosphinimine ligands. *Dalton Trans*. **2010**, *39,* 9626–9632.
11. Lou, S.; Moquist, P. N.; Schaus, S. E. Asymmetric allylboration of acyl imines catalyzed by chiral diols. *J. Am. Chem. Soc.* **2007**, *129*, 49, 15398–15404.
12. Gianatassio, R. ; Lopchuk, J. M.; Wang, J. ; Pan, C. -M.; Malins, L. R.; Prieto, L.; Brandt, T. A.; Collins, M. R.; Gallego, G. M.; Sach, N. W.; Spangler, J. E.; Zhu, H.; Zhu, J.; Baran, P. S. Strain-release amination. *Science* **2016,** *351*, 241-246.
13. Hsu, C. -H.; Lin, H. -B.; Hou, X. -Z.; Tapales, V. P. P.; Shih, C. -K.; Miñoza, Z.; Tsai, Y. -S., Tsai, Z. -N.; Chan, C. -L.; Liao, H. -H. Azetidines with all-carbon quaternary centers: merging relay catalysis with strain release functionalization. *J. Am. Chem. Soc*. **2023**, *145*, 19049–19059.
14. Fawcett, A.; Murtaza, A.; Gregson, C. H. U.; Aggarwal, V. K. Strain-release-driven homologation of boronic esters: application to the modular synthesis of azetidines *J. Am. Chem. Soc*. **2019**, *141*, 4573–4578.
15. Buonomo, J. A., Eiden, C. G. & Aldrich, C. C. Scalable synthesis of hydrido-disiloxanes from silanes: a one-pot preparation of 1,3-diphenyldisiloxane from phenylsilane. *Synthesis* **2018**, *50*, 278–281.
16. Dutta, S.; Erchinger, J. E.; Strieth-Kalthoff, F.; Kleinmans, R.; Glorius, F. Energy transfer photocatalysis: exciting modes of reactivity. *Chem. Soc. Rev*. **2024**, *53*, *3*, 1068−1089.
17. Stefan, S.; Arthur, S. EasySpin, a comprehensive software package for spectral simulation and analysis in EPR. *Journal of Magnetic Resonance* **2006**, *178*, 42–55.
18. Fabrice, M. -S.; Joachim, E. K.; Frank, P.; Jean, P. F.; Jacques, L. The camphorquinone/amine and camphorquinone/amine/phosphine oxide derivative photoinitiating systems: overview, mechanistic approach, and role of the excitation light source. *Macromol. Chem. Phys.* **2015**, *216*, 2161−2170.
19. Khrizanforova, V. V.; Kholin, K. V.; Khrizanforov, M. N.; Kadirov, M. K.; Budnikova, Yu. H. Electrooxidative CH/PH functionalization as a novel way to synthesize benzo[b]phosphole oxides mediated by catalytic amounts of silver acetate. *New J. Chem*., **2018**, *42*, 930–935.
20. Ludovik, N. -D.; Elodie, L.; Fabrice, M. -S.; Jean, -F. L.; Isabelle, C.; Martin, Breugst.; Jacques, Lalevee.; Annie, -C. Gaumont.; Sami, L. Metal-free synthesis of 6-phosphorylated phenanthridines: synthetic and mechanistic insights. *Org.*

Lett. **2016**, *18*, 5900−5903.

21. Jacques, L.; Fabrice, M. -S.; Mohamad, A. T.; Bernadette, G.; Jean, P. F. Photosensitized formation of phosphorus-centred radicals: application to the design of photoinitiating systems. *Macromolecules* **2012**, *45*, *12*, 5032–5039.
22. Liu, J.; Xiao, H. Z.; Fu, Q.; Yu, D. G. Advances in radical phosphorylation from 2016 to 2021. *Chem. Synth.* **2021**, *1*, 9.
23. Zhang, M.; Xie, J.; Zhu, C. A General deoxygenation approach for synthesis of ketones from aromatic carboxylic acids and alkenes. *Nat. Commun.* **2018**, *9*, 3517.
24. Murov. L. S.; Carmichael, I.; Hug, G. Handbook of Photochemistry, Second Edition. *Inc., New York* **1973**, 124.
25. Kuhn, H. ; Braslavsky, S.; Schmidt, R. Chemical actinometry. *Pure Appl. Chem*. **2004**, *76*, 2105-2146.
26. Gonzalez, C.; Schlegel, B. H. Reaction path following in mass-weighted internal coordinates. *J. Phys. Chem.* **1990**, *94*, *14*, 5523–5527.
27. Hratchian, P. H.; Schlegel, B. H. Using hessian updating too increase the efficiency of a hessian based predictor-corrector reaction path following method. *J. Chem. Theory Comput.* **2005**, *1*, *1*, 61–69.
28. Peng, C.; Schlegel, B. H. Combining synchronous transit and quasi-Newton methods to find transition states. *Isr. J. Chem.* **1993**, *33*, 449– 454.
29. Besler, H. B.; Merz Jr, M. K; Kollman, A. P.; *J. Comput. Chem.* **1990**, *11*, *4*, 411-544.
